# Theoretical Analysis of Coordination Geometries in Transition Metal–Histidine Complexes Using Quantum Chemical Calculations

**DOI:** 10.3390/molecules29133003

**Published:** 2024-06-25

**Authors:** Dapeng Zhang, Naoki Kishimoto

**Affiliations:** Department of Chemistry, Graduate School of Science, Tohoku University, 6-3, Aoba, Aramaki, Aoba-ku, Sendai 980-8578, Japan; zhang.dapeng.c5@tohoku.ac.jp

**Keywords:** transition metal amino acid complex, conformational search, coordination formation

## Abstract

A theoretical investigation utilizing density functional theory (DFT) calculations was conducted to explore the coordination complexes formed between histidine (His) ligands and various divalent transition metal ions (Mn^2+^, Fe^2+^, Co^2+^, Ni^2+^, Cu^2+^, and Zn^2+^). Conformational exploration of the His ligand was initially performed to assess its stability upon coordination. Both 1:1 and 1:2 of metal-to-ligand complexes were scrutinized to elucidate their structural features and the relative stability of the complexes. This study examined the ability of His to act as a bidentate or tridentate coordinating ligand, along with the differences in coordination geometry when solvent effects were incorporated. The reduced density gradient (RDG) analysis and local electron attachment energy (LEAE) analysis were employed to elucidate the interaction planes and the nucleophilic and electrophilic properties. The electronic properties were analyzed through electrostatic potential (ESP) maps and natural population analysis (NPA) of atomic charge distributions. This computational study provides valuable insights into the diverse coordination modes of His and its interactions with divalent transition metal ions, contributing to a better understanding of the role of this amino acid ligand in the formation of transition metal complexes. The findings can aid in the design and construction of self-assembled structures involving His-metal coordination.

## 1. Introduction

Transition metal amino acid complexes are highly diverse compounds with extensive applications in mimicking bio-related structures or functions and artificially constructing self-assembled systems [1,2,3]. These complexes, formed through the metal–ligand interactions of metal centers with functional groups in the amino acids, exhibit flexible conformations governed by weak interactions, including coordination and intramolecular and intermolecular hydrogen bonds [4,5]. Understanding and leveraging the structural features of amino acids are paramount for constructing self-assembled complexes. This process is influenced by various factors, including the choice of metal centers, the positioning of functional groups, the manner in which intra-/intermolecular hydrogen bonds are formed, as well as the surrounding environmental conditions such as the distributions of the counter anions, solvents, and water molecules [6,7]. Achieving a targeted metal–ligand complex presents challenges in the rational design and control of a bottom-up approach to assembling building blocks while simultaneously managing undesired pathways that may lead to irrelevant interactions and the formation of distinct intra- and intermolecular intermediates [8,9,10,11]. The inherent flexibility exhibited by amino acids emphasizes the significance of theoretical investigations into their interactions with metal centers and the ensuing alterations stemming from conformational changes. Such investigations are pivotal for comprehending the versatility and potential properties of these ligands, thereby facilitating the artificial design of sophisticated systems.

Quantum chemical calculations have proven indispensable for elucidating the stabilities, structural features, and bonding properties of amino acid–metal complexes, furnishing detailed molecular-level insights into the nuanced interplay between ligand flexibility and metal coordination [12,13,14]. In recent years, density functional theory (DFT) calculations have been extensively employed to scrutinize isomerization phenomena in amino acids [15,16], delineating their dynamic behavior in the presence of surrounding explicit water molecules [17,18], and predict the relative stability and coordination preferences of metal ions in complex formations with more sophisticated structures such as tripeptides [19]. The theoretical knowledge garnered from quantum chemical investigations paves the way for the rational design of artificial molecular architectures with tailored properties for various applications.

The choice of amino acid ligands and metal ions plays a crucial role in determining the coordination structure. Common amino acids such as glycine (Gly), histidine (His), cysteine (Cys), alanine (Ala), and tyrosine (Tyr) complexed with biologically relevant metal ions like Fe(II), Cu(II), and Zn(II) are of particular interest [20,21,22,23,24,25,26]. These systems serve as model platforms for unraveling the underlying interactions and exploring the potential applications of these versatile ligands in the context of artificial self-assembled molecular systems. Histidine features an imidazole side chain in addition to carboxyl and amino groups, providing further coordination sites with metal ions. Importantly, the proton transfer and tautomerization process exhibited in the histidine structure [27,28] enables the formation of a diverse array of functional binding sites. This facilitates access to more intricate chelate rings with the metal center, potentially imparting unique structures and properties to the resulting complexes. Consequently, an in-depth examination of the conformational changes in histidine is imperative to elucidate the stable conformations it can adopt. In this study, we employed an efficient conformational exploration algorithm with high accuracy to investigate the reaction network associated with conformational changes. The most stable structure identified and its tautomer were subsequently coordinated with various metal ions, and their geometries were compared with the corresponding coordination configurations. This comparative analysis facilitated the evaluation of structural alterations and structural stability resulting from coordination for different metal centers upon complexation with amino acid ligands.

Histidine possesses the capability to act as a tridentate ligand with various metal ions, thereby facilitating the formation of an octahedral coordination geometry when employed in dimeric model complexes. However, when two histidine residues coordinate with a single metal center, a scenario can arise where both bidentate and tridentate coordination modes coexist simultaneously [29]. This situation is particularly relevant in the case of Cu(II) complexes [30]. When evaluating the potential for the formation of tridentate coordination geometries, it becomes crucial to concurrently examine the viability of octahedral structural motifs involving histidine ligands, as well as the influence of the central charge and the nature of the metal center. Herein, we focused our investigation on divalent transition metal cations, including Mn(II), Fe(II), Co(II), Ni(II), Cu(II), and Zn(II). For the metal ions Mn, Fe, and Co, both high-spin (HS) and low-spin (LS) states were examined to elucidate the impact of diverse spin-state configurations on the propensity to facilitate different coordination structures. This comprehensive approach enables a thorough understanding of the factors governing the formation of metal complexes with histidine-based ligands and the associated structural motifs.

## 2. Results and Discussion

### 2.1. Conformational Changes and Coordination Sites of Histidine

**Figure 1:**
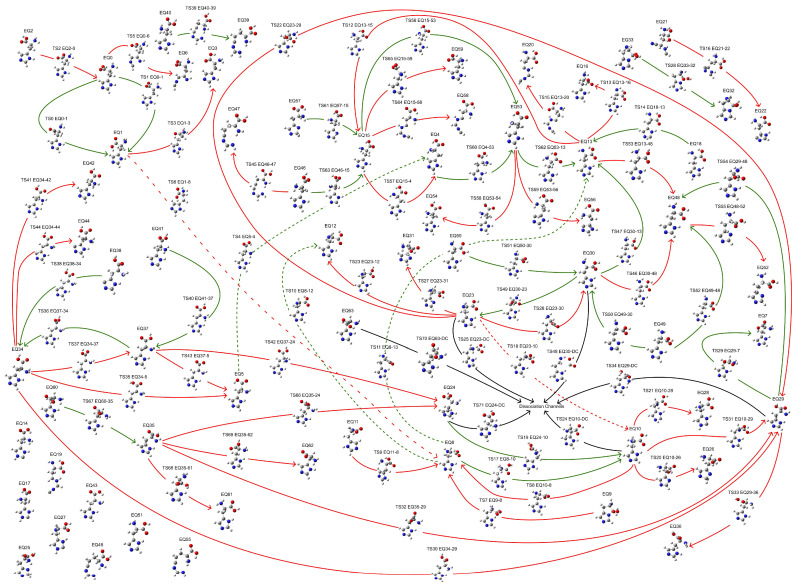
Reaction network illustrating conformational changes of a His molecule. Red pathways indicate routes from a stable EQ structure toward a less stable EQ structure, while green pathways indicate routes to a more stable EQ structure. TS*n* EQ*r*-*p* indicates the *n*th TS structure from EQ*r* to EQ*p*. For simplicity, dashed lines represent pathways with the same meaning as solid lines.

Exploring the potential energy surface (PES) is crucial for obtaining energetically reasonable molecular structures. Herein, a fully automatic and efficient conformational exploration method, the anharmonic downward distortion following (ADDF) algorithm [31,32,33,34], was employed to optimize the structure of His at the B3LYP/6-31G* level of theory [35,36,37,38,39,40,41,42,43,44,45,46,47,48,49] using the global reaction route mapping (GRRM) program [50,51] interfaced with Gaussian 16 [52]. A limited PES exploration was conducted, tracing the seven largest anharmonic downward distortions (*l-ADDF7*) and related pathways surrounding equilibrium (EQ) structures while considering only the 10 lowest local minima (*NLowest* = 10). This restricted exploration identified 64 EQ structures, 72 transition state (TS) structures, and structures within the dissociation channel (DC), collectively forming a potential reaction network that describes the conformational changes, as shown in Figure 1. Although a comprehensive search for global reaction routes is feasible with the ADDF algorithm in the GRRM program, it demands significantly more computational resources. For the present study, aimed at determining a stable conformation of histidine, the limited search proved sufficient. The most stable structure found in the exploration (EQ 13, a π-His structure) was further optimized employing the B3LYP-D3(BJ)/def2-TZVP level of theory [53,54,55,56] using the GRRM program interfaced with Gaussian 16. The B3LYP functional, augmented with Grimme’s dispersion correction (D3) and the Becke–Johnson damping, accounts for the dispersion interactions that the basic B3LYP functional fails to capture, thereby enabling an accurate and reliable description of structures governed by weak non-covalent interactions [57,58,59]. This approach provides a robust framework for correctly representing the coordination and hydrogen bonding interactions prevalent in metal–ligand complexes. Moreover, the polarizable continuum model (PCM) [60] and the solvation model based on density (SMD) [61] were incorporated into the optimized models to account for the solvation effects. The calculations were performed in an aqueous environment (ε = 78.39). In addition to the conformational exploration, all model calculations were conducted at 298.15 K.

**Figure 2:**
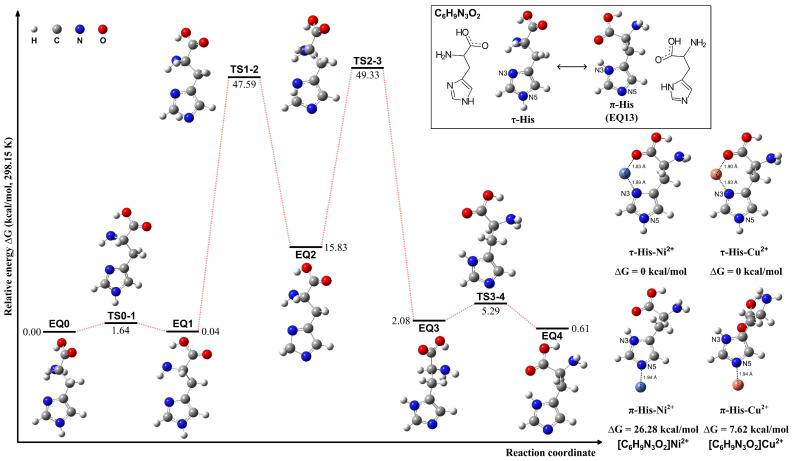
Structures of the τ- and π-tautomeric forms of the His molecule, possible pathways for their interconversion, and the exemplified complexes of τ-His and π-His coordinating with Ni^2+^ and Cu^2+^ ions, respectively.

The τ-His tautomer was optimized based on the conformation of the π-His structure (EQ 13). The tautomerization pathways between τ- and π-His tautomers were calculated employing the sphere contraction walk (SCW) [62] method and the two-point scaled hypersphere search (2PSHS) [63] method implemented in the GRRM program. The SCW method WAS utilized to find intermediate structures between two minima on the PES, while the 2PSHS method performs double-ended saddle-point optimization to find a TS structure connecting two EQ structures. The calculations were conducted in a vacuum at 298.15 K. The structures and the interconversion pathways are given in Figure 2. The proton tautomerism between the N3 and N5 atoms of the imidazole ring results in nearly isoenergetic π-His and τ-His structures. However, upon metal ion coordination, the τ-His tautomer exhibits a distinct advantage. The N3 atom in τ-His is optimally positioned to form bidentate or tridentate chelation motifs by engaging proximal carboxylate and amino groups, facilitating tighter binding to the metal center. This can be confirmed in the exemplified complexes of Ni^2+^ and Cu^2+^ ions, where the metal is coordinated by either the N3 atom or N5 atom. When τ-His acts as a bidentate ligand, it is more stable than the analogous N5 coordination observed for the π-His tautomer.

### 2.2. Coordination Modes of His Ligand to Metal Centers ([ML]^2+^)

Divalent transition metal ions, including Mn^2+^, Fe^2+^, Co^2+^, Ni^2+^, Cu^2+^, and Zn^2+^, were investigated as coordination centers (M) with the functional groups from one or two τ-His ligands (L), which can potentially act as bidentate or tridentate ligands. The feasibility of forming an octahedral coordination geometry was investigated through model calculations. The His ligand provided three coordinating sites, while three water molecules were incorporated to satisfy the remaining coordination sites and achieve the targeted octahedral geometry. Incorporating water molecules into coordination with transition metal ions, such as Mn^2+^, Fe^2+^, and Co^2+^, leads to the formation of octahedral coordination with His ligands. However, challenges arise with Cu^2+^ and Zn^2+^ ions due to the dissociation of carboxyl groups from coordination, as illustrated by the results presented in Supporting Information Figure S1 and Table S1. The reduced density gradient (RDG) analysis [64] using the Multiwfn program [65] revealed relatively weak interactions between the O1 atom and the Cu^2+^ or Zn^2+^ center, as depicted in Supporting Information Figure S2. This highlights the intricate nature of treating transition metal ions with consideration of weak interactions from solvents, where the coordination mode for ligands can be altered.

For Mn^2+^, Fe^2+^, and Co^2+^, computational models were constructed to explore their HS and LS states. In the 1:1 or 1:2 metal–ligand complex models, tridentate coordination with a stable octahedral geometry formed upon chelation of dual His ligands to the metal center was considered a favorable interaction mode. However, if an additional His ligand interacted with the metal center, preventing the formation of the octahedral coordination (also including the distorted octahedral coordination) arising from two tridentate binding sites, the tridentate coordination was deemed unstable. In such cases, the His ligand likely reverted to a bidentate-binding motif. This systematic approach enabled an efficient evaluation of the potential coordination geometries and coordination stabilities accessible to these transition metal ions with His-based ligands. In this study, the His ligand coordination mode, whether tridentate or bidentate, was determined by the atomic distances. Distances less than 2.50 Å indicated a tridentate function, while longer distances indicated a bidentate motif. In addition, our investigation encompassed not only the model calculations for [ML]^2+^ and [ML_2_]^2+^ complexes but also the deprotonated His ligand (the -COOH group is deprotonated), denoted as [M(L-H)]^+^ and [M(L-H)_2_]. To investigate the influence of solvation effects on the coordination geometries, all the models were compared to their respective counterparts incorporating the PCM or the SMD. The structures optimized using the PCM model are denoted by appending ‘_PCM’ to the complex name, while those optimized with SMD are denoted by appending ‘_SMD’ to the complex name.

**Figure 3:**
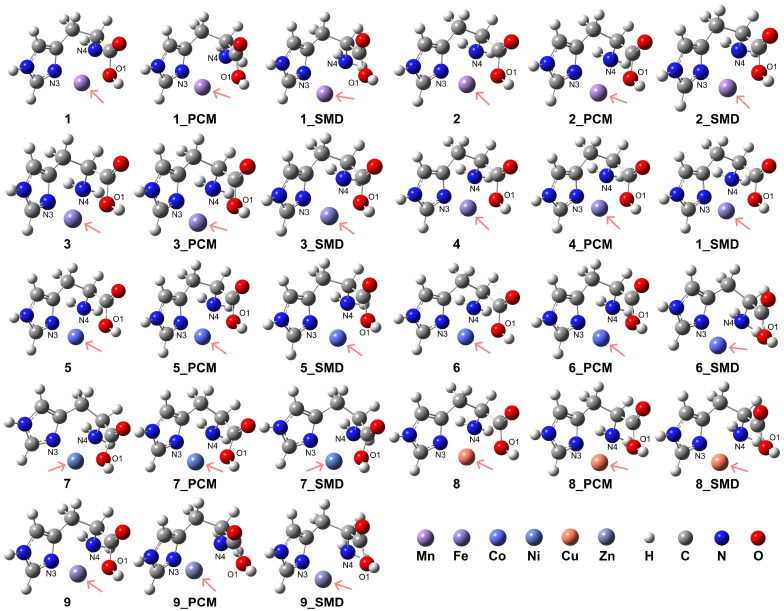
Model complexes, [ML]^2+^, formed by the His ligand coordinating in a bidentate or tridentate mode with a divalent transition metal center. The pink arrows indicate the position of the metal center: Mn (complex **1** for HS state and complex **2** for LS state), Fe (complex **3** for HS and complex **4** for LS), Co (complex **5** for HS and complex **6** for LS), Ni (complex **7**), Cu (complex **8**), and Zn (complex **9**).

The model complexes for [ML]^2+^ are depicted in Figure 3, with computational details, coordination modes of the ligand, relative energies, and key atomic distances provided in Table 1. The formation of these model complexes in aqueous conditions exhibited higher stability compared to vacuum conditions. Furthermore, the HS state complexes of Mn^2+^, Fe^2+^, and Co^2+^ demonstrated enhanced stability relative to their respective LS state counterparts. Notably, the results indicate that the presence of an aqueous medium facilitated the formation of these coordination complexes. The coordination of the His ligand adopts a tridentate mode for both the HS and LS states of Mn^2+^ under vacuum conditions (complexes **1** and **2**). However, the introduction of solvation effects reveals weak interactions between the O1 atom and the Mn^2+^ center in the HS state, suggesting a preference for bidentate coordination involving the N3 atom of the imidazole side chain and the N4 atom of the amino group. In this bidentate mode, the O1 atom is situated at distances of 2.98 Å and 3.00 Å from the Mn^2+^ center when employing the PCM and the SMD, respectively. For the LS state of Mn^2+^, although tridentate coordination is formed, the interactions between the O1 atom and the Mn^2+^ center are similarly weak, corroborated by the longer distances of 2.23 Å and 2.32 Å obtained from the PCM and SMD models, respectively.

For complexes **1** and **2** in the vacuum condition, the RDG analysis revealed an intense interaction between the O1 atom of the His ligand and the Mn^2+^ center, as well as between O1 and the N4 atom, in contrast to the interaction with the N3 atom of the imidazole ring (see Supporting Information Figure S3). When the PCM model was applied, the interaction between O1 and the metal center was not observed. However, upon applying the SMD, despite the atomic distance between O1 and the metal center exceeding 2.5 Å, weak interactions were evident between O1 and the metal center, as well as between O1 and the N4 atom. This suggests that the interaction with the Mn^2+^ ion may still persist even at larger atomic separations.

In contrast, for Fe^2+^ (complexes **3** and **4**), the tridentate coordination mode persists in both HS and LS states. However, the introduction of solvation effects weakens the interactions in the HS state, with the O1 atom exhibiting atomic distances of 2.37 Å and 2.33 Å from the Fe^2+^ center when employing the PCM and the SMD, respectively. These elongated distances suggest a propensity toward a bidentate coordination mode, departing from the tridentate arrangement observed under vacuum conditions. Similarly, the HS state Co^2+^ complexes **5** and **6** maintain a tridentate coordination mode for the His ligand, albeit with weakened interactions in aqueous conditions. In these solvated systems, the atomic distances between the O1 atom and the Co^2+^ center are 2.36 Å and 2.33 Å when employing the PCM and the SMD, respectively. While the LS state Co^2+^ complexes exhibit a preference for tridentate coordination in vacuum and under the PCM model, the inclusion of solvation effects induces weaker interactions between the carboxyl group and the Co^2+^ center, with increased distances of 2.42 Å for the PCM solvation models. This weakening is particularly highlighted by the increased distances of 2.96 Å between the O1 atom and the Co^2+^ center for the SMD solvation model, which suggests a bidentate coordination mode. The RDG analysis for complexes **3**, **4**, **5**, and **6** revealed intense non-covalent interactions between the O1 atom and the metal center, as well as between the O1 atom and the N4 group (see Supporting Information Figure S3). The inclusion of solvation effects, modeled using the PCM model or the SMD, intensified these interactions. However, for complexes **5** and **6**, the interactions appeared less prominent when employing the SMD compared to the PCM model, suggesting a potential influence of the solvation model on the patterns of weak interactions.

Ni^2+^ complex **7** exhibits a stable tridentate coordination configuration for the His ligand in both vacuum and aqueous environments. In contrast, for Cu^2+^ ions (complex **8**), the introduction of solvation effects favors a bidentate coordination mode, as evidenced by the increased atomic distance of 3.03 Å between the O1 atom and the Cu^2+^ center, as confirmed by the SMD. Even when employing the PCM, the atomic distance between O1 and Cu^2+^ is relatively long at 2.35 Å, indicating a weak interaction. This behavior suggests a propensity for bidentate coordination in solvated Cu^2+^ complexes, deviating from the tridentate arrangement observed in the vacuum state. In addition, Zn^2+^ complex **9** exhibits a stable tridentate coordination mode for the His ligand under vacuum conditions. However, in aqueous environments, a preference for bidentate coordination emerges, suggesting a solvent-induced shift in the coordination occurrence. This propensity for bidentate coordination is evidenced by the elongated atomic distances of 3.37 Å and 3.15 Å between the O1 atom and the Zn^2+^ center when employing the PCM and the SMD, respectively. These increased distances indicate a weakening of the O1-Zn^2+^ interaction in the solvated systems, facilitating the transition from a tridentate to a bidentate coordination geometry.

The RDG analysis indicates that for complexes **7** and **8**, non-covalent interactions coexist with similar intensities under vacuum conditions (see Supporting Information Figure S3). However, when solvation effects were considered, the weak interaction between the O1 atom and the N4 atom combined with the interaction between O1 and the metal center. In the case of complex **8**, while this interaction persists when applying the PCM model, its intensity is relatively lower when the SMD is applied. This reduced interaction intensity may stem from the elongated distance between O1 and the Cu^2+^ center in complex **8**. In vacuum conditions, weak interactions within complex **9** show similar intensities, with the persistence of the weak interaction between O1 and N4 atoms. However, solvent effects caused the interaction between O1 and the metal center to vanish, indicating a potential restriction of coordination distances with the Zn^2+^ ions to relatively short distances.

### 2.3. Coordination Modes of Two His Ligands to Metal Centers ([ML_2_]^2+^)

**Figure 4:**
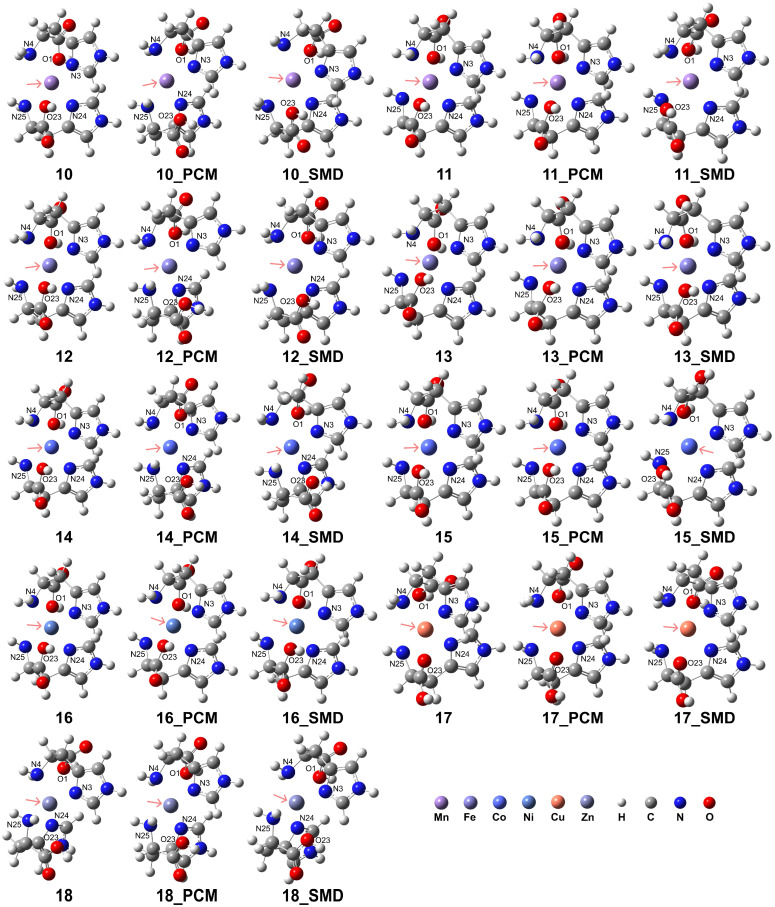
Model complexes, [ML_2_]^2+^, formed by two His ligands coordinating with a divalent transition metal center. The pink arrows indicate the position of the metal center: Mn (complex **10** for HS state and complex **11** for LS state), Fe (complex **12** for HS and complex **13** for LS), Co (complex **14** for HS and complex **15** for LS), Ni (complex **16**), Cu (complex **17**), and Zn (complex **18**).

The computational results indicate that the coordination mode of the His ligand is influenced by the metal spin states and the solvation environment. Our results confirmed that the formation of these complexes in aqueous environments further augmented their stability. The results are presented in Figure 4, and detailed information regarding the coordination is provided in Table 2. The RDG analysis (see Supporting Information, Figure S4) revealed that the carboxyl group exhibits a more pronounced interaction with the metal center, whereas the N atoms in the imidazole ring demonstrate a less intense interaction with the metal center. In the case of complexes **10**, **12**, **14**, and **17**, the incorporation of solvation models can disrupt the interactions between the carboxyl group and the metal center. Analogous intense interactions were observed for complexes **11**, **13**, and **15**. However, the interaction between the ligand and the metal center in complex **18** was considerably weaker.

The HS state complexes of Mn^2+^, Fe^2+^, and Co^2+^ exhibited enhanced stability relative to their respective LS state counterparts. For the HS state of Mn^2+^, the His ligands adopted a bidentate coordination mode due to the elongated atomic distances of 3.50 Å and 3.52 Å for O1 and O23 atoms to the Mn^2+^ center, respectively, in the PCM. In contrast, the elongated distances of 2.96 and 3.13 Å obtained using the solvation model of the model of SMD suggest a tridentate coordination. For the LS state of Mn^2+^, both vacuum and PCM results exhibit distorted octahedral coordination, whereas the calculations with SMD indicate dissociation of the O1 and O23 atoms from the coordination sphere, with distances of 3.02 and 3.01 Å, respectively. In the case of HS Fe^2+^, distorted octahedral coordination is observed in a vacuum. At the same time, solvation effects weaken the interaction of the Fe^2+^ ion with the His ligand, as evidenced by the longer O1-Fe^2+^ and O23-Fe^2+^ distances of 3.46 and 3.39 Å in the PCM model and 3.10 and 3.09 Å in the solvation model of SMD, which favored a bidentate mode. Conversely, the LS Fe^2+^ exhibits a stable tridentate coordination with an octahedral geometry. For HS Co^2+^, octahedral coordination is observed in a vacuum, but in aqueous environments, the O1-Co^2+^ and O23-Co^2+^ distances elongate to 3.46 and 3.48 Å (PCM) and 3.12 Å and 3.41 Å (SMD), indicating a preference for the His to act as a bidentate ligand. Although the LS Co^2+^ can form distorted octahedral complexes in vacuum or PCM solvation, the interaction of the Co^2+^ ion with the carboxyl group is weak, as evidenced by the elongated O1-Co^2+^ and O23-Co^2+^ distances, suggesting that a bidentate mode for the His ligands is more favorable. The His ligands act as a stable tridentate ligand for Ni^2+^, forming distorted octahedral complexes in both vacuum and aqueous environments. However, for Cu^2+^ and Zn^2+^, the elongated O1-M^2+^ and O23-M^2+^ distances observed in vacuum and aqueous solvents confirm a bidentate coordination mode in which the carboxyl groups are dissociated from the metal center.

### 2.4. Coordiantion Modes of Deprotonated His Ligand to Metal Centers ([M(L-H)]^+^)

**Figure 5:**
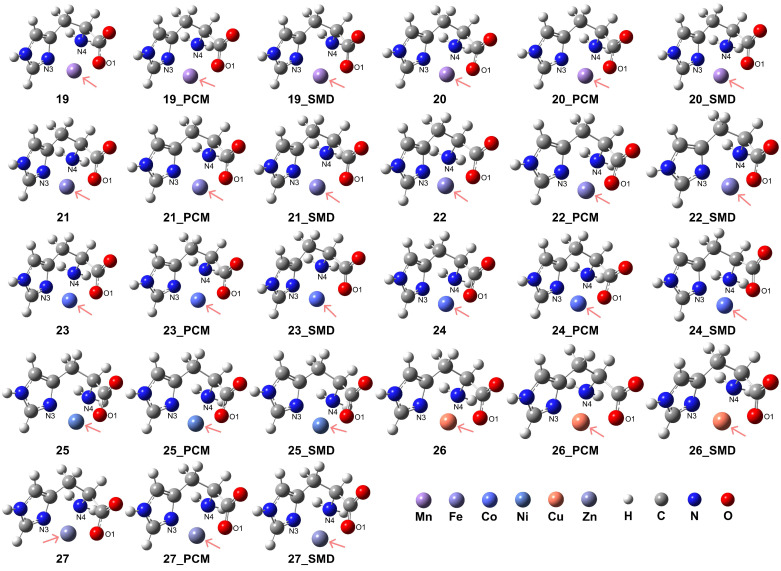
Model complexes, [M(L-H)]^+^, formed between a deprotonated His ligand and various divalent transition metal centers. The metal centers are indicated by pink arrows: Mn (complex **19** for HS state and complex **20** for LS state), Fe (complex **21** for HS and complex **22** for LS), Co (complex **23** for HS and complex **24** for LS), Ni (complex **25**), Cu (complex **26**), and Zn (complex **27**).

The carboxylate group (-COO^−^) can form stable coordination with the divalent metal centers, resulting in the formation of stable tridentate coordination. This is confirmed in our model complexes in which the His ligand is confirmed to play a role as a stable tridentate ligand coordinating with the examined metal ions, shown in the complex structures 19–27 in Figure 5 and the details of coordination configurations in Table 3. The presence of an aqueous medium promoted the formation of stable coordination complexes. For the transition metal ions Mn^2+^, Fe^2+^, and Co^2+^, the HS state complexes exhibited superior stability when compared to their corresponding LS state analogues. 

The RDG analysis for complexes **19** and **20** under vacuum conditions reveals the coexistence of non-covalent interactions with similar intensities (see Supporting Information Figure S5). However, upon considering solvation effects, the weak interaction between the O1 atom and the N4 atom became coupled with the interaction between O1 and the metal center. Similar coupled interactions were observed for complexes **21** and **22**, while for complexes **23** and **24**, these interactions were intensified. In complexes **25**, **26**, and **27**, weak interactions were detected with comparable intensities among the O1 atom, N4 atom, and N3 atom with the metal center.

### 2.5. Coordiantion Modes of Two Deprotonated His Ligands to Metal Centers ([M(L-H)_2_])

**Figure 6:**
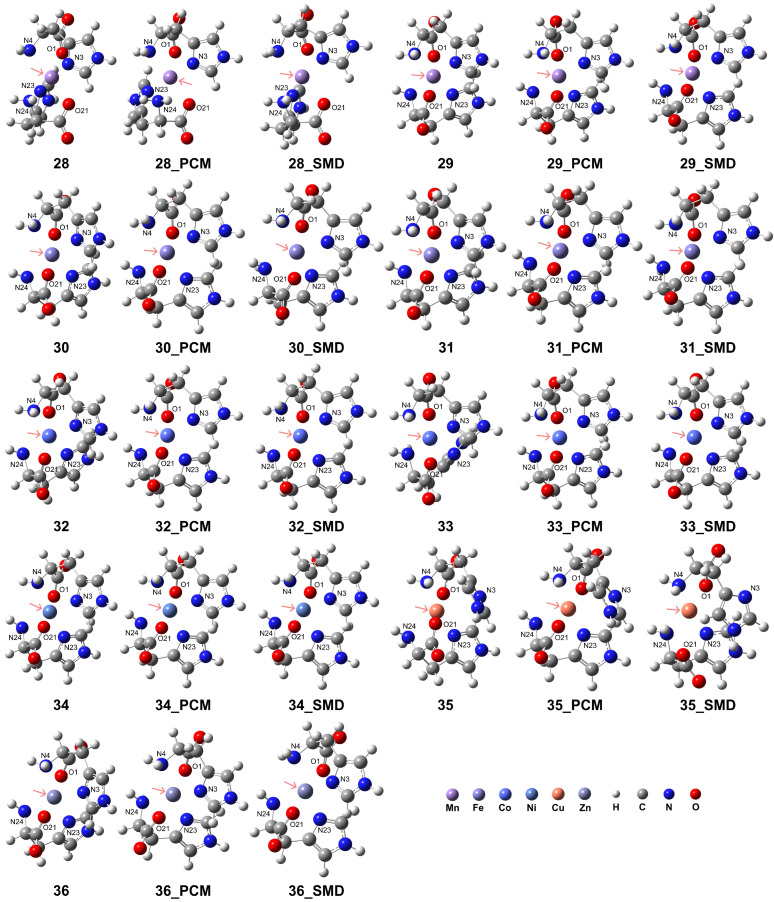
Model complexes, [M(L-H)_2_], formed by two deprotonated His ligands coordinating with a divalent transition metal center. The pink arrows indicate the position of the metal center: Mn (complex **28** for HS state and complex **29** for LS state), Fe (complex **30** for HS and complex **31** for LS), Co (complex **32** for HS and complex **33** for LS), Ni (complex **34**), Cu (complex **35**), and Zn (complex **36**).

While the [M(L-H)]^+^ complexes exhibited tridentate coordination configurations, the His ligands as tridentate sites can form distinct octahedral coordination geometries in the [M(L-H)_2_] complexes. All examined transition metal ions, in addition to Cu^2+^, can form distorted coordination geometries with two His ligands. The structures are shown in Figure 6, and detailed geometries are presented in Table 4. The RDG analysis for complexes **28**–**34** and **36** revealed that the interactions between N4 and N24 atoms, or between O1 and O21 atoms in complexes **28**–**34**, or both, are more intense compared to those involving the N3 and N23 atoms in the imidazole ring (see Supporting Information Figure S6). However, complex **35** exhibits different properties, with the N atoms in the imidazole ring showing more intense interactions with the metal center. To further analyze the properties of the transition metal-His complexes, the electrostatic potential (ESP) maps (with the van der Waals radii in ref [66]) and natural atomic charges derived from natural population analysis (NPA) for the complexes in vacuum conditions are provided in Supporting Information Figures S7 and S8 and Table S2, respectively. The calculations were conducted at the B3LYP-D3(BJ)/def2-TZVP level of theory. After coordination, the nucleophilic regions are located near the carboxyl groups. The local electron attachment energy (LEAE) analysis [67], performed using the Multiwfn program, was employed to determine the electrophilic regions. The results revealed that for complexes **26** and **28**, the electrophilic regions were localized around the nitrogen atom of the amino group. In contrast, for the remaining model complexes, the electrophilic regions were distributed surrounding the nitrogen atom within the imidazole ring (see Supporting Information Figure S9). For Mn^2+^, Fe^2+^, and Co^2+^ ions, the metal charge was significantly reduced for the LS complexes compared to the HS complexes, with values of 1.061 and 0.313 for HS Mn^2+^ and LS Mn^2+^, respectively; 0.983 and 0.224 for HS Fe^2+^ and LS Fe^2+^, respectively; and 0.919 and 0.617 for HS Co^2+^ and LS Co^2+^, respectively. Additionally, the charge of the metal center is reduced more substantially for Ni and Cu compared to Zn, with values of 0.856, 0.961, and 1.239, respectively.

For the HS state Mn^2+^ (complex **28**), different types of distorted octahedral coordination geometries were observed. In a vacuum, coordination was formed with an O1-Mn-N24 angle of 159.52°, an N3-Mn-N23 angle of 171.06°, and an N4-Mn-O21 angle of 159.50°, causing the formation of a distorted octahedral geometry. The solvation effect leads to elongated metal–ligand distances and modifies the coordination geometry. With the PCM model, the coordination exhibits an O1-Mn-N24 angle of 167.27°, an N3-Mn-N23 angle of 179.19°, and an N4-Mn-O21 angle of 167.11°, making this structure close to octahedral geometries. In the SMD results, the structure exhibits a distorted octahedral coordination with an O1-Mn-N24 angle of 157.05°, an N3-Mn-N23 angle of 158.15°, and an N4-Mn-O21 angle of 159.00°, representing a distinct coordination geometry with elongated atomic distances while forming a distorted octahedral geometry. 

For the LS state of Mn^2+^ (complex **29**), smaller metal–ligand distances were confirmed compared to the HS states. In a vacuum, the distorted geometry had an O1-Mn-O21 angle of 173.33°, an N3-Mn-N24 angle of 170.15°, and an N4-Mn-N23 angle of 170.17°, which are close to octahedral coordination and differ from the configuration in the HS state. In the PCM model, the distorted geometry had an O1-Mn-O21 angle of 172.89°, an N3-Mn-N24 angle of 172.19°, and an N4-Mn-N23 angle of 172.15°. In the SMD model, these values were an O1-Mn-O21 angle of 173.53°, an N3-Mn-N24 angle of 171.76°, and an N4-Mn-N23 angle of 171.74°. Since in the LS state, the dihedral angles are closer to planar, the distortions are smaller compared to those in the HS state, and it can be considered that the LS state is capable of generating more stable coordination conformations. 

Two His ligands coordinating with Fe^2+^ resulted in different octahedral geometries with shorter bond distances in the HS and LS states compared to those of Mn^2+^, respectively. In the vacuum condition with HS Fe^2+^ (complex **30**), the coordination formed with an O1-Fe-O21 angle of 177.25°, an N3-Fe-N24 angle of 163.38°, and an N4-Fe-N23 angle of 163.46°. This geometry changed to another octahedral coordination with an O1-Fe-O21 angle of 175.47°, an N3-Fe-N24 angle of 167.66°, and an N4-Fe-N23 angle of 168.04° when applying the PCM model. A large distortion in coordination configuration was confirmed when employing the SMD, with an O1-Fe-O21 angle of 175.34°, an N3-Fe-N24 angle of 161.20°, and an N4-Fe-N23 angle of 163.36°. This indicates that the His ligands coordinating with the HS state of Fe^2+^ may cause distinct coordination modes, and it is challenging to maintain conformational stability when changing the solution conditions. 

In contrast, for the LS state of Fe^2+^ (complex **31**), the coordination had an O1-Fe-O21 angle of 176.32°, an N3-Fe-N24 angle of 170.89°, and an N4-Fe-N23 angle of 170.91°, making it closer to octahedral coordination. After the addition of the PCM model, the coordination exhibited an O1-Fe-O21 angle of 175.62°, an N3-Fe-N24 angle of 172.58°, and an N4-Fe-N23 angle of 172.66°. When applying the aqueous environments based on the SMD, the values were an O1-Fe-O21 angle of 176.06°, an N3-Fe-N24 angle of 172.65°, and an N4-Fe-N23 angle of 172.71°. For the Fe^2+^ ions, these dihedral angles were close to a planar, and the angles throughout the metal center were approximately 180 degrees, rendering them more similar to octahedral coordination.

Coordinating with Co^2+^ exhibited geometries similar to those observed in the Fe^2+^ complexes. These Co^2+^ complexes also showed shorter bond distances in the HS and LS states compared to those of Mn^2+^, respectively. In the vacuum condition for the HS state of Co^2+^ (complex **32**), the coordination exhibited an O1-Co-O21 angle of 171.28°, an N3-Co-N24 angle of 166.30°, and an N4-Co-N23 angle of 166.28°, resembling a distorted octahedral geometry. The distortion became less pronounced when applying the PCM model, with an O1-Co-O21 angle of 169.99°, an N3-Co-N24 angle of 170.60°, and an N4-Co-N23 angle of 170.82°. In the model complexes with the SMD, an O1-Co-O21 angle of 173.18°, an N3-Co-N24 angle of 169.99°, and an N4-Co-N23 angle of 168.33° were observed. 

For the LS state of Co^2+^ ions (complex **33**), the His ligands exhibited shortened distances to the Co^2+^ center compared to those with HS Co^2+^ ions, indicating stronger metal–ligand interactions. In vacuum conditions, the coordination around the LS Co^2+^ exhibited an O1-Co-O21 angle of 174.28°, an N3-Co-N24 angle of 172.04°, and an N4-Co-N23 angle of 163.65°. Applying the PCM model resulted in a coordination geometry with an O1-Co-O21 angle of 170.35°, an N3-Co-N24 angle of 170.56°, and an N4-Co-N23 angle of 170.75°. Meanwhile, the complex models with the SMD exhibited an O1-Co-O21 angle of 170.80°, an N3-Co-N24 angle of 170.85°, and an N4-Co-N23 angle of 171.10°. These results indicate that the solvation effect had a minimal impact on the coordination geometry around the Co^2+^ center, as the examined angles remained similar under both vacuum and aqueous conditions, likely due to the strengthened coordination interactions.

Similar results were observed for the coordination of Ni^2+^ ion complex **34**, where the examined angles remained comparable under vacuum and aqueous conditions. The coordination geometry exhibited an O1-Ni-O21 angle of 176.50°, an N3-Ni-N24 angle of 166.85°, and an N4-Ni-N23 angle of 166.85°. When employing the PCM model, these angles changed to 175.27°, 170.19°, and 169.93°, respectively. Furthermore, applying the SMD resulted in angles of 175.76°, 169.90°, and 169.96° for O1-Ni-O21, N3-Ni-N24, and N4-Ni-N23, respectively. These findings indicate that the coordination geometry around the Ni^2+^ center in complex **34** remained relatively consistent across vacuum and aqueous environments, with only minor variations observed in the bond angles.

For Cu^2+^ coordination, the Jahn–Teller effect must be considered when examining octahedral coordination geometries due to the unique electronic structure of copper. In the present study (complex **35**), we did not confirm the formation of an octahedral structure directly by two His ligands. Instead, one His residue acted as a stable bidentate ligand, while another histidine exhibited additional weak interactions, indicating the potential to form a tridentate ligand. The N3 atom in the amino group was relatively distant from the metal center at 2.96 Å compared to the coordinating carboxylate (O1) and imidazole N4 atoms, resulting in a weaker interaction. Consequently, the complex structure more closely resembled a square planar molecular geometry formed by two bidentate ligands. Such structures are common for Cu^2+^ complexes with His-containing residue and proteins [68] and possess potential pharmacological applications [69]. 

In a vacuum, the coordination exhibited an O1-Cu-O21 angle of 164.52°, an N3-Cu-N24 angle of 151.34°, and an N4-Cu-N23 angle of 164.48°. However, when considering aqueous environments using the PCM model, the angles changed to 167.96°, 169.71°, and 167.22°, respectively, exhibiting one His ligand acting as a bidentate ligand and the other as a tridentate ligand. The imidazole N3 atom was relatively distant from the Cu^2+^ center, at 3.23 Å. Furthermore, upon applying the SMD, the coordination displayed an O1-Cu-O21 angle of 150.51°, an N3-Cu-N24 angle of 125.91°, and an N4-Cu-N23 angle of 166.95°. An O23 atom from the carboxyl group in one His ligand and an N3 atom from the imidazole side chain in the other His ligand dissociated from the coordination sphere, with distances of 3.96 Å and 3.72 Å, respectively. These findings indicate that the coordination geometry consisted of two sets of bidentate ligands, and octahedral coordination was not formed.

The coordination of dual His ligands with Zn^2+^ (complex **36**) indicated that the solvation effect led to shortened distances between the nitrogen atoms of the imidazole side chain and amino group (N4, N3, N25, and N24) and the metal center, while the distances between the carboxyl oxygen atoms (O1 and O23) group and the metal center were elongated. Despite these changes, a distorted octahedral coordination geometry was formed under both conditions. In a vacuum, the coordination exhibited an O1-Zn-O21 angle of 174.11°, an N3-Zn-N24 angle of 163.05°, and an N4-Zn-N23 angle of 163.07°. When considering the PCM model, these angles changed to 175.68°, 167.59°, and 167.66°, respectively. The angles for the models with the SMD were 172.22°, 155.10°, and 162.03°, respectively. Despite slight differences in the distortion of the coordination configurations observed, the solvation effect had a minimal impact on the overall octahedral geometry.

## 3. Conclusions

In summary, we conducted DFT calculations and theoretically examined complexes formed by His ligands and divalent transition metal ions (Mn^2+^, Fe^2+^, Co^2+^, Ni^2+^, Cu^2+^, and Zn^2+^). The stable conformations were obtained using an efficient conformational exploration method. Based on the most stable conformations, we examined the structural features and the influence of coordination sites on relative stabilities for τ- and π-His structures. The model complexes were investigated with His acting as either a bidentate or tridentate ligand. The calculations were performed employing solvation models, including PCM and SMD, considering both HS and LS states for Mn^2+^, Fe^2+^, and Co^2+^. For [ML]^2+^ complexes with a single His ligand, LS Mn^2+^, HS and LS Fe^2+^, HS Co^2+^, and Ni^2+^ formed stable tridentate coordination, while others were influenced by an aqueous environment. When forming [ML_2_]^2+^ complexes with two His ligands, HS and LS Fe^2+^ and Ni^2+^ formed octahedral coordination or distorted octahedral coordination, whereas the solvation effect played a significant role for other metal ions. 

For deprotonated His ligand–metal coordination structures [M(L-H)]^+^, coordination with all metal ions can lead to a stable tridentate coordination geometry. However, when employing two deprotonated His ligands in metal coordination structures [M(L-H)_2_], only the coordination with Cu^2+^ ion results in a geometry other than a distorted octahedral coordination. Notably, the His ligand may still prefer to act as a bidentate mode rather than a tridentate ligand, especially for complexes with elongated bond distances between the functional groups and the metal centers in the distorted octahedral coordination. Thereby the final coordination geometry may depend on the rotation of carboxyl and amino groups. This work provides a fundamental investigation into transition metal His complexes, establishing a basic understanding of metal–ligand interactions essential for designing more complicated structures.

## Figures and Tables

**Table 1:** Relative energies of the His-metal complexes [ML]^2+^ in vacuum and aqueous environments and atomic distances in the coordination ([MC_6_H_9_N_3_O_2_]^2+^).

Complex	Metal Center	Charge and Spin Multiplicity	Δ*G* (kcal/mol, 298.15 K)	Coordination Mode	Atomic Distances of N3-M^2+^, N4-M^2+^, O1-M^2+^ (Å)
**1**	Mn^2+^	2, 6 (HS)	0	Tridentate	2.03, 2.14, 2.15
**1_PCM**			−221.81	Bidentate	2.11, 2.20, 2.98
**1_SMD**			−257.84		2.17, 2.25, 3.00
**2**		2, 2 (LS)	0	Tridentate	1.97, 2.04, 2.05
**2_PCM**			−214.49		2.04, 2.11, 2.23
**2_SMD**			−246.66		2.05, 2.12, 2.32
**3**	Fe^2+^	2, 5 (HS)	0	Tridentate	1.97, 2.08, 2.09
**3_PCM**			−216.58		2.05, 2.15, 2.37
**3_SMD**			−249.35		2.09, 2.17, 2.33
**4**		2, 1 (LS)	0	Tridentate	1.89, 1.95, 1.93
**4_PCM**			−209.09		1.93, 1.97, 1.98
**4_SMD**			−238.05		1.93, 1.98, 2.01
**5**	Co^2+^	2, 4 (HS)	0	Tridentate	1.93, 2.05, 2.05
**5_PCM**			−214.69		2.01, 2.09, 2.36
**5_SMD**			−246.78		2.04, 2.12, 2.33
**6**		2, 2 (LS)	0	Tridentate	1.88, 1.91, 2.06
**6_PCM**			−210.08		1.90, 1.94, 2.42
**6_SMD**			−241.21	Bidentate	1.90, 1.94, 2.96
**7**	Ni^2+^	2, 3	0	Tridentate	1.91, 2.00, 2.00
**7_PCM**			−210.77		1.97, 2.03, 2.16
**7_SMD**			−220.15		1.95, 2.01, 2.08
**8**	Cu^2+^	2, 2	0	Tridentate	1.92, 2.00, 2.11
**8_PCM**			−188.23		1.90, 1.97, 2.35
**8_SMD**			−243.20	Bidentate	1.94, 1.99, 3.03
**9**	Zn^2+^	2, 1	0	Tridentate	1.92, 2.01, 2.09
**9_PCM**			−220.68	Bidentate	1.98, 2.06, 3.37
**9_SMD**			−254.26		2.02, 2.07, 3.15

**Table 2:** Relative energies of the dual His-metal complexes [ML_2_]^2+^ in vacuum and aqueous environments and bond distances in the coordination ([M(C_6_H_9_N_3_O_2_)_2_]^2+^).

Complex	Metal Center	Charge and Spin Multiplicity	Δ*G* (kcal/mol, 298.15 K)	Coordination Mode	Distances of N4-M^2+^, O1-M^2+^, N3-M^2+^ (Å)	Distances of N25-M^2+^, O23-M^2+^, N24-M^2+^ (Å)
**10**	Mn^2+^	2, 6 (HS)	0	Distorted octahedral	2.28, 2.41, 2.17	2.28, 2.41, 2.17
**10_PCM**			−162.14	-	2.22, 3.50, 2.14	2.25, 3.52, 2.15
**10_SMD**			−191.85		2.25, 2.96, 2.21	2.27, 3.13, 2.21
**11**		2, 2 (LS)	0	Distorted octahedral	2.12, 2.13, 2.06	2.12, 2.13, 2.06
**11_PCM**			−155.97		2.10, 2.12, 2.05	2.10, 2.12, 2.04
**11_SMD**			−180.76	-	2.10, 3.02, 2.06	2.10, 3.01, 2.07
**12**	Fe^2+^	2, 5 (HS)	0	Distorted octahedral	2.24, 2.27, 2.11	2.23, 2.34, 2.13
**12_PCM**			−160.80	-	2.17, 3.46, 2.06	2.17, 3.39, 2.06
**12_SMD**			−188.95		2.18, 3.10, 2.10	2.19, 3.09, 2.11
**13**		2, 1 (LS)	0	Octahedral	2.06, 2.03, 2.02	2.06, 2.03, 2.02
**13_PCM**			−156.69	Distorted octahedral	2.04, 2.02, 2.00	2.04, 2.03, 2.00
**13_SMD**			−177.56	Octahedral	2.04, 2.02, 2.00	2.04, 2.02, 2.00
**14**	Co^2+^	2, 4 (HS)	0	Octahedral	2.19, 2.24, 2.10	2.19, 2.24, 2.10
**14_PCM**			−165.09	-	2.10, 3.46, 2.01	2.11, 3.48, 2.01
**14_SMD**			−190.57		2.10, 3.12, 2.04	2.12, 3.41, 2.03
**15**		2, 2 (LS)	0	Distorted octahedral	2.02, 2.40, 1.97	2.02, 2.41, 1.97
**15_PCM**			−156.80		2.01, 2.35, 1.96	2.01, 2.35, 1.96
**15_SMD**			−179.80	-	2.00, 2.76, 1.98	2.00, 2.94, 1.97
**16**	Ni^2+^	2, 3	0	Distorted octahedral	2.16, 2.23, 2.08	2.16, 2.24, 2.08
**16_PCM**			−155.25		2.14, 2.26, 2.07	2.14, 2.26, 2.07
**16_SMD**			−176.98		2.13, 2.27, 2.07	2.13, 2.27, 2.07
**17**	Cu^2+^	2, 2	0	-	2.09, 3.34, 1.97	2.07, 2.37, 2.03
**17_PCM**			−149.58		2.06, 2.84, 1.99	2.05, 2.50, 2.02
**17_SMD**			−175.83		2.07, 3.00, 2.01	2.04, 2.93, 2.03
**18**	Zn^2+^	2, 1	0	-	2.10, 3.56, 1.99	2.10, 3.56, 1.99
**18_PCM**			−158.65		2.10, 3.45, 2.02	2.11, 3.47, 2.02
**18_SMD**			−184.42		2.12, 3.43, 2.05	2.11, 3.26, 2.06

**Table 3:** Relative energies of the His-metal complexes [M(L-H)]^+^ in vacuum and aqueous environments and bond distances in the coordination ([MC_6_H_8_N_3_O_2_]^+^).

Complex	Metal Center	Charge and Spin Multiplicity	Δ*G* (kcal/mol, 298.15 K)	Coordination Mode	Distances of N3-M^2+^, N4-M^2+^, O1-M^2+^ (Å)
**19**	Mn^2+^	1, 6 (HS)	0		2.08, 2.17, 1.92
**19_PCM**			−93.39	Tridentate	2.16, 2.22, 2.08
**19_SMD**			−119.16		2.19, 2.26, 2.18
**20**		1, 2 (LS)	0		2.00, 2.05, 1.85
**20_PCM**			−84.73	Tridentate	2.04, 2.09, 1.99
**20_SMD**			−108.40		2.08, 2.11, 2.06
**21**	Fe^2+^	1, 5 (HS)	0		2.02, 2.12, 1.86
**21_PCM**			−85.64	Tridentate	2.08, 2.16, 1.98
**21_SMD**			−110.15		2.12, 2.19, 2.06
**22**		1, 1 (LS)	0	Tridentate	1.90, 1.95, 1.81
**22_PCM**			−81.45		1.92, 1.96, 1.89
**22_SMD**			−101.77		1.93, 1.97, 1.92
**23**	Co^2+^	1, 4 (HS)	0	Tridentate	1.99, 2.07, 1.85
**23_PCM**			−85.35		2.03, 2.11, 1.97
**23_SMD**			−109.29		2.08, 2.13, 2.06
**24**		1, 2 (LS)	0	Tridentate	1.96, 2.00, 1.79
**24_PCM**			−77.51		2.00, 2.02, 1.84
**24_SMD**			−99.29		1.93, 1.92, 2.09
**25**	Ni^2+^	1, 3	0	Tridentate	1.94, 2.01, 1.85
**25_PCM**			−83.07		1.98, 2.03, 1.95
**25_SMD**			−88.11		1.97, 2.01, 1.96
**26**	Cu^2+^	1, 2	0	Tridentate	1.97, 2.00, 1.87
**26_PCM**			−64.37		1.95, 1.98, 1.92
**26_SMD**			−98.13		2.06, 1.97, 2.09
**27**	Zn^2+^	1, 1	0	Tridentate	1.97, 2.06, 1.89
**27_PCM**			−89.92		2.02, 2.09, 2.03
**27_SMD**			−113.68		2.08, 2.11, 2.06

**Table 4:** Relative energies of the dual His-metal complexes [M(L-H)_2_] in vacuum and aqueous environments and bond distances in the coordination ([M(C_6_H_8_N_3_O_2_)_2_]).

Complex	Metal Center	Charge and Spin Multiplicity	Δ*G* (kcal/mol, 298.15 K)	Coordination Mode	Distances of N4-M^2+^, O1-M^2+^, N3-M^2+^ (Å)	Distances of N24-M^2+^, O21-M^2+^, N23-M^2+^ (Å)
**28**	Mn^2+^	0, 6 (HS)	0	Distorted octahedral	2.34, 2.12, 2.27	2.34, 2.12, 2.27
**28_PCM**			−34.35		2.31, 2.18, 2.25	2.30, 2.18, 2.25
**28_SMD**			−50.26		2.31, 2.31, 2.28	2.30, 2.29, 2.32
**29**		0, 2 (LS)	0	Distorted octahedral	2.10, 1.99, 2.03	2.10, 1.99, 2.03
**29_PCM**			−33.16		2.10, 2.02, 2.05	2.10, 2.02, 2.05
**29_SMD**			−50.29		2.12, 2.08, 2.07	2.12, 2.08, 2.07
**30**	Fe^2+^	0, 5 (HS)	0	Distorted octahedral	2.29, 2.03, 2.21	2.29, 2.04, 2.21
**30_PCM**			−32.28		2.25, 2.09, 2.20	2.25, 2.09, 2.20
**30_SMD**			−47.59		2.25, 2.16, 2.20	2.23, 2.19, 2.21
**31**		0, 1 (LS)	0	Distorted octahedral	2.04, 1.98, 1.99	2.04, 1.98, 1.99
**31_PCM**			−33.89		2.04, 2.01, 2.01	2.04, 2.00, 2.01
**31_SMD**			−47.87		2.04, 2.02, 2.01	2.04, 2.02, 2.01
**32**	Co^2+^	0, 4 (HS)	0	Distorted octahedral	2.22, 2.03, 2.16	2.22, 2.03, 2.16
**32_PCM**			−33.85		2.20, 2.09, 2.15	2.20, 2.09, 2.15
**32_SMD**			−48.63		2.19, 2.16, 2.15	2.20, 2.15, 2.14
**33**		0, 2 (LS)	0	Distorted octahedral	2.28, 1.95, 1.97	2.00, 1.93, 2.44
**33_PCM**			−33.96		2.01, 2.25, 1.98	2.01, 2.25, 1.98
**33_SMD**			−50.40		2.00, 2.29, 1.98	2.00, 2.29, 1.98
**34**	Ni^2+^	0, 3	0	Distorted octahedral	2.15, 2.04, 2.10	2.15, 2.04, 2.10
**34_PCM**			−35.41		2.14, 2.09, 2.10	2.14, 2.09, 2.10
**34_SMD**			−49.63		2.12, 2.11, 2.10	2.12, 2.12, 2.10
**35**	Cu^2+^	0, 2	0	-	2.05, 1.95, 2.96	2.41, 1.98, 2.03
**35_PCM**			−31.00		2.04, 1.98, 3.23	2.30, 2.01, 2.01
**35_SMD**			−54.38		2.04, 2.00, 3.72	2.05, 3.96, 1.99
**36**	Zn^2+^	0, 1	0	Distorted octahedral	2.24, 2.04, 2.20	2.24, 2.04, 2.20
**36_PCM**			−34.00		2.21, 2.13, 2.16	2.21, 2.13, 2.16
**36_SMD**			−50.38		2.16, 2.39, 2.15	2.19, 2.24, 2.14

## Data Availability

The original contributions presented in the study are included in the article/Supplementary Material, further inquiries can be directed to the corresponding author.

## Supplementary Materials

The following supporting information can be downloaded at: https://www.mdpi.com/article/10.3390/molecules29133003/s1, Figure S1: Optimized coordination structures for the model complexes of transition metals with His ligand and water molecules; Table S1: Selected bond distances (Å) for the coordinated groups and water molecules in the transition metal-His-water complexes; Figure S2: RDG analysis of model complexes with transition metals coordinated to His ligands and water molecules; Figure S3: RDG analysis of model complexes **1**–**9**; Figure S4: RDG analysis of model complexes **10**–**18**; Figure S5: RDG analysis of model complexes **19**–**27**; Figure S6: RDG analysis of model complexes **28**–**36**; Figure S7: ESP maps for transition metal-His complexes **28**–**36**; Figure S8: Charge distributions (NPA) for transition metal-His complexes for transition metal-His complexes **28**–**36**; Table S2: NPA charges for the coordinated groups and metal centers in the transition metal-His complexes **28**–**36**; Figure S9: LEAE analysis for transition metal-His complexes **28**–**36**, LEAE Analysis results, and calculated coordinates for conformational search and model complexes.
