# Supplementary material for: Theoretical Analysis of Coordination Geometries in Transition Metal–Histidine Complexes Using Quantum Chemical Calculations
Source: Molecules. 2024 Jun 25;29(13):3003. doi: 10.3390/molecules29133003 (PMC11243457; doi:10.3390/molecules29133003)
Supplement: Supplementary file 1 [file molecules-29-03003-s001.zip › molecules-3033896-supplementary.pdf]

# Theoretical Analysis of Coordination Geometries in Transition Metal-Histidine Complexes Using Quantum Chemical Calculations

*Dapeng Zhang,*[†] *Naoki Kishimoto*[*†]

[†] Department of Chemistry, Graduate School of Science, Tohoku University, 6-3, Aoba, Aramaki, Aoba-ku, Sendai 980-8578, Japan

**Corresponding Author**

***E-mail**: kishimoto@tohoku.ac.jp

# Supporting Information

# Table of Contents


**Figure S1.** Structures for the model complexes of transition metals with His ligand and water molecules ……………………………………………….…..………..**S3**

**Table S1.** Distances in the transition metal-His-water complexes ……..................**S4**

**Figure S2.** RDG analysis for the model complexes of transition metals with His ligand and water molecules ..…………… ………………………….……..**S5**

**Figure S3.** RDG analysis for the model complexes 1-9……………………………..**S6**

**Figure S4.** RDG analysis for the model complexes 10-18…………………………..**S7**

**Figure S5.** RDG analysis for the model complexes 19-27…………………………..**S8**

**Figure S6.** RDG analysis for the model complexes 28-36…………………………..**S9**

**Figure S7.** ESP maps for transition metal-His complexes ……………….....…….**S10**

**Figure S8.** Charge distributions (NPA) for transition metal-His complexes ……...**S11**

**Table S2.** NPA charges for the coordinated groups and metal centers in the transition metal-His complexes …………………………………...………...**S12**

**Figure S9.** LEAE results for the model complexes 28-36…………..…………......**S13**

Details of LEAE analysis …………………...…………………………….........**S14**

**Coordinate** ……………………………………………………………...………….**S18**

**Figure S1.** Optimized coordination structures for the model complexes of transition metals with His ligand and water molecules.

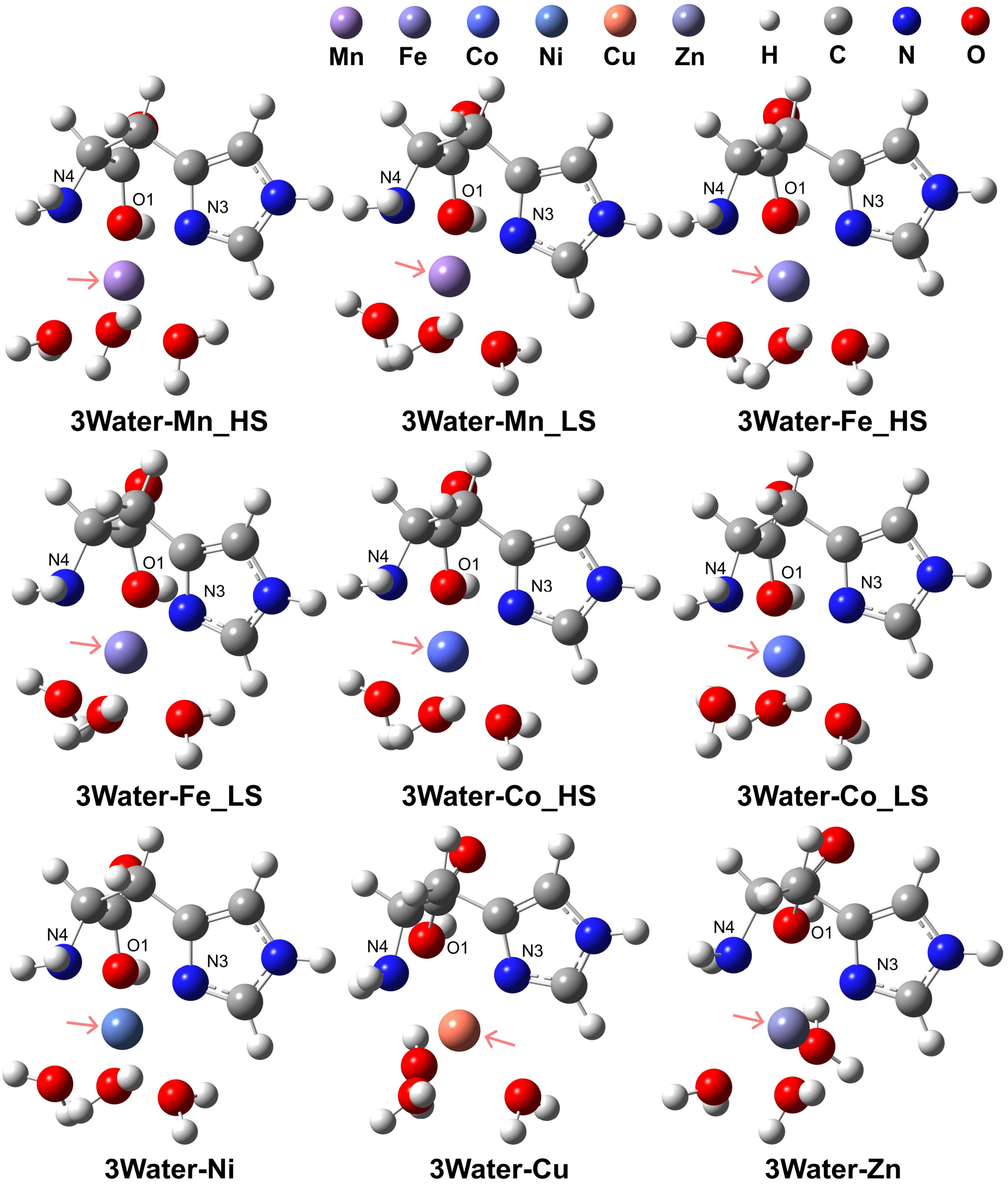

**Table S1.** Selected bond distances (Å) for the coordinated groups and water molecules in the transition metal-His-water complexes.

| Complex | Metal center | Charge and spin multiplicity | Coordination mode | Distances of $H_2O$-$M^{2+}$ | Distances of N4-$M^{2+}$, O1-$M^{2+}$, N3-$M^{2+}$ |
|---|---|---|---|---|---|
| 3Water-Mn_HS | $Mn^{2+}$ | 0, 6 (HS) | Octahedral coordination | 2.26, 2.21, 2.23 | 2.24, 2.39, 2.16 |
| 3Water-Mn_LS | | 0, 2 (LS) | | 2.09, 2.13, 2.12 | 2.08, 2.14, 2.03 |
| 3Water-Fe_HS | $Fe^{2+}$ | 0, 5 (HS) | | 2.18, 2.23, 2.20 | 2.19, 2.27, 2.08 |
| 3Water-Fe_HS | | 0, 1 (LS) | | 2.08, 2.03, 2.08 | 2.02, 2.04, 1.99 |
| 3Water-Co_HS | $Co^{2+}$ | 0, 4 (HS) | | 2.13, 2.17, 2.20 | 2.14, 2.24, 2.06 |
| 3Water-Co_HS | | 0, 2 (LS) | | 2.04, 2.27, 2.03 | 1.97, 2.42, 1.95 |
| 3Water-Ni | $Ni^{2+}$ | 0, 3 | | 2.14, 2.10, 2.16 | 2.09, 2.18, 2.03 |
| 3Water-Cu | $Cu^{2+}$ | 0, 2 | - | 2.08, 2.32, 2.07 | 2.02, 2.23, 1.96 |
| 3Water-Zn | $Zn^{2+}$ | 0, 1 | - | 2.26, 2.07, 2.09 | 2.09, 3.46, 2.03 |

**Figure S2.** RDG analysis of model complexes with transition metals coordinated to His ligands and water molecules. Dashed black lines indicate regions of weak non-covalent interactions, solid blue arrows highlight areas of highest interaction density, and dashed blue arrows show regions of intermediate interaction density.

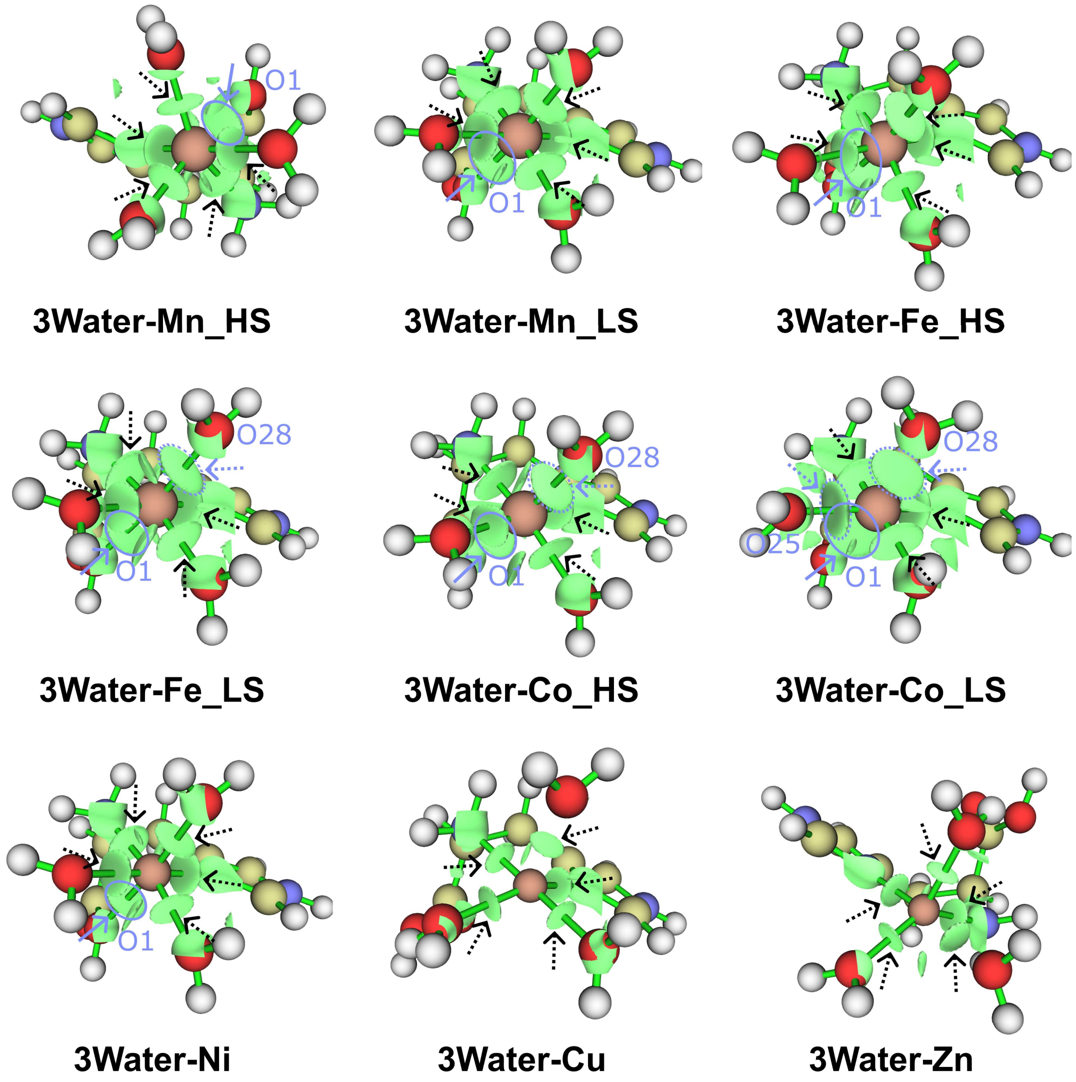

**Figure S3.** RDG analysis of model complexes 1-9. Dashed black lines indicate regions of weak non-covalent interactions, solid blue arrows highlight areas of highest interaction density.

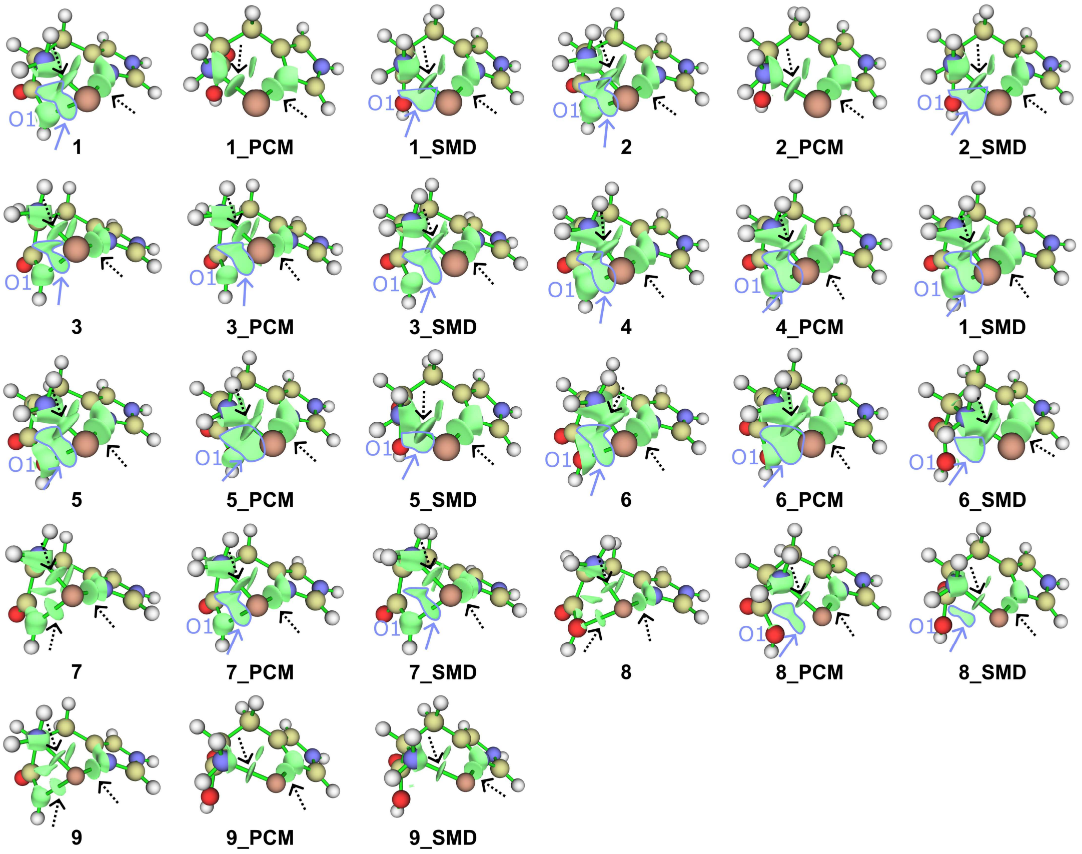

**Figure S4.** RDG analysis of model complexes 10-18. Dashed black lines indicate regions of weak non-covalent interactions, solid blue arrows highlight areas of highest interaction density.

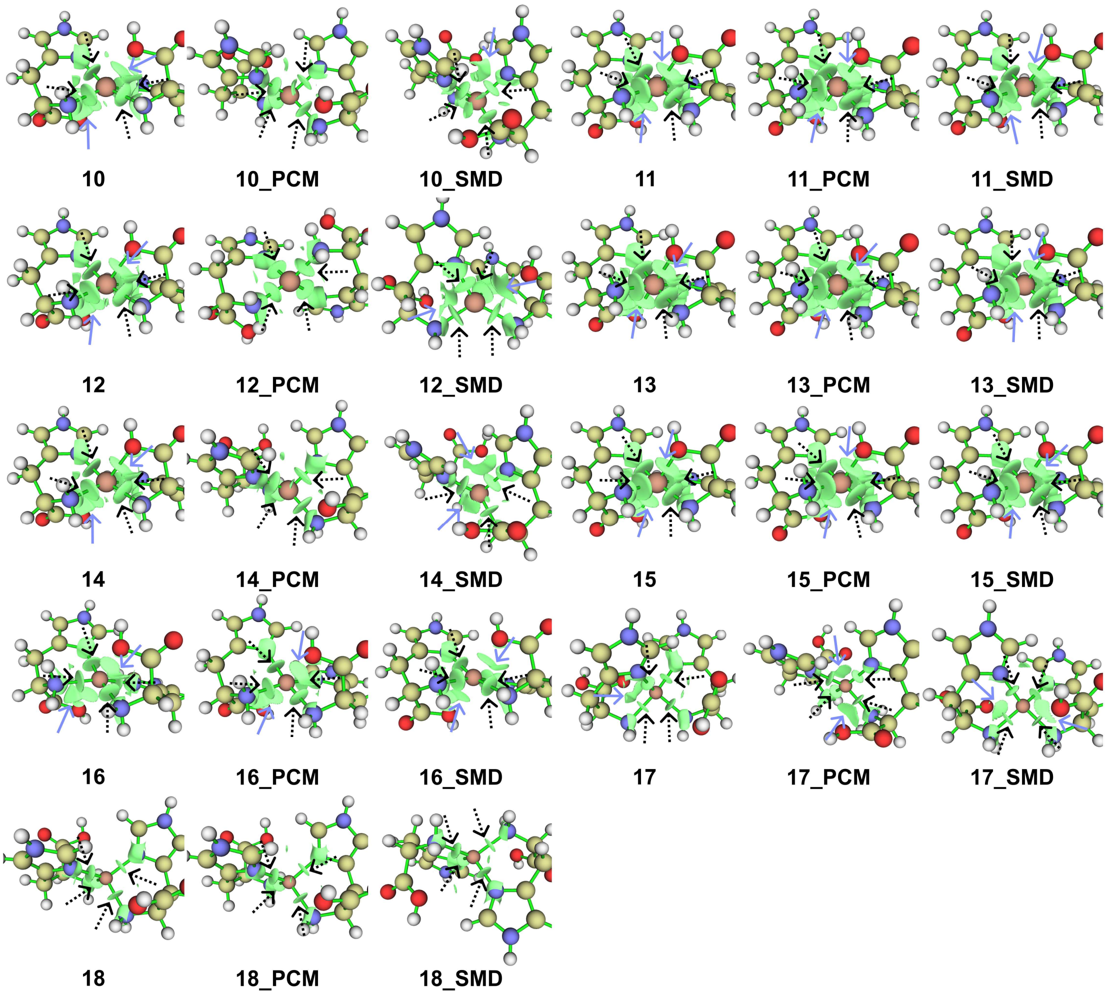

**Figure S5.** RDG analysis of model complexes 19-27. Dashed black lines indicate regions of weak non-covalent interactions, solid blue arrows highlight areas of highest interaction density.

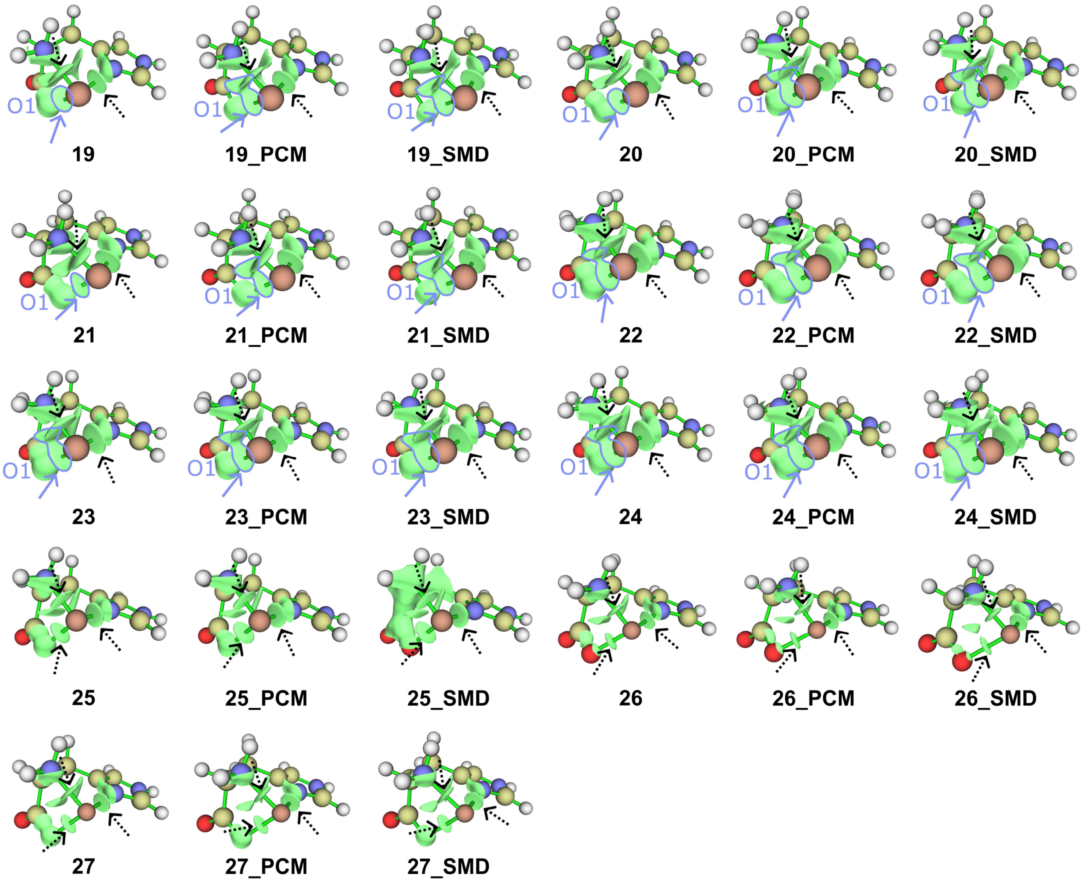

**Figure S6.** RDG analysis of model complexes 28-36. Dashed black lines indicate regions of weak non-covalent interactions, solid blue arrows highlight areas of highest interaction density.

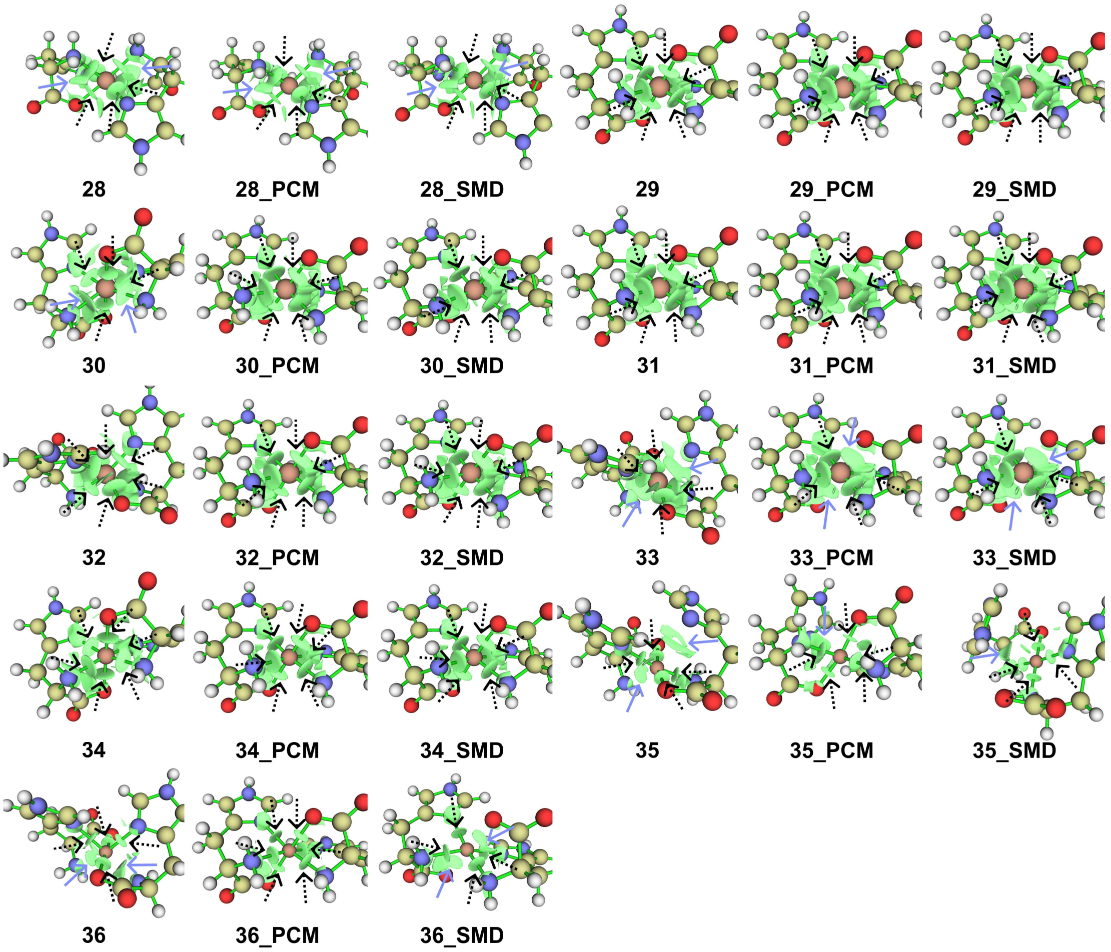

**Figure S7.** ESP maps for transition metal-His complexes 28-36.

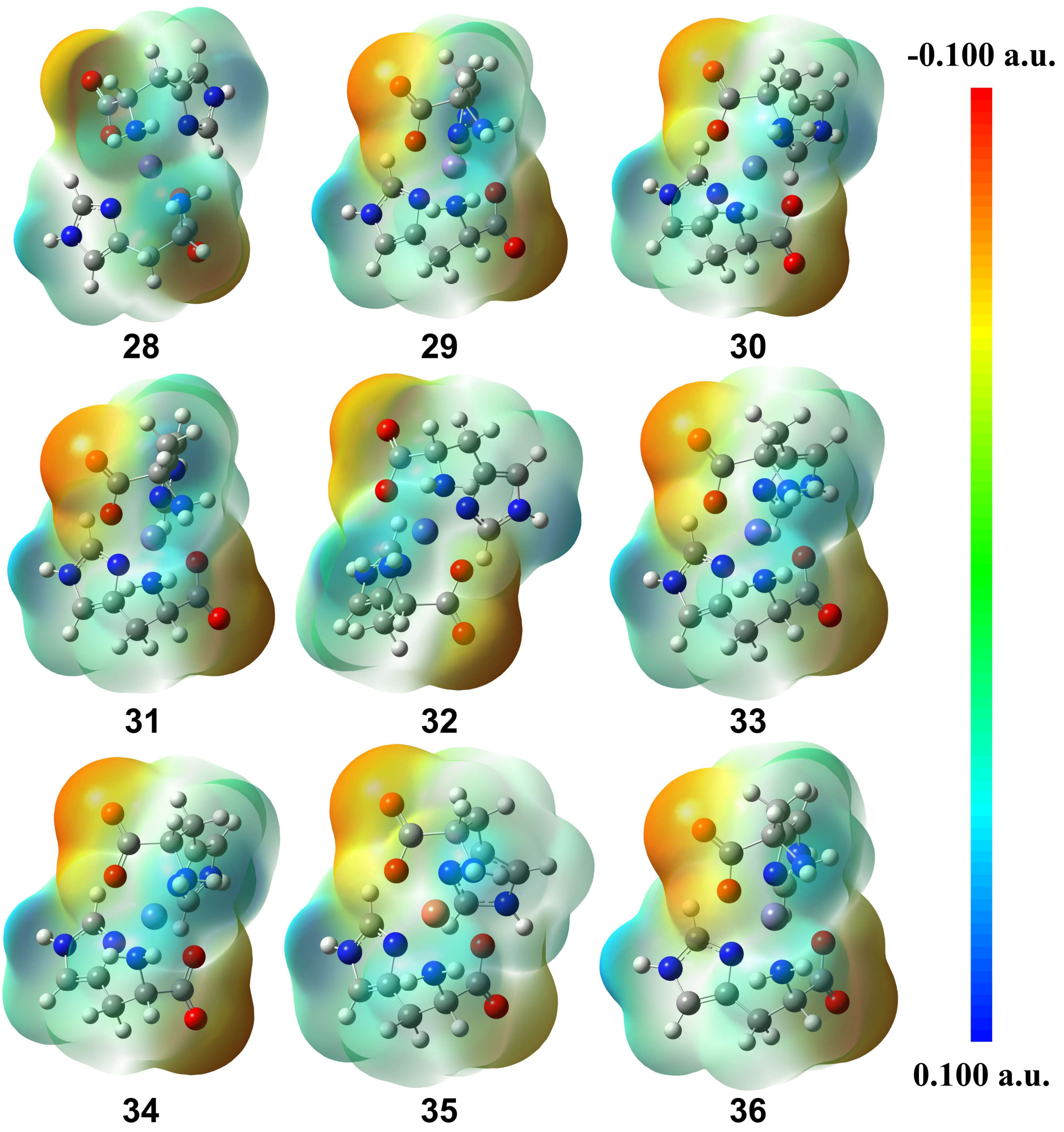

**Figure S8.** Charge distributions (NPA) for transition metal-His complexes for transition metal-His complexes 28-36.

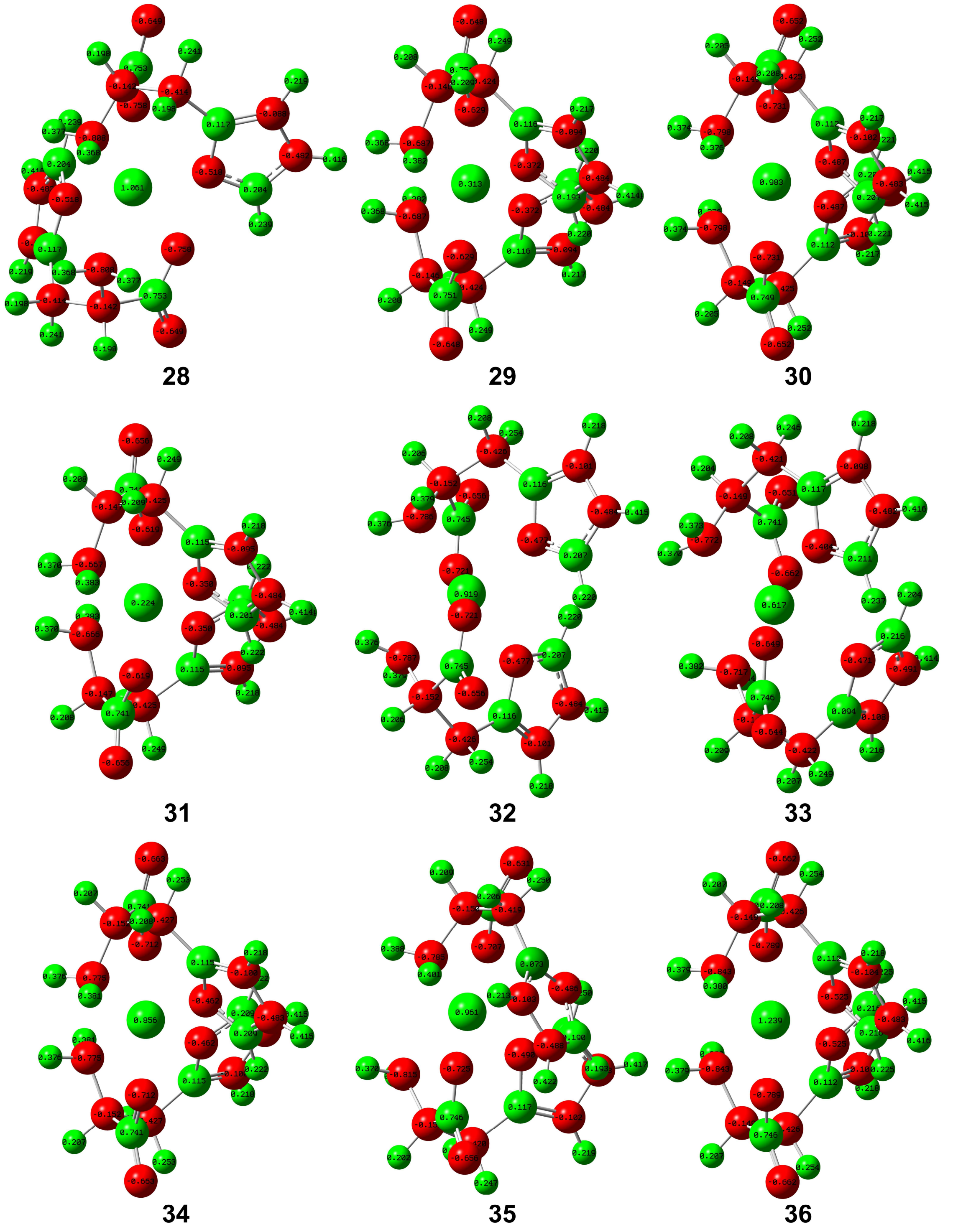

**Table S2.** NPA charges for the coordinated groups and metal centers in the transition metal-His complexes 28-36.

| Complex | Metal center | Charge and spin multiplicity | N4 O1 N3 | N24 O21 N23 | $M^{2+}$ |
|---|---|---|---|---|---|
| 28 | $Mn^{2+}$ | 0, 6 (HS) | -0.808, -0.758, -0.518 | -0.808, -0.758, -0.518 | 1.061 |
| 29 | | 0, 2 (LS) | -0.687, -0.629, -0.372 | -0.687, -0.629, -0.372 | 0.313 |
| 30 | $Fe^{2+}$ | 0, 5 (HS) | -0.798, -0.731, -0.487 | -0.798, -0.731, -0.487 | 0.983 |
| 31 | | 0, 1 (LS) | -0.667, -0.619, -0.350 | -0.666, -0.619, -0.350 | 0.224 |
| 32 | $Co^{2+}$ | 0, 4 (HS) | -0.786, -0.721, -0.477 | -0.787, -0.721, -0.477 | 0.919 |
| 33 | | 0, 2 (LS) | -0.772, -0.662, -0.404 | -0.717, -0.649, -0.471 | 0.617 |
| 34 | $Ni^{2+}$ | 0, 3 | -0.775, -0.712, -0.462 | -0.775, -0.712, -0.462 | 0.856 |
| 35 | $Cu^{2+}$ | 0, 2 | -0.785, -0.707, -0.486 | -0.815, -0.725, -0.490 | 0.961 |
| 36 | $Zn^{2+}$ | 0, 1 | -0.843, -0.789, -0.525 | -0.843, -0.789, -0.525 | 1.239 |

**Figure S9.** LEAE analysis for transition metal-His complexes 28-36. The red circles highlight the regions exhibiting the highest electrophilicity.

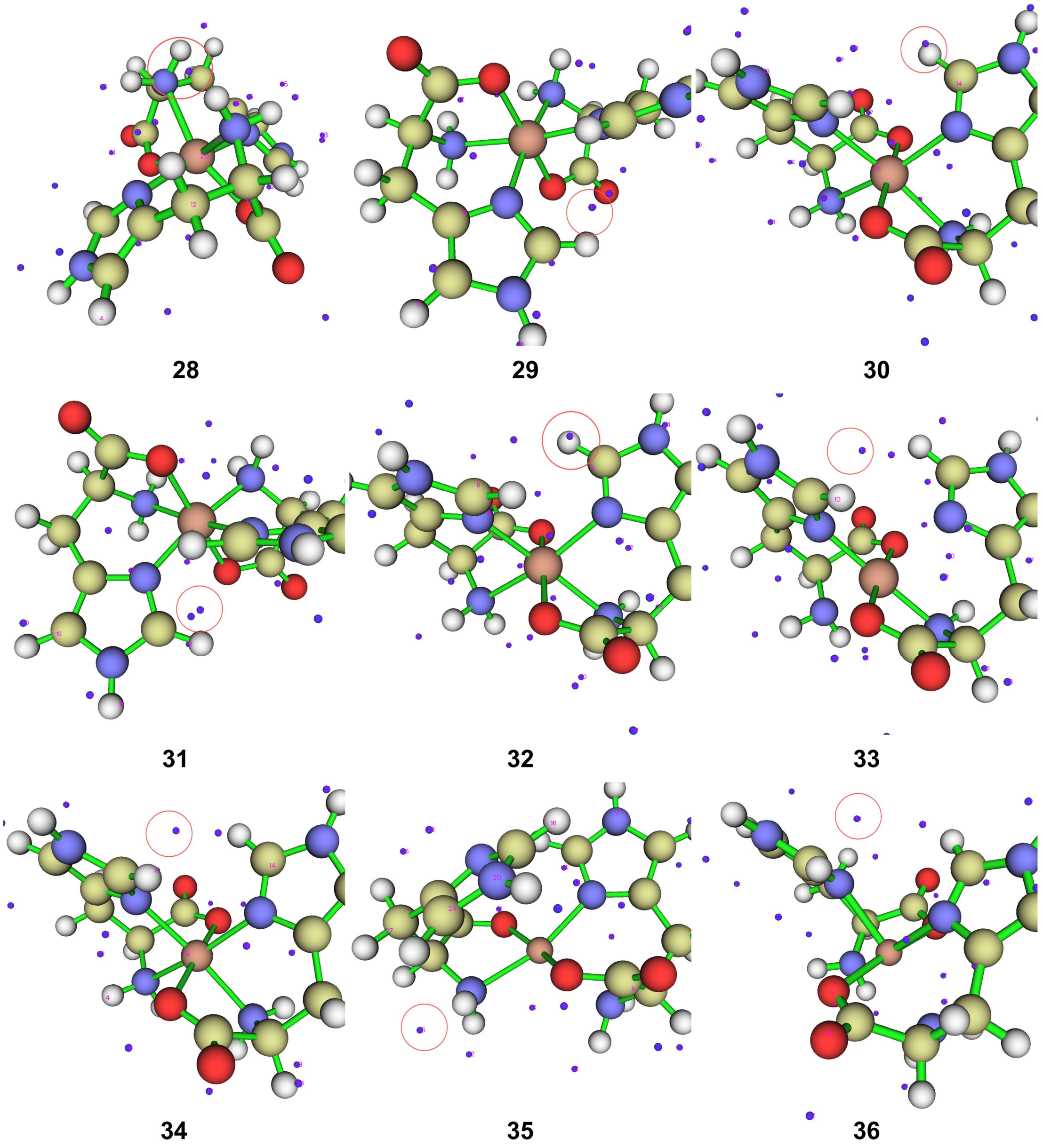

# LEAE Analysis Results: * Denotes Highest Electrophilicity

Complex 28

| | # | a.u. | eV | kcal/mol | X/Y/Z coordinate(Angstrom) | | |
|---|---|---|---|---|---|---|---|
| | 1 | -0.00134944 | -0.036720 | -0.846785 | -4.867370 | 2.091620 | 0.463504 |
| | 2 | -0.00108986 | -0.029657 | -0.683898 | -4.259228 | -0.063116 | -1.482214 |
| | 3 | -0.00086943 | -0.023658 | -0.545575 | -4.015957 | 3.572580 | -0.916923 |
| | 4 | -0.00102343 | -0.027849 | -0.642212 | -3.511100 | 1.882855 | -2.817720 |
| | 5 | -0.00000590 | -0.000161 | -0.003704 | -3.359060 | -3.706530 | -1.924896 |
| | 6 | -0.00034203 | -0.009307 | -0.214627 | -2.033601 | -0.131130 | -1.043411 |
| | 7 | -0.00445539 | -0.121237 | -2.795804 | -1.832299 | 3.460532 | 0.019507 |
| * | 8 | -0.00678801 | -0.184711 | -4.259545 | -1.687755 | -0.113450 | 3.433407 |
| | 9 | -0.00298521 | -0.081232 | -1.873249 | -1.342802 | 1.268845 | 1.563282 |
| | 10 | -0.00405268 | -0.110279 | -2.543098 | -1.269784 | 1.467241 | -2.064824 |
| | 11 | -0.00300469 | -0.081762 | -1.885474 | -0.724931 | 0.970402 | 1.562178 |
| | 12 | -0.00103155 | -0.028070 | -0.647306 | -0.081209 | 0.111909 | -1.397575 |
| | 13 | -0.00386485 | -0.105168 | -2.425233 | -0.106694 | 2.524275 | 2.159711 |
| | 14 | -0.00385782 | -0.104977 | -2.420820 | 0.094776 | -2.534879 | 2.175006 |
| | 15 | -0.00299499 | -0.081498 | -1.879388 | 0.761366 | -0.952343 | 1.558941 |
| | 16 | -0.00297963 | -0.081080 | -1.869750 | 1.285820 | -1.271854 | 1.551182 |
| | 17 | -0.00403905 | -0.109908 | -2.534544 | 1.304746 | -1.395547 | -2.033461 |
| | 18 | -0.00678205 | -0.184549 | -4.255806 | 1.587248 | 0.092799 | 3.437402 |
| | 19 | -0.00442573 | -0.120430 | -2.777188 | 1.879693 | -3.446656 | 0.042911 |
| | 20 | -0.00033855 | -0.009212 | -0.212443 | 2.110325 | 0.194146 | -1.012427 |
| | 21 | -0.00000591 | -0.000161 | -0.003710 | 3.375804 | 3.670901 | -1.951723 |
| | 22 | -0.00102370 | -0.027856 | -0.642379 | 3.473592 | -1.935900 | -2.845327 |
| | 23 | -0.00086829 | -0.023627 | -0.544860 | 4.016132 | -3.568656 | -0.909607 |
| | 24 | -0.00108346 | -0.029483 | -0.679885 | 4.205132 | 0.036086 | -1.544620 |
| | 25 | -0.00134321 | -0.036551 | -0.842876 | 4.862815 | -2.090580 | 0.466253 |

Complex 29

| | # | a.u. | eV | kcal/mol | X/Y/Z coordinate(Angstrom) | | |
|---|---|---|---|---|---|---|---|
| | 1 | -0.00506480 | -0.137820 | -3.178213 | -3.893787 | 2.646954 | 0.315709 |
| | 2 | -0.00437576 | -0.119070 | -2.745831 | -3.736166 | 0.899538 | 2.735750 |
| | 3 | -0.00132952 | -0.036178 | -0.834285 | -2.272480 | 3.935784 | 1.214178 |
| | 4 | -0.00208466 | -0.056726 | -1.308142 | -2.037720 | 0.899595 | -0.793777 |
| | 5 | -0.00261901 | -0.071267 | -1.643455 | -2.038832 | 2.596321 | 3.442015 |
| | 6 | -0.00242653 | -0.066029 | -1.522675 | -1.916855 | -0.542751 | 1.637520 |
| | 7 | -0.00317174 | -0.086307 | -1.990300 | -0.569372 | -3.526886 | -0.044644 |
| | 8 | -0.00548265 | -0.149191 | -3.440421 | -0.294003 | 1.272473 | 2.645468 |
| | 9 | -0.01541025 | -0.419334 | -9.670088 | -0.243597 | 2.698740 | 0.116380 |
| * | 10 | -0.01542591 | -0.419760 | -9.679914 | 0.179745 | 2.679251 | -0.111614 |
| | 11 | -0.00545948 | -0.148560 | -3.425881 | 0.339202 | 1.270666 | -2.648111 |
| | 12 | -0.00317662 | -0.086440 | -1.993362 | 0.601684 | -3.545762 | 0.051874 |
| | 13 | -0.00242546 | -0.066000 | -1.522000 | 1.925702 | -0.529569 | -1.643378 |
| | 14 | -0.00261386 | -0.071127 | -1.640226 | 1.990626 | 2.602459 | -3.426761 |
| | 15 | -0.00208979 | -0.056866 | -1.311367 | 2.093400 | 0.800802 | 0.802660 |
| | 16 | -0.00133121 | -0.036224 | -0.835349 | 2.274470 | 3.922411 | -1.160556 |
| | 17 | -0.00436736 | -0.118842 | -2.740561 | 3.778167 | 0.899050 | -2.702140 |
| | 18 | -0.00506032 | -0.137698 | -3.175399 | 3.831884 | 2.646639 | -0.267518 |

Complex 30

| | # | a.u. | eV | kcal/mol | X/Y/Z coordinate(Angstrom) | | |
|---|---|---|---|---|---|---|---|
| | 1 | -0.00038396 | -0.010448 | -0.240936 | -5.067092 | -1.285522 | -1.094095 |
| | 2 | -0.00017925 | -0.004878 | -0.112482 | -4.964076 | 1.263204 | 0.726290 |
| | 3 | -0.00015392 | -0.004188 | -0.096587 | -4.497629 | 1.267498 | -2.338601 |
| | 4 | -0.00201560 | -0.054847 | -1.264810 | -4.077565 | -0.581715 | 2.585611 |
| | 5 | -0.00184938 | -0.050324 | -1.160504 | -3.949710 | -2.640163 | 0.428454 |
| | 6 | -0.00009905 | -0.002695 | -0.062158 | -3.248265 | 2.738671 | 1.020113 |
| | 7 | -0.00012993 | -0.003536 | -0.081534 | -3.144497 | -1.280631 | -2.569410 |
| | 8 | -0.00011876 | -0.003231 | -0.074520 | -3.078001 | 3.956135 | -1.343374 |
| | 9 | -0.00091372 | -0.024864 | -0.573368 | -2.686513 | -1.689838 | 3.708815 |
| | 10 | -0.00141991 | -0.038638 | -0.891005 | -2.371339 | -3.693423 | 2.006221 |
| | 11 | -0.00041081 | -0.011179 | -0.257790 | -1.849429 | 2.220008 | -2.225384 |
| | 12 | -0.00127961 | -0.034820 | -0.802971 | -1.725047 | 0.871027 | 0.956001 |
| | 13 | -0.00385556 | -0.104915 | -2.419405 | -1.034176 | -1.492032 | -0.876082 |
| | 14 | -0.00362310 | -0.098589 | -2.273529 | -0.593091 | -2.772985 | 0.997437 |
| * | 15 | -0.00735884 | -0.200244 | -4.617745 | -0.665873 | -0.521717 | 2.866497 |
| | 16 | -0.00151220 | -0.041149 | -0.948920 | -0.601810 | 0.935481 | 0.926509 |
| | 17 | -0.00219882 | -0.059833 | -1.379781 | -0.297407 | -1.188200 | -2.047329 |
| | 18 | -0.00219426 | -0.059709 | -1.376921 | 0.312613 | 1.208293 | -2.025432 |
| | 19 | -0.00151160 | -0.041133 | -0.948547 | 0.565840 | -0.969432 | 0.913330 |
| | 20 | -0.00724254 | -0.197080 | -4.544766 | 0.642772 | 0.522356 | 2.842998 |
| | 21 | -0.00353301 | -0.096138 | -2.216997 | 0.580320 | 2.743999 | 0.938749 |
| | 22 | -0.00385822 | -0.104987 | -2.421070 | 1.020061 | 1.501609 | -0.843638 |
| | 23 | -0.00041359 | -0.011254 | -0.259535 | 1.763460 | -2.213634 | -2.209355 |
| | 24 | -0.00125823 | -0.034238 | -0.789549 | 1.729975 | -0.873634 | 0.931996 |
| | 25 | -0.00138455 | -0.037676 | -0.868819 | 2.385877 | 3.711529 | 2.000144 |
| | 26 | -0.00088796 | -0.024163 | -0.557205 | 2.637872 | 1.792381 | 3.737486 |
| | 27 | -0.00011882 | -0.003233 | -0.074564 | 3.108606 | -3.973664 | -1.303780 |
| | 28 | -0.00012877 | -0.003504 | -0.080805 | 3.167712 | 1.239592 | -2.554609 |
| | 29 | -0.00009980 | -0.002716 | -0.062628 | 3.306892 | -2.663378 | 0.999469 |
| | 30 | -0.00178274 | -0.048511 | -1.118689 | 3.964019 | 2.635855 | 0.414862 |

| | | | | | | |
|---|---|---|---|---|---|---|
| 31 | -0.00194532 | -0.052935 | -1.220707 | 4.062744 | 0.637333 | 2.625126 |
| 32 | -0.00015246 | -0.004149 | -0.095668 | 4.495106 | -1.353106 | -2.313361 |
| 33 | -0.00017373 | -0.004727 | -0.109018 | 4.978209 | -1.171011 | 0.786051 |
| 34 | -0.00037243 | -0.010134 | -0.233701 | 5.062349 | 1.301269 | -1.051939 |

Complex 31

| # | a.u. | eV | kcal/mol | X/Y/Z coordinate(Angstrom) | | |
|---|---|---|---|---|---|---|
| 1 | -0.00561216 | -0.152715 | -3.521686 | -3.856146 | 2.627388 | 0.313086 |
| 2 | -0.00497087 | -0.135264 | -3.119270 | -3.683731 | 0.934021 | 2.757500 |
| 3 | -0.00147204 | -0.040056 | -0.923719 | -2.276431 | 3.897414 | 1.151952 |
| 4 | -0.00226085 | -0.061521 | -1.418707 | -1.995922 | 0.931368 | -0.793964 |
| 5 | -0.00289185 | -0.078691 | -1.814668 | -1.995021 | 2.625378 | 3.441407 |
| 6 | -0.00262904 | -0.071540 | -1.649751 | -1.884191 | -0.539798 | 1.639418 |
| 7 | -0.00436984 | -0.118909 | -2.742117 | -0.496720 | -3.518451 | -0.044166 |
| 8 | -0.00019487 | -0.005303 | -0.122286 | -0.289144 | -1.495778 | 2.294478 |
| 9 | -0.00603206 | -0.164141 | -3.785181 | -0.189646 | 1.253086 | 2.605416 |
| * 10 | -0.01875338 | -0.510305 | -11.767932 | -0.072187 | 2.645369 | 0.070397 |
| 11 | -0.01873770 | -0.509879 | -11.758097 | 0.126345 | 2.689945 | -0.086956 |
| 12 | -0.00603593 | -0.164246 | -3.787604 | 0.211677 | 1.310837 | -2.630254 |
| 13 | -0.00019560 | -0.005322 | -0.122740 | 0.293742 | -1.500194 | -2.266221 |
| 14 | -0.00437712 | -0.119107 | -2.746686 | 0.461251 | -3.504036 | -0.031302 |
| 15 | -0.00263329 | -0.071656 | -1.652419 | 1.799352 | -0.482923 | -1.672292 |
| 16 | -0.00289999 | -0.078913 | -1.819776 | 1.929071 | 2.630270 | -3.425641 |
| 17 | -0.00227685 | -0.061956 | -1.428747 | 2.047150 | 0.834073 | 0.803749 |
| 18 | -0.00147813 | -0.040222 | -0.927543 | 2.273907 | 3.937226 | -1.190992 |
| 19 | -0.00495991 | -0.134966 | -3.112391 | 3.721188 | 0.903497 | -2.704573 |
| 20 | -0.00562334 | -0.153019 | -3.528701 | 3.807195 | 2.679032 | -0.309540 |

Complex 32

| # | a.u. | eV | kcal/mol | X/Y/Z coordinate(Angstrom) | | |
|---|---|---|---|---|---|---|
| 1 | -0.00507318 | -0.138048 | -3.183468 | -4.019392 | -0.573771 | 2.682446 |
| 2 | -0.00481177 | -0.130935 | -3.019434 | -3.846491 | -2.744773 | 0.646647 |
| 3 | -0.00019658 | -0.005349 | -0.123355 | -3.185172 | 2.682317 | 0.938154 |
| 4 | -0.00030780 | -0.008376 | -0.193149 | -3.257389 | 3.758433 | -1.534304 |
| 5 | -0.00029240 | -0.007957 | -0.183482 | -2.853836 | -1.731940 | -2.402889 |
| 6 | -0.00176267 | -0.047965 | -1.106091 | -2.452944 | -1.542354 | 3.818920 |
| 7 | -0.00281254 | -0.076533 | -1.764896 | -2.220955 | -3.634181 | 2.251280 |
| 8 | -0.00263822 | -0.071790 | -1.655509 | -2.117355 | 0.807360 | 0.979434 |
| 9 | -0.00203375 | -0.055341 | -1.276197 | -1.838794 | -1.539556 | -0.554854 |
| 10 | -0.00092041 | -0.025046 | -0.577566 | -1.567842 | 2.197693 | -2.270041 |
| 11 | -0.00051662 | -0.014058 | -0.324185 | -1.172313 | 0.268211 | -3.787455 |
| 12 | -0.01377889 | -0.374943 | -8.646393 | -0.566409 | -0.385017 | 2.854529 |
| 13 | -0.00525072 | -0.142879 | -3.294881 | -0.529586 | 1.029330 | 0.878288 |
| 14 | -0.00699093 | -0.190233 | -4.386879 | -0.433451 | -2.646346 | 1.023310 |
| 15 | -0.00232847 | -0.063361 | -1.461140 | -0.028414 | 0.900876 | -2.241663 |
| 16 | -0.00234050 | -0.063688 | -1.468690 | -0.002891 | -0.995027 | -2.201098 |
| 17 | -0.00522391 | -0.142150 | -3.278054 | 0.519670 | -1.004162 | 0.901854 |
| * 18 | -0.01378429 | -0.375090 | -8.649780 | 0.519068 | 0.371699 | 2.813976 |
| 19 | -0.00697091 | -0.189688 | -4.374317 | 0.442769 | 2.696400 | 1.087323 |
| 20 | -0.00051705 | -0.014070 | -0.324453 | 1.216534 | -0.306695 | -3.790467 |
| 21 | -0.00091854 | -0.024995 | -0.576391 | 1.600378 | -2.227324 | -2.261908 |
| 22 | -0.00202963 | -0.055229 | -1.273615 | 1.869950 | 1.535889 | -0.560873 |
| 23 | -0.00264095 | -0.071864 | -1.657224 | 2.094152 | -0.811477 | 0.966857 |
| 24 | -0.00281329 | -0.076554 | -1.765371 | 2.203285 | 3.637606 | 2.237596 |
| 25 | -0.00175376 | -0.047722 | -1.100499 | 2.420046 | 1.550271 | 3.822416 |
| 26 | -0.00029180 | -0.007940 | -0.183107 | 2.827068 | 1.744069 | -2.431243 |
| 27 | -0.00019661 | -0.005350 | -0.123375 | 3.154673 | -2.702031 | 0.953886 |
| 28 | -0.00030769 | -0.008373 | -0.193080 | 3.259563 | -3.775429 | -1.513837 |
| 29 | -0.00479719 | -0.130538 | -3.010286 | 3.818622 | 2.700079 | 0.576461 |
| 30 | -0.00505581 | -0.137576 | -3.172573 | 4.037356 | 0.599274 | 2.688161 |

Complex 33

| # | a.u. | eV | kcal/mol | X/Y/Z coordinate(Angstrom) | | |
|---|---|---|---|---|---|---|
| 1 | -0.00113656 | -0.030927 | -0.713203 | -4.909370 | -1.437639 | -1.171062 |
| 2 | -0.00063594 | -0.017305 | -0.399060 | -4.786120 | 1.377345 | 0.379709 |
| 3 | -0.00599434 | -0.163114 | -3.761508 | -4.064553 | -2.388873 | 1.156982 |
| 4 | -0.00608507 | -0.165583 | -3.818443 | -3.833601 | 0.204353 | 2.593451 |
| 5 | -0.00068157 | -0.018546 | -0.427689 | -2.639941 | -2.144589 | -2.098182 |
| 6 | -0.00315612 | -0.085882 | -1.980494 | -2.442304 | -2.774929 | 4.025115 |
| 7 | -0.00303227 | -0.082512 | -1.902783 | -2.443859 | -3.125226 | 2.975600 |
| 8 | -0.00206976 | -0.056321 | -1.298796 | -2.231169 | -0.777145 | 3.928176 |
| 9 | -0.00322301 | -0.087702 | -2.022468 | -2.171700 | -1.556699 | -0.549770 |
| 10 | -0.00269578 | -0.073356 | -1.691629 | -2.025165 | 0.895706 | 0.639177 |
| 11 | -0.00728564 | -0.198252 | -4.571813 | -0.690387 | -2.749794 | 1.370781 |
| 12 | -0.00163240 | -0.044420 | -1.024344 | -0.670836 | 0.043322 | -3.687790 |
| * 13 | -0.01001211 | -0.272443 | -6.282700 | -0.503982 | -0.114466 | 2.697974 |
| 14 | -0.00080066 | -0.021787 | -0.502424 | -0.454202 | 3.105128 | -0.740795 |
| 15 | -0.00275673 | -0.075014 | -1.729873 | -0.091346 | 0.509841 | 2.458981 |
| 16 | -0.00070244 | -0.019114 | -0.440788 | -0.065102 | 2.441124 | 1.020146 |
| 17 | -0.00050340 | -0.013698 | -0.315886 | 0.107827 | -1.173535 | -2.171171 |
| 18 | -0.00064314 | -0.017501 | -0.403579 | 0.928427 | -3.550832 | -0.295383 |
| 19 | -0.00181677 | -0.049437 | -1.140043 | 0.952801 | 1.024231 | 3.315777 |
| 20 | -0.00017160 | -0.004669 | -0.107679 | 1.159310 | 1.359530 | -0.419974 |
| 21 | -0.00066738 | -0.018160 | -0.418785 | 1.458171 | 4.330841 | 1.590900 |
| 22 | -0.00027358 | -0.007445 | -0.171675 | 2.179706 | -1.225436 | 0.740200 |
| 23 | -0.00029801 | -0.008109 | -0.187002 | 2.410690 | -0.625680 | 1.696735 |
| 24 | -0.00009882 | -0.002689 | -0.062010 | 2.504444 | -4.186058 | -1.058878 |

25 -0.00017289 -0.004705 -0.108490 2.670126 2.847971 3.120360

26 -0.00345617 -0.094047 -2.168780 2.778133 0.310093 -2.748354

27 -0.00032188 -0.008759 -0.201981 2.960328 2.501499 -0.605180

28 -0.00052953 -0.014409 -0.332283 3.979641 -1.757637 -0.959505

29 -0.00039637 -0.010786 -0.248724 4.229552 1.862028 2.023986

Complex 34

# a.u. eV kcal/mol X/Y/Z coordinate(Angstrom)

1 -0.00024837 -0.006758 -0.155855 -4.045844 1.592872 -1.320346

2 -0.00560735 -0.152584 -3.518667 -3.964499 -0.531352 2.674345

3 -0.00539261 -0.146740 -3.383918 -3.762872 -2.726540 0.674606

4 -0.00218912 -0.059569 -1.373695 -2.873369 -0.654798 -2.929980

5 -0.00005386 -0.001466 -0.033795 -2.707086 3.929312 -1.557569

6 -0.00181620 -0.049421 -1.139686 -2.373340 -1.462973 3.868896

7 -0.00361931 -0.098486 -2.271151 -2.165174 -3.559584 3.420147

8 -0.00302291 -0.082258 -1.896907 -2.075521 0.789641 0.900475

9 -0.00011782 -0.003206 -0.073934 -2.059371 2.026523 -2.367460

10 -0.00242353 -0.065948 -1.520791 -1.870702 -1.595550 -0.534572

11 -0.00029808 -0.008111 -0.187049 -1.012140 3.398766 -0.808903

* 12 -0.01630309 -0.443630 -10.230350 -0.405658 -0.258899 2.732566

13 -0.00679228 -0.184827 -4.262221 -0.343270 -2.640546 1.091767

14 -0.00678824 -0.184717 -4.259687 0.357489 2.656724 1.094011

15 -0.01627918 -0.442979 -10.215347 0.404574 0.262891 2.725179

16 -0.00029838 -0.008119 -0.187235 0.982330 -3.399519 -0.817399

17 -0.00241309 -0.065663 -1.514238 1.820832 1.592872 -0.536168

18 -0.00302302 -0.082261 -1.896975 2.052061 -0.793813 0.880096

19 -0.00011800 -0.003211 -0.074045 2.097530 -2.065900 -2.355334

20 -0.00361671 -0.098416 -2.269521 2.138008 3.571843 3.413811

21 -0.00180774 -0.049191 -1.134376 2.369568 1.432264 3.843489

22 -0.00005406 -0.001471 -0.033924 2.676133 -3.916112 -1.563076

23 -0.00218707 -0.059513 -1.372408 2.836316 0.577924 -2.992409

24 -0.00539306 -0.146753 -3.384200 3.765738 2.757062 0.697749

25 -0.00559650 -0.152288 -3.511857 3.959903 0.542923 2.676200

26 -0.00024770 -0.006740 -0.155433 4.040867 -1.590126 -1.249469

Complex 35

# a.u. eV kcal/mol X/Y/Z coordinate(Angstrom)

1 -0.00250489 -0.068162 -1.571847 -4.451917 -1.355224 -1.892083

2 -0.00731951 -0.199174 -4.593063 -3.919195 -2.729975 0.860275

3 -0.00110487 -0.030065 -0.693320 -3.667422 2.173469 0.546129

4 -0.00656684 -0.178693 -4.120760 -3.548896 -0.294619 2.557002

5 -0.00149247 -0.040612 -0.936542 -3.502086 0.758998 -3.074116

6 -0.00371119 -0.100987 -2.328807 -2.664880 -1.828946 -2.681565

7 -0.00022854 -0.006219 -0.143413 -2.315890 3.614278 -1.339714

8 -0.00301787 -0.082121 -1.893746 -2.226243 -3.681751 2.235246

9 -0.00439237 -0.119522 -2.756255 -1.925112 -1.621289 -0.935567

10 -0.00275313 -0.074916 -1.727614 -1.879967 -1.519424 3.660577

11 -0.00386717 -0.105231 -2.426686 -1.858551 0.595606 0.695505

12 -0.00592786 -0.161305 -3.719792 -0.810956 0.796517 -3.687289

13 -0.00056250 -0.015306 -0.352975 -0.754932 0.965413 1.351420

14 -0.00914892 -0.248955 -5.741040 -0.611590 -3.107795 0.681550

15 -0.00379955 -0.103391 -2.384255 -0.425362 3.091605 -0.698870

16 -0.00853799 -0.232330 -5.357672 -0.231782 -0.724762 2.409305

17 -0.00008620 -0.002346 -0.054094 -0.256868 3.996491 1.710319

18 -0.00306987 -0.083535 -1.926376 0.304802 -0.405766 -2.548009

19 -0.00014019 -0.003815 -0.087968 0.780493 1.416202 3.757891

20 -0.00043159 -0.011744 -0.270828 1.280916 -1.247138 0.880054

21 -0.00327654 -0.089159 -2.056060 1.378807 -3.428856 -0.703913

22 -0.00672262 -0.182932 -4.218509 1.996386 0.228245 -3.607884

23 -0.00007246 -0.001972 -0.045467 2.421710 1.499733 2.657994

24 -0.00038364 -0.010439 -0.240739 2.947000 -3.900443 -0.847567

* 25 -0.01020422 -0.277671 -6.403250 3.062892 1.075139 -2.399173

26 -0.00029347 -0.007986 -0.184153 3.532834 -0.680642 1.710319

27 -0.00213256 -0.058030 -1.338201 4.287861 -1.301475 -0.643140

Complex 36

# a.u. eV kcal/mol X/Y/Z coordinate(Angstrom)

1 -0.00350728 -0.095438 -2.200851 -4.030247 -0.781762 2.661824

2 -0.00332657 -0.090521 -2.087456 -3.765951 -2.711621 0.399701

3 -0.00000861 -0.000234 -0.005401 -3.345344 3.741607 -1.402236

4 -0.00119619 -0.032550 -0.750624 -2.420803 -1.900417 3.760636

5 -0.00192942 -0.052502 -1.210728 -2.097562 -3.772296 1.984912

6 -0.00190628 -0.051873 -1.196211 -2.132420 0.791814 1.070237

7 -0.00131406 -0.035757 -0.824584 -1.771941 -1.476872 -0.573097

8 -0.00023252 -0.006327 -0.145909 -1.506461 0.404695 -3.808193

9 -0.00000180 -0.000049 -0.001127 -0.733143 -1.286173 -2.301913

* 10 -0.00945836 -0.257375 -5.935217 -0.553072 -0.540251 2.884402

11 -0.00411978 -0.112105 -2.585201 -0.427236 -2.707110 0.933389

12 -0.00001636 -0.000445 -0.010264 -0.321400 2.002955 -2.246725

13 -0.00001589 -0.000432 -0.009973 0.355313 -1.998682 -2.252503

14 -0.00402736 -0.109590 -2.527208 0.419448 2.679087 0.880860

15 -0.00935513 -0.254566 -5.870439 0.560471 0.556447 2.891733

16 -0.00000192 -0.000052 -0.001206 0.736954 1.306256 -2.250055

17 -0.00022980 -0.006253 -0.144200 1.567871 -0.436352 -3.801363

18 -0.00126708 -0.034479 -0.795104 1.701312 1.467198 -0.577620

19 -0.00187296 -0.050966 -1.175302 2.112815 -0.793626 1.067941

20 -0.00190146 -0.051741 -1.193188 2.094408 3.772196 1.928869

| | | | | | | |
|---|---|---|---|---|---|---|
| 21 | -0.00117666 | -0.032019 | -0.738369 | 2.431941 | 1.897342 | 3.740706 |
| 22 | -0.00000834 | -0.000227 | -0.005232 | 3.371226 | -3.731170 | -1.388144 |
| 23 | -0.00324498 | -0.088300 | -2.036255 | 3.809742 | 2.760075 | 0.448623 |
| 24 | -0.00343197 | -0.093389 | -2.153596 | 4.034405 | 0.847668 | 2.694266 |

# Calculated Coordinate

## Conformational Search

EQ0

| | | | |
|---|---|---|---|
| O | 0.873854922445 | -0.728615055157 | -2.857860184519 |
| O | 2.125495378746 | 0.392466092709 | -1.399041020258 |
| N | -1.581920301736 | 0.923050614906 | 1.367380239130 |
| N | 1.143208400258 | -2.445132682471 | -0.311383291143 |
| N | -1.184525005442 | 2.960995858823 | 0.583097877966 |
| C | 0.541685379628 | -0.358422567808 | 0.844654163720 |
| C | 0.476186412983 | -1.145550125254 | -0.477441888406 |
| C | -0.294535798207 | 0.884379005220 | 0.850347608975 |
| C | 1.234519426963 | -0.392799257219 | -1.587063936745 |
| C | -0.085094458291 | 2.157649031290 | 0.370014086894 |
| C | -2.061654150132 | 2.191076821126 | 1.187049801938 |
| H | 0.227822904065 | -1.027906040709 | 1.657382675071 |
| H | 1.591509105317 | -0.111284544353 | 1.022912254309 |
| H | -0.585984539049 | -1.226982176952 | -0.784044074550 |
| H | -2.053572170294 | 0.166943590651 | 1.842826973998 |
| H | 0.802752036942 | 2.530785860009 | -0.121403569618 |
| H | 0.610023597881 | -3.027028252686 | 0.332304349763 |
| H | 1.192394221399 | -2.949692225649 | -1.194683512434 |
| H | -3.047755979824 | 2.488918913643 | 1.518816814973 |
| H | 0.075890616345 | -1.282752860112 | -2.843865369058 |

EQ1

| | | | |
|---|---|---|---|
| O | 0.826038665519 | 0.624239193345 | -2.077667875920 |
| O | 2.167912729929 | -1.141493061278 | -2.250174740140 |
| N | -1.645744981186 | 0.751342122263 | 1.070535817897 |
| N | 1.328784272807 | -2.365271293417 | 0.095660173208 |
| N | -1.501062564220 | 2.908546594764 | 0.561970538700 |
| C | 0.728055973986 | -0.100101211774 | 0.840808440217 |
| C | 0.695544330373 | -1.123728627071 | -0.331573781004 |
| C | -0.308888621021 | 0.978419410162 | 0.780941685292 |
| C | 1.310320663684 | -0.556006064319 | -1.632937094632 |
| C | -0.257449598191 | 2.322322940053 | 0.467554585686 |
| C | -2.307945715754 | 1.939544482653 | 0.929151904594 |
| H | 0.608475610267 | -0.688098589245 | 1.758732087320 |
| H | 1.719071709731 | 0.365841276832 | 0.896582572209 |
| H | -0.357615592740 | -1.350105591137 | -0.555002581440 |
| H | -2.046075644016 | -0.128411862962 | 1.366317788624 |
| H | 0.617866920704 | 2.900787161036 | 0.199757003630 |
| H | 1.345960685114 | -3.002637362791 | -0.700427859275 |
| H | 2.316058072980 | -2.175958157560 | 0.276353849808 |
| H | -3.371792154287 | 2.032273795669 | 1.103621237522 |
| H | 0.194519688748 | 1.008421696883 | -1.436554891566 |

EQ2

| | | | |
|---|---|---|---|
| O | 1.030436943320 | -1.041221188542 | -2.805418197641 |
| O | 1.941643509101 | 0.555725450313 | -1.504321807816 |
| N | -1.580092660407 | 0.917132027335 | 1.358935531700 |
| N | 1.063983142870 | -2.449675634831 | -0.349382389417 |
| N | -1.174026428053 | 2.993693405227 | 0.689493498463 |
| C | 0.553207000354 | -0.335327082142 | 0.817531577673 |
| C | 0.454634492849 | -1.119187970576 | -0.505281461225 |
| C | -0.284485908662 | 0.906324445825 | 0.861012809753 |
| C | 1.226343844419 | -0.411297943062 | -1.618197240369 |
| C | -0.069903519519 | 2.203256821219 | 0.453117270094 |
| C | -2.060273458945 | 2.191553150051 | 1.235802221914 |
| H | 0.254611403459 | -1.011406853670 | 1.630951243599 |
| H | 1.603889471884 | -0.086236226863 | 0.987962066681 |
| H | -0.601740279907 | -1.154488802214 | -0.827068589224 |
| H | -2.062444300410 | 0.133762889178 | 1.775840142028 |
| H | 0.823858587576 | 2.600624143801 | -0.006747072671 |
| H | 0.529489050776 | -2.986480488630 | 0.331972670182 |
| H | 1.004784143211 | -2.955120153600 | -1.231602078203 |
| H | -3.053479408456 | 2.469544071261 | 1.563605779132 |
| H | 1.576793615001 | -0.567053335799 | -3.460662269755 |

EQ3

| | | | |
|---|---|---|---|
| O | 1.596293618699 | -1.276164300388 | -2.738369747729 |
| O | 1.704429673768 | 0.706355334747 | -1.700801594427 |
| N | -1.361701924149 | 1.032744292217 | 1.772404137185 |
| N | 0.519617133860 | -2.492896482287 | -0.710642198415 |
| N | -1.286171459856 | 2.990563384682 | 0.733032851929 |
| C | 0.451867441701 | -0.340735758847 | 0.680325842467 |
| C | 0.319746948836 | -1.031469549940 | -0.702139127353 |
| C | -0.339961554136 | 0.925817965868 | 0.838425964951 |
| C | 1.282528394476 | -0.424857687963 | -1.748950501986 |
| C | -0.323366488390 | 2.152546787031 | 0.212478016804 |

| | | | |
|---|---|---|---|
| C | -1.887398476656 | 2.290130002826 | 1.668346250571 |
| H | 0.111675575725 | -1.061280537392 | 1.436222387158 |
| H | 1.514596564025 | -0.155400185926 | 0.880115018673 |
| H | -0.692360999506 | -0.852663547304 | -1.086986564996 |
| H | -1.638354162240 | 0.321350349295 | 2.433822692638 |
| H | 0.333367449892 | 2.463905710170 | -0.586026153474 |
| H | -0.288873596472 | -2.976938000101 | -0.327404519939 |
| H | 1.322762252542 | -2.749602303245 | -0.136712638152 |
| H | -2.699401534235 | 2.631332378587 | 2.297203837707 |
| H | 1.203452330204 | -2.142047121750 | -2.469130310165 |

EQ4

| | | | |
|---|---|---|---|
| O | 1.707466636911 | -1.782859870449 | -2.595898115820 |
| O | 3.015418548719 | -0.593624247701 | -1.212316277742 |
| N | -0.742572142736 | 1.183376921243 | 2.132216740641 |
| N | -0.526382826871 | -1.028937388035 | -1.509318812968 |
| N | -2.188418433270 | 2.466110075819 | 1.041779571912 |
| C | 0.741068461957 | 0.225579237864 | 0.325021463797 |
| C | 0.622036434052 | -0.178968396772 | -1.153288479970 |
| C | -0.407646557141 | 1.067131464773 | 0.794380365091 |
| C | 1.917769930932 | -0.870240223989 | -1.636532088926 |
| C | -1.322093778120 | 1.870730620009 | 0.149302028848 |
| C | -1.810564496958 | 2.034524032974 | 2.222754289556 |
| H | 0.813278874830 | -0.689095790915 | 0.933103366447 |
| H | 1.700063926716 | 0.742737648074 | 0.451484705960 |
| H | 0.538264997325 | 0.732694769711 | -1.760058254561 |
| H | -0.265046511661 | 0.735275309359 | 2.901694814073 |
| H | -1.401928362425 | 2.070008992151 | -0.911879192022 |
| H | -0.678899635783 | -1.733556092036 | -0.787374498104 |
| H | -1.380631166701 | -0.478583712248 | -1.556150250168 |
| H | -2.264794757213 | 2.293780731133 | 3.169903928487 |
| H | 0.723485480664 | -1.821842444748 | -2.700665749962 |

EQ5

| | | | |
|---|---|---|---|
| O | 1.985258565655 | -0.694772847447 | -2.612570205145 |
| O | 2.456440610451 | -2.161787407282 | -1.005162690418 |
| N | -1.230256500932 | 0.685752343492 | 1.398534918586 |
| N | 0.117983310376 | -1.682413477216 | 0.435215984430 |
| N | -1.990916972798 | 2.762205238624 | 1.174315874931 |
| C | 0.833631708391 | 0.633097247749 | -0.050606187527 |
| C | 0.512390692637 | -0.768110295175 | -0.640274034121 |
| C | -0.360509444159 | 1.319361547356 | 0.532430367121 |
| C | 1.729940079090 | -1.293459015853 | -1.421610329534 |
| C | -0.853119815238 | 2.599955132003 | 0.416158692192 |
| C | -2.190435322760 | 1.590635439779 | 1.743352869899 |
| H | 1.625584875537 | 0.495405124149 | 0.701212635501 |
| H | 1.262748610005 | 1.278870733244 | -0.825553748690 |
| H | -0.336917970857 | -0.666144055274 | -1.328968387212 |
| H | -1.160264936948 | -0.307552700264 | 1.600433916199 |
| H | -0.445054171643 | 3.416574006259 | -0.166187653316 |
| H | -0.284591577199 | -2.525578553549 | 0.026796920769 |
| H | 0.974348566312 | -2.000755388657 | 0.893308022327 |
| H | -3.007576791930 | 1.343769456884 | 2.408302273611 |
| H | 1.295145705761 | -0.041460171293 | -2.817743415331 |

EQ6

| | | | |
|---|---|---|---|
| O | 0.746879687845 | -0.718558403601 | -2.837019492597 |
| O | 1.920542990594 | 0.569848197075 | -1.457009848922 |
| N | -1.617359245282 | 0.289649475415 | 1.617952355555 |
| N | 1.476386680446 | -2.397067372166 | -0.373033229132 |
| N | -1.118413447712 | 2.802993611658 | 0.589747385534 |
| C | 0.643999012065 | -0.471852034182 | 0.901814918301 |
| C | 0.601717681043 | -1.215669193111 | -0.445320193074 |
| C | -0.480456007314 | 0.545401238940 | 1.068315613595 |
| C | 1.147814073252 | -0.343311660919 | -1.591681145519 |
| C | -0.215954806821 | 1.878647836594 | 0.530185371758 |
| C | -2.066530263418 | 3.549642941916 | 0.530592272015 |
| H | 0.552342913960 | -1.216591721044 | 1.701781212915 |
| H | 1.629057979607 | -0.005059095508 | 0.995150193681 |
| H | -0.454607138292 | -1.461005660802 | -0.672103648818 |
| H | -1.641699349039 | -0.682928280510 | 1.941126160277 |
| H | 0.734333378277 | 2.120708090339 | 0.068967205842 |
| H | 1.101291689461 | -3.058973860336 | 0.304116433096 |
| H | 1.514317499107 | -2.880504912913 | -1.268935517243 |
| H | -2.309572041549 | 4.269147732456 | 1.314357791873 |
| H | 0.048741582444 | -1.391921000437 | -2.777253562065 |

EQ7

| | | | |
|---|---|---|---|
| O | 0.932239605390 | 0.535722709647 | -2.066581066749 |
| O | 2.578050155182 | -0.889451120349 | -1.609547334320 |
| N | -0.205446010450 | 1.082079925495 | 1.224926279294 |
| N | 1.235542680393 | -1.394036899269 | 0.828228295741 |
| N | -1.476833088676 | 2.787708566453 | 0.577266654138 |
| C | -0.909523746097 | -0.820819990898 | -0.273248518093 |

C 0.529918667287 -1.337818833651 -0.469304572861
C -0.995195652730 0.599836891392 0.198180797039
C 1.437800804393 -0.530630510323 -1.425058462270
C -1.772206225310 1.677209872877 -0.180739829045
C -0.529288889468 2.393795623803 1.403644679376
H -1.464910164615 -0.920548726485 -1.213247155614
H -1.405010920404 -1.497804658396 0.438667999478
H 0.463611719081 -2.330863255898 -0.944338118810
H 0.540683164688 0.521937216165 1.633400417556
H -2.540473586698 1.704759855976 -0.943639717476
H 0.891428002243 -2.189257212307 1.365555568792
H 2.221164511935 -1.574331551084 0.635209370099
H -0.042510101720 3.015231771038 2.143544399947
H 0.096071256008 0.832840107500 -1.650522726897

EQ8

O 1.730794050141 -0.221582669035 -2.522602250856
O -0.348769653082 -1.000574087903 -2.218252382870
N -1.373670841984 0.902712093089 -0.187455765386
N 2.357545184683 -0.264281160014 -0.008759730809
N -1.199922620937 2.410201642257 1.437292289627
C 0.009620068690 -1.046587988021 0.639760781556
C 1.201999514839 -1.110616066091 -0.343779171516
C -0.577554089037 0.321946030339 0.784702567116
C 0.763256824703 -0.770966153623 -1.786789195676
C -0.488893478065 1.279607692286 1.772465477794
C -1.713908747590 2.147925391748 0.253593541808
H -0.749952649137 -1.758774063646 0.298362833958
H 0.354420222657 -1.389066084807 1.621826893827
H 1.558885096762 -2.148593187639 -0.385084142420
H -1.588423190417 0.470852839475 -1.079017334344
H 0.028940627840 1.202894967408 2.720651650422
H 2.029430937791 0.614747754511 0.396203235378
H 2.954631911606 -0.710353703527 0.683195368451
H -2.336859746005 2.816059099632 -0.326243659823
H 2.475522191082 -0.072015544652 -1.882483841497

EQ9

O 1.094051401366 0.479968738422 -2.191420137745
O -0.649195524932 -0.979879901735 -1.962997412515
N -0.342731985007 0.708207814994 -0.323445641635
N 2.440733763028 -0.551353958460 -0.182184023646
N -1.378717627624 2.231878338663 0.903369122638
C 0.251645553224 -1.158136130298 0.984567339222
C 1.098952601322 -1.134394437208 -0.320201094005
C -0.400222551749 0.196807416802 0.955393881886
C 0.298646671477 -0.202882259142 -1.296171656998
C -1.044070968644 1.153742906198 1.701357879669
C -0.946344733218 1.931945239510 -0.307443939732
H -0.491396154023 -1.964805773449 0.938449115736
H 0.873986335818 -1.334984338389 1.868376468051
H 1.195826327527 -2.117553955004 -0.784722662625
H -1.029236204718 -0.430856596986 -2.669084244749
H -1.282560120732 1.145623946439 2.756554776679
H 2.461373736485 0.133328575899 0.572724867142
H 3.132019334609 -1.264322681764 0.032338651113
H -1.021772217788 2.562399999759 -1.182556924835
H 2.000754740299 0.411513669562 -1.805563964291

EQ10

O 1.788041697997 -1.494283776947 -2.653968038941
O -0.059104590328 -0.269014626343 -2.330982757643
N -1.254926366506 1.086918586075 -0.144630048987
N 2.252539661300 0.234381797698 -0.179347624081
N -1.525866318902 2.205436198169 1.758194381747
C 0.100686515342 -0.947674293942 0.558881606079
C 1.327249738735 -0.881829321756 -0.396545534048
C -0.597513147184 0.350769337573 0.824390951321
C 0.917657726500 -0.833994893178 -1.870441295858
C -0.784445914709 1.068755296382 1.986139216775
C -1.784810939718 2.184402056637 0.466196089468
H -0.603344403606 -1.703870668396 0.182191588587
H 0.462570196799 -1.321659632677 1.523710450236
H 1.903301779664 -1.804889734039 -0.277445780618
H -1.231348117388 0.849761629430 -1.131458273142
H -0.434554905428 0.816185152206 2.979478522086
H 1.716991341440 1.086835736020 -0.017067764634
H 2.792027776522 0.068660401364 0.667743863696
H -2.345811659441 2.933811342967 -0.076666974200
H 1.485814996368 -1.369605216560 -3.574263375669

EQ11

O 2.193863277363 -0.037438918928 -2.149037233967
O 0.231886572984 -1.001335062741 -2.674027982080

| | | | |
|---|---|---|---|
| N | -1.759605145063 | 0.680466685387 | 1.424252715392 |
| N | 2.087520624210 | -0.560524506714 | 0.387214372082 |
| N | -1.058831391137 | 2.626107761088 | 0.619400740116 |
| C | -0.415995887378 | -1.051091888135 | 0.169974031122 |
| C | 1.015062853443 | -1.206520523449 | -0.386899587889 |
| C | -0.791036851233 | 0.368480897418 | 0.485076462802 |
| C | 1.085977971476 | -0.733414687408 | -1.861936586763 |
| C | -0.377845318736 | 1.594756304392 | 0.007851616160 |
| C | -1.883333831069 | 2.042675582514 | 1.457987505418 |
| H | -1.097581317699 | -1.479209197661 | -0.575599485842 |
| H | -0.506022537542 | -1.662162301787 | 1.078713762686 |
| H | 1.235620374165 | -2.279854754725 | -0.430808407694 |
| H | -2.296736783818 | 0.015486329974 | 1.962773884836 |
| H | 0.365216307004 | 1.793582460035 | -0.753513337426 |
| H | 1.725928621253 | 0.265671739030 | 0.863554229027 |
| H | 2.470028524536 | -1.183384628390 | 1.093203977993 |
| H | -2.583510180580 | 2.543487441318 | 2.113519676889 |
| H | 2.693444372484 | -0.000229055784 | -1.291945577190 |

EQ12

| | | | |
|---|---|---|---|
| O | 1.829534678454 | -1.179366985943 | -2.763199489923 |
| O | -0.193193484732 | -0.426725005768 | -2.274180341535 |
| N | -1.357526233429 | 1.008158799886 | -0.137379775620 |
| N | 2.249886160144 | 0.077331136944 | 0.016034376671 |
| N | -1.417990802145 | 2.288106100200 | 1.679793659012 |
| C | 0.086975079256 | -0.961194001312 | 0.581599531161 |
| C | 1.291925218317 | -0.957653501791 | -0.385936455824 |
| C | -0.580789138208 | 0.361802804580 | 0.807558228633 |
| C | 0.870481739205 | -0.835123632396 | -1.863343115159 |
| C | -0.641612798510 | 1.177375524892 | 1.917709168528 |
| C | -1.825229893412 | 2.153625457614 | 0.433374167809 |
| H | -0.636293152315 | -1.707830294539 | 0.230182497341 |
| H | 0.463038200104 | -1.307901890208 | 1.548866850356 |
| H | 1.794892485280 | -1.931886076862 | -0.280530581806 |
| H | -1.436722259741 | 0.694588081444 | -1.098366954685 |
| H | -0.174994463371 | 1.012190982398 | 2.880650284399 |
| H | 2.993065451822 | 0.198482699591 | -0.669327962639 |
| H | 1.766244609799 | 0.969967752465 | 0.106977329000 |
| H | -2.455503672166 | 2.851080542765 | -0.102692968864 |
| H | 2.593852316592 | -1.571529919605 | -2.308226655335 |

EQ13

| | | | |
|---|---|---|---|
| O | 1.617820511355 | -1.507405480522 | -2.616397216104 |
| O | 0.116857471022 | 0.120066010304 | -2.340346885456 |
| N | -1.281329780985 | 1.259425062239 | -0.182715378939 |
| N | 2.464183147029 | -1.524178326518 | -0.192025268482 |
| N | -2.216860381918 | 1.671763084565 | 1.792515884847 |
| C | 1.070592606417 | 0.520281889398 | 0.428261937641 |
| C | 1.174794873921 | -0.821375914249 | -0.344069635846 |
| C | -0.314577439481 | 0.980367152206 | 0.762093892915 |
| C | 0.908340865185 | -0.668468537423 | -1.861952522322 |
| C | -0.920786261518 | 1.246738265697 | 1.970543322700 |
| C | -2.397942522995 | 1.661450715900 | 0.486528681469 |
| H | 1.600964165407 | 0.388124537428 | 1.378357216858 |
| H | 1.619803623394 | 1.290201018159 | -0.133661750200 |
| H | 0.396127540988 | -1.499093380395 | 0.028436314104 |
| H | -1.142010461852 | 1.098459688343 | -1.176733495160 |
| H | -0.487214884844 | 1.162713098469 | 2.959484529117 |
| H | 3.233995199000 | -0.855982854303 | -0.157394174220 |
| H | 2.492351528883 | -2.056382587709 | 0.674152954393 |
| H | -3.312114616787 | 1.932383458095 | -0.025040669751 |
| H | 2.211617500251 | -1.981076339255 | -1.975767671462 |

EQ14

| | | | |
|---|---|---|---|
| O | 2.331995128638 | -1.329323759474 | -2.526874484062 |
| O | 0.646049982262 | -2.577013000531 | -1.727903397423 |
| N | -1.075433002219 | 2.083357454023 | 0.336108321657 |
| N | 1.558248327109 | 0.867433827030 | -1.368960259114 |
| N | -1.384804786783 | 1.819642930445 | 2.393135052894 |
| C | -0.578382780723 | -0.277506844572 | -0.520330043867 |
| C | 0.937133452517 | -0.345907702160 | -0.813235594634 |
| C | -0.937319570335 | 0.705026081338 | 0.548873473750 |
| C | 1.267130310265 | -1.540304382878 | -1.738282872207 |
| C | -1.137075601429 | 0.546768855076 | 1.884784824027 |
| C | -1.368007870757 | 2.820088534007 | 1.457330091189 |
| H | -1.109251648875 | -0.039211745732 | -1.453890448941 |
| H | -0.906032709339 | -1.281035405713 | -0.236056637954 |
| H | 1.455749588114 | -0.553610611551 | 0.131911790739 |
| H | -1.036802734232 | 2.514328201349 | -0.575700203735 |
| H | -1.124786686974 | -0.345603195600 | 2.492907785124 |
| H | 0.964877592281 | 1.281644279973 | -2.087657776509 |
| H | 1.699537437797 | 1.573107942989 | -0.650132130565 |
| H | -1.584123323084 | 2.008664310140 | 3.363913331189 |
| H | 2.607605595145 | -0.397104986382 | -2.344174951968 |

EQ15

| | | | |
|---|---|---|---|
| O | 2.226700438265 | -1.864896946155 | -2.178751176019 |
| O | 1.460678091618 | 0.028958888343 | -3.110247456920 |
| N | -1.900396588694 | 1.290094185658 | 0.268468185220 |
| N | 1.169059577104 | -1.425680292001 | 0.155440925131 |
| N | -1.680986580409 | 1.439831670072 | 2.473208344746 |
| C | 0.466868395450 | 0.927048353960 | -0.556267329588 |
| C | 0.627293952338 | -0.569435926326 | -0.910806484227 |
| C | -0.541984533957 | 1.174005721988 | 0.519243039740 |
| C | 1.484843218906 | -0.748103926507 | -2.184196104338 |
| C | -0.444900519595 | 1.264852959963 | 1.889806522563 |
| C | -2.531148320479 | 1.454371223011 | 1.471398110342 |
| H | 1.440999953202 | 1.315730903309 | -0.232825111851 |
| H | 0.216286895428 | 1.467705382379 | -1.475382851132 |
| H | -0.360005364579 | -0.977002816738 | -1.167581329364 |
| H | -2.334600346113 | 1.308525638682 | -0.643916305124 |
| H | 0.456071306456 | 1.231388201550 | 2.489449887682 |
| H | 1.916100999873 | -0.938641081616 | 0.651453941646 |
| H | 0.454308244414 | -1.633607102664 | 0.849239711760 |
| H | -3.602737144176 | 1.582305440074 | 1.549540764007 |
| H | 2.061067167055 | -2.278295102066 | -1.294550367095 |

EQ16

| | | | |
|---|---|---|---|
| O | 0.486879786203 | -1.802669880282 | -2.280283839097 |
| O | 0.759065467010 | 0.401106866038 | -2.302532820029 |
| N | -1.077523841891 | 1.435569168082 | -0.240827387564 |
| N | 2.835799610573 | -1.033098339862 | -0.300752562627 |
| N | -2.362645944235 | 1.306834248281 | 1.567666479392 |
| C | 1.161446819373 | 0.547680338597 | 0.550133219027 |
| C | 1.393889959620 | -0.745227962730 | -0.252574678996 |
| C | -0.275046006342 | 0.910932000390 | 0.758238740413 |
| C | 0.824980721623 | -0.634893851779 | -1.677230494291 |
| C | -1.098461615590 | 0.846780126246 | 1.860060110390 |
| C | -2.312943342971 | 1.649538932818 | 0.297183852865 |
| H | 1.643758360569 | 0.410554721836 | 1.522183126850 |
| H | 1.691317628213 | 1.362470307014 | 0.038864200128 |
| H | 0.905919112065 | -1.576478300184 | 0.274091189347 |
| H | -0.779408499257 | 1.577161335683 | -1.198106156750 |
| H | -0.842154590206 | 0.501852622965 | 2.853892438807 |
| H | 3.022314219102 | -1.903929729125 | -0.796074897711 |
| H | 3.310344962667 | -0.295296243301 | -0.821245936892 |
| H | -3.132586807420 | 2.055868018148 | -0.280672579679 |
| H | 0.516186549525 | -2.531486110021 | -1.637666717198 |

EQ17

| | | | |
|---|---|---|---|
| O | 2.232902867521 | -1.848732984431 | -2.176095443613 |
| O | 1.556444724529 | 0.092727841638 | -3.079099863588 |
| N | -1.919475759279 | 1.118053963451 | 0.316928839625 |
| N | 1.068069383585 | -1.465958149155 | 0.115570342728 |
| N | -1.698390513298 | 1.500644637717 | 2.368264525605 |
| C | 0.475569772886 | 0.932145916876 | -0.544688734883 |
| C | 0.603790053989 | -0.556068354046 | -0.943451999543 |
| C | -0.536942086882 | 1.169815761898 | 0.528528513972 |
| C | 1.516924482605 | -0.715168658862 | -2.180706164803 |
| C | -0.403977703537 | 1.412741927614 | 1.860312166587 |
| C | -2.687402133709 | 1.327848795999 | 1.435353030946 |
| H | 1.455392059348 | 1.292585369472 | -0.206818827805 |
| H | 0.238406884755 | 1.503048085693 | -1.449331480127 |
| H | -0.381691328391 | -0.922774980933 | -1.260891999394 |
| H | -2.341314423941 | 0.983815451064 | -0.590766414682 |
| H | 0.482298876694 | 1.544109048622 | 2.463586898802 |
| H | 1.782976212238 | -1.010604311023 | 0.683308952405 |
| H | 0.307579211714 | -1.703849499818 | 0.747870872057 |
| H | -1.913017283599 | 1.694697576127 | 3.334677896568 |
| H | 2.015261241526 | -2.284929598786 | -1.314294136085 |

EQ18

| | | | |
|---|---|---|---|
| O | 1.569037269858 | -1.630885182241 | -2.587747155015 |
| O | 0.174095955590 | 0.098627626606 | -2.353720550897 |
| N | -1.297604639234 | 1.261828271325 | -0.176832907169 |
| N | 2.429784511500 | -1.591832210368 | -0.158039862060 |
| N | -2.145146090470 | 1.672990064112 | 1.702494660581 |
| C | 1.084181738142 | 0.505716620261 | 0.401940267970 |
| C | 1.162107867803 | -0.857634011691 | -0.335799490110 |
| C | -0.294069117446 | 0.978853144723 | 0.749466053026 |
| C | 0.910186638906 | -0.727599844911 | -1.857068976982 |
| C | -0.847176155912 | 1.246142198848 | 1.964043152662 |
| C | -2.476520458530 | 1.694338847317 | 0.368623912635 |
| H | 1.642949206355 | 0.400679623400 | 1.339131405145 |
| H | 1.612291779186 | 1.259549532675 | -0.199042867695 |
| H | 0.364253961541 | -1.507783537179 | 0.045179911579 |
| H | -1.153114030073 | 1.112717375498 | -1.169155467612 |
| H | -0.428687218010 | 1.179334175820 | 2.957772575427 |

| | | | |
|---|---|---|---|
| H | 3.221161568127 | -0.948455936433 | -0.169604429152 |
| H | 2.451710472390 | -2.075533999005 | 0.736394241433 |
| H | -2.803731517011 | 1.947054119154 | 2.415414084191 |
| H | 2.136903531669 | -2.118825339760 | -1.937532808603 |

EQ19

| | | | |
|---|---|---|---|
| O | 1.501833765965 | -1.544254513156 | -2.831410871526 |
| O | 0.043671847679 | 0.038918074292 | -2.255118044337 |
| N | -0.919793978023 | 1.123993423775 | 0.118775527264 |
| N | 2.718898946818 | -1.647048843486 | -0.576923792324 |
| N | -2.453548400770 | 1.901021617195 | 1.693425191643 |
| C | 1.425935729441 | 0.362343123553 | 0.333796801693 |
| C | 1.417032386854 | -0.956422751296 | -0.491674744374 |
| C | 0.116218455979 | 0.827512972201 | 0.900817121793 |
| C | 0.913440308142 | -0.754720809576 | -1.939711513531 |
| C | -2.662450600335 | 2.130822008627 | 2.840451435998 |
| C | -2.136894752674 | 1.615877363831 | 0.408568562241 |
| H | 2.087872707005 | 0.187410084945 | 1.189118948602 |
| H | 1.921178677059 | 1.136981730500 | -0.277645042053 |
| H | 0.710129218216 | -1.652968811786 | -0.023454373061 |
| H | -0.765135282077 | 0.906307413755 | -0.877703017506 |
| H | 0.007189578957 | 1.014984947587 | 1.960558618974 |
| H | 3.480384016847 | -0.970085803004 | -0.625320843267 |
| H | 2.883911043348 | -2.224086897554 | 0.243972563363 |
| H | -2.858556003922 | 1.739277568635 | -0.382430298038 |
| H | 2.197557854283 | -2.031215844337 | -2.312285313296 |

EQ20

| | | | |
|---|---|---|---|
| O | 1.839871665548 | -0.882122402949 | -2.976136469053 |
| O | 0.079625591112 | 0.326846658538 | -2.325178089563 |
| N | -0.916442085525 | 0.909779413076 | 0.233909121394 |
| N | 2.734987018540 | -1.434933091644 | -0.648854417901 |
| N | -2.525072353770 | 1.590479801647 | 1.816058375886 |
| C | 1.411220670885 | 0.472298429629 | 0.396697000349 |
| C | 1.433522314685 | -0.732352421596 | -0.599258935788 |
| C | 0.124168498530 | 0.959432862770 | 1.043888046714 |
| C | 1.045562192458 | -0.362866957345 | -2.046018103844 |
| C | -2.715013170376 | 1.644426158511 | 3.030000165882 |
| C | -2.225964416114 | 1.301316475115 | 0.612459477145 |
| H | 2.088605344609 | 0.190582601634 | 1.213132463445 |
| H | 1.879279891147 | 1.352683344477 | -0.075166777811 |
| H | 0.679446998461 | -1.458294326600 | -0.267519219138 |
| H | -0.844574476260 | 0.650894589806 | -0.765695398955 |
| H | -2.593082420942 | 2.631893240789 | 3.498636717985 |
| H | 3.490865245367 | -0.804685177515 | -0.384327125694 |
| H | 2.761101704831 | -2.217820536832 | -0.000859066603 |
| H | -2.969409812273 | 1.350550801653 | -0.174127469911 |
| H | 2.516793413306 | -1.398030680270 | -2.455504845452 |

EQ21

| | | | |
|---|---|---|---|
| O | 1.340490942165 | -1.662469985841 | -2.283283743057 |
| O | -0.379577543609 | -0.266479905302 | -2.019556291758 |
| N | -1.185813831548 | 1.899011570316 | -0.385782943368 |
| N | 2.537352785279 | -1.075684700133 | -0.086606771793 |
| N | -1.702228767203 | -0.459745287684 | 2.100823043782 |
| C | 1.010761651862 | 0.881051089990 | 0.381585094500 |
| C | 1.154168872867 | -0.566082602971 | -0.142028891017 |
| C | -0.404736788897 | 1.400286836551 | 0.756362679527 |
| C | 0.622792012693 | -0.791832262152 | -1.578161749030 |
| C | -1.152369522272 | 0.374901645059 | 1.510251164144 |
| C | -1.591005477250 | 3.149939823319 | -0.404810267262 |
| H | 1.612675048467 | 0.936575599449 | 1.296254191786 |
| H | 1.439595148489 | 1.594601890520 | -0.332061738508 |
| H | 0.547113382181 | -1.226378103649 | 0.491104234660 |
| H | -1.286700376463 | 1.192815512953 | -1.125077335436 |
| H | -0.293637248883 | 2.281387755790 | 1.397375128183 |
| H | 3.209598201656 | -0.337599734554 | -0.292412248026 |
| H | 2.763254917509 | -1.444713099565 | 0.833504689033 |
| H | -2.118669005877 | 3.266099793487 | -1.376811315460 |
| H | 2.101365361433 | -1.903552471684 | -1.691458345631 |

EQ22

| | | | |
|---|---|---|---|
| O | 1.314321866897 | -1.570349576405 | -2.458576847037 |
| O | 0.273955155385 | 0.404019122115 | -2.380283531777 |
| N | -1.077521137246 | 1.571530995343 | -0.221739713473 |
| N | 2.084315555586 | -1.582750623487 | -0.007673470987 |
| N | -2.016495998768 | 0.686073885996 | 1.641092719809 |
| C | 1.259577776284 | 0.807469427918 | 0.353461509380 |
| C | 1.028585185674 | -0.586106402739 | -0.272731883074 |
| C | -0.025770750543 | 1.517986267539 | 0.802265021349 |
| C | 0.830668912249 | -0.514149761740 | -1.808010633533 |
| C | -0.718773302898 | 0.898372929886 | 2.026319980789 |
| C | -2.152489259825 | 1.051912313446 | 0.350154111908 |
| H | 1.857321500631 | 0.684963583209 | 1.262364687230 |

| | | | |
|---|---|---|---|
| H | 1.827686465957 | 1.435819558936 | -0.342488280797 |
| H | 0.099052220255 | -1.007318888670 | 0.132002423385 |
| H | -0.899647408863 | 1.605825919693 | -1.223974396122 |
| H | 0.224933482912 | 2.546554857669 | 1.105448799369 |
| H | 3.002061740666 | -1.138550258372 | 0.008051400768 |
| H | 1.949575411600 | -2.017620628004 | 0.902086946792 |
| H | -3.078498181300 | 0.933554086478 | -0.210609829179 |
| H | 1.754909692660 | -2.117167362643 | -1.753773888660 |

EQ23

| | | | |
|---|---|---|---|
| O | 1.907557656824 | -0.745823897765 | -2.639824225831 |
| O | -0.301140119202 | -0.645499017250 | -2.256838720591 |
| N | -1.414019512165 | 0.949248032244 | -0.136543434450 |
| N | 2.299121818741 | -0.163191721975 | 0.119423419282 |
| N | -1.285840158146 | 2.361021715750 | 1.575223432150 |
| C | 0.030197950489 | -1.008440055525 | 0.588814819673 |
| C | 1.240218521924 | -1.043754201370 | -0.368780454672 |
| C | -0.585048924535 | 0.343219957588 | 0.791556249446 |
| C | 0.827763141343 | -0.785690046001 | -1.825067379216 |
| C | -0.528809146425 | 1.240653331790 | 1.836337450732 |
| C | -1.798472501916 | 2.151412725872 | 0.380199854138 |
| H | -0.721619532374 | -1.722185220924 | 0.231867910288 |
| H | 0.387660575320 | -1.368843056144 | 1.557770707582 |
| H | 1.640559048991 | -2.068259389616 | -0.364461972532 |
| H | -1.600506398026 | 0.567250917045 | -1.055606641011 |
| H | 0.007886925427 | 1.128840186962 | 2.769836986365 |
| H | 3.066973245236 | -0.124125457850 | -0.546724467108 |
| H | 1.933591864771 | 0.782003863371 | 0.229506231731 |
| H | -2.451241679227 | 2.828993802322 | -0.154525221385 |
| H | 1.570902043386 | -0.584271087121 | -3.541688178034 |

EQ24

| | | | |
|---|---|---|---|
| O | 2.628685685693 | 0.358411712611 | -1.334064961596 |
| O | 1.265265289396 | -0.684486541928 | -2.793468639269 |
| N | -0.796695281134 | 0.828132553678 | 1.323567334303 |
| N | 1.618914937430 | -0.874416160352 | 0.867008174305 |
| N | -1.668213042212 | 2.532990055311 | 0.198032712526 |
| C | -0.507026194611 | -0.977148079163 | -0.418425527488 |
| C | 1.029141750201 | -1.206842336088 | -0.431354784412 |
| C | -0.916429015062 | 0.394675071673 | 0.012533950350 |
| C | 1.631487282697 | -0.502586099151 | -1.653926020535 |
| C | -1.458473233070 | 1.470349080326 | -0.653805350487 |
| C | -1.257490119056 | 2.113303815366 | 1.375369733170 |
| H | -0.893819545355 | -1.169996447143 | -1.422696192742 |
| H | -0.938704803826 | -1.729021103856 | 0.253462871430 |
| H | 1.185187256056 | -2.277788427193 | -0.609776398247 |
| H | -0.384966529179 | 0.282215301164 | 2.068653912613 |
| H | -1.716263996754 | 1.530968169979 | -1.703242149147 |
| H | 2.540093027036 | -1.301430266304 | 0.944668125254 |
| H | 1.784227323821 | 0.129984901637 | 0.907164231172 |
| H | -1.271894611455 | 2.687112119602 | 2.292749256480 |
| H | 2.914952278081 | 0.762227070784 | -2.175552387810 |

EQ25

| | | | |
|---|---|---|---|
| O | 1.370878493541 | -0.421699869147 | -2.748787541482 |
| O | -0.378634924708 | -1.529354216278 | -1.875053471669 |
| N | -0.187625920006 | 1.495287035449 | 0.022096165911 |
| N | 2.484796979962 | 0.091786839008 | -0.384018493594 |
| N | -2.063311915193 | 1.919285331020 | 1.430422639475 |
| C | 0.370036698079 | -0.809085161573 | 0.748802457386 |
| C | 1.422630349060 | -0.904204392726 | -0.383158532975 |
| C | -0.418824911232 | 0.468039705483 | 0.767921220094 |
| C | 0.694031715467 | -0.979662775855 | -1.728005246128 |
| C | -1.587622386427 | 0.745054931091 | 1.643738231000 |
| C | -1.207625026772 | 2.468369091651 | 0.385382068326 |
| H | -0.328984732016 | -1.649712018301 | 0.664661567348 |
| H | 0.877704051966 | -0.925343702892 | 1.717793213068 |
| H | 1.901676770578 | -1.889416191538 | -0.279427270871 |
| H | -0.740331383142 | 3.397790183878 | 0.736787523153 |
| H | -2.015375904532 | 0.064314100920 | 2.376868594130 |
| H | 2.067077606232 | 1.016445917519 | -0.474215904653 |
| H | 2.975262007762 | 0.064917255260 | 0.507881973587 |
| H | -1.814629322125 | 2.723377248224 | -0.493300181578 |
| H | 0.808914135884 | -0.558022860608 | -3.535430890215 |

EQ26

| | | | |
|---|---|---|---|
| O | 1.228855267598 | 0.404086238335 | -2.054381609624 |
| O | 0.526597775445 | -1.700134798073 | -2.454656936926 |
| N | -1.620509747201 | 0.727698379170 | -0.122747987672 |
| N | 2.390282697840 | -0.318415883135 | 0.245152072764 |
| N | -0.919094746062 | 2.506014663551 | 1.307480489909 |
| C | -0.060198667775 | -1.046429674758 | 0.583615870889 |
| C | 1.280071473433 | -1.139622855517 | -0.222213058964 |
| C | -0.653175743529 | 0.330771585948 | 0.634086751718 |

| | | | |
|---|---|---|---|
| C | 0.982181983920 | -0.877553238004 | -1.694598581302 |
| C | -0.226622564747 | 1.444480314576 | 1.521055935484 |
| C | -1.843254509472 | 2.115660900313 | 0.248140118436 |
| H | -0.787779809420 | -1.730460319857 | 0.136038057763 |
| H | 0.149170557609 | -1.396690551572 | 1.603259178206 |
| H | 1.590964236038 | -2.188299133421 | -0.175627333856 |
| H | -1.699234535639 | 2.765337874035 | -0.625586722133 |
| H | 0.559061225788 | 1.403604572738 | 2.273027043010 |
| H | 2.198423802756 | 0.670830527115 | 0.108298653915 |
| H | 2.557926366859 | -0.475738090413 | 1.236554992707 |
| H | -2.878109899157 | 2.257879359841 | 0.587045047914 |
| H | 0.958080537019 | 0.480440438871 | -2.988864823318 |

EQ27

| | | | |
|---|---|---|---|
| O | 0.965375342179 | -2.016022421438 | -2.379863714392 |
| O | 0.533445500562 | 0.188022444978 | -2.500415871396 |
| N | -1.106528120190 | 1.129226285265 | -0.163918221029 |
| N | 2.261783974540 | 0.473370235562 | -0.124167004541 |
| N | -1.712785586936 | 1.959870983563 | 1.669432751693 |
| C | 0.320837748802 | -0.896922558463 | 0.570154148717 |
| C | 1.493900073404 | -0.720740987981 | -0.437282827396 |
| C | -0.513524914186 | 0.325356022763 | 0.818849827948 |
| C | 0.951994531450 | -0.764337173450 | -1.870580965247 |
| C | -0.910030089596 | 0.872791956959 | 1.998808410981 |
| C | -1.856305218461 | 2.170576907828 | 0.321329190865 |
| H | -0.301535239912 | -1.736305254362 | 0.228214229815 |
| H | 0.761122500420 | -1.197715935791 | 1.525488178613 |
| H | 2.145903887641 | -1.593011632263 | -0.324349827436 |
| H | -0.932047773341 | 1.012253576955 | -1.153902407459 |
| H | -0.690803330902 | 0.577994464807 | 3.014379794980 |
| H | 3.047243082015 | 0.565776249656 | -0.765788844621 |
| H | 1.680686814282 | 1.299845664614 | -0.255605700666 |
| H | -2.151211725683 | 2.563211757984 | 2.348411213585 |
| H | 0.572041566700 | -1.959041376271 | -3.272465659539 |

EQ28

| | | | |
|---|---|---|---|
| O | 1.793168610323 | -1.578252233974 | -2.662370050328 |
| O | -0.092525382200 | -0.412518040646 | -2.323730291022 |
| N | -1.209338598367 | 1.086848325485 | -0.116613805081 |
| N | 2.264694444773 | 0.188227244588 | -0.231598064281 |
| N | -1.433141288580 | 2.132359585450 | 1.692555248285 |
| C | 0.129628241882 | -0.996500990112 | 0.563157004969 |
| C | 1.345505414190 | -0.939120543546 | -0.408931749867 |
| C | -0.549561507772 | 0.309933731263 | 0.839306369065 |
| C | 0.911951551303 | -0.933037122121 | -1.876018763552 |
| C | -0.695721422901 | 0.994994395684 | 2.006709024001 |
| C | -1.775215709845 | 2.236947037227 | 0.365887011249 |
| H | -0.591805355887 | -1.735095681706 | 0.186050047304 |
| H | 0.496622538278 | -1.386985215796 | 1.519574346659 |
| H | 1.931803687224 | -1.853648977004 | -0.273016754640 |
| H | -1.222449911706 | 0.821603795177 | -1.094182185501 |
| H | -0.357550906653 | 0.759789198517 | 3.005394333017 |
| H | 1.727824049021 | 1.051930883906 | -0.157608455570 |
| H | 2.765125802912 | 0.083407338330 | 0.648813151466 |
| H | -1.704941786722 | 2.832680013746 | 2.365526998166 |
| H | 1.469124071167 | -1.482132919103 | -3.578573026059 |

EQ29

| | | | |
|---|---|---|---|
| O | -0.042721045706 | -1.827595450276 | -1.777227287195 |
| O | 0.867606424670 | -0.004337238331 | -2.739121187806 |
| N | -0.342598043579 | 1.593789390212 | 0.598484084238 |
| N | 2.189498246654 | 0.723880384923 | -0.484512639446 |
| N | -2.502922454117 | 1.324198127689 | 1.046915781197 |
| C | 0.614481927667 | -0.734289605952 | 0.771967698313 |
| C | 1.521691080459 | -0.585107168533 | -0.474447716899 |
| C | -0.519344806932 | 0.241403081342 | 0.823293316903 |
| C | 0.752240717552 | -0.738547825703 | -1.781843018625 |
| C | -1.861098256984 | 0.106932569034 | 1.101980862391 |
| C | -1.560926553978 | 2.190764388291 | 0.732620193301 |
| H | 0.211073998083 | -1.750481847405 | 0.801829953317 |
| H | 1.265984943362 | -0.627693214479 | 1.652341331993 |
| H | 2.237333047135 | -1.426156329410 | -0.461009975304 |
| H | 0.542617239074 | 1.969917440270 | 0.270061951789 |
| H | -2.398419987910 | -0.801579099899 | 1.342823543556 |
| H | 3.052569595142 | 0.680388045735 | 0.051935215698 |
| H | 2.424792035992 | 0.967134835941 | -1.445570921781 |
| H | -1.704528483779 | 3.253796489007 | 0.590237703568 |
| H | -0.487849839590 | -1.849420759369 | -2.645367607678 |

EQ30

| | | | |
|---|---|---|---|
| O | 0.807628616731 | -1.854266675001 | -2.343321033615 |
| O | 0.565502052830 | 0.379225446679 | -2.312502138129 |
| N | -1.103696585490 | 1.458889158797 | -0.208264863686 |
| N | 2.743917625608 | -1.174565433172 | -0.248737192905 |

| | | | |
|---|---|---|---|
| N | -2.323405658953 | 1.409556325980 | 1.648679448822 |
| C | 1.136043246504 | 0.516177165065 | 0.524771941908 |
| C | 1.327043646658 | -0.782329145575 | -0.280060275695 |
| C | -0.282929122629 | 0.925030131161 | 0.769347930723 |
| C | 0.836171388609 | -0.651324001931 | -1.723879138159 |
| C | -1.065253588023 | 0.911349442085 | 1.902679130963 |
| C | -2.310110990899 | 1.725452805858 | 0.370291801473 |
| H | 1.625654028964 | 0.354930602141 | 1.489516925649 |
| H | 1.685566239981 | 1.322470017464 | 0.019108822631 |
| H | 0.754461320024 | -1.588145384471 | 0.192214624723 |
| H | -0.833086876904 | 1.561450211767 | -1.178456652392 |
| H | -0.783080772028 | 0.575984022630 | 2.892724270798 |
| H | 2.875797211239 | -2.055505311995 | -0.742220306998 |
| H | 3.309423023116 | -0.476139175057 | -0.731063994681 |
| H | -3.137141989406 | 2.148538611968 | -0.184766636300 |
| H | 0.543596052820 | -1.686512342081 | -3.268129820486 |

EQ31

| | | | |
|---|---|---|---|
| O | 1.258983315555 | 0.415806949340 | -2.210989091149 |
| O | 0.065681278544 | -1.484831279729 | -2.423613355789 |
| N | -1.454082785973 | 0.880585303211 | -0.294962281774 |
| N | 1.971191276688 | -0.062688126148 | 0.391805104942 |
| N | -0.414995604776 | 2.330728419033 | 1.197440009306 |
| C | -0.250054272154 | -1.171292893276 | 0.557721420095 |
| C | 1.050213276821 | -0.990698906327 | -0.257375830480 |
| C | -1.032545073832 | 0.116186814465 | 0.880598256443 |
| C | 0.715432007644 | -0.731243983598 | -1.735077274891 |
| C | -0.360025451676 | 1.159123706805 | 1.738086424796 |
| C | -1.087236460983 | 2.085523289249 | -0.038603696763 |
| H | -0.910826181568 | -1.846690620427 | 0.006877408372 |
| H | 0.014340619439 | -1.650652378677 | 1.506503502547 |
| H | 1.529311054074 | -1.977833564560 | -0.263175887930 |
| H | -1.949382393086 | -0.201685097921 | 1.405748355324 |
| H | 0.095544491184 | 0.986682204031 | 2.709053288546 |
| H | 2.909903358419 | -0.189744920605 | 0.018801486680 |
| H | 1.718000406340 | 0.898151274286 | 0.172240130383 |
| H | -1.266567357274 | 2.918288169906 | -0.712900450380 |
| H | 0.966023263900 | 0.481634495621 | -3.139646313583 |

EQ32

| | | | |
|---|---|---|---|
| O | 1.175605222506 | 0.457891699512 | -2.163444704713 |
| O | -0.722830647275 | -0.844818654589 | -1.972847711109 |
| N | -0.337175750754 | 0.707853358888 | -0.327131752905 |
| N | 2.485402552415 | -0.695550352174 | -0.041152250631 |
| N | -1.420314509703 | 2.209685392336 | 0.882046119499 |
| C | 0.288736841211 | -1.140097769549 | 0.991218546445 |
| C | 1.124750103107 | -1.130487724959 | -0.319503415779 |
| C | -0.378244527697 | 0.206030253388 | 0.958499125226 |
| C | 0.308338692494 | -0.183503266347 | -1.274220729076 |
| C | -1.051175392067 | 1.149461944044 | 1.692222482441 |
| C | -0.985946544348 | 1.911937542103 | -0.324666251633 |
| H | -0.450747779047 | -1.950077570380 | 0.973231218274 |
| H | 0.939060697576 | -1.299170308719 | 1.855799392706 |
| H | 1.173951187850 | -2.124336379500 | -0.781094964606 |
| H | -0.299420424115 | -1.520851391772 | -2.528962883197 |
| H | -1.290597057329 | 1.144910938778 | 2.746841192575 |
| H | 2.968745272178 | -0.514544242314 | -0.919692708611 |
| H | 2.461663374298 | 0.198145433703 | 0.450773802328 |
| H | -1.099240973788 | 2.527294030682 | -1.206734036314 |
| H | 0.623296243757 | 0.889043374333 | -2.837571897921 |

EQ33

| | | | |
|---|---|---|---|
| O | 1.170525731280 | 0.495135287215 | -2.155645616814 |
| O | -0.630206653353 | -0.841418826443 | -2.080964285417 |
| N | -0.343380227972 | 0.715756413232 | -0.318830253959 |
| N | 2.493231109775 | -0.685689528540 | -0.050892725711 |
| N | -1.446685339965 | 2.197542273176 | 0.899751614119 |
| C | 0.307006276425 | -1.132485559327 | 0.996955501584 |
| C | 1.129707961013 | -1.119918137672 | -0.322860336207 |
| C | -0.386040947497 | 0.201933747787 | 0.963895048736 |
| C | 0.322229763303 | -0.167259106858 | -1.281421766518 |
| C | -1.074902799753 | 1.131843671418 | 1.701322071813 |
| C | -0.997418945400 | 1.918275672164 | -0.306599104663 |
| H | -0.411172284283 | -1.963289205763 | 1.007515514188 |
| H | 0.970431029887 | -1.267500364757 | 1.855707338702 |
| H | 1.171584247214 | -2.104258223708 | -0.798844911997 |
| H | -1.431236898517 | -0.983208412785 | -1.552227285820 |
| H | -1.322141448027 | 1.114442516943 | 2.754093129893 |
| H | 2.972999838730 | -0.526845429936 | -0.935816093842 |
| H | 2.476402243001 | 0.219618836335 | 0.420253620004 |
| H | -1.098962225127 | 2.544309169329 | -1.182350532560 |
| H | 0.663009150998 | 0.642034613549 | -2.971597605789 |

EQ34

| | | | |
|---|---|---|---|
| O | 1.049545492419 | -2.508589940602 | -1.598286218483 |
| O | 1.828121453700 | -0.893528749792 | -2.966757168972 |
| N | -0.566007316057 | 1.832177117396 | 0.839471300213 |
| N | 1.532033327822 | 1.070681610508 | -0.985710535300 |
| N | -2.187835189288 | 1.399633170354 | 2.296506020556 |
| C | -0.610955400197 | -0.147963569631 | -0.726190172598 |
| C | 0.935895191858 | -0.256582137719 | -0.795547366536 |
| C | -1.098964299608 | 0.613561703397 | 0.465647271162 |
| C | 1.335131276517 | -1.224191382608 | -1.910004254203 |
| C | -2.100238455868 | 0.375106801434 | 1.380261317226 |
| C | -1.245910808469 | 2.251293098291 | 1.944835022860 |
| H | -0.952803610287 | 0.319061479284 | -1.663254111170 |
| H | -1.041412186990 | -1.153654138646 | -0.702030885046 |
| H | 1.290363657434 | -0.668529366082 | 0.155430907890 |
| H | 0.261090761813 | 2.216164871123 | 0.391351415441 |
| H | -2.766284593188 | -0.477286675387 | 1.424933036011 |
| H | 2.538254554421 | 1.013120823910 | -0.831988654856 |
| H | 1.431537372293 | 1.321258656462 | -1.970829674752 |
| H | -1.014030444070 | 3.179247990633 | 2.450925680496 |
| H | 1.304906523110 | -3.047300471253 | -2.371497908177 |

EQ35

| | | | |
|---|---|---|---|
| O | 0.710791491384 | 0.242950468158 | -2.753765182795 |
| O | 0.248231717191 | -1.793607236635 | -1.906782889370 |
| N | -0.563574156124 | 1.486806559803 | 0.584193533315 |
| N | 2.010509133228 | 1.116788503868 | -0.631815511289 |
| N | -2.565124576779 | 0.829229598573 | 1.292916330584 |
| C | 0.846672185995 | -0.607077867719 | 0.718815131790 |
| C | 1.615157176278 | -0.300908574581 | -0.588827510337 |
| C | -0.444280034684 | 0.138205354608 | 0.866338422449 |
| C | 0.773186759637 | -0.710657834157 | -1.793352736570 |
| C | -1.694146986967 | -0.237364123739 | 1.305402891803 |
| C | -1.853320610508 | 1.843847926289 | 0.845156813461 |
| H | 0.641852436729 | -1.680678832204 | 0.752265375764 |
| H | 1.529714130649 | -0.387415764721 | 1.553946567883 |
| H | 2.483636395995 | -0.981836843983 | -0.622116417457 |
| H | 0.187818507064 | 2.010484552369 | 0.143224939203 |
| H | -2.012898576047 | -1.219763323504 | 1.629530662657 |
| H | 2.781852771284 | 1.265203487267 | 0.017008218720 |
| H | 2.346461450614 | 1.361228603221 | -1.560631814754 |
| H | -2.218482142331 | 2.850851037757 | 0.691149619834 |
| H | 0.167523442648 | -0.133339935763 | -3.472341498155 |

EQ36

| | | | |
|---|---|---|---|
| O | 0.175759422134 | -2.050102615163 | -1.797828353682 |
| O | 0.464344471468 | 0.062252400221 | -2.533299527135 |
| N | -0.319847696675 | 1.696051644320 | 0.870662970739 |
| N | 2.386355968159 | 0.552517308462 | -0.670616655896 |
| N | -2.510371026900 | 1.344315249478 | 0.776501364436 |
| C | 0.654058307339 | -0.655804221124 | 0.791403815056 |
| C | 1.563008326432 | -0.632132093932 | -0.481898283360 |
| C | -0.477934713542 | 0.320172898113 | 0.800593619670 |
| C | 0.699007923301 | -0.805061618237 | -1.722890661618 |
| C | -1.841497108629 | 0.141021095788 | 0.745488020280 |
| C | -1.567479950188 | 2.256586842265 | 0.848019785607 |
| H | 0.234163537728 | -1.661489043583 | 0.898156611351 |
| H | 1.314742333240 | -0.502858575021 | 1.657768400372 |
| H | 2.216727020736 | -1.511501667835 | -0.410711550272 |
| H | 0.560852057288 | 2.188195813215 | 0.896652667636 |
| H | -2.376520840944 | -0.798384455443 | 0.691193635871 |
| H | 2.773882901796 | 0.888011169344 | 0.206687953879 |
| H | 1.852258366930 | 1.288744534481 | -1.125189043503 |
| H | -1.720692984141 | 3.326985506936 | 0.887772774721 |
| H | -0.403685981755 | -2.055781212927 | -2.583215009301 |

EQ37

| | | | |
|---|---|---|---|
| O | 2.471207308862 | -1.300598049311 | -2.247968930955 |
| O | 0.357102293664 | -2.028951491998 | -2.520567802718 |
| N | -0.349399923060 | 1.580616686456 | 1.201174199360 |
| N | 1.854039973775 | 0.510464848838 | -0.328454900423 |
| N | -2.423384126754 | 1.595208668434 | 1.999901162410 |
| C | -0.532851418348 | 0.255037368797 | -0.942096486775 |
| C | 0.818243848360 | -0.457573191121 | -0.713468446252 |
| C | -1.108330275243 | 0.854308126507 | 0.302728335615 |
| C | 1.153455163627 | -1.341754997897 | -1.922534947933 |
| C | -2.383772656547 | 0.884810926740 | 0.820428161559 |
| C | -1.182743761782 | 1.990553251908 | 2.200171133319 |
| H | -0.382178235911 | 1.022494333681 | -1.719053710231 |
| H | -1.240709134514 | -0.466776536388 | -1.357060004595 |
| H | 0.692270422800 | -1.152594915787 | 0.127980337629 |
| H | 0.660439059389 | 1.655103695287 | 1.118267159861 |
| H | -3.276685167469 | 0.435432476295 | 0.404738231792 |
| H | 2.719226924914 | 0.020799658741 | -0.106791270879 |
| H | 2.073996622581 | 1.104345421426 | -1.128345981074 |

| | | | |
|---|---|---|---|
| H | -0.833898334904 | 2.569793535696 | 3.045074767622 |
| H | 2.580958519854 | -1.914127851568 | -2.999521242803 |

EQ38

| | | | |
|---|---|---|---|
| O | 1.008319735687 | -2.473957753209 | -1.828884213385 |
| O | 1.507188880363 | -0.653708778043 | -3.065553330085 |
| N | -0.337280851231 | 1.733845842865 | 1.103666651704 |
| N | 1.636998971755 | 0.977660823362 | -0.792526382198 |
| N | -2.518344995655 | 1.631687232991 | 1.691021746328 |
| C | -0.448601347967 | -0.310373344162 | -0.328958192988 |
| C | 1.053954478911 | -0.368005618991 | -0.694043659885 |
| C | -0.727331771329 | 0.319962737296 | 1.038891057914 |
| C | 1.228852602293 | -1.152081622138 | -1.996410499696 |
| C | -2.211799668251 | 0.318120546875 | 1.443273724072 |
| C | -1.429198501270 | 2.389948119811 | 1.455436018179 |
| H | -0.989510811310 | 0.249743447670 | -1.103353267460 |
| H | -0.862653834537 | -1.322350416877 | -0.317330086894 |
| H | 1.576670545130 | -0.915242528515 | 0.099265897026 |
| H | 0.530785963943 | 2.083415104451 | 0.701783286518 |
| H | -0.189108526970 | -0.234586710685 | 1.823778036807 |
| H | 2.653076919551 | 0.898898286409 | -0.827236799351 |
| H | 1.376635372109 | 1.360410128983 | -1.703581978128 |
| H | -1.425821110059 | 3.475434064364 | 1.545178588339 |
| H | 1.097745807838 | -2.888858862505 | -2.708236326178 |

EQ39

| | | | |
|---|---|---|---|
| O | 1.397026375428 | -2.465031867707 | -1.782001298749 |
| O | 1.907007506406 | -0.497750807041 | -2.750367389527 |
| N | 0.347269271749 | 2.320805932133 | 2.001470710424 |
| N | 0.521476733417 | 0.954377899732 | -0.830637232633 |
| N | -1.837427714830 | 1.905408343777 | 1.239110415659 |
| C | -0.945772160246 | -1.024885500099 | -0.818717756512 |
| C | 0.512368566818 | -0.495381588886 | -0.753428979766 |
| C | -1.783060119306 | -0.461682705128 | 0.311669826421 |
| C | 1.349139667491 | -1.113626591211 | -1.867918585556 |
| C | -1.732907137868 | 0.772316673360 | 0.758974438211 |
| C | -0.914771651965 | 2.466395717932 | 2.128871250298 |
| H | -1.371980509437 | -0.745142999034 | -1.791693470795 |
| H | -0.956696305013 | -2.117532297964 | -0.757797043106 |
| H | 0.939777385442 | -0.845158395010 | 0.196207236611 |
| H | 0.831636697752 | 2.870111168930 | 2.715919650967 |
| H | -2.458107198139 | -1.132901589357 | 0.839237616547 |
| H | 0.909488414902 | 1.387396554415 | 0.004294648226 |
| H | 1.026634479159 | 1.263763863132 | -1.657107746024 |
| H | -1.397306066180 | 3.094917163475 | 2.884090631130 |
| H | 1.935893464397 | -2.766306162201 | -2.537969116437 |

EQ40

| | | | |
|---|---|---|---|
| O | 1.616663458275 | -2.258789312045 | -1.237692163108 |
| O | 1.779968199992 | -1.029671588781 | -3.116922845760 |
| N | -0.459313092033 | 0.769302488030 | 1.890499024986 |
| N | 0.332725524325 | 1.023598232729 | -2.055374475685 |
| N | -2.048231081302 | 2.288819924687 | 1.580016787079 |
| C | -0.412563943465 | -0.520945679614 | -0.289973726009 |
| C | 0.780151309088 | -0.017985574301 | -1.146016877464 |
| C | -0.956892387886 | 0.538021207602 | 0.616724547197 |
| C | 1.422093026085 | -1.130879570032 | -1.965119283563 |
| C | -1.932033096503 | 1.497165753874 | 0.457465943095 |
| C | -1.152221709786 | 1.822914115318 | 2.420862433506 |
| H | -1.200295965120 | -0.843310676342 | -0.978034127717 |
| H | -0.099790204819 | -1.397073804977 | 0.288379599893 |
| H | 1.577458285709 | 0.301271407979 | -0.443431028833 |
| H | 0.256593645222 | 0.227779662585 | 2.354014921821 |
| H | -2.561094719685 | 1.651937550165 | -0.409203555543 |
| H | 0.155187280489 | 1.882020083130 | -1.541201119117 |
| H | 1.042289917273 | 1.200757800131 | -2.761667934456 |
| H | -0.961144103986 | 2.197403178282 | 3.417988041914 |
| H | 2.066396274989 | -2.887619778532 | -1.833338060424 |

EQ41

| | | | |
|---|---|---|---|
| O | 2.510354645012 | -1.506713484235 | -2.025996087700 |
| O | 0.445106021712 | -1.557157975034 | -2.920671296591 |
| N | -0.515158746891 | 1.925208651668 | 0.837030925363 |
| N | 1.744421751259 | 0.301505841041 | -0.168600836718 |
| N | -2.230447764177 | 1.510704767839 | 2.185488626441 |
| C | -0.627762032843 | 0.085213296898 | -0.888843543194 |
| C | 0.727812404757 | -0.629055825664 | -0.688618669939 |
| C | -1.122298036771 | 0.784869141164 | 0.336218418893 |
| C | 1.174728425203 | -1.258462268600 | -2.003889484136 |
| C | -2.177628937140 | 0.558778070987 | 1.190396826596 |
| C | -1.216512265396 | 2.313505942333 | 1.943347233027 |
| H | -0.513208925834 | 0.798070899240 | -1.715151300403 |
| H | -1.369525701435 | -0.650935243448 | -1.209987939751 |
| H | 0.565627459655 | -1.485333081534 | -0.008939200956 |

| | | | |
|---|---|---|---|
| H | 0.327893620027 | 2.332063849174 | 0.453926522828 |
| H | -2.910281039232 | -0.236225123634 | 1.135261663993 |
| H | 1.668143421799 | 0.332943453406 | 0.845798766090 |
| H | 2.673605130011 | -0.055970333664 | -0.378415884681 |
| H | -0.944760445162 | 3.183970873885 | 2.526024399645 |
| H | 2.694953198809 | -1.927666883891 | -2.886914302079 |

EQ42

| | | | |
|---|---|---|---|
| O | 0.937854066879 | -2.082691029714 | -2.487679324814 |
| O | 2.241029758551 | -0.279220894322 | -2.854842162752 |
| N | -0.604703685537 | 2.110799008272 | 0.783484544997 |
| N | 2.087294531628 | 0.349436291718 | -0.166214783866 |
| N | -2.035969961848 | 1.226373990905 | 2.231963702037 |
| C | -0.347850223161 | 0.310809004403 | -0.985415919865 |
| C | 1.018376399851 | -0.430230655056 | -0.768106310795 |
| C | -0.933764525677 | 0.861654315366 | 0.274064837249 |
| C | 1.497400798758 | -0.904534134093 | -2.130782269647 |
| C | -1.812810142196 | 0.343895472240 | 1.198475113439 |
| C | -1.301446285205 | 2.278657567429 | 1.949410252251 |
| H | -0.189931294408 | 1.113622834553 | -1.719136409692 |
| H | -1.060519530775 | -0.391873672445 | -1.432129487442 |
| H | 0.821649856159 | -1.306254804204 | -0.141196768742 |
| H | -0.019187522162 | 2.797440647594 | 0.329285624791 |
| H | -2.302329214258 | -0.621091216018 | 1.170274381634 |
| H | 1.755054845633 | 0.831069823043 | 0.665220981027 |
| H | 2.434543567531 | 1.033829888366 | -0.835295012582 |
| H | -1.230524742252 | 3.181993391358 | 2.540763813932 |
| H | 1.255995584012 | -2.275677054629 | -3.390408504986 |

EQ43

| | | | |
|---|---|---|---|
| O | 2.409537253761 | -1.311550844569 | -2.423052834907 |
| O | 0.315521437144 | -2.075539889416 | -2.438882552216 |
| N | -0.353638938906 | 1.595056010289 | 1.226635898963 |
| N | 1.898261918225 | 0.457124799018 | -0.282034201434 |
| N | -2.446907799420 | 1.576620217709 | 1.971245852208 |
| C | -0.478821414878 | 0.299767735562 | -0.946093665386 |
| C | 0.839902349694 | -0.469815790382 | -0.706737615005 |
| C | -1.081595449078 | 0.875952851271 | 0.295786553564 |
| C | 1.145344229622 | -1.358979558159 | -1.937909053267 |
| C | -2.369522383304 | 0.886531297613 | 0.781639145313 |
| C | -1.217243998882 | 1.980572239055 | 2.211130426238 |
| H | -0.281525845457 | 1.085175181621 | -1.694376266370 |
| H | -1.192214243410 | -0.389091480104 | -1.404395739601 |
| H | 0.676360372144 | -1.168176266726 | 0.122953454232 |
| H | 0.654236419643 | 1.689022582197 | 1.184300960204 |
| H | -3.246553875337 | 0.435893287496 | 0.334990340939 |
| H | 2.720161780594 | 0.002655684794 | 0.106587181133 |
| H | 2.167612080888 | 1.120017981332 | -1.005934096194 |
| H | -0.895890840852 | 2.549182952775 | 3.073980104770 |
| H | 2.918582272729 | -0.674948031501 | -1.891208075340 |

EQ44

| | | | |
|---|---|---|---|
| O | 0.770018763978 | -2.422746323196 | -1.316155459034 |
| O | 1.901643408841 | -1.020420137390 | -2.672802945913 |
| N | -0.698469773111 | 2.040884802083 | 0.649589419317 |
| N | 1.470081015849 | 1.149181239805 | -0.962535178029 |
| N | -1.491672120494 | 0.820208944715 | 2.388403056704 |
| C | -0.795600578952 | 0.156681716629 | -1.037803115730 |
| C | 0.706112368670 | -0.092865142941 | -0.771512812186 |
| C | -1.498779636651 | 0.949274331890 | 0.076284370551 |
| C | 1.208215145573 | -1.204496799291 | -1.695217778792 |
| C | -1.997391853199 | 0.158225345719 | 1.296730675307 |
| C | -0.732497002926 | 1.844171360902 | 1.961831355015 |
| H | -0.883572291180 | 0.675761099202 | -2.000943164425 |
| H | -1.326565972968 | -0.795870488424 | -1.123148521596 |
| H | 0.827010097875 | -0.433920630846 | 0.262878888390 |
| H | 0.073607454928 | 2.481188147421 | 0.152484734592 |
| H | -2.427572768475 | 1.378627767089 | -0.334434803631 |
| H | 2.406033035885 | 1.029447222895 | -0.575205141146 |
| H | 1.619114060595 | 1.266003146010 | -1.966586534499 |
| H | -0.172601927584 | 2.493425674920 | 2.634026434096 |
| H | 1.104802059327 | -3.061342809647 | -1.974462475864 |

EQ45

| | | | |
|---|---|---|---|
| O | 2.332369264485 | -1.622409457940 | -1.438694314355 |
| O | 0.590429767220 | -1.866657780063 | -2.838957546694 |
| N | 0.274186097975 | 2.124059863661 | 2.249021158318 |
| N | 1.283563375102 | 0.744387820727 | -0.606806513458 |
| N | -1.721634288265 | 0.948662558534 | 1.883947022896 |
| C | -0.914327989199 | 0.144878352235 | -1.584961123588 |
| C | 0.374188803414 | -0.360841452168 | -0.922299593206 |
| C | -1.867076989714 | 0.872922717591 | -0.655455114243 |
| C | 1.072742066804 | -1.343825466373 | -1.859291276703 |
| C | -1.766395426408 | 0.931574807436 | 0.648289677757 |

| | | | |
|---|---|---|---|
| C | -0.921922920987 | 1.861688487725 | 2.596698513007 |
| H | -0.632633390736 | 0.819460315447 | -2.401973976487 |
| H | -1.436563643182 | -0.703756612061 | -2.041704094242 |
| H | 0.093682850774 | -0.970042162244 | -0.037882096893 |
| H | 0.709234122546 | 2.757811016274 | 2.924103239312 |
| H | -2.712218285639 | 1.389109403156 | -1.107801475434 |
| H | 0.908411837223 | 1.295793905980 | 0.167138784893 |
| H | 2.166249089072 | 0.360794578381 | -0.275949917804 |
| H | -1.419504644140 | 2.238495526291 | 3.495367326625 |
| H | 2.696753243892 | -2.258850868412 | -2.082143680524 |

EQ46

| | | | |
|---|---|---|---|
| O | 2.499382138914 | -1.059078802900 | -2.461298624592 |
| O | 0.588726358148 | -2.225430259993 | -2.226434044110 |
| N | -0.992693327034 | 0.393320779121 | 1.747206505391 |
| N | 1.261311819000 | 1.151559589015 | -1.506416387619 |
| N | -1.724410287463 | 2.489445037407 | 1.704263507954 |
| C | -0.643571430644 | -0.180892678117 | -0.697851334114 |
| C | 0.875089405805 | -0.151984747750 | -0.970138098514 |
| C | -1.056432318834 | 0.740734442710 | 0.406330945012 |
| C | 1.258693036053 | -1.255318611546 | -1.951923205066 |
| C | -1.507762776238 | 2.042097966735 | 0.418319787302 |
| C | -1.408564859616 | 1.474016564583 | 2.475916351996 |
| H | -1.153148648515 | 0.111574703125 | -1.621221286389 |
| H | -0.942436333869 | -1.212544874046 | -0.483390207726 |
| H | 1.398743950584 | -0.416262915006 | -0.027773040187 |
| H | -0.728616866357 | -0.510860077848 | 2.112424686309 |
| H | -1.700982282233 | 2.675657978079 | -0.437735472838 |
| H | 1.015632965833 | 1.869714628395 | -0.826595986694 |
| H | 2.267937440600 | 1.190166635509 | -1.646744537413 |
| H | -1.461426619886 | 1.456807117093 | 3.556621932820 |
| H | 2.669699995317 | -1.809277237181 | -3.061848196486 |

EQ47

| | | | |
|---|---|---|---|
| O | 2.495216309011 | -1.350537495232 | -2.072631742380 |
| O | 0.576323414135 | -1.500643939083 | -3.235622970704 |
| N | 0.281088027210 | 2.143507992420 | 2.273596660903 |
| N | 1.309206028487 | 0.489319718439 | -0.457677781549 |
| N | -1.912537274809 | 2.076522559585 | 1.422613220872 |
| C | -0.931432533268 | -0.196824702339 | -1.229842675747 |
| C | 0.517324087338 | -0.638643777278 | -0.945290222165 |
| C | -1.762087402826 | -0.049804723847 | 0.032892371555 |
| C | 1.155812008040 | -1.189594580236 | -2.219009424014 |
| C | -1.805024064588 | 1.037680023143 | 0.761543110087 |
| C | -0.952291114383 | 2.431023584211 | 2.395411202920 |
| H | -0.889004854132 | 0.748359599315 | -1.779771574311 |
| H | -1.407886575696 | -0.933469244603 | -1.881573928544 |
| H | 0.483308073984 | -1.500829602348 | -0.247228705308 |
| H | 0.814578098366 | 2.533166346134 | 3.054764760442 |
| H | -2.317469024162 | -0.912662490109 | 0.396095047603 |
| H | 0.927294495814 | 0.848793408437 | 0.419579109503 |
| H | 2.261103863197 | 0.185292238294 | -0.267247401033 |
| H | -1.387988474478 | 3.017428198460 | 3.209239006789 |
| H | 2.817716519317 | -1.710533538727 | -2.920287852112 |

EQ48

| | | | |
|---|---|---|---|
| O | 1.222962292120 | -1.751244006528 | -2.503396477232 |
| O | 0.367903633608 | 0.312693207511 | -2.319211907274 |
| N | -1.186881068537 | 1.408172725500 | -0.187630598531 |
| N | 2.628234775277 | -1.285157987282 | -0.229078060311 |
| N | -2.275172232156 | 1.511735810149 | 1.747569595675 |
| C | 1.097572853377 | 0.509810332081 | 0.465596884728 |
| C | 1.239413295089 | -0.813725786031 | -0.317153826653 |
| C | -0.302035795899 | 0.945792667216 | 0.768355506410 |
| C | 0.892123502517 | -0.649672445463 | -1.794648495301 |
| C | -1.003559744353 | 1.025488287266 | 1.951252832051 |
| C | -2.349320211351 | 1.727143329853 | 0.450077386733 |
| H | 1.609312534668 | 0.369414629165 | 1.424821042711 |
| H | 1.655675843909 | 1.286369298912 | -0.071156275883 |
| H | 0.499920714893 | -1.533849946434 | 0.081781749072 |
| H | -0.978035380912 | 1.429063324856 | -1.178497882058 |
| H | -0.653863468197 | 0.766359880136 | 2.942880286981 |
| H | 2.842002057385 | -1.505170329044 | 0.742098039811 |
| H | 2.734979841384 | -2.147912862359 | -0.758335945751 |
| H | -3.212425573791 | 2.110981591735 | -0.077929948500 |
| H | 0.976165849088 | -1.572133224210 | -3.430764596581 |

EQ49

| | | | |
|---|---|---|---|
| O | 0.547643709776 | -1.824530180420 | -2.241549191253 |
| O | 0.711841960401 | 0.415526257808 | -2.293486257554 |
| N | -1.066295820936 | 1.465217576923 | -0.218041884602 |
| N | 2.793858330432 | -1.166119154828 | -0.400302818683 |
| N | -2.352001706302 | 1.335438854516 | 1.589743049780 |
| C | 1.155831966926 | 0.521437861170 | 0.553140359626 |

| | | | |
|---|---|---|---|
| C | 1.385922801863 | -0.770597613774 | -0.267918418142 |
| C | -0.272837446709 | 0.911226280933 | 0.771589750829 |
| C | 0.842826519891 | -0.635265445690 | -1.691427200368 |
| C | -1.096587554430 | 0.846660907716 | 1.873399994046 |
| C | -2.297102586856 | 1.695292486932 | 0.324372805372 |
| H | 1.624113247791 | 0.369371315751 | 1.532482625255 |
| H | 1.704170132594 | 1.336173519814 | 0.060823962649 |
| H | 0.854706785234 | -1.604325446690 | 0.202334656305 |
| H | -0.764087956587 | 1.610790141572 | -1.173213899542 |
| H | -0.846801142078 | 0.481623762469 | 2.861733782318 |
| H | 3.147505047469 | -1.483156268317 | 0.499980952937 |
| H | 3.353592386042 | -0.357299672114 | -0.668850626241 |
| H | -3.109330487088 | 2.126304621272 | -0.246126303002 |
| H | 0.260569353167 | -1.645205735608 | -3.157421742114 |

EQ50

| | | | |
|---|---|---|---|
| O | -0.138041058996 | -0.473744703832 | -2.298579471116 |
| O | 2.052756406348 | -0.768858867741 | -2.701200486207 |
| N | -1.516538889266 | 0.871347082129 | -0.108907724891 |
| N | 2.668137718948 | -1.082092146421 | 0.017658801129 |
| N | -2.271472656610 | 1.422951465094 | 1.907601096225 |
| C | 1.006296091241 | 0.730630349728 | 0.242668595635 |
| C | 1.325985055156 | -0.668998621694 | -0.375294279155 |
| C | -0.399847393833 | 0.951358796297 | 0.704631419628 |
| C | 1.156498527675 | -0.650712035097 | -1.898218367367 |
| C | -0.900772140101 | 1.297067511446 | 1.939665979743 |
| C | -2.606555130890 | 1.153175727861 | 0.664234167422 |
| H | 1.655184451466 | 0.814409525296 | 1.119310954278 |
| H | 1.323396212928 | 1.518584203470 | -0.456917355187 |
| H | 0.611711693909 | -1.393648484117 | 0.032142762106 |
| H | -1.501045880251 | 0.567103562683 | -1.073293966205 |
| H | -0.341159949416 | 1.466152248161 | 2.850806187257 |
| H | 2.856952468346 | -2.013042849009 | -0.352611023967 |
| H | 3.346791308555 | -0.476442735748 | -0.445226509463 |
| H | -3.613505619208 | 1.145584493399 | 0.267735507651 |
| H | -0.119442385454 | -0.467541937379 | -3.275206944340 |

EQ51

| | | | |
|---|---|---|---|
| O | 2.010201795353 | -0.852045575789 | -2.567310859475 |
| O | -0.142268362473 | -0.269791975736 | -2.362308348893 |
| N | -1.314226208236 | 1.111069267917 | -0.145419952635 |
| N | 2.222707001083 | -1.826932288180 | -0.145607148590 |
| N | -2.080418904858 | 1.886522576692 | 1.793571773682 |
| C | 1.062922747773 | 0.388119231194 | 0.422805591735 |
| C | 1.061703828420 | -0.971405646003 | -0.364589108796 |
| C | -0.281648177072 | 0.956383332253 | 0.761232745741 |
| C | 0.884682723376 | -0.680249745991 | -1.850996626841 |
| C | -0.785975957196 | 1.445437451251 | 1.946310214563 |
| C | -2.365077311587 | 1.662684557851 | 0.527211754411 |
| H | 1.582933570140 | 0.200904742900 | 1.369108829909 |
| H | 1.685339084922 | 1.113107082734 | -0.124537607199 |
| H | 0.171748852012 | -1.532244461815 | -0.058301155307 |
| H | -1.283688940015 | 0.773629093967 | -1.103210218022 |
| H | -0.279852017799 | 1.507701509405 | 2.901649691559 |
| H | 2.311388907294 | -2.025824666619 | 0.848878491016 |
| H | 3.076382083689 | -1.354886800691 | -0.437086216845 |
| H | -3.310314411294 | 1.875477724791 | 0.045340132223 |
| H | 1.793786608942 | -0.596965866894 | -3.484755199427 |

EQ52

| | | | |
|---|---|---|---|
| O | 0.864832871558 | -1.444209352483 | -2.638535337684 |
| O | 0.860351286177 | 0.757983710480 | -2.161966343324 |
| N | -1.490628929506 | 0.374530811972 | -0.031437421826 |
| N | 2.593141766648 | -1.284631584362 | -0.301299974888 |
| N | -1.891292437009 | 2.196082952075 | 1.354306411727 |
| C | 0.918109737894 | 0.244795542072 | 0.656987365232 |
| C | 1.177712631728 | -0.875825310598 | -0.358164070911 |
| C | -0.558895859453 | 0.315179507527 | 1.096390390170 |
| C | 0.931845011605 | -0.387369179768 | -1.787914283507 |
| C | -0.926679201573 | 1.536559629140 | 1.898302554273 |
| C | -2.185214594576 | 1.432785916320 | 0.180931580517 |
| H | 1.534972118591 | 0.075401302596 | 1.547794195919 |
| H | 1.227370832256 | 1.196396640661 | 0.216465892094 |
| H | 0.476516179089 | -1.708736888304 | -0.181968461786 |
| H | -0.794901972171 | -0.592713671490 | 1.675678522249 |
| H | -0.459483111494 | 1.854631864760 | 2.827697350038 |
| H | 2.780427393270 | -1.710531709545 | 0.605357172650 |
| H | 2.761151098605 | -2.008513347871 | -0.998692961246 |
| H | -2.977613638712 | 1.770294906382 | -0.480635170972 |
| H | 0.754571380631 | -1.067097483900 | -3.531842076335 |

EQ53

| | | | |
|---|---|---|---|
| O | 2.591878398672 | -1.403515424449 | -1.870465810618 |
| O | 1.282238823428 | -0.252445576460 | -3.292536215261 |

| | | | |
|---|---|---|---|
| N | -1.841507861347 | 1.306996563538 | 0.298626959908 |
| N | 0.817792299803 | -1.548982313463 | 0.025778690408 |
| N | -1.599886973923 | 1.471714257723 | 2.500911547540 |
| C | 0.511081357858 | 0.851783572504 | -0.531304739828 |
| C | 0.462962025440 | -0.610797101791 | -1.047609598572 |
| C | -0.487732855545 | 1.138778579556 | 0.542118237286 |
| C | 1.474707657532 | -0.742641172892 | -2.204137698364 |
| C | -0.375775595908 | 1.241368865377 | 1.911696915247 |
| C | -2.455398984050 | 1.508944562725 | 1.504455157087 |
| H | 1.515200129310 | 1.046400452229 | -0.136487094188 |
| H | 0.376366597649 | 1.511154053296 | -1.396171300989 |
| H | -0.531115742440 | -0.797657742772 | -1.475789342226 |
| H | -2.283569718588 | 1.323152952880 | -0.610034736610 |
| H | 0.529800598640 | 1.183327369722 | 2.502594635101 |
| H | 0.513925178463 | -1.187081002981 | 0.929586724088 |
| H | 0.354141303222 | -2.445608107234 | -0.105721520549 |
| H | -3.520664031333 | 1.679102822413 | 1.588836747081 |
| H | 2.445024400808 | -1.684633277513 | -0.930462792903 |

EQ54

| | | | |
|---|---|---|---|
| O | 2.679769983684 | -1.105444861980 | -1.778495859825 |
| O | 1.153060111131 | -0.543018245600 | -3.338321202840 |
| N | -1.786838160986 | 1.434718939519 | 0.281090999619 |
| N | 0.699374699200 | -1.666867888849 | -0.014450553662 |
| N | -1.632603254518 | 1.424565387332 | 2.496690474014 |
| C | 0.552438878751 | 0.844700516501 | -0.490683989437 |
| C | 0.466522021380 | -0.632127948902 | -1.013168775700 |
| C | -0.461809810512 | 1.141279708737 | 0.564177391852 |
| C | 1.428891681766 | -0.770751589044 | -2.182174502161 |
| C | -0.405347291904 | 1.136454007697 | 1.940583683685 |
| C | -2.437604593313 | 1.599166253581 | 1.472793002218 |
| H | 1.555079902421 | 1.007906735296 | -0.078988886700 |
| H | 0.440050255838 | 1.531427768918 | -1.340939130041 |
| H | -0.531890899847 | -0.782266565694 | -1.437527917224 |
| H | -2.188043969268 | 1.536814894549 | -0.640691745985 |
| H | 0.463553611416 | 0.958094445085 | 2.561219578290 |
| H | 1.662327115880 | -1.618435220108 | 0.314067836623 |
| H | 0.099085953524 | -1.492169509567 | 0.790959252307 |
| H | -3.489494281676 | 1.846920413835 | 1.527305240224 |
| H | 3.227325927939 | -1.139746073634 | -2.585509896124 |

EQ55

| | | | |
|---|---|---|---|
| O | 1.414254966008 | -1.971312927496 | -2.272426857520 |
| O | 2.553090507442 | -0.068289107655 | -2.632437368845 |
| N | -2.774651080561 | -0.080386845132 | 0.896451333086 |
| N | -0.033731824123 | -1.046485735554 | -0.340951094853 |
| N | -1.358879544907 | 1.332395488084 | 2.115231447196 |
| C | 1.370439955465 | 1.091689055385 | -0.277659500433 |
| C | 0.660686466697 | -0.000164319385 | -1.101653679187 |
| C | 0.421817240364 | 2.038425137771 | 0.440985236064 |
| C | 1.660978582944 | -0.669751442172 | -2.078098337202 |
| C | -0.516751688988 | 1.656943031478 | 1.272387044501 |
| C | -2.624632926591 | 0.820799168859 | 1.784198268823 |
| H | 2.051496011521 | 0.609287657114 | 0.438007695480 |
| H | 2.000569046591 | 1.671252470938 | -0.955793541759 |
| H | -0.091332304449 | 0.483354071313 | -1.738798011325 |
| H | -3.751989667465 | -0.376376663969 | 0.846177781479 |
| H | 0.489154520414 | 3.103847691982 | 0.231967874075 |
| H | 0.476015995090 | -1.277461550094 | 0.510989774125 |
| H | -0.984602192353 | -0.779841984326 | -0.070240640073 |
| H | -3.419680128029 | 1.221721149229 | 2.419198067354 |
| H | 0.658395066998 | -2.167148310804 | -1.656030510065 |

EQ56

| | | | |
|---|---|---|---|
| O | 2.333010880395 | -1.544190335487 | -1.837507291418 |
| O | 0.870303804719 | -0.470235205066 | -3.169628009429 |
| N | -1.749858865624 | 1.656293293253 | 0.210017013999 |
| N | 1.018752214623 | -1.192055979504 | 0.359478288086 |
| N | -1.541806675715 | 0.611676733166 | 2.201376354253 |
| C | 0.588216495943 | 1.069131592891 | -0.583190037086 |
| C | 0.458020125449 | -0.451011475439 | -0.782142267150 |
| C | -0.310098978861 | 1.645795509926 | 0.525938429030 |
| C | 1.230482968055 | -0.826508335792 | -2.070018567516 |
| C | -0.249156127748 | 0.903784966276 | 1.859084450345 |
| C | -2.353822583748 | 1.065193047378 | 1.230167052493 |
| H | 1.624199513863 | 1.300308704060 | -0.309566426440 |
| H | 0.385715807063 | 1.564834893247 | -1.538013883941 |
| H | -0.595198869249 | -0.696083658166 | -0.976082757974 |
| H | -2.213310965159 | 2.073888493905 | -0.587271479198 |
| H | 0.003302243047 | 2.682080940880 | 0.727527770736 |
| H | 0.991850427842 | -0.612270995315 | 1.212872937369 |
| H | 0.467562144247 | -2.025613601767 | 0.554742667028 |
| H | -3.437353378937 | 0.964104897726 | 1.258326002175 |
| H | 2.339585573591 | -1.659579019992 | -0.846823581274 |

EQ57

| | | | |
|---|---|---|---|
| O | 1.931638684451 | -1.884781952985 | -2.475963579776 |
| O | 2.099063839101 | 0.342263781023 | -2.713730346182 |
| N | -1.482678789865 | 0.235045205790 | 0.841740292796 |
| N | 0.471272117376 | -1.776069522391 | -0.328299355523 |
| N | -1.789736516628 | 2.229275363813 | 2.111473817198 |
| C | 0.662279449072 | 0.771688003397 | -0.273879698637 |
| C | 0.510468643742 | -0.518137614348 | -1.089973551290 |
| C | -0.497807681183 | 1.045002341953 | 0.637068492040 |
| C | 1.606477736613 | -0.620278544288 | -2.171727498362 |
| C | -0.698190538982 | 2.282393093956 | 1.435745892218 |
| C | -2.357184429190 | 0.929146588315 | 1.777224951982 |
| H | 1.587945211317 | 0.734291229027 | 0.321501682183 |
| H | 0.802191685874 | 1.611549987306 | -0.965291423949 |
| H | -0.441183973095 | -0.463182689183 | -1.634812748291 |
| H | -2.485910720401 | 0.333895609158 | 2.690955901650 |
| H | -0.031742872895 | 3.141806128651 | 1.464829680026 |
| H | 1.180499244794 | -1.764061846406 | 0.405538051109 |
| H | -0.430444590411 | -1.842576186225 | 0.141615034845 |
| H | -3.353862997673 | 1.061786448639 | 1.335909340427 |
| H | 1.404765280329 | -2.440818185643 | -1.847176855480 |

EQ58

| | | | |
|---|---|---|---|
| O | 2.824429402433 | -1.112064249139 | -2.260899505847 |
| O | 1.041085453679 | -0.733713100479 | -3.580413544922 |
| N | -1.301604602005 | 1.709498212904 | 0.374197123394 |
| N | 1.467610557293 | -1.365391215567 | -0.064237114112 |
| N | -2.012197915651 | 1.550738864656 | 2.615398543962 |
| C | 0.348669465561 | 0.662663907576 | -0.907920829889 |
| C | 0.687469623629 | -0.822959821594 | -1.186156109464 |
| C | -0.333262891475 | 0.822488284742 | 0.420991165553 |
| C | 1.518633879844 | -0.898455629857 | -2.481438295148 |
| C | -1.951797313402 | 0.841515684335 | 3.615123833526 |
| C | -2.128775450279 | 2.087657581277 | 1.466384930882 |
| H | 1.304926496770 | 1.206295633892 | -0.828207248629 |
| H | -0.186839390939 | 1.074525810152 | -1.782110900462 |
| H | -0.249657475626 | -1.363962619537 | -1.370691641981 |
| H | -1.553616957486 | 2.214293756562 | -0.488921412784 |
| H | -1.272731487031 | 1.158817190204 | 4.417863534411 |
| H | 1.144669287707 | -0.905165879698 | 0.790256151080 |
| H | 1.293681353561 | -2.362951146383 | 0.043494105834 |
| H | -2.862252916565 | 2.862763387212 | 1.281667343241 |
| H | 2.890953750871 | -1.219830657748 | -1.274642870607 |

EQ59

| | | | |
|---|---|---|---|
| O | 2.478480324232 | -1.404956420353 | -0.869233340426 |
| O | 1.988387056752 | 0.501309567577 | -1.948036421158 |
| N | -1.930137484493 | 0.732168907950 | 0.847616741823 |
| N | 0.005825346529 | -2.051117907213 | -0.405294237564 |
| N | -0.438807842568 | 1.991498591063 | 1.903950137242 |
| C | -0.811671505322 | 0.183521996774 | -1.363947720205 |
| C | 0.161056869299 | -1.016603528493 | -1.438741823890 |
| C | -0.850558981173 | 0.846884368600 | -0.018909728383 |
| C | 1.633296999644 | -0.537396814386 | -1.440306725691 |
| C | 0.054078391216 | 1.625092591696 | 0.670543225253 |
| C | -1.633065901468 | 1.447983574915 | 1.974947329977 |
| H | -0.500453149708 | 0.902726360321 | -2.127821705625 |
| H | -1.817171394861 | -0.162090713741 | -1.637233220746 |
| H | 0.021449178176 | -1.508455675610 | -2.410902101982 |
| H | -2.808791484389 | 0.278275930288 | 0.640958769083 |
| H | 1.031661931796 | 1.942293186446 | 0.334077116764 |
| H | -0.200525725441 | -1.615044205494 | 0.493711136893 |
| H | -0.756222333834 | -2.688467825594 | -0.622131724185 |
| H | -2.321996206033 | 1.531903863625 | 2.805191900893 |
| H | 1.900286342034 | -2.121220982753 | -0.504573831396 |

EQ60

| | | | |
|---|---|---|---|
| O | 0.288060321184 | 0.124823465877 | -2.562639261274 |
| O | 0.501957165070 | -2.004434282062 | -1.859812358876 |
| N | -0.492398825047 | 1.573522039963 | 1.183399685244 |
| N | 2.101892291936 | 1.079407924248 | -0.902406587020 |
| N | -2.577677381578 | 0.886369747612 | 0.852585607121 |
| C | 0.896860766662 | -0.506842732463 | 0.727817352642 |
| C | 1.653213573092 | -0.278143856552 | -0.624149927173 |
| C | -0.398522559098 | 0.228434784002 | 0.853850497605 |
| C | 0.778256616797 | -0.830467175706 | -1.744380953224 |
| C | -1.703989222891 | -0.159686683421 | 0.652626095803 |
| C | -1.819096180446 | 1.909745111442 | 1.174083936439 |
| H | 0.703359886137 | -1.579603833508 | 0.828842983433 |
| H | 1.583558192403 | -0.230344024641 | 1.540528957953 |
| H | 2.537970140129 | -0.925494396972 | -0.598583935070 |
| H | 0.277143597092 | 2.176429795342 | 1.436053948242 |
| H | -2.057628920779 | -1.144057175753 | 0.374764705265 |

| | | | |
|---|---|---|---|
| H | 2.670066854139 | 1.428484333617 | -0.133284668559 |
| H | 1.308963971951 | 1.704357694671 | -1.026391983943 |
| H | -2.164165778288 | 2.907835646616 | 1.410022197482 |
| H | -0.300441737360 | -0.334054732950 | -3.191834950606 |

EQ61

| | | | |
|---|---|---|---|
| O | 0.028936790874 | 0.580782159519 | -2.314822543170 |
| O | 1.677758063570 | -0.795526322680 | -2.961136355900 |
| N | -1.549551288295 | 1.060868737292 | 0.062415232556 |
| N | 2.523352607389 | -1.195070996742 | -0.414123009532 |
| N | -2.409212112164 | 0.813568753724 | 2.096339934128 |
| C | 0.911883489204 | 0.484244470033 | 0.400618190136 |
| C | 1.164014709103 | -0.688808105987 | -0.591630365928 |
| C | -0.497281111174 | 0.668701520253 | 0.870197866778 |
| C | 1.019112966948 | -0.320773703390 | -2.064637708739 |
| C | -1.062783351396 | 0.526952020081 | 2.118165992437 |
| C | -2.666702776645 | 1.127011779171 | 0.844429786048 |
| H | 1.520062129982 | 0.269394645159 | 1.285114156090 |
| H | 1.324164059708 | 1.407752978692 | -0.025384678237 |
| H | 0.378128233316 | -1.448731345782 | -0.414321222680 |
| H | -1.475495959432 | 1.213126009434 | -0.933678554432 |
| H | -0.564714551291 | 0.240035344054 | 3.035808586360 |
| H | 2.562784595919 | -1.791807194014 | 0.408683567287 |
| H | 2.785088853151 | -1.757171410698 | -1.221395818030 |
| H | -3.634032797358 | 1.407975727651 | 0.448743208422 |
| H | 0.020659629948 | 0.701707065321 | -3.283818950470 |

EQ62

| | | | |
|---|---|---|---|
| O | 0.587507104414 | -0.119949292564 | -2.917923704016 |
| O | 0.540380039211 | -2.059041924709 | -1.816030627374 |
| N | -0.474979569217 | 1.513239681364 | 1.364102911584 |
| N | 1.802174692257 | 1.235643645150 | -0.755482129472 |
| N | -2.586739275864 | 0.980378448206 | 0.931822217730 |
| C | 0.830157383920 | -0.541033046658 | 0.665769681394 |
| C | 1.569264344687 | -0.202583931372 | -0.653742729318 |
| C | -0.443671223872 | 0.220967916194 | 0.857203539879 |
| C | 0.841913981230 | -0.890786201235 | -1.836923376626 |
| C | -1.763282783623 | -0.072525316960 | 0.596865318734 |
| C | -1.782813601110 | 1.912879767552 | 1.392573645515 |
| H | 0.615076298999 | -1.612195101851 | 0.660358249471 |
| H | 1.518114261875 | -0.342598716294 | 1.495992052452 |
| H | 2.552821870856 | -0.686497722174 | -0.608183696942 |
| H | 0.331087798348 | 2.036282230572 | 1.677221014542 |
| H | -2.161207557597 | -0.991535294442 | 0.187085804092 |
| H | 2.486261653957 | 1.501949538058 | -1.456544033042 |
| H | 0.948980932457 | 1.779114210255 | -0.852866844483 |
| H | -2.083126515657 | 2.885754582615 | 1.759175958891 |
| H | 0.888252134597 | 0.784549387349 | -2.716722723204 |

EQ63

| | | | |
|---|---|---|---|
| O | 0.391027716511 | 0.293616840453 | -2.535138190557 |
| O | 0.678778516556 | -1.894039312869 | -2.136263932528 |
| N | -0.543716017701 | 1.591787817830 | 0.588738887796 |
| N | 2.140807522246 | 1.045277074046 | -0.684793771336 |
| N | -2.606180911977 | 0.890480049607 | 1.026668929667 |
| C | 0.830731562638 | -0.553495018509 | 0.692568842727 |
| C | 1.688559067926 | -0.358126537383 | -0.580341060960 |
| C | -0.452342441881 | 0.216778206961 | 0.749113715117 |
| C | 0.875477667336 | -0.744879497611 | -1.827813314782 |
| C | -1.740744047113 | -0.180266699936 | 1.023622810122 |
| C | -1.856832750292 | 1.935212803159 | 0.753201409040 |
| H | 0.590621678375 | -1.618358625590 | 0.764692769978 |
| H | 1.470018829704 | -0.319906146958 | 1.559408316957 |
| H | 2.521622303382 | -1.071920084618 | -0.529703637996 |
| H | 0.213753718925 | 2.187087068828 | 0.282698018706 |
| H | -2.089450652764 | -1.185542172437 | 1.221659810960 |
| H | 2.566530026782 | 1.334036511494 | 0.196193300501 |
| H | 2.876444172325 | 1.123372925331 | -1.387324547160 |
| H | -2.202806402850 | 2.956318621760 | 0.659915778405 |
| H | 0.660181923346 | 1.108710611417 | -2.060633227280 |

TS0

| | | | |
|---|---|---|---|
| O | 0.506110642628 | -0.270754485362 | -2.700392070002 |
| O | 2.305597204323 | 0.094086575007 | -1.443233469607 |
| N | -1.610182369067 | 0.883626293359 | 1.245038339822 |
| N | 1.305212865505 | -2.432229168771 | -0.218832497452 |
| N | -1.170960222398 | 2.926014088001 | 0.493568183105 |
| C | 0.565328033552 | -0.372390049965 | 0.881881149807 |
| C | 0.534046959218 | -1.205202167290 | -0.419446295304 |
| C | -0.285340742552 | 0.857939970492 | 0.832975068729 |
| C | 1.210503883585 | -0.393379012372 | -1.545225990901 |
| C | -0.050675658931 | 2.133222849204 | 0.369884361508 |
| C | -2.085545213787 | 2.147743224956 | 1.026861888383 |
| H | 0.256509401234 | -1.019931458238 | 1.714462713434 |

| | | | |
|---|---|---|---|
| H | 1.610807166346 | -0.103637606752 | 1.056889187084 |
| H | -0.525589570759 | -1.385210352991 | -0.692135755844 |
| H | -2.109662614754 | 0.124357769620 | 1.685946934777 |
| H | 0.870240161266 | 2.517293113638 | -0.048239476530 |
| H | 0.844857712652 | -3.017711858172 | 0.475783963838 |
| H | 1.360860380057 | -2.977759019158 | -1.076909391803 |
| H | -3.097247786797 | 2.436738766224 | 1.279922973472 |
| H | -0.380052504471 | -0.652710516851 | -2.591059482326 |

TS1

| | | | |
|---|---|---|---|
| O | 1.145129333177 | -0.944287493275 | -2.861672138669 |
| O | 2.008561305216 | 0.532687087092 | -1.439266789357 |
| N | -1.533621086131 | 0.957161551279 | 1.488381422571 |
| N | 0.962512950040 | -2.485684736213 | -0.357187536465 |
| N | -1.229241834044 | 2.973288049452 | 0.613411682922 |
| C | 0.530009448812 | -0.338216330956 | 0.812674618177 |
| C | 0.456089063698 | -1.110033331766 | -0.522694128196 |
| C | -0.301334713011 | 0.908810715762 | 0.851035404277 |
| C | 1.271412296477 | -0.401359142393 | -1.616411246704 |
| C | -0.149281475095 | 2.169371305784 | 0.317361577610 |
| C | -2.038350650058 | 2.216684341248 | 1.320666745566 |
| H | 0.208795708631 | -1.029602121813 | 1.601096898921 |
| H | 1.581359030211 | -0.107346625289 | 1.007596452835 |
| H | -0.593473564298 | -1.104958264103 | -0.864068269424 |
| H | -1.949146441986 | 0.212406180928 | 2.029616431983 |
| H | 0.684133737096 | 2.533159723027 | -0.266595948244 |
| H | 0.206886094579 | -3.155702745807 | -0.252709865933 |
| H | 1.553391269125 | -2.791057871162 | -1.124481805245 |
| H | -2.990506845926 | 2.518742979345 | 1.737074022801 |
| H | 0.480730545881 | -1.653902619138 | -2.846573708141 |

TS2

| | | | |
|---|---|---|---|
| O | 1.130340268416 | -1.034442374558 | -2.839183672305 |
| O | 1.963506039404 | 0.564880244545 | -1.468899816859 |
| N | -1.578404833831 | 0.927098145441 | 1.399036701680 |
| N | 1.055300308139 | -2.431213038502 | -0.364232786049 |
| N | -1.199863198732 | 2.991566971192 | 0.678482125171 |
| C | 0.544622429055 | -0.328645518998 | 0.829060604893 |
| C | 0.461723773395 | -1.094139189968 | -0.505820346707 |
| C | -0.293963615298 | 0.912812685264 | 0.873118798657 |
| C | 1.246108732730 | -0.385336917710 | -1.612500010541 |
| C | -0.096366432718 | 2.202545954351 | 0.433593710210 |
| C | -2.068432295288 | 2.196426316706 | 1.262020505305 |
| H | 0.234310957660 | -1.013434065374 | 1.630367608641 |
| H | 1.594156150930 | -0.085714538669 | 1.015526829346 |
| H | -0.594926590784 | -1.116914621535 | -0.838248218679 |
| H | -2.042876459985 | 0.151742319108 | 1.849993287929 |
| H | 0.785846907791 | 2.597770469209 | -0.050027010812 |
| H | 0.477737228526 | -2.996128838484 | 0.256121515047 |
| H | 1.060655669721 | -2.894847778117 | -1.271024924158 |
| H | -3.054943624138 | 2.476213181535 | 1.607996799136 |
| H | 0.351899945538 | -0.703899155146 | -3.316784274308 |

TS3

| | | | |
|---|---|---|---|
| O | 0.856805588932 | -0.889120626408 | -2.912976302191 |
| O | 1.948670363669 | 0.476525038499 | -1.534671493247 |
| N | -1.567482247272 | 0.925004442142 | 1.379567504280 |
| N | 0.910227906858 | -2.508729978522 | -0.432492398608 |
| N | -1.144598999652 | 3.001122557885 | 0.717452162957 |
| C | 0.541773353312 | -0.346341860092 | 0.791829157654 |
| C | 0.398456439055 | -1.118607064425 | -0.533142822189 |
| C | -0.279336946423 | 0.903606445248 | 0.863762225374 |
| C | 1.132035866397 | -0.398341782650 | -1.672857049911 |
| C | -0.053858367820 | 2.199842044450 | 0.459739154798 |
| C | -2.032938684398 | 2.205998634423 | 1.270935815248 |
| H | 0.254510461092 | -1.026076586205 | 1.606414227797 |
| H | 1.599389859368 | -0.099263039714 | 0.928771036840 |
| H | -0.671902103026 | -1.160412258047 | -0.791283346918 |
| H | -2.054846781846 | 0.143486531385 | 1.794564583648 |
| H | 0.837739298426 | 2.589292977728 | -0.011597181784 |
| H | 1.353792724799 | -2.645571802024 | 0.473170139565 |
| H | 1.631102064932 | -2.692011424879 | -1.127326672715 |
| H | -3.017806022263 | 2.494230892664 | 1.614519853149 |
| H | 0.153163563131 | -1.558520325896 | -2.858166612202 |

TS4

| | | | |
|---|---|---|---|
| O | 1.763517340289 | -2.138211806411 | -2.319396017320 |
| O | 2.976650202116 | -0.926162012327 | -0.915488962048 |
| N | -1.207693599252 | 0.656790446169 | 1.669733185704 |
| N | -0.493312132751 | -1.357206034539 | -0.686922753903 |
| N | -1.956695039950 | 2.697279278383 | 1.217721688593 |
| C | 0.891987982145 | 0.447707302459 | 0.269452737093 |
| C | 0.606228313110 | -0.418624680057 | -0.976354756614 |
| C | -0.300131297657 | 1.201259223799 | 0.770496221176 |

| | | | |
|---|---|---|---|
| C | 1.888270076117 | -1.174054202304 | -1.368241225229 |
| C | -0.798156179243 | 2.458202261620 | 0.511390866285 |
| C | -2.171072404281 | 1.598536623495 | 1.906721560473 |
| H | 1.291155006786 | -0.206429468739 | 1.052835000094 |
| H | 1.692618228913 | 1.147852943718 | 0.019071242308 |
| H | 0.376302458643 | 0.262264263523 | -1.818043172723 |
| H | -1.126866588684 | -0.253201875924 | 2.102708393097 |
| H | -0.376662518847 | 3.210153105519 | -0.143327340023 |
| H | -1.298279442998 | -0.806331472213 | -0.389206894733 |
| H | -0.810881536404 | -1.835438940895 | -1.529922209327 |
| H | -2.994748687206 | 1.425763788130 | 2.586812914726 |
| H | 0.840487732272 | -2.239773951920 | -2.599162543780 |

TS5

| | | | |
|---|---|---|---|
| O | 0.749452712152 | -0.670232589686 | -2.836074714538 |
| O | 2.044231507779 | 0.471226948009 | -1.433890361869 |
| N | -1.591591251329 | 0.570053039890 | 1.513772082172 |
| N | 1.298409953616 | -2.421708896321 | -0.352550712469 |
| N | -1.029968046862 | 2.869378186858 | 0.531635963366 |
| C | 0.613657110796 | -0.421619352036 | 0.894199985302 |
| C | 0.533003742167 | -1.169150896955 | -0.449101894144 |
| C | -0.404106294522 | 0.701532946735 | 1.014268818079 |
| C | 1.178371665041 | -0.350717473755 | -1.584287052810 |
| C | -0.049804899378 | 2.008116344996 | 0.484565219641 |
| C | -2.237778449535 | 2.898058964397 | 0.740700639009 |
| H | 0.440756716255 | -1.145836806692 | 1.699223470664 |
| H | 1.633920116626 | -0.042374334962 | 1.002696760274 |
| H | -0.535840528865 | -1.322140925864 | -0.697420761407 |
| H | -1.781818634503 | -0.392626070912 | 1.802487712522 |
| H | 0.904313500621 | 2.253776808648 | 0.041587553596 |
| H | 0.848874816290 | -3.047083534036 | 0.313920646970 |
| H | 1.318130475678 | -2.908341815265 | -1.247295756060 |
| H | -2.705590332835 | 3.344971803430 | 1.617677141314 |
| H | -0.018575085374 | -1.263545128573 | -2.787079063534 |

TS6

| | | | |
|---|---|---|---|
| O | 0.127900843325 | 0.209645095804 | -2.171640029096 |
| O | 2.023495332920 | -0.925434189108 | -2.381802047031 |
| N | -1.907254060813 | 0.964967176647 | 0.854692065449 |
| N | 2.005452276269 | -1.915679072239 | 0.113484846261 |
| N | -0.772992180936 | 2.861428301984 | 0.657605032311 |
| C | -0.143626384651 | -0.847286688907 | 0.711657546554 |
| C | 0.726734300491 | -1.472283728354 | -0.440115939611 |
| C | -0.584581206458 | 0.590451220308 | 0.646287344829 |
| C | 1.042245478331 | -0.667856692957 | -1.723453612312 |
| C | 0.085165912044 | 1.794175838591 | 0.532773927333 |
| C | -1.959016661967 | 2.329006634309 | 0.857250807622 |
| H | -1.040488639407 | -1.465743435110 | 0.838384787623 |
| H | 0.456860861795 | -0.983340259545 | 1.617934662673 |
| H | 0.127398323303 | -2.308004366771 | -0.851834412746 |
| H | -2.682048704830 | 0.334213558022 | 1.006921558271 |
| H | 1.143803636039 | 1.942201823173 | 0.363800271461 |
| H | 1.886546979458 | -2.797255391900 | 0.607807101931 |
| H | 2.643489926759 | -2.080457855095 | -0.663885697596 |
| H | -2.883560696754 | 2.873600411516 | 0.995873822396 |
| H | -0.433192974815 | 0.512101856422 | -1.429749770655 |

TS7

| | | | |
|---|---|---|---|
| O | 1.386259764226 | -0.020889297398 | -2.414748712390 |
| O | -0.590043528370 | -1.002191206299 | -2.153911021634 |
| N | -0.869056967995 | 0.765122417291 | -0.332004289062 |
| N | 2.317772886840 | -0.265295449243 | -0.095845443205 |
| N | -1.216581970326 | 2.353832849517 | 1.254668426866 |
| C | 0.114627268317 | -1.113710594087 | 0.849761286474 |
| C | 1.125035795327 | -1.102930343170 | -0.310392073743 |
| C | -0.491880154738 | 0.252336911211 | 0.895331437218 |
| C | 0.532651111132 | -0.582661800965 | -1.634014288193 |
| C | -0.695704449049 | 1.222087407526 | 1.851554710986 |
| C | -1.285157125850 | 2.032420649137 | -0.030462105858 |
| H | -0.629277321909 | -1.903934154527 | 0.665215427818 |
| H | 0.635642356310 | -1.383208336206 | 1.774962676582 |
| H | 1.465428898531 | -2.121730903065 | -0.551975397719 |
| H | -1.242089801551 | -0.558371462076 | -1.514495968130 |
| H | -0.516487297265 | 1.180093618459 | 2.919999471572 |
| H | 2.053225532566 | 0.535376662294 | 0.483887392121 |
| H | 3.056044401666 | -0.775883775693 | 0.380946024534 |
| H | -1.655113765290 | 2.707886922981 | -0.793908393858 |
| H | 2.187226812614 | 0.118244632894 | -1.795105873047 |

TS8

| | | | |
|---|---|---|---|
| O | 1.297326844669 | 0.272211770036 | -2.215422103463 |
| O | -0.083031285154 | -1.519668329293 | -2.298255034167 |
| N | -1.381734170267 | 0.857804357249 | -0.217924799701 |
| N | 2.421250341661 | -0.515905189045 | 0.068177006036 |

| | | | |
|---|---|---|---|
| N | -1.055165126858 | 2.471043034778 | 1.274397404883 |
| C | -0.015118146661 | -1.078051222102 | 0.673552444616 |
| C | 1.210907489632 | -1.242138256377 | -0.288887429871 |
| C | -0.554985996035 | 0.312972394149 | 0.752480046083 |
| C | 0.762896034142 | -0.893002049122 | -1.708622287924 |
| C | -0.372667365456 | 1.336746221201 | 1.655212705032 |
| C | -1.646058485589 | 2.148390843208 | 0.144894926059 |
| H | -0.794699775697 | -1.780888241479 | 0.353863118982 |
| H | 0.306303993230 | -1.390873386147 | 1.673563526057 |
| H | 1.450371714748 | -2.312672040374 | -0.307342919689 |
| H | -1.703481717380 | 0.385292818057 | -1.052246408728 |
| H | 0.203015152448 | 1.309863928316 | 2.571844134334 |
| H | 2.211000688323 | 0.478820385270 | 0.140784055015 |
| H | 2.739546700365 | -0.819630096699 | 0.987282847238 |
| H | -2.275953590308 | 2.797315035842 | -0.449016866849 |
| H | 2.050979959360 | 0.080558198505 | -2.796631610681 |

TS9

| | | | |
|---|---|---|---|
| O | 1.986875333045 | -0.059313935740 | -2.323635353938 |
| O | -0.104771707187 | -0.875490183727 | -2.424691064759 |
| N | -1.991398126048 | 0.652514840439 | 0.715263805517 |
| N | 2.369896023077 | -0.628248288602 | 0.192042582120 |
| N | -0.974424219992 | 2.611300206201 | 0.921883739553 |
| C | -0.151541274906 | -1.037379285595 | 0.462281236298 |
| C | 1.133016131275 | -1.204349888120 | -0.369466834414 |
| C | -0.638315415390 | 0.376997504769 | 0.624102217963 |
| C | 0.920151095386 | -0.684454156734 | -1.812286098847 |
| C | -0.040060404699 | 1.612207288465 | 0.760596243132 |
| C | -2.136217004303 | 2.000130046956 | 0.886085809068 |
| H | -0.929152619536 | -1.617948047405 | -0.045908320931 |
| H | 0.007323465062 | -1.510471696131 | 1.442660531384 |
| H | 1.308095668716 | -2.281810167665 | -0.482252198684 |
| H | -2.735294588243 | -0.021312896288 | 0.599878026735 |
| H | 1.012301980086 | 1.863315118081 | 0.734442829800 |
| H | 2.170512719999 | 0.170063272686 | 0.788846073210 |
| H | 2.869577410278 | -1.307148668645 | 0.759840678004 |
| H | -3.105670159498 | 2.471930667257 | 0.975232256096 |
| H | 2.650523852775 | -0.050330332501 | -1.588055693418 |

TS10

| | | | |
|---|---|---|---|
| O | 1.866135402079 | -0.931526058969 | -2.728152375986 |
| O | -0.277743093140 | -0.624485840884 | -2.252199795717 |
| N | -1.339448431741 | 1.002737728557 | -0.153482198660 |
| N | 2.286685304020 | 0.000587220988 | -0.001274085887 |
| N | -1.360101878062 | 2.323282970304 | 1.635245486458 |
| C | 0.051830231590 | -0.984800655965 | 0.592500484846 |
| C | 1.256635928904 | -0.994440088122 | -0.375932563147 |
| C | -0.592770114521 | 0.348435665148 | 0.808120333592 |
| C | 0.841752611897 | -0.833524691592 | -1.842272126358 |
| C | -0.625829236439 | 1.189669700109 | 1.900675106943 |
| C | -1.769578154146 | 2.175932063383 | 0.391742406016 |
| H | -0.676488500834 | -1.724034730569 | 0.237806549786 |
| H | 0.415909647915 | -1.340391003974 | 1.562650258249 |
| H | 1.705071992523 | -2.000406080508 | -0.303854288386 |
| H | -1.458065646453 | 0.657318281211 | -1.099174395706 |
| H | -0.175664037107 | 1.025848497284 | 2.872064237123 |
| H | 2.556410132259 | 0.572408482995 | -0.799375681931 |
| H | 1.874795697019 | 0.652490613688 | 0.667116298515 |
| H | -2.376683348084 | 2.881627860930 | -0.159950113041 |
| H | 2.688538702441 | -1.173210775499 | -2.266890984206 |

TS11

| | | | |
|---|---|---|---|
| O | 1.780432983985 | 0.321298253627 | -2.157539088128 |
| O | -0.255880797804 | -0.614845196797 | -2.192431751354 |
| N | -1.518258228729 | 0.665948136059 | -0.005192575698 |
| N | 2.674817481939 | -1.150853311020 | -0.225538923520 |
| N | -1.495207376462 | 2.600860871280 | 1.094792652071 |
| C | 0.548598700465 | -0.521899055514 | 0.927738560538 |
| C | 1.204255222889 | -1.171643068936 | -0.344027562185 |
| C | -0.373010031295 | 0.650503754860 | 0.773288798819 |
| C | 0.823574066931 | -0.460960503027 | -1.658345898990 |
| C | -0.398583243293 | 1.850140631054 | 1.448992363650 |
| C | -2.132394573218 | 1.866047455966 | 0.207467408472 |
| H | 0.039561205054 | -1.326133012681 | 1.475139454851 |
| H | 1.359465649073 | -0.177584663504 | 1.579988777727 |
| H | 0.808083822055 | -2.189243512755 | -0.447739847936 |
| H | -1.692449589818 | 0.023181618804 | -0.769790823120 |
| H | 0.311561072869 | 2.205198424797 | 2.185619559988 |
| H | 2.974733549063 | -1.231821684825 | 0.742562433499 |
| H | 3.092437102682 | -1.928591781773 | -0.733983611802 |
| H | -3.036177011104 | 2.149630667989 | -0.315495365080 |
| H | 2.545493654842 | 0.216364321442 | -1.534710170691 |

TS12

| | | | |
|---|---|---|---|
| O | 1.970737851806 | -1.768554641958 | -2.470361587828 |
| O | 0.776479324498 | 0.084614723529 | -2.899331993619 |
| N | -1.619485961962 | 1.315773386226 | 0.038594116956 |
| N | 1.835133369171 | -1.558532157769 | 0.118030301717 |
| N | -2.029106990100 | 1.525187383664 | 2.211338019323 |
| C | 0.841674935236 | 0.814158796387 | -0.092570272242 |
| C | 0.914911845459 | -0.658665339816 | -0.597067726891 |
| C | -0.401457454675 | 1.132878187282 | 0.672650550092 |
| C | 1.222209098117 | -0.719570233631 | -2.111683180545 |
| C | -0.690246603646 | 1.264477285807 | 2.010871403053 |
| C | -2.555994006406 | 1.551051238701 | 1.007340901277 |
| H | 1.711458134954 | 1.016461252218 | 0.543236190846 |
| H | 0.921272989091 | 1.476106793866 | -0.961988412858 |
| H | -0.083096720199 | -1.105297203728 | -0.509650915180 |
| H | -1.763478813218 | 1.312647291317 | -0.962601654611 |
| H | -0.002463966633 | 1.194515279123 | 2.844100235352 |
| H | 2.675520094210 | -1.058980911020 | 0.406437641264 |
| H | 1.405967360219 | -1.929244040203 | 0.962342280852 |
| H | -3.596273171023 | 1.735412414970 | 0.773779032781 |
| H | 2.185878971331 | -2.227996348485 | -1.618763713965 |

TS13

| | | | |
|---|---|---|---|
| O | 0.931193187670 | -1.818114087137 | -2.449907629528 |
| O | 0.513923620114 | 0.356433553750 | -2.309016784243 |
| N | -1.156393607920 | 1.416117646529 | -0.219074015806 |
| N | 2.717803452121 | -1.225308891626 | -0.229401327215 |
| N | -2.319740211991 | 1.437118461453 | 1.674786040797 |
| C | 1.117589033186 | 0.545541481068 | 0.491393747601 |
| C | 1.304820498366 | -0.771442614829 | -0.285234804366 |
| C | -0.299127641852 | 0.944619056413 | 0.758519639008 |
| C | 0.860200975223 | -0.657755913107 | -1.747585315970 |
| C | -1.046905089625 | 0.972729387425 | 1.914836884949 |
| C | -2.349044194828 | 1.690206573290 | 0.382200784167 |
| H | 1.617150896426 | 0.417191855985 | 1.458725625942 |
| H | 1.652812592337 | 1.338369997970 | -0.048832632106 |
| H | 0.672351300657 | -1.536736963938 | 0.191782530806 |
| H | -0.914578669859 | 1.474828007186 | -1.200978134888 |
| H | -0.730146291325 | 0.690211364642 | 2.911020979730 |
| H | 3.076934292975 | -1.453929767529 | -1.154038370076 |
| H | 3.303117521315 | -0.467798964834 | 0.116712859288 |
| H | -3.199571758811 | 2.071266184459 | -0.167570852858 |
| H | 1.152099020295 | -2.557958814600 | -1.857268810528 |

TS14

| | | | |
|---|---|---|---|
| O | 1.594783645430 | -1.610705987241 | -2.596950435398 |
| O | 0.125627527656 | 0.052213345381 | -2.345637923272 |
| N | -1.298801120166 | 1.221903990208 | -0.181748039795 |
| N | 2.458891632542 | -1.566473924831 | -0.170460467975 |
| N | -2.133342097481 | 1.784668260065 | 1.774206439225 |
| C | 1.069783811641 | 0.491525355963 | 0.406123211323 |
| C | 1.172269270409 | -0.866500594434 | -0.338757783538 |
| C | -0.318150157345 | 0.943881032704 | 0.751248145334 |
| C | 0.903864886223 | -0.741673259023 | -1.858561549928 |
| C | -0.871823532186 | 1.227959699707 | 1.968650911389 |
| C | -2.412741262409 | 1.776148052166 | 0.394241233964 |
| H | 1.625023177527 | 0.390362540079 | 1.345189250586 |
| H | 1.582699351456 | 1.261855796744 | -0.185877811392 |
| H | 0.394337773543 | -1.538138002817 | 0.046416725028 |
| H | -1.163198398817 | 1.044445945038 | -1.173191389960 |
| H | -0.432132297255 | 1.130602136315 | 2.952580048555 |
| H | 3.233860965755 | -0.903576404318 | -0.172961496402 |
| H | 2.495165290832 | -2.066991479107 | 0.714071214592 |
| H | -3.121625895937 | 1.179077663924 | 1.329973077395 |
| H | 2.185438246738 | -2.080601817882 | -1.952903408576 |

TS15

| | | | |
|---|---|---|---|
| O | 1.851291394027 | -0.896591375057 | -2.931973088080 |
| O | 0.100696324620 | 0.352535797440 | -2.334572716417 |
| N | -0.970125266342 | 0.950615452914 | 0.185173244643 |
| N | 2.692506219676 | -1.425697338404 | -0.579201421287 |
| N | -2.559245031281 | 1.571362183641 | 1.773496742765 |
| C | 1.350816901998 | 0.495422362855 | 0.419471953515 |
| C | 1.394511492133 | -0.715618871813 | -0.566702850617 |
| C | 0.042941583559 | 0.977068631974 | 1.025906808426 |
| C | 1.046108769631 | -0.352800052479 | -2.025566685674 |
| C | -2.364199123747 | 1.509227817026 | 2.993623495813 |
| C | -2.294360081438 | 1.319609813303 | 0.553028076423 |
| H | 2.007863022410 | 0.220310110560 | 1.254286399761 |
| H | 1.828525227740 | 1.372314973355 | -0.048838756472 |
| H | 0.629097179146 | -1.436444632238 | -0.250276629704 |
| H | -0.868066787364 | 0.710871180460 | -0.815988130715 |
| H | -2.128395414269 | 2.456324289247 | 3.499626255049 |
| H | 3.447091299951 | -0.793998689373 | -0.314179867282 |
| H | 2.702422913668 | -2.194821357723 | 0.085550457489 |

| | | | |
|---|---|---|---|
| H | -3.041700892898 | 1.362452551836 | -0.227821907363 |
| H | 2.509085074916 | -1.416220623478 | -2.391140756554 |

TS16

| | | | |
|---|---|---|---|
| O | 1.278636876536 | -1.584003114398 | -2.441451803111 |
| O | 0.342147331833 | 0.444594499189 | -2.395097365952 |
| N | -1.103491595397 | 1.535183268257 | -0.255952282525 |
| N | 2.023159779503 | -1.589473537580 | 0.026314933756 |
| N | -1.882113405936 | 0.570722368953 | 1.910230077464 |
| C | 1.251983545419 | 0.837761927564 | 0.331397444031 |
| C | 1.007038779305 | -0.563488279077 | -0.271216830108 |
| C | -0.028846107029 | 1.535001247185 | 0.827505785940 |
| C | 0.840075247350 | -0.498418506621 | -1.808246617219 |
| C | -0.690534314877 | 0.896673170591 | 1.991816095254 |
| C | -2.246398310799 | 1.089541105225 | 0.107515818101 |
| H | 1.900084789082 | 0.741546298857 | 1.207431413184 |
| H | 1.768364096044 | 1.472499566609 | -0.397373489132 |
| H | 0.056627620999 | -0.949454070580 | 0.120625014758 |
| H | -0.794093310151 | 1.701975728259 | -1.215984040700 |
| H | 0.202545162574 | 2.585106294221 | 1.048380671626 |
| H | 2.956607399415 | -1.180488046373 | 0.051771083016 |
| H | 1.855956834162 | -2.010666921317 | 0.937024496536 |
| H | -3.015769381538 | 1.033495559527 | -0.672682336448 |
| H | 1.685503658273 | -2.142342028528 | -1.726093245883 |

TS17

| | | | |
|---|---|---|---|
| O | 1.910181494236 | -1.139218863367 | -2.697257032594 |
| O | -0.209054156498 | -0.480933459484 | -2.275862173832 |
| N | -1.381331867423 | 0.997406625737 | -0.130029084675 |
| N | 2.267772140809 | 0.054246300252 | -0.113856452603 |
| N | -1.382886320482 | 2.308800608818 | 1.665101708452 |
| C | 0.038785805250 | -0.985128458449 | 0.581777250662 |
| C | 1.253072162787 | -0.977269410403 | -0.380877787024 |
| C | -0.610254463677 | 0.344692401425 | 0.814545121551 |
| C | 0.873505652714 | -0.817903838108 | -1.854742049830 |
| C | -0.632264113552 | 1.181299357051 | 1.910626138901 |
| C | -1.812158413090 | 2.164364759149 | 0.427929016067 |
| H | -0.694565913234 | -1.718008171782 | 0.219413781232 |
| H | 0.393369993709 | -1.353550252850 | 1.551425391063 |
| H | 1.752375325207 | -1.951572728130 | -0.320017654047 |
| H | -1.492499958721 | 0.668188204759 | -1.081990074062 |
| H | -0.162890442977 | 1.017980572261 | 2.872920643798 |
| H | 1.800724365407 | 0.890656138683 | 0.238002786535 |
| H | 2.898445550595 | -0.256506575474 | 0.621114329017 |
| H | -2.433732931608 | 2.867929789576 | -0.110245219899 |
| H | 2.389925620830 | -0.334560185470 | -2.950592860674 |

TS18

| | | | |
|---|---|---|---|
| O | 0.826325970325 | -2.003077649523 | -2.318770007171 |
| O | 0.608793186589 | 0.226928913568 | -2.503191223029 |
| N | -1.055259165309 | 1.097869509479 | -0.168322588682 |
| N | 2.295048635366 | 0.498456480082 | -0.198576605584 |
| N | -1.755617552896 | 1.954749343584 | 1.761879938942 |
| C | 0.344559566088 | -0.908812956002 | 0.576898904285 |
| C | 1.516761906572 | -0.712610364678 | -0.439385397099 |
| C | -0.501278204798 | 0.303915915227 | 0.824545185841 |
| C | 0.950799106684 | -0.745791854361 | -1.853027180105 |
| C | -0.956341593512 | 0.859853987954 | 1.999987042570 |
| C | -1.781817148056 | 2.073529803308 | 0.451618682621 |
| H | -0.256968594194 | -1.766898944128 | 0.246089235322 |
| H | 0.787146486803 | -1.199773781843 | 1.535875402814 |
| H | 2.155659664801 | -1.600762139954 | -0.348552882615 |
| H | -0.820392224850 | 1.047600666865 | -1.153407917746 |
| H | -0.757672181014 | 0.517818466757 | 3.007970872352 |
| H | 2.229211298936 | 1.117905722745 | -1.003321581571 |
| H | 1.899578233386 | 1.013013381130 | 0.585683894994 |
| H | -2.303588910315 | 2.842543007653 | -0.102894671466 |
| H | 0.417215549464 | -1.937361856228 | -3.203029686126 |

TS19

| | | | |
|---|---|---|---|
| O | 2.850296894150 | -0.184473158674 | -1.855798513303 |
| O | 0.762750146705 | -0.449549471970 | -2.655168066864 |
| N | -1.835566365127 | 0.633517716432 | 1.112346760635 |
| N | 2.060337751878 | -0.484155705040 | 0.675214346612 |
| N | -1.287479614100 | 2.631587274528 | 0.322592130252 |
| C | -0.273916704764 | -0.972056872187 | -0.030138665340 |
| C | 1.227285612545 | -1.097554114704 | -0.354356250147 |
| C | -0.777829312831 | 0.415877336487 | 0.243241230025 |
| C | 1.544370573689 | -0.526251142977 | -1.734845190394 |
| C | -0.466654118136 | 1.670461988553 | -0.226787425310 |
| C | -2.099857019908 | 1.975378482351 | 1.119609582945 |
| H | -0.823165077786 | -1.424846410515 | -0.865415490168 |
| H | -0.470198612885 | -1.604435263840 | 0.847751381069 |
| H | 1.415376711040 | -2.183920706877 | -0.483517798106 |

| | | | |
|---|---|---|---|
| H | -2.326413637700 | -0.076753349540 | 1.636388150482 |
| H | 0.312871769078 | 1.940946282458 | -0.924314298170 |
| H | 1.926075560029 | -0.971564256211 | 1.558697150711 |
| H | 3.042650463220 | -0.567364389057 | 0.426531409693 |
| H | -2.886995578034 | 2.407126382980 | 1.723656384145 |
| H | 2.965655206015 | 0.127319831207 | -2.773343703157 |

TS20

| | | | |
|---|---|---|---|
| O | 1.061968234405 | 0.458947514958 | -2.014135014859 |
| O | 0.565626554490 | -1.688639403825 | -2.478101022934 |
| N | -1.575636124195 | 0.742529704568 | -0.144419156674 |
| N | 2.390748322444 | -0.298265163560 | 0.213940360023 |
| N | -0.947304038461 | 2.499780927383 | 1.252581352870 |
| C | -0.029686501774 | -1.061610353463 | 0.632424134785 |
| C | 1.279630291189 | -1.132645617528 | -0.227723170494 |
| C | -0.628680108785 | 0.307037319725 | 0.731845757735 |
| C | 0.943059157810 | -0.855823529769 | -1.687637284698 |
| C | -0.299119893993 | 1.369805965420 | 1.587389205533 |
| C | -1.700866246524 | 2.137138154105 | 0.197150132655 |
| H | -0.764971442607 | -1.758346492797 | 0.215388019073 |
| H | 0.228184631892 | -1.422195623951 | 1.636333786886 |
| H | 1.610786993180 | -2.175579625680 | -0.207274814049 |
| H | -1.085427786930 | 1.700886705827 | -0.857496504758 |
| H | 0.394610562902 | 1.352052674352 | 2.421684307257 |
| H | 2.130010334652 | 0.685556260547 | 0.190821184262 |
| H | 2.633240844200 | -0.526706901460 | 1.176362379765 |
| H | -2.538615535367 | 2.713433352367 | -0.179529825631 |
| H | 0.815322156672 | 0.521014658298 | -2.956661091331 |

TS21

| | | | |
|---|---|---|---|
| O | 1.795528829948 | -1.559907970553 | -2.658817471681 |
| O | -0.119676442709 | -0.443302843617 | -2.320903269189 |
| N | -1.204699591578 | 1.048757328010 | -0.121127328754 |
| N | 2.230195141100 | 0.236395253679 | -0.244696954773 |
| N | -1.346170001840 | 2.194215316972 | 1.750465677400 |
| C | 0.130431458484 | -1.008559788834 | 0.566543857375 |
| C | 1.341702277511 | -0.915158076136 | -0.409263811976 |
| C | -0.581370685656 | 0.281199959176 | 0.846740699708 |
| C | 0.901795849200 | -0.933401629016 | -1.875160399724 |
| C | -0.736465629535 | 0.970405067375 | 2.017393107851 |
| C | -1.646049235951 | 2.250280939161 | 0.376918597805 |
| H | -0.577297094827 | -1.757873887979 | 0.185727038803 |

| | | | |
|---|---|---|---|
| H | 0.504619183212 | -1.393245362132 | 1.521747910586 |
| H | 1.953920715428 | -1.811554403593 | -0.268376759652 |
| H | -1.224947715392 | 0.774182929675 | -1.098505665142 |
| H | -0.416609736890 | 0.693185470920 | 3.013122025576 |
| H | 1.684821521237 | 1.096537055215 | -0.209863371202 |
| H | 2.725555936096 | 0.167238341957 | 0.641684684133 |
| H | -2.516910405982 | 2.341414293827 | 1.358477923002 |
| H | 1.468369040641 | -1.480907187899 | -3.575674204245 |

TS22

| | | | |
|---|---|---|---|
| O | 0.047251065393 | -1.785617308118 | -1.945747012338 |
| O | 0.635018973974 | 0.341533349640 | -2.408945866782 |
| N | -0.534269267082 | 1.570831057255 | 0.225580534987 |
| N | 2.643633361704 | 0.273285694785 | -0.412357688648 |
| N | -2.541067956666 | 1.283203239951 | 1.137260229389 |
| C | 0.668204376017 | -0.620793003230 | 0.831413673870 |
| C | 1.571803131321 | -0.722041145115 | -0.436806694564 |
| C | -0.514446100528 | 0.303611387530 | 0.789661506423 |
| C | 0.723022299149 | -0.644223766976 | -1.704861553501 |
| C | -1.765084533491 | 0.165545780168 | 1.348389673684 |
| C | -1.776199238945 | 2.099297844144 | 0.447413567782 |
| H | 0.277856035440 | -1.617573099318 | 1.060398110239 |
| H | 1.353836225771 | -0.348782215849 | 1.644388945710 |
| H | 2.041711999381 | -1.710221130448 | -0.406591651645 |
| H | 0.158714210381 | 1.952636984173 | -0.401995429489 |
| H | -2.143368841041 | -0.683376735487 | 1.903713769504 |
| H | 3.359446855594 | 0.006378297749 | -1.087337931465 |
| H | 2.287100548461 | 1.163767080973 | -0.758910525760 |
| H | -2.058965307308 | 3.076881593940 | 0.079643784181 |
| H | -0.513485887832 | -1.622321510254 | -2.728569511073 |

TS23

| | | | |
|---|---|---|---|
| O | 1.929739599641 | -0.838778144526 | -2.704242508524 |
| O | -0.265362944612 | -0.560012308998 | -2.262763693387 |
| N | -1.409026367812 | 0.974050658646 | -0.124671533407 |
| N | 2.271717958597 | -0.094537026312 | 0.063254763721 |
| N | -1.319846368108 | 2.341549060462 | 1.625302404792 |
| C | 0.031848886304 | -0.996754907921 | 0.588143834012 |
| C | 1.234813896315 | -1.028854897246 | -0.378659809150 |
| C | -0.592460811809 | 0.349161709016 | 0.802027098347 |
| C | 0.838428426990 | -0.812977251661 | -1.844470562232 |
| C | -0.561015660077 | 1.219366879522 | 1.870635869263 |

| | | | |
|---|---|---|---|
| C | -1.809220984056 | 2.160328307164 | 0.415911326115 |
| H | -0.717519393236 | -1.718250704501 | 0.239568282797 |
| H | 0.397227371825 | -1.353431423933 | 1.555865666923 |
| H | 1.669535714380 | -2.039896563568 | -0.336148244481 |
| H | -1.574587506047 | 0.619467575335 | -1.058349500938 |
| H | -0.039648647475 | 1.086553591469 | 2.810098669423 |
| H | 3.007953127377 | -0.032352034945 | -0.636781210707 |
| H | 1.865869124759 | 0.834470255585 | 0.171826623885 |
| H | -2.454592997328 | 2.848697857687 | -0.113903733740 |
| H | 2.105779498640 | -1.745862168512 | -3.002795972943 |

TS24

| | | | |
|---|---|---|---|
| O | 1.208031134346 | -1.614761213766 | -2.432953681697 |
| O | 0.525034720707 | 0.523529087744 | -2.167356777830 |
| N | -1.636022347746 | 0.745386040507 | -0.090625941588 |
| N | 2.096439522618 | 0.526873470986 | 0.254042833334 |
| N | -0.800848134748 | 2.288185448500 | 1.311635165057 |
| C | -0.306731692754 | -1.261123906234 | 0.489130502899 |
| C | 1.742151501233 | -0.577744665865 | -0.395729574010 |
| C | -0.772086510887 | 0.022218014058 | 0.708899417424 |
| C | 1.101015192820 | -0.451925752039 | -1.727193090473 |
| C | -0.155805443681 | 1.039198729023 | 1.549771300806 |
| C | -1.586432751718 | 2.069985607523 | 0.309686789458 |
| H | -0.769277920714 | -1.905176075375 | -0.253200377191 |
| H | 0.195407552820 | -1.770492789171 | 1.304025455447 |
| H | 2.194391386671 | -1.545333898652 | -0.184829154721 |
| H | -2.011013377631 | 0.421606238483 | -0.971073866416 |
| H | 0.139779693974 | 0.827528725575 | 2.576319500067 |
| H | 2.696406844807 | 0.278336009324 | 1.045325408405 |
| H | 0.997506728279 | 1.047994428122 | 0.898075781850 |
| H | -2.193574500602 | 2.818562824950 | -0.186235798594 |
| H | 0.766564051783 | -1.445107246599 | -3.285369708544 |

TS25

| | | | |
|---|---|---|---|
| O | 2.077322087118 | -0.522644539375 | -2.629319210681 |
| O | -0.331514737410 | -0.135120866965 | -3.157887639690 |
| N | -1.269513788000 | 1.076918819588 | -0.084852658116 |
| N | 1.986852109343 | -0.189999499742 | -0.190263842794 |
| N | -1.332469971788 | 2.527121075604 | 1.600463483437 |
| C | -0.115418581217 | -0.945122891196 | 0.904741939571 |
| C | 1.060881008179 | -1.092619208856 | -0.026290205298 |
| C | -0.658211445231 | 0.447085800822 | 0.987426197237 |
| C | 0.299441316074 | -0.684229670641 | -2.331023415573 |
| C | -0.713011201974 | 1.368523776807 | 2.008883790966 |
| C | -1.648816604684 | 2.316819406680 | 0.339845564600 |
| H | -0.883067312637 | -1.660873422246 | 0.586793676081 |
| H | 0.203307399394 | -1.265231976308 | 1.907528738378 |
| H | 1.258731542100 | -2.069373718729 | -0.451618310092 |
| H | -1.348875261205 | 0.702906520559 | -1.023840854412 |
| H | -0.349973878837 | 1.251246845376 | 3.022224154209 |
| H | 2.358212821472 | -0.239859769042 | -1.263213519769 |
| H | 1.771982290646 | 0.728004060027 | 0.199153539998 |
| H | -2.148815473158 | 3.020711190756 | -0.312157317139 |
| H | 2.154498782820 | 0.232559429260 | -3.237807306823 |

TS26

| | | | |
|---|---|---|---|
| O | 1.704443917685 | 0.309543989747 | -2.098300360577 |
| O | -0.304485045115 | -0.699257704316 | -2.199879613893 |
| N | -1.555993043702 | 0.591838307522 | 0.070861100881 |
| N | 2.611414652285 | -1.415067243159 | -0.140372898897 |
| N | -1.373820791571 | 2.610666694130 | 0.985567361663 |
| C | 0.531792039912 | -0.608521553525 | 0.923850722964 |
| C | 1.175511461537 | -1.265609486798 | -0.355461058416 |
| C | -0.356906099453 | 0.586560288845 | 0.766460705747 |
| C | 0.761137322872 | -0.539082410704 | -1.637146565929 |
| C | -0.284068564881 | 1.842211780628 | 1.327197295294 |
| C | -2.107750150633 | 1.832190928399 | 0.219436145612 |
| H | 0.003704653625 | -1.401432918014 | 1.468242970251 |
| H | 1.365376206140 | -0.313436043782 | 1.568117220301 |
| H | 0.752152579017 | -2.268400573122 | -0.470958532982 |
| H | -1.839997435341 | -0.117782075370 | -0.592217732873 |
| H | 0.493800127851 | 2.226825545046 | 1.974813858919 |
| H | 3.031206063703 | -1.932840457031 | -0.911093401888 |
| H | 3.052385426292 | -0.495943456546 | -0.147024656000 |
| H | -3.041806214498 | 2.107490366273 | -0.252573993850 |
| H | 1.324655499469 | 0.742188876910 | -2.886845002509 |

TS27

| | | | |
|---|---|---|---|
| O | 1.268599823139 | 0.358300088670 | -2.192001545080 |
| O | 0.184402579138 | -1.606355017236 | -2.421711801696 |
| N | -1.504377614479 | 0.878944942555 | -0.285003640404 |
| N | 2.217288597012 | -0.239717054345 | 0.317987053100 |
| N | -0.848464194177 | 2.474760044661 | 1.221371968871 |
| C | -0.085033316381 | -1.109773466053 | 0.588637023108 |

| | | | |
|---|---|---|---|
| C | 1.205277994676 | -1.101919432466 | -0.275258211000 |
| C | -0.689483266084 | 0.256107097669 | 0.774556376902 |
| C | 0.822113448391 | -0.837270779092 | -1.738133877637 |
| C | -0.324620734671 | 1.307727863259 | 1.635952544142 |
| C | -1.548763952782 | 2.169238654307 | 0.117865298103 |
| H | -0.806352793425 | -1.786502811751 | 0.120883814970 |
| H | 0.180024808139 | -1.513452711268 | 1.570605649441 |
| H | 1.584774813809 | -2.130269194847 | -0.263413754317 |
| H | -1.971977579183 | 0.185440924433 | 0.673452979306 |
| H | 0.290178884694 | 1.239160009020 | 2.525399939501 |
| H | 3.099409688131 | -0.349278020600 | -0.178602676973 |
| H | 1.951804989170 | 0.736540259280 | 0.199166170694 |
| H | -2.151407121222 | 2.896743912100 | -0.412192261851 |
| H | 0.928237566446 | 0.443829097066 | -3.102816456852 |

TS28

| | | | |
|---|---|---|---|
| O | 1.133174277614 | 0.547936926601 | -2.154535623006 |
| O | -0.663245051267 | -0.841786993926 | -2.050650493840 |
| N | -0.326728679275 | 0.716345433212 | -0.319166782799 |
| N | 2.470168078697 | -0.666783224013 | -0.039065791546 |
| N | -1.413098263263 | 2.209532692194 | 0.896506299984 |
| C | 0.278526183428 | -1.150034372087 | 0.985258487956 |
| C | 1.120381947942 | -1.124299244023 | -0.321370806103 |
| C | -0.389817195817 | 0.196411497112 | 0.957796812522 |
| C | 0.310262989675 | -0.170493143068 | -1.281533022123 |
| C | -1.065852228482 | 1.134357408742 | 1.696269162073 |
| C | -0.960663043145 | 1.926452142425 | -0.308408805203 |
| H | -0.455591963371 | -1.966752175547 | 0.965887240893 |
| H | 0.925146510182 | -1.314052813882 | 1.852046599311 |
| H | 1.195197156841 | -2.115024221852 | -0.792494814769 |
| H | -0.609227502434 | -1.793538919370 | -1.873508252170 |
| H | -1.318641839804 | 1.117227765131 | 2.747791144422 |
| H | 2.926113897702 | -0.418220559527 | -0.916396243767 |
| H | 2.427651102135 | 0.195977483609 | 0.504346193362 |
| H | -1.044896171886 | 2.553967125882 | -1.184283457887 |
| H | 0.774863001919 | 0.388411841128 | -3.042709668093 |

TS29

| | | | |
|---|---|---|---|
| O | -0.238286411122 | -1.674735644422 | -1.693281736025 |
| O | 0.883074119863 | 0.015391494570 | -2.707747944494 |
| N | -0.326147053905 | 1.562024609130 | 0.576811399103 |
| N | 2.265082516252 | 0.706241561424 | -0.480864644434 |
| N | -2.498537034476 | 1.226959672591 | 0.911146015915 |
| C | 0.700283629220 | -0.725544335370 | 0.807440733832 |
| C | 1.558952386247 | -0.581637878676 | -0.473172005221 |
| C | -0.467549476046 | 0.210173076469 | 0.825236833961 |
| C | 0.702412656827 | -0.673008016456 | -1.736034840945 |
| C | -1.820320830807 | 0.032516185743 | 1.028014154612 |
| C | -1.570645138379 | 2.118783759766 | 0.631463975876 |
| H | 0.336529740571 | -1.753441827994 | 0.885599832171 |
| H | 1.360487880669 | -0.554000467345 | 1.669349361730 |
| H | 2.241141873951 | -1.450078313896 | -0.517877273998 |
| H | 0.559821782546 | 1.963110585623 | 0.279691078179 |
| H | -2.337068039061 | -0.884945803149 | 1.282105461150 |
| H | 3.131720867520 | 0.637199584652 | 0.047941055628 |
| H | 2.501567556582 | 0.942227690001 | -1.443629562892 |
| H | -1.741987622697 | 3.173118196799 | 0.459145855091 |
| H | -1.085630908095 | -1.300776811723 | -1.387898626105 |

TS30

| | | | |
|---|---|---|---|
| O | 1.123782703013 | -2.522400322332 | -1.816060621422 |
| O | 1.261253298629 | -0.664805423447 | -3.088803510029 |
| N | -0.267650138520 | 1.544672353627 | 1.292179649630 |
| N | 1.478204652353 | 0.996688813974 | -0.835050023654 |
| N | -2.460439355383 | 1.853668922186 | 1.502473879011 |
| C | -0.122590259356 | -0.721515977526 | 0.126169103401 |
| C | 1.208596106435 | -0.428785469159 | -0.663958712607 |
| C | -0.865619114542 | 0.458509257801 | 0.678086140727 |
| C | 1.201348019611 | -1.182086153012 | -1.995402550092 |
| C | -2.217054833356 | 0.673663208109 | 0.835643205134 |
| C | -1.268039501190 | 2.357334055623 | 1.743058124967 |
| H | -0.821266532824 | -1.269743514635 | -0.513751610759 |
| H | 0.130846247554 | -1.417082925403 | 0.936260747125 |
| H | 2.038012382971 | -0.846446574232 | -0.081540605386 |
| H | 0.707533101661 | 1.778275542503 | 1.143157430378 |
| H | -3.028801939632 | 0.031466869881 | 0.517542851644 |
| H | 2.349423344931 | 1.102460747547 | -1.353791727142 |
| H | 0.760915027046 | 1.398677247068 | -1.439571049193 |
| H | -1.068411665709 | 3.300626997942 | 2.234412004308 |
| H | 1.111177408972 | -2.921639162821 | -2.706839767588 |

TS31

| | | | |
|---|---|---|---|
| O | 0.687981062566 | -2.002122809505 | -2.287144555142 |
| O | 0.609180477551 | 0.233450170159 | -2.530682421915 |

| | | | |
|---|---|---|---|
| N | -0.832491293898 | 1.284972925138 | -0.104886071891 |
| N | 2.108623344763 | 0.540222408399 | -0.188426187750 |
| N | -2.178882819424 | 1.733797594685 | 1.607570415199 |
| C | 0.262339855576 | -0.919094980985 | 0.590190937568 |
| C | 1.422059405280 | -0.721699600254 | -0.423942201432 |
| C | -0.641219208905 | 0.264198980489 | 0.806427407950 |
| C | 0.873951493562 | -0.736099300436 | -1.851560348209 |
| C | -1.488934607280 | 0.567846275354 | 1.849322525714 |
| C | -1.750683391740 | 2.139394125276 | 0.429433926357 |
| H | -0.307570946228 | -1.800692410357 | 0.266241517070 |
| H | 0.709434792343 | -1.194587329023 | 1.553925081548 |
| H | 2.059200531803 | -1.617762127996 | -0.339474366138 |
| H | -0.277913536548 | 1.417891066485 | -0.944137445167 |
| H | -1.644587127683 | 0.002920453084 | 2.760107632054 |
| H | 2.379266177977 | 0.611955758577 | 0.789597088915 |
| H | 2.955491676817 | 0.604746206084 | -0.748671095050 |
| H | -2.064446865300 | 3.040182193860 | -0.081314089294 |
| H | 0.288408445057 | -1.933811244878 | -3.175264781131 |

TS32

| | | | |
|---|---|---|---|
| O | -0.361260710109 | -0.356205297901 | -2.000352427233 |
| O | 1.471767695133 | -1.420086383970 | -2.754285008532 |
| N | -0.508342618447 | 1.560379635862 | 0.518891769164 |
| N | 2.061974359919 | 0.876992546741 | -0.578812918569 |
| N | -2.543161378119 | 1.182666955243 | 1.330305831171 |
| C | 0.641299774832 | -0.688617161668 | 0.721890774254 |
| C | 1.542941271275 | -0.510505039010 | -0.519969935530 |
| C | -0.541244352413 | 0.221973247041 | 0.855879239035 |
| C | 0.893282958837 | -0.826344803299 | -1.869107007311 |
| C | -1.804931363505 | 0.020114745250 | 1.362291827690 |
| C | -1.735977759411 | 2.081594304850 | 0.805625134212 |
| H | 0.291116824079 | -1.726714756638 | 0.753788712897 |
| H | 1.308821445438 | -0.576913138200 | 1.592463155861 |
| H | 2.354916932301 | -1.247842837905 | -0.443934669868 |
| H | 0.268115454302 | 1.968989480670 | 0.007181756680 |
| H | -2.223176935639 | -0.899772777372 | 1.751246850451 |
| H | 2.654574364824 | 1.030247340655 | 0.238113226728 |
| H | 2.674410591434 | 0.973180071700 | -1.389152850625 |
| H | -1.983998840215 | 3.117540427294 | 0.614948020679 |
| H | -0.655320256484 | -0.605564517936 | -2.897820984780 |

TS33

| | | | |
|---|---|---|---|
| O | 0.070816622884 | -2.066926615525 | -1.652482890155 |
| O | 0.544572982420 | -0.123648633585 | -2.689661850064 |
| N | -0.356076803454 | 1.669671622068 | 1.235103021800 |
| N | 2.324179869201 | 0.643556786501 | -0.749294667387 |
| N | -2.468333113379 | 1.378601876056 | 0.616496276856 |
| C | 0.710361235052 | -0.598546523662 | 0.776280539403 |
| C | 1.563154546862 | -0.581534311746 | -0.527896345631 |
| C | -0.444609180070 | 0.353484106356 | 0.805892314921 |
| C | 0.684601568210 | -0.864071430117 | -1.742088374445 |
| C | -1.761676237910 | 0.211962388605 | 0.429965064643 |
| C | -1.596423879999 | 2.231994695294 | 1.104357136854 |
| H | 0.331831006061 | -1.615134745743 | 0.919923885114 |
| H | 1.394882651130 | -0.403736838602 | 1.613562392921 |
| H | 2.250965942499 | -1.438190532686 | -0.448751440314 |
| H | 0.459588335620 | 2.113952648714 | 1.631633884127 |
| H | -2.237292548268 | -0.676786191517 | 0.036336436146 |
| H | 2.239416193577 | 1.276052895814 | 0.040410838853 |
| H | 1.950242048830 | 1.122522922794 | -1.566755201617 |
| H | -1.797820834215 | 3.258236275991 | 1.381889319575 |
| H | -0.467796803327 | -2.159377769942 | -2.460967808716 |

TS34

| | | | |
|---|---|---|---|
| O | 0.120992312429 | -2.068256482928 | -1.832928113184 |
| O | 0.716146061567 | 0.288377600592 | -2.677010646338 |
| N | -0.428764799183 | 1.554815993990 | 0.265893835049 |
| N | 2.338648232426 | 0.810709257987 | -0.260998230987 |
| N | -2.344588607239 | 1.400734819745 | 1.384707521724 |
| C | 0.509572073147 | -0.773626787137 | 0.421341856562 |
| C | 1.702570998997 | -0.500757254992 | -0.374585585461 |
| C | -0.495282345281 | 0.240548650087 | 0.709158458230 |
| C | 1.136760619316 | -0.481889862127 | -1.911439369863 |
| C | -1.702970497723 | 0.182360395491 | 1.390840677964 |
| C | -1.556144239211 | 2.201510426951 | 0.702318000381 |
| H | -0.003227132564 | -1.736368009337 | -0.793505594763 |
| H | 0.606893790705 | -1.585249991009 | 1.138887670484 |
| H | 2.385185069764 | -1.358741028799 | -0.348919992952 |
| H | 0.377491811863 | 1.943926013536 | -0.208685912409 |
| H | -2.136667106085 | -0.678207238090 | 1.884116781539 |
| H | 2.537391984168 | 0.976249950998 | 0.723601885175 |
| H | 3.219929594716 | 0.843545411353 | -0.773113436805 |
| H | -1.744937364749 | 3.244011635298 | 0.482613949085 |
| H | -0.696981812937 | -1.807371124992 | -2.292874063973 |

TS35

| | | | |
|---|---|---|---|
| O | 1.035691850370 | -2.538924361084 | -1.654512808277 |
| O | 1.956472354301 | -0.898876928139 | -2.913837219857 |
| N | -0.575689746353 | 1.832150479743 | 0.830950273023 |
| N | 1.486184129946 | 1.107182185765 | -1.034397646975 |
| N | -2.171931246336 | 1.389281982364 | 2.313152918568 |
| C | -0.617871276462 | -0.159560508604 | -0.719805896031 |
| C | 0.928737426675 | -0.234319976615 | -0.829800178521 |
| C | -1.097999689462 | 0.604410121877 | 0.474153946220 |
| C | 1.345321656353 | -1.218654478947 | -1.927671041389 |
| C | -2.083891280400 | 0.359454551017 | 1.403636875835 |
| C | -1.245885313642 | 2.250339205131 | 1.942359311106 |
| H | -0.999648079680 | 0.284226097513 | -1.653320004302 |
| H | -1.028800230777 | -1.173027728620 | -0.659538900204 |
| H | 1.311786781172 | -0.631296075582 | 0.117760480926 |
| H | 0.241148058244 | 2.221718427629 | 0.368009219225 |
| H | -2.738333175148 | -0.501028590409 | 1.464350546309 |
| H | 2.498656086388 | 1.067176406765 | -0.921672275892 |
| H | 1.346389432422 | 1.364447093113 | -2.012787532240 |
| H | -1.018877710141 | 3.184616596269 | 2.438786310521 |
| H | 0.126595501217 | -2.747686526817 | -1.922094612804 |

TS36

| | | | |
|---|---|---|---|
| O | 2.246134318080 | -2.104700733841 | -1.622684505169 |
| O | 0.720669373344 | -1.240908871432 | -3.042890070571 |
| N | -0.415993948075 | 1.716281712739 | 1.030083285107 |
| N | 1.707047858981 | 0.777458643345 | -0.688562603662 |
| N | -2.317933045141 | 1.519328387450 | 2.162721307075 |
| C | -0.650676664507 | 0.037352000873 | -0.846607577304 |
| C | 0.818606290125 | -0.406313600704 | -0.749670144230 |
| C | -1.143756514636 | 0.735113089347 | 0.382998707055 |
| C | 1.206625951107 | -1.289044284407 | -1.934415431533 |
| C | -2.313310888122 | 0.639201386136 | 1.103070859417 |
| C | -1.159942046362 | 2.142872794942 | 2.091169624830 |
| H | -0.755482798726 | 0.666104762793 | -1.742946758860 |
| H | -1.278709753943 | -0.841059061450 | -1.028037513792 |
| H | 0.976306165081 | -0.973083614391 | 0.173091203316 |
| H | 0.537636661916 | 1.945685842847 | 0.767282658268 |
| H | -3.155130736076 | -0.014296687022 | 0.912126525455 |
| H | 2.670001460438 | 0.480934997053 | -0.533978887199 |
| H | 1.688316365710 | 1.257817446675 | -1.589350389856 |
| H | -0.810163072031 | 2.904991827515 | 2.775185930793 |
| H | 2.473185686738 | -2.587063298961 | -2.440925713380 |

TS37

| | | | |
|---|---|---|---|
| O | 0.499433415278 | -1.864891372967 | -2.572858446893 |
| O | 2.626393950657 | -1.482811399903 | -1.935678735455 |
| N | -0.706465868735 | 1.847570794333 | 0.701248484541 |
| N | 1.143115906637 | 1.151681093418 | -1.400642306763 |
| N | -2.032005432978 | 1.434608786239 | 2.436756371961 |
| C | -0.619595985045 | -0.356949987589 | -0.535294668329 |
| C | 0.876445930749 | -0.200886484263 | -0.860174915791 |
| C | -1.071528893712 | 0.518688070854 | 0.593321185899 |
| C | 1.442001929368 | -1.256684424655 | -1.813154003014 |
| C | -1.892042716121 | 0.294942914818 | 1.675996788584 |
| C | -1.300160518370 | 2.343126885103 | 1.825097624316 |
| H | -1.195087293802 | -0.159145375495 | -1.452025221422 |
| H | -0.826459065667 | -1.399514819486 | -0.273397474369 |
| H | 1.460100030811 | -0.290805752376 | 0.061723792149 |
| H | -0.029730292911 | 2.275932201604 | 0.076411571792 |
| H | -2.392867837834 | -0.625976473793 | 1.946795044836 |
| H | 2.145852106053 | 1.259799254895 | -1.552036358640 |
| H | 0.699563649049 | 1.244572717139 | -2.315695248567 |
| H | -1.164144365001 | 3.366610849626 | 2.149093275785 |
| H | 0.979300107553 | -2.475092109665 | -3.165715295339 |

TS38

| | | | |
|---|---|---|---|
| O | 1.034742802253 | -2.499048820785 | -1.689906782490 |
| O | 1.780150927766 | -0.819384010881 | -3.000122081339 |
| N | -0.505732011875 | 1.841112564204 | 0.878134362351 |
| N | 1.583842922362 | 1.036188812143 | -0.908422419332 |
| N | -2.308778336260 | 1.548115808599 | 2.172555082487 |
| C | -0.560676353094 | -0.184180802010 | -0.631156569883 |
| C | 0.977798866356 | -0.293707041183 | -0.766623313565 |
| C | -1.017226076637 | 0.571110925578 | 0.589785906677 |
| C | 1.323482563290 | -1.205028855102 | -1.946170468478 |
| C | -2.245302993879 | 0.375814843049 | 1.361665574556 |
| C | -1.335494678589 | 2.354346018229 | 1.822754687868 |
| H | -0.958682127952 | 0.315522440463 | -1.524711434306 |
| H | -1.010751797983 | -1.180310708100 | -0.595973578364 |
| H | 1.370026705887 | -0.760351898489 | 0.144393603122 |
| H | 0.361495607257 | 2.194339243110 | 0.479966803530 |
| H | -0.986925070048 | -0.196587594972 | 1.573957990091 |

| | | | |
|---|---|---|---|
| H | 2.594219679353 | 0.957608862728 | -0.796656981610 |
| H | 1.452328259781 | 1.337881971025 | -1.875610530272 |
| H | -1.163026751430 | 3.342627832327 | 2.240462403369 |
| H | 1.248391401697 | -3.002675731867 | -2.498711943370 |

TS39

| | | | |
|---|---|---|---|
| O | 1.155576644229 | -2.518593832831 | -1.892718928084 |
| O | 2.058851300338 | -0.691604271328 | -2.848530158359 |
| N | 0.161363893293 | 2.356420399553 | 1.541132365453 |
| N | 0.651999220351 | 1.006443250325 | -1.223663611231 |
| N | -2.157686391133 | 1.896053759198 | 1.602460232644 |
| C | -0.775122704624 | -0.932127323834 | -0.636769561848 |
| C | 0.680405474499 | -0.395319896893 | -0.876025229132 |
| C | -1.259146275185 | -0.270979196747 | 0.589404794671 |
| C | 1.360984199156 | -1.184422144912 | -1.992013904044 |
| C | -1.905335304647 | 0.807996102959 | 0.954649001046 |
| C | -1.030016719211 | 2.619624804356 | 1.949023335540 |
| H | -1.364504501965 | -0.660881455554 | -1.520810374812 |
| H | -0.745977655292 | -2.018038790774 | -0.523133776933 |
| H | 1.262809266138 | -0.633316611042 | 0.035040366130 |
| H | 0.831405677725 | 3.008983837122 | 1.951527383428 |
| H | -2.792208683946 | 0.085883796047 | 0.647215253682 |
| H | 0.597758632674 | 1.574007486256 | -0.373641125758 |
| H | 1.491920194493 | 1.243602016436 | -1.745135877601 |
| H | -1.265443306631 | 3.453001092977 | 2.619120225204 |
| H | 1.668547519635 | -2.926193427389 | -2.616083270524 |

TS40

| | | | |
|---|---|---|---|
| O | 2.513722470872 | -1.383983402079 | -2.116604506983 |
| O | 0.401359313371 | -1.718949334966 | -2.817116438885 |
| N | -0.456390335184 | 1.864308037802 | 0.921501257458 |
| N | 1.774559866121 | 0.315564245922 | -0.161857490858 |
| N | -2.286260247322 | 1.528106626629 | 2.134982560201 |
| C | -0.602035012150 | 0.132536429909 | -0.907187167293 |
| C | 0.744161873113 | -0.600135707399 | -0.688155530043 |
| C | -1.117775157202 | 0.805912306860 | 0.322922499847 |
| C | 1.164772078395 | -1.279246086438 | -1.988709851202 |
| C | -2.244167579714 | 0.626471273395 | 1.093682395531 |
| C | -1.195021266972 | 2.252270498048 | 2.002427588493 |
| H | -0.457048418122 | 0.865015287174 | -1.712441911091 |
| H | -1.341836936307 | -0.588637574574 | -1.264260194553 |
| H | 0.560350015502 | -1.432664406254 | 0.011078061328 |
| H | 0.452106430320 | 2.193445678561 | 0.620549558330 |
| H | -3.033711104149 | -0.099631619469 | 0.947691982780 |
| H | 1.873274160197 | 0.170129167903 | 0.839715911247 |
| H | 2.677380821221 | 0.098726391665 | -0.575795889728 |
| H | -0.892300739824 | 3.064220116600 | 2.650691068015 |
| H | 2.670659219177 | -1.845586028447 | -2.962078879070 |

TS41

| | | | |
|---|---|---|---|
| O | 0.858493399092 | -2.025525031483 | -2.467520456483 |
| O | 2.346616440312 | -0.372440153951 | -2.831516497815 |
| N | -0.503887881685 | 1.991084369011 | 0.845815454993 |
| N | 2.037997688404 | 0.570096860791 | -0.301796226647 |
| N | -2.108057239454 | 1.270314345191 | 2.201010962330 |
| C | -0.390586780592 | 0.222000134991 | -0.957928058163 |
| C | 1.057133961534 | -0.359913002019 | -0.771786229107 |
| C | -0.956003932221 | 0.802454720024 | 0.295203695928 |
| C | 1.521450798476 | -0.899451524279 | -2.117605869591 |
| C | -1.945197374792 | 0.385727050396 | 1.157394506063 |
| C | -1.226923191999 | 2.221844499247 | 1.983124946743 |
| H | -0.347364909773 | 0.983388002858 | -1.749323940092 |
| H | -1.054798677554 | -0.574759111426 | -1.308998664062 |
| H | 0.974819470165 | -1.208848230274 | -0.080767966554 |
| H | 0.245974659333 | 2.551423753019 | 0.466807907976 |
| H | -2.556036484227 | -0.504179654476 | 1.075713057832 |
| H | 2.066636391057 | 0.839746124274 | 0.664590248883 |
| H | 2.609400050303 | 1.054631463617 | -0.973726148088 |
| H | -1.068200975124 | 3.093915086841 | 2.603840762041 |
| H | 1.181267419184 | -2.269119663487 | -3.355974625972 |

TS42

| | | | |
|---|---|---|---|
| O | 2.460808695009 | -1.220498473081 | -2.365690458353 |
| O | 0.268977001145 | -1.534979820879 | -2.771979902243 |
| N | -0.337521171166 | 1.176323519166 | 1.571050118592 |
| N | 1.680203850864 | 0.552024379455 | -0.309124234736 |
| N | -2.321631929000 | 2.169730953693 | 1.421976732739 |
| C | -0.511483465840 | -0.707279638176 | -0.144706016900 |
| C | 0.990248832919 | -0.724617544168 | -0.557235436166 |
| C | -1.019205552991 | 0.513153593344 | 0.564830333029 |
| C | 1.148591255167 | -1.205568135821 | -2.009727445627 |
| C | -2.242649737245 | 1.142669413493 | 0.507019676720 |
| C | -1.153889099329 | 2.171094986673 | 2.029135434670 |
| H | -1.118715482427 | -0.848110683658 | -1.043161711805 |

| | | | |
|---|---|---|---|
| H | -0.690742115822 | -1.595978925918 | 0.475537648407 |
| H | 1.519531892974 | -1.470193027923 | 0.049516824550 |
| H | 0.662360827642 | 1.084677195172 | 1.704324626687 |
| H | -3.078242042817 | 0.899413213828 | -0.137074241882 |
| H | 2.640821855960 | 0.481950739736 | -0.640336145115 |
| H | 1.234949695000 | 1.295760743424 | -0.846667322119 |
| H | -0.843532912012 | 2.863517768389 | 2.800809759639 |
| H | 2.491065938553 | -1.526297283502 | -3.291764089212 |

TS43

| | | | |
|---|---|---|---|
| O | 2.516911841385 | -1.793423920044 | -1.798984598859 |
| O | 0.655320949332 | -1.314253086010 | -3.002230170203 |
| N | -0.371432370063 | 1.631095678629 | 1.143590879120 |
| N | 1.798775100218 | 0.707396158902 | -0.525199152260 |
| N | -2.371934053395 | 1.503653548009 | 2.103314360099 |
| C | -0.591341668747 | 0.183087906355 | -0.917161755968 |
| C | 0.827269340108 | -0.385064054211 | -0.758226637677 |
| C | -1.126255918240 | 0.796914945612 | 0.339916500378 |
| C | 1.253113312390 | -1.226848386966 | -1.963819795578 |
| C | -2.356042024698 | 0.740920588285 | 0.956342109291 |
| C | -1.161680123268 | 2.016784853987 | 2.186700868893 |
| H | -0.576943923235 | 0.905740606508 | -1.747328587441 |
| H | -1.264526480070 | -0.617656421346 | -1.237614286106 |
| H | 0.862179225057 | -1.029303610995 | 0.130486987203 |
| H | 0.616873845166 | 1.796828172208 | 0.977251386706 |
| H | -3.233212931438 | 0.194963277560 | 0.632783744440 |
| H | 2.729368054822 | 0.309031977206 | -0.404295864486 |
| H | 1.845727535275 | 1.294816145608 | -1.359356219778 |
| H | -0.805085330657 | 2.667760100263 | 2.974171187930 |
| H | 2.441562335273 | -2.657720954515 | -1.362398284120 |

TS44

| | | | |
|---|---|---|---|
| O | 0.886460900310 | -2.285313509266 | -1.929148896721 |
| O | 2.223989839231 | -0.669272410909 | -2.759944420892 |
| N | -0.452479587825 | 1.800525173165 | 1.011860481203 |
| N | 1.830647657483 | 0.954523772414 | -0.514833688306 |
| N | -2.141381285985 | 1.062175574123 | 2.285403445165 |
| C | -0.491312833871 | 0.218396507577 | -0.961591741787 |
| C | 0.975124308319 | -0.220260211977 | -0.724573061431 |
| C | -1.115909662670 | 0.804045415990 | 0.281971820854 |
| C | 1.450322979111 | -1.059733715834 | -1.912458497323 |
| C | -2.190812168637 | 0.253068035187 | 1.113294452910 |
| C | -1.104412995114 | 1.860327868683 | 2.202548822688 |
| H | -0.496723677011 | 0.936909392042 | -1.792249908476 |
| H | -1.099678393241 | -0.641855404981 | -1.254450227911 |
| H | 1.005262371486 | -0.850243502597 | 0.170893631706 |
| H | 0.470243612643 | 2.145876438618 | 0.757475043812 |
| H | -2.285973226469 | 1.115367044795 | 0.008560802218 |
| H | 2.717477268824 | 0.657447961408 | -0.109190014415 |
| H | 2.074332798596 | 1.329089202836 | -1.433597713739 |
| H | -0.779553071348 | 2.544250600602 | 2.982177782395 |
| H | 1.213852060830 | -2.737428094492 | -2.730276208910 |

TS45

| | | | |
|---|---|---|---|
| O | 2.530660677148 | -1.278868378337 | -2.075155009253 |
| O | 0.558084279543 | -1.716370820982 | -3.068187579949 |
| N | 0.215838836261 | 1.750550181695 | 2.102061281183 |
| N | 1.297967293899 | 0.708056590839 | -0.656210611609 |
| N | -2.073923414555 | 2.099350764707 | 1.625056936001 |
| C | -0.915778484906 | -0.256099208944 | -1.213196532047 |
| C | 0.587822894575 | -0.534508478437 | -0.925878877025 |
| C | -1.516102157329 | 0.149954607232 | 0.071593426926 |
| C | 1.180218410014 | -1.229205155752 | -2.152205853751 |
| C | -1.944752728549 | 1.216841072404 | 0.692228677172 |
| C | -0.922059061488 | 2.286521144700 | 2.370753695822 |
| H | -0.966566084407 | 0.518611143286 | -1.986916516673 |
| H | -1.358453041061 | -1.181537114862 | -1.591077319185 |
| H | 0.643638307628 | -1.279885916451 | -0.108238758425 |
| H | 0.909635414671 | 2.012775989571 | 2.804038833458 |
| H | -2.871183883474 | 0.923987542559 | 0.007810488058 |
| H | 0.993061725852 | 1.088191609536 | 0.247612999188 |
| H | 2.294961600778 | 0.516944306702 | -0.589354852382 |
| H | -1.087370337806 | 2.948761045057 | 3.226583327524 |
| H | 2.834870397602 | -1.738852489640 | -2.880254855695 |

TS46

| | | | |
|---|---|---|---|
| O | 1.440235902886 | -1.637204335866 | -2.582526954997 |
| O | 0.234824913126 | 0.232499908458 | -2.325296357154 |
| N | -1.239626470797 | 1.365923772651 | -0.172573239906 |
| N | 2.564188787708 | -1.391660879734 | -0.116053575500 |
| N | -2.226168964127 | 1.567149857952 | 1.809102110045 |
| C | 1.096302190317 | 0.543304876434 | 0.419801804838 |
| C | 1.234412529985 | -0.805260042661 | -0.341554488001 |
| C | -0.295464008767 | 0.977645702426 | 0.759373163290 |

| | | | |
|---|---|---|---|
| C | 0.905232048593 | -0.650595440832 | -1.826075498227 |
| C | -0.935710463968 | 1.116054669673 | 1.971832966571 |
| C | -2.373253423308 | 1.701978886542 | 0.506902045367 |
| H | 1.642945778178 | 0.406142018650 | 1.356606532106 |
| H | 1.635018215057 | 1.319121445433 | -0.140395042038 |
| H | 0.455527373592 | -1.479812166483 | 0.046531628229 |
| H | -1.085165471285 | 1.324242687651 | -1.172626545585 |
| H | -0.528017020290 | 0.922996559723 | 2.956280731101 |
| H | 2.481486452105 | -2.289943266706 | 0.350436768228 |
| H | 3.042271717282 | -1.569084203104 | -0.993983413430 |
| H | -3.271351743194 | 2.036720456997 | 0.004311916831 |
| H | 1.160788630694 | -1.458224767579 | -3.500818354114 |

TS47

| | | | |
|---|---|---|---|
| O | 1.070311818541 | -1.815828372659 | -2.525603473330 |
| O | 0.390746507749 | 0.322005909111 | -2.316759012534 |
| N | -1.179186819587 | 1.428867014639 | -0.190231938927 |
| N | 2.632872869796 | -1.300696570446 | -0.153591250145 |
| N | -2.279198261993 | 1.498509427833 | 1.739815110239 |
| C | 1.106638685233 | 0.527219916832 | 0.473127658430 |
| C | 1.265590728082 | -0.787852497080 | -0.318713000387 |
| C | -0.298384931254 | 0.954337217910 | 0.764329372743 |
| C | 0.873805016117 | -0.653727433934 | -1.795099524589 |
| C | -1.007179832064 | 1.013797791650 | 1.943945024760 |
| C | -2.346901385940 | 1.733263552604 | 0.445586079252 |
| H | 1.616099116171 | 0.369214229403 | 1.428095543068 |
| H | 1.653889859702 | 1.323670077887 | -0.051540622427 |
| H | 0.591315690688 | -1.541116781103 | 0.109187278056 |
| H | -0.971440495092 | 1.465812568313 | -1.180245034617 |
| H | -0.661894726074 | 0.739551305939 | 2.932883540399 |
| H | 2.728912637093 | -2.222001461405 | -0.573464017965 |
| H | 3.313727577798 | -0.678933087069 | -0.587675254935 |
| H | -3.208512877283 | 2.120744651728 | -0.082146685973 |
| H | 1.985101007614 | -1.874469264273 | -2.841712474134 |

TS48

| | | | |
|---|---|---|---|
| O | 1.320670356829 | -1.528773691767 | -2.634384204528 |
| O | -0.152530213109 | -0.053471621181 | -3.615646468305 |
| N | -0.794563859882 | 1.082630570332 | -0.032451248086 |
| N | 2.530696265840 | -0.803992743709 | -0.538415732297 |
| N | -2.108859810089 | 1.945195746879 | 1.547605342346 |
| C | 1.180707245531 | 0.184990823960 | 1.276379485693 |
| C | 1.421685059126 | -0.729907354708 | 0.125452266690 |
| C | -0.165817465606 | 0.852327284292 | 1.173995258287 |
| C | 0.277303561818 | -0.348409975698 | -2.538020944402 |
| C | -0.995792728785 | 1.395070529818 | 2.131089288263 |
| C | -1.956883821572 | 1.727854416799 | 0.252682877097 |
| H | 1.201625224021 | -0.418233037943 | 2.197177932031 |
| H | 2.008577958616 | 0.905964193350 | 1.352964300438 |
| H | 0.639019877230 | -1.406535570789 | -0.199593607652 |
| H | -0.471412175718 | 0.685510025455 | -0.939255911402 |
| H | -0.843820785941 | 1.428080739260 | 3.202984504396 |
| H | 2.415127359444 | -1.262143820441 | -1.494423575022 |
| H | 3.288267034614 | -0.154074287421 | -0.350213943504 |
| H | -2.660134359966 | 2.011132611686 | -0.518732260319 |
| H | 1.441003760325 | -1.693035879133 | -3.589603292829 |

TS49

| | | | |
|---|---|---|---|
| O | -0.125665368367 | -1.834189798514 | -1.667757904912 |
| O | 0.730170236563 | 0.135048386837 | -2.350907013443 |
| N | -0.557274317655 | 1.552648575695 | -0.046179712366 |
| N | 2.918277459957 | -0.235171611271 | -0.554399649155 |
| N | -2.472733126879 | 1.273879062461 | 1.047926142400 |
| C | 0.929406256000 | -0.282953149449 | 0.938797226706 |
| C | 1.626680617462 | -0.886860567412 | -0.338330071491 |
| C | -0.365086160874 | 0.455443957285 | 0.781459145454 |
| C | 0.716471572595 | -0.789586777770 | -1.560333432201 |
| C | -1.562837703211 | 0.320527452962 | 1.446257466019 |
| C | -1.839706536772 | 1.986358743293 | 0.141828902588 |
| H | 0.744503208691 | -1.092673987532 | 1.650561438018 |
| H | 1.692251756950 | 0.359203811914 | 1.397751634403 |
| H | 1.815541681795 | -1.947753265586 | -0.151900099972 |
| H | 0.066891111148 | 1.844841530904 | -0.786242564976 |
| H | -1.812050733047 | -0.412106784312 | 2.203421640185 |
| H | 3.438730980146 | -0.742357827665 | -1.269856830284 |
| H | 2.754858239624 | 0.691054482528 | -0.951344715536 |
| H | -2.251350640112 | 2.821821906738 | -0.409170359985 |
| H | -0.704510556231 | -1.655651436785 | -2.434135277421 |

TS50

| | | | |
|---|---|---|---|
| O | 0.624824165581 | -1.840335664718 | -2.270050286155 |
| O | 0.654090232785 | 0.406358401105 | -2.297244890471 |
| N | -1.068656094478 | 1.459261540400 | -0.217990829316 |
| N | 2.772528304145 | -1.120088033511 | -0.352492137430 |

| | | | |
|---|---|---|---|
| N | -2.344626160211 | 1.362497779374 | 1.598956333844 |
| C | 1.152635044765 | 0.512078522336 | 0.558861785014 |
| C | 1.377757965910 | -0.775599661984 | -0.264811729538 |
| C | -0.274835731124 | 0.908574145896 | 0.772451197496 |
| C | 0.829137313701 | -0.637833154026 | -1.694055581327 |
| C | -1.091888139641 | 0.865637088564 | 1.880575978406 |
| C | -2.293963754385 | 1.706097119763 | 0.328522143786 |
| H | 1.621398375627 | 0.347172819670 | 1.533827820010 |
| H | 1.705432749497 | 1.327015402351 | 0.071098946607 |
| H | 0.845461937232 | -1.605983926013 | 0.210365197413 |
| H | -0.769757115892 | 1.580596475546 | -1.178032169888 |
| H | -0.838301477695 | 0.511295758181 | 2.871828482998 |
| H | 3.093242594667 | -2.051783042733 | -0.160900065160 |
| H | 3.462086694050 | -0.403164683679 | -0.501778124853 |
| H | -3.106321061806 | 2.136032543519 | -0.242672492295 |
| H | 0.357452085632 | -1.663813594260 | -3.192290753545 |

TS51

| | | | |
|---|---|---|---|
| O | -0.269380026436 | -1.025391618506 | -2.027659794691 |
| O | 1.714299730118 | -0.176105729703 | -2.671049129915 |
| N | -1.233629592520 | 1.382830087355 | -0.187112824756 |
| N | 2.832232921798 | -1.035047639174 | -0.183178362807 |
| N | -2.321686246781 | 1.244490773711 | 1.743921376300 |
| C | 1.122783990764 | 0.613256745732 | 0.424824605690 |
| C | 1.411051851660 | -0.722368940293 | -0.323145721130 |
| C | -0.308086698994 | 0.917720184036 | 0.733479355211 |
| C | 0.999540743912 | -0.609609666061 | -1.791716926910 |
| C | -1.013787613876 | 0.849883275526 | 1.913614937953 |
| C | -2.420076259079 | 1.552918268713 | 0.469813106355 |
| H | 1.668720224035 | 0.536376484601 | 1.369649802428 |
| H | 1.590574191364 | 1.432579433725 | -0.140299298416 |
| H | 0.824194148936 | -1.515456215624 | 0.148742046864 |
| H | -1.075616839087 | 1.511136252258 | -1.175686850447 |
| H | -0.640377509238 | 0.543560949381 | 2.882446697309 |
| H | 3.013644277560 | -1.969436981290 | -0.548220405080 |
| H | 3.361060549598 | -0.401846181815 | -0.784382669198 |
| H | -3.312028696237 | 1.905915257291 | -0.030996503264 |
| H | -0.416048487138 | -0.925392149960 | -2.988696417087 |

TS52

| | | | |
|---|---|---|---|
| O | 0.724489171519 | -1.824527788487 | -2.333436872757 |
| O | 0.610145456942 | 0.417851466680 | -2.287181037819 |
| N | -1.084088773718 | 1.459089001181 | -0.208696654455 |
| N | 2.769948657550 | -1.152428814461 | -0.387554320851 |
| N | -2.338122084674 | 1.383996154855 | 1.624735437597 |
| C | 1.135399209305 | 0.490740787063 | 0.547498192550 |
| C | 1.344198794616 | -0.793720470987 | -0.279530234385 |
| C | -0.285594124085 | 0.902555774601 | 0.773768435168 |
| C | 0.843574528783 | -0.632638888168 | -1.718433203119 |
| C | -1.088352192918 | 0.873578579312 | 1.892750590775 |
| C | -2.299072670828 | 1.722076180235 | 0.352158669060 |
| H | 1.597652831407 | 0.320715721921 | 1.526599007542 |
| H | 1.697739929579 | 1.299471380266 | 0.060759179037 |
| H | 0.770686125747 | -1.610804260551 | 0.174341945513 |
| H | -0.793716222585 | 1.571165619463 | -1.172671005261 |
| H | -0.826588822372 | 0.520132552079 | 2.882276599548 |
| H | 3.346022754557 | -0.489974091619 | 0.123919902398 |
| H | 2.949071239138 | -2.078211037641 | -0.013519477300 |
| H | -3.113517472172 | 2.159132977448 | -0.210511974013 |
| H | 0.474031315927 | -1.637264533633 | -3.258215889636 |

TS53

| | | | |
|---|---|---|---|
| O | 1.390592901658 | -1.688163217861 | -2.604461208412 |
| O | 0.260621877908 | 0.243028765823 | -2.327165632684 |
| N | -1.236236067826 | 1.367784096819 | -0.171929202064 |
| N | 2.546867436973 | -1.371853420713 | -0.215629527721 |
| N | -2.240392764290 | 1.584604401912 | 1.799410209825 |
| C | 1.075574134501 | 0.502636530738 | 0.442473822777 |
| C | 1.185640566280 | -0.826977295917 | -0.340984365350 |
| C | -0.312313157992 | 0.957688167899 | 0.770295627384 |
| C | 0.901047599101 | -0.652297942519 | -1.832622023263 |
| C | -0.961894920584 | 1.106850258411 | 1.975898247190 |
| C | -2.370061824791 | 1.725309431222 | 0.496020205890 |
| H | 1.596774684144 | 0.361674154345 | 1.396688746663 |
| H | 1.634940443620 | 1.274177630588 | -0.101930449999 |
| H | 0.402903363610 | -1.512772375788 | 0.035394157413 |
| H | -1.071654206456 | 1.329692316394 | -1.170721740351 |
| H | -0.570062378471 | 0.902640765674 | 2.964633515287 |
| H | 2.788620154600 | -1.443831729756 | 0.771065978426 |
| H | 2.560807738476 | -2.318909093324 | -0.588463701491 |
| H | -3.255398074183 | 2.078863706549 | -0.015979703535 |
| H | 2.281022492691 | -1.462859128603 | -2.922433561981 |

TS54

| | | | |
|---|---|---|---|
| O | -0.167357569488 | -1.608320471048 | -1.638931480866 |
| O | 0.920405777066 | 0.226443460617 | -2.364812374279 |
| N | -0.613803456180 | 1.556783379230 | -0.055061328540 |
| N | 2.966698140091 | -0.303907997820 | -0.536533156586 |
| N | -2.529399492532 | 1.120727731550 | 0.984077454605 |
| C | 1.006798657823 | -0.112970833283 | 0.950286089026 |
| C | 1.631799493832 | -0.854080845535 | -0.293912163955 |
| C | -0.350881996555 | 0.495652594296 | 0.797602034423 |
| C | 0.788159630898 | -0.658770479024 | -1.546939479353 |
| C | -1.551296418660 | 0.257995762402 | 1.426235177406 |
| C | -1.933074224803 | 1.879774873506 | 0.089945288336 |
| H | 0.966265795013 | -0.809367775506 | 1.793160092707 |
| H | 1.729939028928 | 0.670736859181 | 1.209377923597 |
| H | 1.617403081322 | -1.934835993031 | -0.085007855405 |
| H | 0.033546609077 | 1.932904928437 | -0.735751410124 |
| H | -1.760263173805 | -0.488901709790 | 2.181506117351 |
| H | 3.583540537147 | -0.576499374245 | 0.226849682146 |
| H | 3.364931418179 | -0.691893632087 | -1.389900807476 |
| H | -2.398110448769 | 2.675120129112 | -0.477590887533 |
| H | -0.712536251421 | -1.380451684100 | -2.416035404614 |

TS55

| | | | |
|---|---|---|---|
| O | 0.924102040045 | -1.349433593963 | -2.662558481257 |
| O | 1.072865149194 | 0.836888253460 | -2.132223452788 |
| N | -1.445001163675 | 0.513182912393 | -0.076790412907 |
| N | 2.514211673860 | -1.446950313524 | -0.256390963459 |
| N | -2.130873641450 | 2.069256229427 | 1.453644963043 |
| C | 1.039988095696 | 0.288015694039 | 0.660246230998 |
| C | 1.171974148813 | -0.851065197024 | -0.357770370410 |
| C | -0.378011439381 | 0.736581037549 | 0.912185846128 |
| C | 1.037829997229 | -0.318561669224 | -1.786394479102 |
| C | -0.882485029478 | 1.704221836237 | 1.797267447518 |
| C | -2.425214316705 | 1.331911575733 | 0.373843765572 |
| H | 1.488437020885 | -0.027984505894 | 1.610286178893 |
| H | 1.628681824271 | 1.134552867219 | 0.292222862025 |
| H | 0.359896845061 | -1.584876893710 | -0.213970950538 |
| H | -1.189494840025 | -0.280032728879 | 0.873463627982 |
| H | -0.379582413062 | 2.136085862179 | 2.654829210695 |
| H | 2.613940605494 | -1.898676494340 | 0.651434665395 |
| H | 2.609470593766 | -2.180007449622 | -0.957302763382 |
| H | -3.399083994660 | 1.345702703522 | -0.099619776685 |
| H | 0.886039010929 | -0.949039143262 | -3.551776252599 |

TS56

| | | | |
|---|---|---|---|
| O | 2.413578201574 | -1.697049336145 | -2.047270634441 |
| O | 1.407861810940 | -0.032933985047 | -3.177639136162 |
| N | -1.885008515394 | 1.210335827073 | 0.302416909082 |
| N | 0.946247296662 | -1.521220741972 | 0.082269246661 |
| N | -1.619638780332 | 1.518130872385 | 2.486411079226 |
| C | 0.481795098998 | 0.891791364420 | -0.549975138099 |
| C | 0.538849627169 | -0.592428789944 | -0.988641397319 |
| C | -0.517824708954 | 1.164402879023 | 0.525623112028 |
| C | 1.498010004725 | -0.734476635026 | -2.195578195674 |
| C | -0.391643079135 | 1.352683743720 | 1.883440613960 |
| C | -2.492717942002 | 1.431909651427 | 1.508596798811 |
| H | 1.474486414858 | 1.185721684062 | -0.185713772077 |
| H | 0.295722832219 | 1.493239835993 | -1.446460400044 |
| H | -0.450083275425 | -0.886455019394 | -1.361189829327 |
| H | -2.342034084648 | 1.155109036904 | -0.597214049146 |
| H | 0.524848939881 | 1.396576380078 | 2.458760087514 |
| H | 1.263238026391 | -1.011500093691 | 0.905019943495 |
| H | 0.170630194619 | -2.101723843331 | 0.388252183731 |
| H | -3.566486537612 | 1.523454704149 | 1.606153596849 |
| H | 2.227073092768 | -2.074145182080 | -1.146294569548 |

TS57

| | | | |
|---|---|---|---|
| O | 2.491579732903 | -1.675691458888 | -2.145676823901 |
| O | 1.035404981338 | -0.323428912464 | -3.190481089193 |
| N | -1.566916914504 | 2.107570732241 | 0.453237290149 |
| N | 1.689286063863 | -1.123518512032 | 0.251505858880 |
| N | -1.971766586015 | 1.031046345722 | 2.349916148924 |
| C | 0.268793360155 | 0.799891864147 | -0.662760613709 |
| C | 0.766773258193 | -0.647195656297 | -0.795953807412 |
| C | -0.696088640133 | 1.030500724016 | 0.461835288281 |
| C | 1.442156764949 | -0.846558450063 | -2.178451415512 |
| C | -0.972041606604 | 0.390595052992 | 1.650152506320 |
| C | -2.305728276690 | 2.058100575929 | 1.602756082950 |
| H | 1.146155425851 | 1.460761193176 | -0.567829629995 |
| H | -0.195196862605 | 1.061830147302 | -1.620482885330 |
| H | -0.102196499331 | -1.317336573430 | -0.794051124986 |
| H | -1.652792083513 | 2.789107057025 | -0.287831330386 |
| H | -0.509931491326 | -0.500097582436 | 2.053608053322 |
| H | 2.236106744472 | -0.353794893654 | 0.634344488155 |
| H | 1.193186272437 | -1.553711892192 | 1.026228402883 |

| | | | |
|---|---|---|---|
| H | -3.066234617762 | 2.792055914268 | 1.834132409584 |
| H | 2.601755579441 | -1.900041915195 | -1.185250837022 |

TS58

| | | | |
|---|---|---|---|
| O | 2.670961766728 | -1.126493887730 | -1.897808429353 |
| O | 1.041166894473 | -0.533244629139 | -3.362508780284 |
| N | -1.852429956063 | 1.373197836427 | 0.303774373291 |
| N | 0.810087082084 | -1.599102954883 | 0.002455011810 |
| N | -1.579017468902 | 1.441973080465 | 2.506722235697 |
| C | 0.467499824808 | 0.872065279076 | -0.579392151214 |
| C | 0.450331567342 | -0.629922005254 | -1.027135610970 |
| C | -0.500694666726 | 1.155343410937 | 0.521616290205 |
| C | 1.368709461139 | -0.784267704233 | -2.233321557384 |
| C | -0.370301094478 | 1.197264370851 | 1.892325796881 |
| C | -2.445713227842 | 1.544559157595 | 1.524204638733 |
| H | 1.479914860989 | 1.120479965907 | -0.239402352304 |
| H | 0.261418842344 | 1.504157713887 | -1.453350685261 |
| H | -0.559493407841 | -0.865431825779 | -1.380484432633 |
| H | -2.305613079022 | 1.433758014774 | -0.597383008486 |
| H | 0.538391400982 | 1.081621085966 | 2.469302892765 |
| H | 1.742033714463 | -1.379274783438 | 0.351995704217 |
| H | 0.171169001032 | -1.501343405575 | 0.790937312032 |
| H | -3.504115818017 | 1.743396493101 | 1.629440578196 |
| H | 2.777565685261 | -2.090534226432 | -1.953114687015 |

TS59

| | | | |
|---|---|---|---|
| O | 2.523199144596 | -1.439501098789 | -1.904605641616 |
| O | 1.026433373018 | -0.449488614292 | -3.264969279717 |
| N | -1.827192491226 | 1.580295210496 | 0.273658519703 |
| N | 1.061939801100 | -1.336095289119 | 0.228601056225 |
| N | -1.635125026496 | 1.070624552808 | 2.446770295103 |
| C | 0.467005354502 | 0.918087909603 | -0.626831236383 |
| C | 0.492001064127 | -0.602504538965 | -0.908818065676 |
| C | -0.481299911950 | 1.256719158297 | 0.495247062947 |
| C | 1.364510410888 | -0.828529724646 | -2.166337360898 |
| C | -0.347164291641 | 0.845772673494 | 1.886868797115 |
| C | -2.439384090199 | 1.444012049069 | 1.484159261270 |
| H | 1.478585025289 | 1.228026911994 | -0.342428259366 |
| H | 0.210060478079 | 1.443207611021 | -1.551529271382 |
| H | -0.523993565019 | -0.928520274197 | -1.169424606341 |
| H | -2.247185540888 | 1.930156994961 | -0.578223964902 |
| H | 0.074196517267 | 2.039449602222 | 1.296792115293 |
| H | 0.929596529053 | -0.808528063138 | 1.101073952384 |
| H | 0.597010793941 | -2.233186642852 | 0.350438544214 |
| H | -3.498332743861 | 1.657048397978 | 1.599500002809 |
| H | 2.490853263756 | -1.606211084096 | -0.922040537700 |

TS60

| | | | |
|---|---|---|---|
| O | 2.355102812846 | -1.674185556537 | -2.111206481285 |
| O | 1.582688764717 | 0.187665785671 | -3.106827857041 |
| N | -1.505999363892 | 0.323740622791 | 0.974866729044 |
| N | 0.533526617552 | -1.775386950098 | -0.266757716438 |
| N | -1.802917785041 | 2.223990894418 | 2.082394236217 |
| C | 0.690441520715 | 0.725471667016 | -0.287114753526 |
| C | 0.452843749518 | -0.558139604017 | -1.101413559145 |
| C | -0.425850713431 | 1.127323880348 | 0.639552312666 |
| C | 1.519275086407 | -0.635547125483 | -2.224606327411 |
| C | -0.642015263379 | 2.289572110628 | 1.343928860644 |
| C | -2.299675804238 | 1.034088569635 | 1.835906153557 |
| H | 1.628664433073 | 0.599820307441 | 0.274794648356 |
| H | 0.872832287412 | 1.531090193500 | -1.005126278532 |
| H | -0.515005903569 | -0.478996938794 | -1.612908763966 |
| H | -1.693834014416 | -0.600586101981 | 0.616566141463 |
| H | -0.020654170735 | 3.175652902392 | 1.355334017914 |
| H | 0.517397855190 | -1.576943255186 | 0.729386475262 |
| H | -0.192946589643 | -2.453255716794 | -0.479432669158 |
| H | -3.217971548681 | 0.629666934073 | 2.240620360120 |
| H | 2.052816283813 | -2.149938812859 | -1.295034385627 |

TS61

| | | | |
|---|---|---|---|
| O | 2.223420221638 | -1.898617169550 | -2.158083639391 |
| O | 1.618416663492 | 0.066777789916 | -3.058395843954 |
| N | -1.863311783512 | 1.201121550230 | 0.220332453499 |
| N | 1.051109285011 | -1.486535184960 | 0.123224039978 |
| N | -1.555684524380 | 1.482948630641 | 2.515070373115 |
| C | 0.500787179565 | 0.921888454018 | -0.551712680232 |
| C | 0.612095387107 | -0.567302469256 | -0.940759284309 |
| C | -0.530152877624 | 1.186228856862 | 0.504320215205 |
| C | 1.540278774415 | -0.744149764524 | -2.166129465252 |
| C | -0.371455000856 | 1.397468768361 | 1.881154485084 |
| C | -2.459963668693 | 1.348966915629 | 1.528922216423 |
| H | 1.479663586955 | 1.272758441605 | -0.199004115021 |
| H | 0.274132512045 | 1.486623635866 | -1.460227105781 |
| H | -0.376999940526 | -0.910843842996 | -1.271211018771 |

| | | | |
|---|---|---|---|
| H | -2.461835244893 | 0.246485640416 | 0.847032112940 |
| H | 0.561374750012 | 1.476931620424 | 2.430147268528 |
| H | 1.767472503282 | -1.049639890890 | 0.702779421824 |
| H | 0.284292031223 | -1.737352450038 | 0.741873677465 |
| H | -3.506551682278 | 1.618418550063 | 1.617481480344 |
| H | 1.988004599831 | -2.329394013334 | -1.298791457779 |

TS62

| | | | |
|---|---|---|---|
| O | 2.258302640349 | -1.335436635618 | -2.432821968389 |
| O | 0.529316225142 | 0.010124098270 | -2.948123448953 |
| N | -1.518878641799 | 1.417002196752 | 0.061714829479 |
| N | 1.429168506220 | -1.872850249863 | -0.019287457418 |
| N | -2.014457122584 | 1.614693523254 | 2.217827794099 |
| C | 0.844438199333 | 0.572557702721 | 0.030554807535 |
| C | 0.666041044396 | -0.803798967337 | -0.687632545151 |
| C | -0.381861492245 | 1.028669085558 | 0.750156661341 |
| C | 1.132568671410 | -0.671068680394 | -2.148772267698 |
| C | -0.720592975032 | 1.160818202481 | 2.076119645294 |
| C | -2.462901078896 | 1.757958437957 | 0.990230294537 |
| H | 1.681025790824 | 0.506037612144 | 0.735591584520 |
| H | 1.130896333676 | 1.324823206907 | -0.713545647283 |
| H | -0.405818771020 | -1.028965990087 | -0.737447047036 |
| H | -1.607209056513 | 1.448461236560 | -0.946008772174 |
| H | -0.098808603499 | 0.961518363469 | 2.939730639360 |
| H | 1.532132193057 | -1.686038411541 | 0.975395141250 |
| H | 0.948269417519 | -2.766794518968 | -0.101000759712 |
| H | -3.450001276173 | 2.102699391832 | 0.711971388445 |
| H | 2.512593348401 | -1.773948342009 | -1.580412759514 |

TS63

| | | | |
|---|---|---|---|
| O | 2.019108398131 | -1.926307219061 | -2.335206376407 |
| O | 1.737223247568 | 0.233319777238 | -2.964900638643 |
| N | -1.912190698194 | 1.266108809764 | 0.276639372412 |
| N | 1.142453682020 | -1.319004664957 | 0.142004502825 |
| N | -1.715434259399 | 1.453276234177 | 2.480859720402 |
| C | 0.476000002931 | 0.963881017574 | -0.522587609365 |
| C | 0.603727744527 | -0.512600763120 | -0.955045527506 |
| C | -0.553679073778 | 1.182510792910 | 0.540377588244 |
| C | 1.521743242760 | -0.642877187308 | -2.170013883781 |
| C | -0.470125822848 | 1.297829704222 | 1.910487752339 |
| C | -2.556737661190 | 1.432873029732 | 1.471979241647 |
| H | 1.451108882299 | 1.288893232504 | -0.144391449536 |
| H | 0.265137469694 | 1.567688699925 | -1.411537363589 |
| H | -0.391315771040 | -0.856473178674 | -1.304664781603 |
| H | -2.340380411918 | 1.249003936997 | -0.638336738607 |
| H | 0.428026784068 | 1.292618018800 | 2.514838856772 |
| H | 0.593716604540 | -1.136034096348 | 0.981653508423 |
| H | 1.036559903428 | -2.306570350978 | -0.077964523192 |
| H | -3.631887046554 | 1.535241798073 | 1.539381556940 |
| H | 2.890897311691 | -1.989240111170 | -1.910942487353 |

TS64

| | | | |
|---|---|---|---|
| O | 2.645940110620 | -1.172286814456 | -2.278163358896 |
| O | 0.778809253395 | -1.006024903986 | -3.523457725502 |
| N | -1.477803513324 | 1.720598873053 | 0.303853142142 |
| N | 1.422140042020 | -1.244404273568 | 0.008222361275 |
| N | -2.001702646238 | 1.570118859724 | 2.557311263353 |
| C | 0.165677183662 | 0.636400705240 | -0.977891336077 |
| C | 0.558691414442 | -0.855944840640 | -1.116385353394 |
| C | -0.450933909516 | 0.908162890004 | 0.364586043265 |
| C | 1.321905424895 | -1.034861784899 | -2.443166984791 |
| C | -1.458743254492 | 0.676946961554 | 3.222260230603 |
| C | -2.269382034943 | 2.100994938619 | 1.430224017894 |
| H | 1.097114235300 | 1.224899101567 | -1.015533360260 |
| H | -0.437023126222 | 0.924016243823 | -1.856803418932 |
| H | -0.360825705663 | -1.451020995451 | -1.188571920389 |
| H | -1.817158653347 | 2.130458991685 | -0.576102578571 |
| H | -0.611291218809 | 0.967989586838 | 3.857135501688 |
| H | 1.123047732455 | -0.712948774087 | 0.829094860749 |
| H | 1.301215227054 | -2.231671770165 | 0.226731414831 |
| H | -3.074093077446 | 2.806067489389 | 1.277015899899 |
| H | 2.770791157058 | -1.171589535522 | -1.291775556179 |

TS65

| | | | |
|---|---|---|---|
| O | 2.543910685600 | -1.743117349330 | -1.368995699426 |
| O | 2.019750529206 | -0.027192449699 | -2.718541612980 |
| N | -2.068029945265 | 0.825521497449 | 0.662060270243 |
| N | 0.393340589600 | -1.587355851382 | 0.063530302839 |
| N | -0.979414657639 | 1.854680699178 | 2.300520372797 |
| C | -0.327866464452 | 0.593564116452 | -1.169303416064 |
| C | 0.303994541597 | -0.847332010358 | -1.204349032158 |
| C | -0.787188012238 | 1.047638148719 | 0.178380716222 |
| C | 1.714906204646 | -0.805966761916 | -1.845287851709 |
| C | -0.141431975648 | 1.677628235950 | 1.220248979773 |

| | | | |
|---|---|---|---|
| C | -2.129210082489 | 1.339593720239 | 1.928773111544 |
| H | 0.417704006373 | 1.293456375392 | -1.556202387763 |
| H | -1.163732144433 | 0.634288079836 | -1.877343589905 |
| H | -0.286078927377 | -1.469379763315 | -1.888211068441 |
| H | -2.837019552738 | 0.430857239176 | 0.138718590339 |
| H | 0.882170031885 | 2.029864747311 | 1.241252745582 |
| H | 0.565833503519 | -0.939789960746 | 0.833704790188 |
| H | -0.478840010591 | -2.063782844539 | 0.278448480896 |
| H | -3.033223808741 | 1.309307683353 | 2.522739065794 |
| H | 2.036607811817 | -2.175219088101 | -0.634503425105 |

TS66

| | | | |
|---|---|---|---|
| O | 0.523174661817 | 0.163515163460 | -2.711324782019 |
| O | 0.473190404854 | -1.946299905556 | -1.924328702710 |
| N | -0.478928125571 | 1.546483039152 | 0.888556894597 |
| N | 1.955972721791 | 1.162371447631 | -0.665024153637 |
| N | -2.586773336160 | 0.886173975757 | 1.131263125150 |
| C | 0.823008559259 | -0.603292756153 | 0.701117317861 |
| C | 1.608449593183 | -0.270750779509 | -0.599196531476 |
| C | -0.450435504593 | 0.163978193538 | 0.853410278268 |
| C | 0.816426777851 | -0.793908472027 | -1.796607249332 |
| C | -1.765250062416 | -0.213333237607 | 1.010886875014 |
| C | -1.780638668563 | 1.924203543335 | 1.044199062194 |
| H | 0.601363592828 | -1.674408283481 | 0.709897897644 |
| H | 1.500866323403 | -0.400964037155 | 1.541304832449 |
| H | 2.520578730683 | -0.881685151044 | -0.580450093154 |
| H | 0.338087185706 | 2.111823419372 | 0.688398623769 |
| H | -2.162295594794 | -1.219547886297 | 1.050197602982 |
| H | 2.965422702080 | 1.277565733342 | -0.689611662611 |
| H | 1.593822573608 | 1.566712236469 | -1.524593867928 |
| H | -2.081742438641 | 2.962642242720 | 1.090833658751 |
| H | -0.007903262295 | -0.273078681334 | -3.403978355289 |

TS67

| | | | |
|---|---|---|---|
| O | 0.386104099989 | 0.129308095668 | -2.554138923195 |
| O | 0.533047154333 | -2.008686890866 | -1.860619122034 |
| N | -0.489940497271 | 1.582570469272 | 0.868854232067 |
| N | 2.116070752853 | 1.034109168868 | -0.789166270127 |
| N | -2.588069823481 | 0.865869206724 | 0.993563471989 |
| C | 0.871750215168 | -0.548150238793 | 0.760437705708 |
| C | 1.670678788059 | -0.314591941158 | -0.566332075634 |
| C | -0.417654467131 | 0.199593598055 | 0.850323773790 |
| C | 0.825292048337 | -0.843067941030 | -1.720320089176 |
| C | -1.729467662816 | -0.210188510449 | 0.932611277928 |
| C | -1.810587990594 | 1.925622520857 | 0.946371159280 |
| H | 0.665911127962 | -1.619154334181 | 0.855869306080 |
| H | 1.542998580849 | -0.274492866263 | 1.586894767922 |
| H | 2.539320719712 | -0.986033128322 | -0.521567529165 |
| H | 0.303590952149 | 2.194489156967 | 0.743466877225 |
| H | -2.099132532782 | -1.227126049238 | 0.956659954074 |
| H | 2.869888359788 | 1.414884506701 | -0.243858949671 |
| H | 1.592083969049 | 1.648963422633 | -1.386378738681 |
| H | -2.141205045737 | 2.955818647583 | 0.969187129379 |
| H | -0.186547722889 | -0.313154350125 | -3.208892414347 |

TS68

| | | | |
|---|---|---|---|
| O | 0.554507417483 | 0.670253588520 | -2.301820171921 |
| O | 0.001663618202 | -1.490435373850 | -1.994566213378 |
| N | -0.910965301582 | 1.447688320820 | 0.219924655210 |
| N | 2.817033865839 | 0.324372263147 | -0.848675676133 |
| N | -2.623471102740 | 0.528785830894 | 1.296634008100 |
| C | 1.048215907848 | -0.040155855420 | 0.833126164832 |
| C | 1.669588964260 | -0.532222429554 | -0.528856968996 |
| C | -0.400117545109 | 0.331183240018 | 0.862622454560 |
| C | 0.658144163514 | -0.528839915725 | -1.669491413633 |
| C | -1.481694907323 | -0.207007520442 | 1.521100530406 |
| C | -2.245353229214 | 1.509547933771 | 0.505796814825 |
| H | 1.202340822530 | -0.818831242797 | 1.585960571960 |
| H | 1.649323776721 | 0.826369146860 | 1.138025538503 |
| H | 1.921677465956 | -1.596854087780 | -0.415332856314 |
| H | -0.394887241647 | 2.036484344097 | -0.419497538619 |
| H | -1.495127035468 | -1.086852123359 | 2.151519536739 |
| H | 3.522663145431 | 0.212023297307 | -0.121803611885 |
| H | 3.254332094500 | 0.024044765890 | -1.719008273222 |
| H | -2.884898217848 | 2.289710597729 | 0.114036507477 |
| H | -0.126811551310 | 0.536495719219 | -2.988967969162 |

TS69

| | | | |
|---|---|---|---|
| O | 1.199400294245 | -0.078797935864 | -3.036086749776 |
| O | -0.115589673420 | -1.481056549954 | -1.839604313081 |
| N | -0.630164281436 | 1.481509700595 | 0.638969305107 |
| N | 1.899512765511 | 1.189040246910 | -0.696404972287 |
| N | -2.577507374920 | 0.767004890708 | 1.436900682015 |
| C | 0.838264188280 | -0.577795161358 | 0.697476390100 |

| | | | |
|---|---|---|---|
| C | 1.573322237090 | -0.246842171099 | -0.617354541455 |
| C | -0.464887647958 | 0.133435884537 | 0.897030591061 |
| C | 0.782435976210 | -0.688757350139 | -1.851955182227 |
| C | -1.681160354140 | -0.277553930389 | 1.392953836338 |
| C | -1.914357410894 | 1.803797308925 | 0.967207190477 |
| H | 0.658184628439 | -1.656112945079 | 0.735304745667 |
| H | 1.536753102576 | -0.348499153265 | 1.517506326621 |
| H | 2.487005126292 | -0.874703987322 | -0.645270813435 |
| H | 0.085764451817 | 2.032275962132 | 0.174750399235 |
| H | -1.960149675714 | -1.271529226434 | 1.718336660699 |
| H | 2.704498378222 | 1.391005969514 | -0.106369487488 |
| H | 2.147652517682 | 1.427442737786 | -1.653977391122 |
| H | -2.311107559722 | 2.802795593323 | 0.842142461914 |
| H | 1.995813594711 | -0.520363062221 | -3.375129600406 |

TS70

| | | | |
|---|---|---|---|
| O | 0.031720386785 | 0.464540935701 | -2.321451439664 |
| O | 1.113916522784 | -1.905400955031 | -2.528932984992 |
| N | -0.431634670242 | 1.616565261581 | 0.793242317259 |
| N | 1.909516691796 | 0.957986239570 | -0.753376293225 |
| N | -2.569038085257 | 1.004964054704 | 0.796272786235 |
| C | 0.835012231847 | -0.560435558750 | 0.859340207758 |
| C | 1.748502772558 | -0.342402242449 | -0.357243493867 |
| C | -0.430780610393 | 0.233557596162 | 0.827354502357 |
| C | 0.941423068060 | -1.127400670686 | -1.691920839556 |
| C | -1.764480627959 | -0.114499242537 | 0.836595554671 |
| C | -1.734221171982 | 2.023215240385 | 0.761155427155 |
| H | 0.593491835342 | -1.624637187974 | 0.961530458191 |
| H | 1.437013065635 | -0.281409161010 | 1.735956570119 |
| H | 2.635691463166 | -0.990835427311 | -0.299123723081 |
| H | 0.407177680489 | 2.154724036134 | 0.603679558585 |
| H | -2.188718038769 | -1.109029288331 | 0.892681233931 |
| H | 2.759107821943 | 1.055524658447 | -1.311424625364 |
| H | 0.741615866598 | 0.985115685797 | -1.669564818274 |
| H | -2.014454654118 | 3.067144830338 | 0.712454587995 |
| H | -0.807654228555 | 0.421352333150 | -1.827352695118 |

TS71

| | | | |
|---|---|---|---|
| O | 1.742237761579 | -0.246631349502 | -2.675765794245 |
| O | 0.052406514251 | -1.550816191644 | -1.935473966316 |
| N | -0.113040446048 | 1.211477424558 | 1.755418296173 |
| N | 1.334502190645 | 1.812589740740 | -0.613284631990 |
| N | -2.277446299973 | 1.195834113166 | 0.960614476685 |
| C | 0.741656494986 | -0.977483365262 | 0.828786823834 |
| C | 1.442164212616 | -0.413068977867 | -0.342698323585 |
| C | -0.331293375728 | 0.068557354668 | 1.019865521503 |
| C | 0.970438092298 | -0.791245322372 | -1.700892584993 |
| C | -1.652583684972 | 0.105394441813 | 0.498121695197 |
| C | -1.306531974698 | 1.815986028091 | 1.701554504418 |
| H | 0.304575554516 | -1.951999861332 | 0.591433451849 |
| H | 1.387950419852 | -1.051896606912 | 1.705711319940 |
| H | 2.482778471963 | -0.126376753104 | -0.278768972178 |
| H | 1.114725505830 | 2.047763849539 | 0.361255718734 |
| H | -2.143147772876 | -0.631405884622 | -0.127490392362 |
| H | 2.162172540536 | 2.293078316025 | -0.962049868091 |
| H | 0.529885325161 | 2.035348635180 | -1.194794211215 |
| H | -1.503804227399 | 2.745574224330 | 2.225676093728 |
| H | 1.403349939142 | -0.604636964923 | -3.517823103040 |

**Optimized EQ13**

| | | | |
|---|---|---|---|
| O | 1.621563226662 | -1.498340084283 | -2.608217045803 |
| O | 0.128402313749 | 0.121576333312 | -2.334315304928 |
| N | -1.277823923910 | 1.256767957009 | -0.182240495171 |
| N | 2.443699379990 | -1.522174443504 | -0.163674321511 |
| N | -2.200202515142 | 1.643933915543 | 1.789825266597 |
| C | 1.073995976956 | 0.535271345354 | 0.410787332104 |
| C | 1.172891259128 | -0.808261488149 | -0.344794598121 |
| C | -0.305037119117 | 0.984713834942 | 0.751187284970 |
| C | 0.913568747817 | -0.660047404188 | -1.856879650591 |
| C | -0.900552053067 | 1.233908345729 | 1.962837505211 |
| C | -2.394326444347 | 1.642251483166 | 0.490965663573 |
| H | 1.609680718717 | 0.416200958746 | 1.353826482469 |
| H | 1.606319060816 | 1.301438836482 | -0.163301518108 |
| H | 0.378091329243 | -1.466098815344 | 0.018028396390 |
| H | -1.145553792097 | 1.107653924054 | -1.174496519690 |
| H | -0.459746833397 | 1.148240647331 | 2.943327523802 |
| H | 3.224302713933 | -0.876237749965 | -0.113266727358 |
| H | 2.444290706685 | -2.073489041984 | 0.684151166549 |
| H | -3.311228417149 | 1.905482566085 | -0.011072907781 |
| H | 2.212278347003 | -1.974780559905 | -1.978407466503 |

# Optimized Tautomer

| | | | |
|---|---|---|---|
| O | 2.695544538764 | -1.689354712189 | -2.050156335611 |
| O | 2.560218907287 | 0.492841822357 | -2.499528505651 |

| | | | |
|---|---|---|---|
| N | -1.452446445665 | 0.300635171647 | 0.416491324991 |
| N | 0.978811887813 | -1.644180815919 | -0.096701040587 |
| N | -1.762807256752 | 1.477946186682 | 2.252241634584 |
| C | 0.919802018279 | 0.888261827036 | -0.167862151748 |
| C | 0.974190405110 | -0.436699910279 | -0.929796982593 |
| C | -0.288065814310 | 0.980976013756 | 0.710577703176 |
| C | 2.161812780507 | -0.470046830236 | -1.901458031225 |
| C | -0.463449272841 | 1.711894541243 | 1.855936842968 |
| C | -2.313307198936 | 0.620935076046 | 1.353628635369 |
| H | 1.827486595135 | 1.007190014835 | 0.430990072157 |
| H | 0.931687384185 | 1.697311447758 | -0.900672913622 |
| H | 0.082031329682 | -0.496622607033 | -1.557976152474 |
| H | -2.215592584275 | 1.867344225818 | 3.061025571508 |
| H | 0.193820875482 | 2.365178763701 | 2.402034719222 |
| H | 1.522037516661 | -1.496577077182 | 0.747716122064 |
| H | 0.032804065554 | -1.861279476122 | 0.191933718958 |
| H | -3.327215197685 | 0.264034429810 | 1.432774615654 |
| H | 2.199222010562 | -2.263485895351 | -1.422834091350 |

# Tautomerization Pathways

EQ0

| | | | |
|---|---|---|---|
| O | 2.695544538764 | -1.689354712189 | -2.050156335611 |
| O | 2.560218907287 | 0.492841822357 | -2.499528505651 |
| N | -1.452446445665 | 0.300635171647 | 0.416491324991 |
| N | 0.978811887813 | -1.644180815919 | -0.096701040587 |
| N | -1.762807256752 | 1.477946186682 | 2.252241634584 |
| C | 0.919802018279 | 0.888261827036 | -0.167862151748 |
| C | 0.974190405110 | -0.436699910279 | -0.929796982593 |
| C | -0.288065814310 | 0.980976013756 | 0.710577703176 |
| C | 2.161812780507 | -0.470046830236 | -1.901458031225 |
| C | -0.463449272841 | 1.711894541243 | 1.855936842968 |
| C | -2.313307198936 | 0.620935076046 | 1.353628635369 |
| H | 1.827486595135 | 1.007190014835 | 0.430990072157 |
| H | 0.931687384185 | 1.697311447758 | -0.900672913622 |
| H | 0.082031329682 | -0.496622607033 | -1.557976152474 |
| H | -2.215592584275 | 1.867344225818 | 3.061025571508 |
| H | 0.193820875482 | 2.365178763701 | 2.402034719222 |
| H | 1.522037516661 | -1.496577077182 | 0.747716122064 |
| H | 0.032804065554 | -1.861279476122 | 0.191933718958 |
| H | -3.327215197685 | 0.264034429810 | 1.432774615654 |
| H | 2.199222010562 | -2.263485895351 | -1.422834091350 |

EQ1

| | | | |
|---|---|---|---|
| O | 2.498183846241 | -1.552629162359 | -2.396642743763 |
| O | 2.837425767968 | 0.630927651785 | -2.084771301553 |
| N | -1.060237763240 | -0.132231203638 | 1.274906170408 |
| N | 0.758294297391 | -1.794786742420 | -0.482990763668 |
| N | -1.976550375722 | 1.803597574801 | 1.790456728836 |
| C | 1.105354473581 | 0.617311085700 | 0.222706580169 |
| C | 0.994933611201 | -0.410688392918 | -0.908103570152 |
| C | -0.205475306853 | 0.883293094806 | 0.887074019033 |
| C | 2.216117497885 | -0.367701430056 | -1.840201763007 |
| C | -0.766215318497 | 2.093131564765 | 1.198928587502 |
| C | -2.106329565816 | 0.451659394402 | 1.809404608920 |
| H | 1.835029827926 | 0.248184442132 | 0.952571548408 |
| H | 1.510240558586 | 1.544299290551 | -0.181778159631 |
| H | 0.150067339439 | -0.129028849251 | -1.545492246137 |
| H | -2.641531845446 | 2.471994677398 | 2.139285053522 |
| H | -0.422784597058 | 3.102148049224 | 1.055109893620 |
| H | 1.380099690720 | -2.023283992852 | 0.287765415851 |
| H | -0.179538501824 | -1.865736647526 | -0.101997029753 |
| H | -2.971213377598 | -0.045677927777 | 2.217207681588 |
| H | 1.860543915713 | -2.185774495761 | -1.993919195381 |

EQ2

| | | | |
|---|---|---|---|
| O | 2.670068067374 | -1.622940639656 | -2.206687133559 |
| O | 2.724551540264 | 0.608312277670 | -2.246772165951 |
| N | -1.300414864663 | 0.109850161886 | 0.670188115592 |
| N | 0.913507994621 | -1.771001282392 | -0.284594056601 |
| N | -1.799508552710 | 1.878061137656 | 2.164116979755 |
| C | 0.993232907474 | 0.744423697912 | 0.023120244376 |
| C | 1.007259225886 | -0.455038248792 | -0.920124484894 |
| C | -0.294014572455 | 0.901972547959 | 0.760244251482 |
| C | 2.230525145363 | -0.411860507041 | -1.849154281050 |
| C | -0.615144132306 | 2.002842271078 | 1.700508542998 |
| C | -2.317171110413 | 0.671004589426 | 1.541935512658 |
| H | 1.814626625878 | 0.665198887982 | 0.745408987765 |
| H | 1.200775419918 | 1.651841079066 | -0.549567066793 |
| H | 0.142155194628 | -0.369995756330 | -1.584233712619 |
| H | -3.219803062249 | 0.905734331538 | 0.969588218830 |
| H | 0.041269461397 | 2.820856313934 | 1.975308238302 |
| H | 1.492052089152 | -1.811686932417 | 0.548462003690 |
| H | -0.042705911450 | -1.935429532013 | 0.007149350758 |
| H | -2.601458629380 | -0.052466516808 | 2.312107307777 |

| | | | |
|---|---|---|---|
| H | 2.111861963065 | -2.261566545626 | -1.707910128321 |

EQ3

| | | | |
|---|---|---|---|
| O | 2.879968548164 | -1.618723667185 | -1.929558003031 |
| O | 2.189422101700 | 0.341071636096 | -2.745861970351 |
| N | -1.610368721941 | 1.114031224952 | 0.214692660047 |
| N | 1.374623326875 | -1.544313945561 | 0.196122022951 |
| N | -1.749078138810 | 1.017712943034 | 2.416505852813 |
| C | 0.856892409527 | 0.898081873148 | -0.245673886015 |
| C | 1.048689319460 | -0.524852465634 | -0.802941707249 |
| C | -0.310481246057 | 1.004109409947 | 0.668481550672 |
| C | 2.100785669557 | -0.533029566418 | -1.927009637284 |
| C | -0.429546534458 | 0.941625491199 | 2.032568524814 |
| C | -2.432037837672 | 1.124507647747 | 1.305073046514 |
| H | 1.761701201627 | 1.184583395160 | 0.296091849777 |
| H | 0.771163519491 | 1.586759090326 | -1.087426820244 |
| H | 0.120495204822 | -0.836744518637 | -1.290802134220 |
| H | -1.891578131848 | 1.227443666252 | -0.744748639462 |
| H | 0.364097554036 | 0.858980730444 | 2.757596431401 |
| H | 1.952539043645 | -1.156572711305 | 0.934459508738 |
| H | 0.541930420125 | -1.911542303739 | 0.637449337288 |
| H | -3.503011166694 | 1.216240458015 | 1.222633358911 |
| H | 2.592956963358 | -2.147746440598 | -1.151151628243 |

EQ4

| | | | |
|---|---|---|---|
| O | 2.242436562256 | -1.278820800769 | -2.462781210107 |
| O | 0.805380359711 | 0.386945703232 | -2.165032530777 |
| N | -0.886987537404 | 1.277615537851 | -0.105458149993 |
| N | 2.638075511732 | -1.743359471323 | 0.043253229373 |
| N | -2.108744344556 | 1.422471077233 | 1.731950168048 |
| C | 1.293090990386 | 0.297456483094 | 0.725381704710 |
| C | 1.452424810885 | -0.916245401967 | -0.216624802072 |
| C | -0.100004032587 | 0.791429693038 | 0.912328965456 |
| C | 1.459606435403 | -0.516231866645 | -1.705056747886 |
| C | -0.878449711219 | 0.893782172816 | 2.038556385475 |
| C | -2.080884547361 | 1.637020900854 | 0.436611320490 |
| H | 1.655463222320 | -0.006590551051 | 1.708580460021 |
| H | 1.951433546132 | 1.101029307360 | 0.377793675526 |
| H | 0.576627941254 | -1.560522814009 | -0.098402793882 |
| H | -0.596681527509 | 1.275002173088 | -1.074990469911 |
| H | -0.614031597624 | 0.624026758786 | 3.048669066481 |
| H | 3.429506595452 | -1.173715388844 | 0.322988639951 |
| H | 2.468705204713 | -2.417931414815 | 0.777421806896 |
| H | -2.886047421705 | 2.043331947666 | -0.153864215920 |
| H | 2.694484490153 | -1.890379970278 | -1.834910192400 |

**Reoptimized EQ0-EQ4**

EQ0

| | | | |
|---|---|---|---|
| O | 2.695544538764 | -1.689354712189 | -2.050156335611 |
| O | 2.560218907287 | 0.492841822357 | -2.499528505651 |
| N | -1.452446445665 | 0.300635171647 | 0.416491324991 |
| N | 0.978811887813 | -1.644180815919 | -0.096701040587 |
| N | -1.762807256752 | 1.477946186682 | 2.252241634584 |
| C | 0.919802018279 | 0.888261827036 | -0.167862151748 |
| C | 0.974190405110 | -0.436699910279 | -0.929796982593 |
| C | -0.288065814310 | 0.980976013756 | 0.710577703176 |
| C | 2.161812780507 | -0.470046830236 | -1.901458031225 |
| C | -0.463449272841 | 1.711894541243 | 1.855936842968 |
| C | -2.313307198936 | 0.620935076046 | 1.353628635369 |
| H | 1.827486595135 | 1.007190014835 | 0.430990072157 |
| H | 0.931687384185 | 1.697311447758 | -0.900672913622 |
| H | 0.082031329682 | -0.496622607033 | -1.557976152474 |
| H | -2.215592584275 | 1.867344225818 | 3.061025571508 |
| H | 0.193820875482 | 2.365178763701 | 2.402034719222 |
| H | 1.522037516661 | -1.496577077182 | 0.747716122064 |
| H | 0.032804065554 | -1.861279476122 | 0.191933718958 |
| H | -3.327215197685 | 0.264034429810 | 1.432774615654 |
| H | 2.199222010562 | -2.263485895351 | -1.422834091350 |

EQ1

| | | | |
|---|---|---|---|
| O | 2.498183846241 | -1.552629162359 | -2.396642743763 |
| O | 2.837425767968 | 0.630927651785 | -2.084771301553 |
| N | -1.060237763240 | -0.132231203638 | 1.274906170408 |
| N | 0.758294297391 | -1.794786742420 | -0.482990763668 |
| N | -1.976550375722 | 1.803597574801 | 1.790456728836 |
| C | 1.105354473581 | 0.617311085700 | 0.222706580169 |
| C | 0.994933611201 | -0.410688392918 | -0.908103570152 |
| C | -0.205475306853 | 0.883293094806 | 0.887074019033 |
| C | 2.216117497885 | -0.367701430056 | -1.840201763007 |
| C | -0.766215318497 | 2.093131564765 | 1.198928587502 |
| C | -2.106329565816 | 0.451659394402 | 1.809404608920 |
| H | 1.835029827926 | 0.248184442132 | 0.952571548408 |
| H | 1.510240558586 | 1.544299290551 | -0.181778159631 |
| H | 0.150067339439 | -0.129028849251 | -1.545492246137 |

| | | | |
|---|---|---|---|
| H | -2.641531845446 | 2.471994677398 | 2.139285053522 |
| H | -0.422784597058 | 3.102148049224 | 1.055109893620 |
| H | 1.380099690720 | -2.023283992852 | 0.287765415851 |
| H | -0.179538501824 | -1.865736647526 | -0.101997029753 |
| H | -2.971213377598 | -0.045677927777 | 2.217207681588 |
| H | 1.860543915713 | -2.185774495761 | -1.993919195381 |

EQ2

| | | | |
|---|---|---|---|
| O | 2.670068067374 | -1.622940639656 | -2.206687133559 |
| O | 2.724551540264 | 0.608312277670 | -2.246772165951 |
| N | -1.300414864663 | 0.109850161886 | 0.670188115592 |
| N | 0.913507994621 | -1.771001282392 | -0.284594056601 |
| N | -1.799508552710 | 1.878061137656 | 2.164116979755 |
| C | 0.993232907474 | 0.744423697912 | 0.023120244376 |
| C | 1.007259225886 | -0.455038248792 | -0.920124484894 |
| C | -0.294014572455 | 0.901972547959 | 0.760244251482 |
| C | 2.230525145363 | -0.411860507041 | -1.849154281050 |
| C | -0.615144132306 | 2.002842271078 | 1.700508542998 |
| C | -2.317171110413 | 0.671004589426 | 1.541935512658 |
| H | 1.814626625878 | 0.665198887982 | 0.745408987765 |
| H | 1.200775419918 | 1.651841079066 | -0.549567066793 |
| H | 0.142155194628 | -0.369995756330 | -1.584233712619 |
| H | -3.219803062249 | 0.905734331538 | 0.969588218830 |
| H | 0.041269461397 | 2.820856313934 | 1.975308238302 |
| H | 1.492052089152 | -1.811686932417 | 0.548462003690 |
| H | -0.042705911450 | -1.935429532013 | 0.007149350758 |
| H | -2.601458629380 | -0.052466516808 | 2.312107307777 |
| H | 2.111861963065 | -2.261566545626 | -1.707910128321 |

EQ3

| | | | |
|---|---|---|---|
| O | 2.879968548164 | -1.618723667185 | -1.929558003031 |
| O | 2.189422101700 | 0.341071636096 | -2.745861970351 |
| N | -1.610368721941 | 1.114031224952 | 0.214692660047 |
| N | 1.374623326875 | -1.544313945561 | 0.196122022951 |
| N | -1.749078138810 | 1.017712943034 | 2.416505852813 |
| C | 0.856892409527 | 0.898081873148 | -0.245673886015 |
| C | 1.048689319460 | -0.524852465634 | -0.802941707249 |
| C | -0.310481246057 | 1.004109409947 | 0.668481550672 |
| C | 2.100785669557 | -0.533029566418 | -1.927009637284 |
| C | -0.429546534458 | 0.941625491199 | 2.032568524814 |
| C | -2.432037837672 | 1.124507647747 | 1.305073046514 |
| H | 1.761701201627 | 1.184583395160 | 0.296091849777 |
| H | 0.771163519491 | 1.586759090326 | -1.087426820244 |
| H | 0.120495204822 | -0.836744518637 | -1.290802134220 |
| H | -1.891578131848 | 1.227443666252 | -0.744748639462 |
| H | 0.364097554036 | 0.858980730444 | 2.757596431401 |
| H | 1.952539043645 | -1.156572711305 | 0.934459508738 |
| H | 0.541930420125 | -1.911542303739 | 0.637449337288 |
| H | -3.503011166694 | 1.216240458015 | 1.222633358911 |
| H | 2.592956963358 | -2.147746440598 | -1.151151628243 |

EQ4

| | | | |
|---|---|---|---|
| O | 2.242436562256 | -1.278820800769 | -2.462781210107 |
| O | 0.805380359711 | 0.386945703232 | -2.165032530777 |
| N | -0.886987537404 | 1.277615537851 | -0.105458149993 |
| N | 2.638075511732 | -1.743359471323 | 0.043253229373 |
| N | -2.108744344556 | 1.422471077233 | 1.731950168048 |
| C | 1.293090990386 | 0.297456483094 | 0.725381704710 |
| C | 1.452424810885 | -0.916245401967 | -0.216624802072 |
| C | -0.100004032587 | 0.791429693038 | 0.912328965456 |
| C | 1.459606435403 | -0.516231866645 | -1.705056747886 |
| C | -0.878449711219 | 0.893782172816 | 2.038556385475 |
| C | -2.080884547361 | 1.637020900854 | 0.436611320490 |
| H | 1.655463222320 | -0.006590551051 | 1.708580460021 |
| H | 1.951433546132 | 1.101029307360 | 0.377793675526 |
| H | 0.576627941254 | -1.560522814009 | -0.098402793882 |
| H | -0.596681527509 | 1.275002173088 | -1.074990469911 |
| H | -0.614031597624 | 0.624026758786 | 3.048669066481 |
| H | 3.429506595452 | -1.173715388844 | 0.322988639951 |
| H | 2.468705204713 | -2.417931414815 | 0.777421806896 |
| H | -2.886047421705 | 2.043331947666 | -0.153864215920 |
| H | 2.694484490153 | -1.890379970278 | -1.834910192400 |

TS EQ0-EQ1

| | | | |
|---|---|---|---|
| O | 2.644553267157 | -1.686143193026 | -2.126011818419 |
| O | 2.602751180487 | 0.523238977933 | -2.440420100487 |
| N | -1.383133646154 | 0.181586806708 | 0.525029877958 |
| N | 0.942773335140 | -1.685838026152 | -0.161297611431 |
| N | -1.810890738356 | 1.568028784577 | 2.181162559141 |
| C | 0.965050601450 | 0.847274374905 | -0.097051004855 |
| C | 0.977603633928 | -0.435238151297 | -0.926960948653 |
| C | -0.271177957361 | 0.970246998123 | 0.741638187521 |
| C | 2.163884429064 | -0.456754636369 | -1.901058591524 |
| C | -0.521486397541 | 1.834902216920 | 1.775046337139 |

| | | | |
|---|---|---|---|
| C | -2.284899566928 | 0.564914492839 | 1.398601746115 |
| H | 1.855790689096 | 0.877749141067 | 0.538302090337 |
| H | 1.054538412852 | 1.692673405267 | -0.781497573540 |
| H | 0.085216353866 | -0.435797430993 | -1.558371746888 |
| H | -2.306535852040 | 2.031331000215 | 2.923157070117 |
| H | 0.078155958024 | 2.599142184984 | 2.236979922517 |
| H | 1.511667453421 | -1.608070566207 | 0.675761166986 |
| H | -0.008002357706 | -1.861985961475 | 0.141325959842 |
| H | -3.275450777965 | 0.155172353004 | 1.509443505018 |
| H | 2.125023880596 | -2.275672105247 | -1.532118592214 |

TS EQ1-EQ2

| | | | |
|---|---|---|---|
| O | 2.588829835947 | -1.650917718890 | -2.236128023886 |
| O | 2.527672849181 | 0.572397308195 | -2.424425381198 |
| N | -1.302306775914 | 0.049752757108 | 0.721182694832 |
| N | 0.966970544957 | -1.758867362083 | -0.201640313666 |
| N | -1.779617265077 | 1.693290205805 | 2.274089192331 |
| C | 0.966905379394 | 0.770113435642 | -0.029334605018 |
| C | 0.961435353015 | -0.476514873653 | -0.910512443978 |
| C | -0.272047410572 | 0.912035019193 | 0.798935654862 |
| C | 2.114102668787 | -0.439517737731 | -1.926206962334 |
| C | -0.575682845865 | 1.928519566336 | 1.711293875782 |
| C | -2.221107206148 | 0.517817217193 | 1.581305679135 |
| H | 1.844000055737 | 0.752487548760 | 0.626273784734 |
| H | 1.093300795161 | 1.646763028988 | -0.667255144969 |
| H | 0.049798233513 | -0.461083056400 | -1.514351485125 |
| H | -2.702695836137 | 1.702820498710 | 1.371814620578 |
| H | -0.012148081767 | 2.815830617026 | 1.951807486763 |
| H | 1.584228446961 | -1.723146591418 | 0.603310038800 |
| H | 0.035227778945 | -1.955211257633 | 0.144074372883 |
| H | -3.058629805350 | -0.052498521310 | 1.954651630372 |
| H | 2.096681005299 | -2.277759199983 | -1.658280272256 |

TS EQ2-EQ3

| | | | |
|---|---|---|---|
| O | 2.693323954720 | -1.595944714816 | -2.160441626541 |
| O | 2.335961883003 | 0.551399628651 | -2.654161447099 |
| N | -1.470443975871 | 0.250986832694 | 0.485086146129 |
| N | 1.082024805853 | -1.644359270212 | -0.112980079489 |
| N | -1.746494199721 | 1.621408283700 | 2.330535501453 |
| C | 0.841908330782 | 0.876125849545 | -0.226074324632 |
| C | 0.937461887601 | -0.460430778354 | -0.964564101074 |
| C | -0.329369595862 | 0.949039182166 | 0.699851361394 |
| C | 2.065175869630 | -0.425058236281 | -2.009444463092 |
| C | -0.503395544547 | 1.733459142492 | 1.844001382161 |
| C | -2.351363093609 | 0.747285394097 | 1.514775863037 |
| H | 1.760479388767 | 1.048532633668 | 0.342934745168 |
| H | 0.799245173886 | 1.673252564349 | -0.971654748519 |
| H | 0.019564784359 | -0.597033996561 | -1.541871773329 |
| H | -2.448470170302 | 1.064170338873 | 0.265841801107 |
| H | 0.222790146094 | 2.388745298005 | 2.303700802153 |
| H | 1.648863381070 | -1.442318780138 | 0.703669406713 |
| H | 0.179956719209 | -1.963125193893 | 0.215803679870 |
| H | -3.267506856053 | 0.223471274409 | 1.743513622306 |
| H | 2.289588904088 | -2.195357765892 | -1.492719370659 |

TS EQ3-EQ4

| | | | |
|---|---|---|---|
| O | 2.654595605002 | -1.485547914760 | -2.268843523570 |
| O | 1.454829889120 | 0.365796814126 | -2.614046191344 |
| N | -1.284404065295 | 1.230934136258 | 0.035315517468 |
| N | 2.084081012513 | -1.676275846190 | 0.272566001670 |
| N | -2.054251586005 | 1.117891455202 | 2.102315032882 |
| C | 1.154311443518 | 0.709312865538 | 0.248418483864 |
| C | 1.296879335049 | -0.671139189659 | -0.445921878809 |
| C | -0.194032307553 | 0.939554415660 | 0.829135911900 |
| C | 1.817022350473 | -0.520476197027 | -1.886229061299 |
| C | -0.699440384512 | 0.872203008372 | 2.100738934802 |
| C | -2.374252389516 | 1.330399275501 | 0.850476457719 |
| H | 1.899063670430 | 0.784026099324 | 1.042089441292 |
| H | 1.379289466003 | 1.489102958941 | -0.480673406855 |
| H | 0.296751530141 | -1.090820938679 | -0.577924231662 |
| H | -1.257027980452 | 1.372460711208 | -0.961646556366 |
| H | -0.161411224761 | 0.670317049713 | 3.012842302670 |
| H | 2.838979636897 | -1.251085119597 | 0.798422618545 |
| H | 1.517270626248 | -2.206316876763 | 0.920835798759 |
| H | -3.358700337689 | 1.561169319128 | 0.476703200335 |
| H | 2.766445489859 | -2.060424426348 | -1.477594861967 |

# Optimized EQ13-$Ni^{2+}$

| | | | |
|---|---|---|---|
| O | 1.477111115298 | -1.614881243762 | -2.578689824567 |
| O | 0.029195720604 | -0.013203211266 | -2.133344652024 |
| N | -1.235648989319 | 1.441696298429 | -0.216350704219 |
| N | 2.533880112028 | -1.388720917160 | -0.003814224418 |
| N | -2.192205539097 | 1.530207390639 | 1.754963836307 |
| C | 1.102441249967 | 0.627539913643 | 0.365329896980 |

| | | | |
|---|---|---|---|
| C | 1.267092883982 | -0.846472626599 | -0.221966950318 |
| C | -0.291172891434 | 0.989480297730 | 0.681953621922 |
| C | 0.874194337717 | -0.781585396449 | -1.775442107001 |
| C | -0.927175726739 | 1.049485189387 | 1.919687045510 |
| C | -2.353046935352 | 1.753851858731 | 0.437103107781 |
| H | 1.677387767054 | 0.660873962306 | 1.288355054778 |
| H | 1.542624018820 | 1.329639930540 | -0.342991079672 |
| H | 0.516422639237 | -1.511301528519 | 0.211801186950 |
| H | -1.104634478690 | 1.442494248826 | -1.222595429216 |
| H | -0.521349405209 | 0.802363909544 | 2.886692150959 |
| H | 3.370154105996 | -0.835437832731 | -0.146795905414 |
| H | 2.656082813650 | -2.243080115614 | 0.523409498213 |
| H | -3.245381536782 | 2.129843676029 | -0.037411658570 |
| H | 2.148785264343 | -2.156857352950 | -2.130564111597 |
| Ni | -3.509904713602 | 1.903458529664 | 3.128009223708 |

## Optimized Tautomer-$Ni^{2+}$

| | | | |
|---|---|---|---|
| O | 1.848233200495 | -1.323590218491 | -2.575765543410 |
| O | -0.052349722105 | -0.262534698434 | -2.436792643020 |
| N | -1.549530381369 | 1.025887430621 | 0.028085068675 |
| N | 2.496299924493 | -1.389694224412 | -0.177114415955 |
| N | -2.151884472894 | 1.409926776404 | 2.075715161320 |
| C | 0.960023833617 | 0.571875125727 | 0.324846134754 |
| C | 1.168614666228 | -0.819862814464 | -0.345599544549 |
| C | -0.413863889748 | 0.881489594999 | 0.832626769887 |
| C | 0.948140205538 | -0.783876904647 | -1.873593353607 |
| C | -0.804987923055 | 1.123024179981 | 2.115256629573 |
| C | -2.581881731856 | 1.349105251293 | 0.819739281752 |
| H | 1.620298810650 | 0.602897503784 | 1.190507649724 |
| H | 1.306247338094 | 1.367124703025 | -0.342042775596 |
| H | 0.425657543641 | -1.522784020686 | 0.042552181957 |
| H | -2.728932890170 | 1.641761621077 | 2.875656195699 |
| H | -0.246942610256 | 1.123086623901 | 3.035982557384 |
| H | 3.207914449288 | -0.725870505703 | 0.107456533685 |
| H | 2.528109777825 | -2.200522156899 | 0.428567357824 |
| H | -3.596782690376 | 1.538552448869 | 0.509209063919 |
| H | 2.534706190436 | -1.637669217457 | -1.875837029914 |
| Ni | -1.516528713913 | 0.654862977881 | -1.821242974293 |

## Optimized EQ13-$Cu^{2+}$

| | | | |
|---|---|---|---|
| O | 0.725983213056 | -1.999563753380 | -2.011691714176 |
| O | -0.759757801798 | -0.740293509573 | -0.969783307335 |
| N | -0.845951387274 | 2.014278239149 | -0.132591428201 |
| N | 2.735202458910 | -1.172523514097 | -0.393833339524 |
| N | -2.299603084433 | 1.261280390585 | 1.331220325765 |
| C | 1.237035192876 | 0.689326633694 | 0.392348438031 |
| C | 1.439457536168 | -0.821479708465 | 0.022435799489 |
| C | -0.181211069248 | 1.051520785148 | 0.585864957872 |
| C | 0.359610587360 | -1.191615899218 | -1.068815405920 |
| C | -1.126702602251 | 0.601361050847 | 1.521887410558 |
| C | -2.103881178399 | 2.116941950530 | 0.316759244665 |
| H | 1.794778697980 | 0.857835579046 | 1.316492137856 |
| H | 1.685895839612 | 1.318025285865 | -0.378327937802 |
| H | 1.160208629818 | -1.446653002246 | 0.874594110243 |
| H | -0.465253047584 | 2.551519305706 | -0.901419683442 |
| H | -0.998483342376 | -0.148119439027 | 2.284959125309 |
| H | 3.277309102777 | -0.508061808219 | -0.930433806801 |
| H | 3.294759171898 | -1.766776084592 | 0.201464505871 |
| H | -2.832659684589 | 2.802319495689 | -0.086041839406 |
| H | 1.674455765687 | -2.228719914063 | -1.899936731397 |
| Cu | -3.956341185697 | 1.028792897025 | 2.312187114454 |

## Optimized Tautomer-$Cu^{2+}$

| | | | |
|---|---|---|---|
| O | 1.867809352806 | -1.429119820800 | -2.575955511151 |
| O | 0.095536123782 | -0.176561356285 | -2.468957943130 |
| N | -1.536415486238 | 1.094602692958 | 0.022389457569 |
| N | 2.372302332825 | -1.417823070627 | 0.021405450989 |
| N | -2.119944001022 | 1.343285696254 | 2.111662101065 |
| C | 0.953131257693 | 0.660166554194 | 0.264768966651 |
| C | 1.146305388217 | -0.799046040240 | -0.329416837177 |
| C | -0.397659494958 | 0.937631585303 | 0.805806607155 |
| C | 1.004496684819 | -0.773043298516 | -1.891018629898 |
| C | -0.783696327208 | 1.099338815017 | 2.128477120633 |
| C | -2.553934409665 | 1.346352603661 | 0.833219066941 |
| H | 1.671172321737 | 0.773667814837 | 1.074167885435 |
| H | 1.214462641180 | 1.399106599825 | -0.496217475300 |
| H | 0.323686356857 | -1.440313555163 | -0.000141317617 |
| H | -2.695321178176 | 1.545134273314 | 2.921547083534 |
| H | -0.213334578981 | 1.044682022474 | 3.040988860285 |
| H | 3.187385924944 | -0.836191746935 | 0.170558414181 |
| H | 2.349008122139 | -2.240985367595 | 0.607868548978 |
| H | -3.569372642177 | 1.563582299504 | 0.542254466689 |
| H | 2.521863331315 | -1.848808496721 | -1.969218141534 |
| Cu | -1.436920805785 | 0.777531270624 | -1.875975867599 |

# Model Complexes

1

| | | | |
|---|---|---|---|
| O | -0.926328087672 | -2.602449955945 | -0.343807141430 |
| O | 1.313801045508 | -2.228961027578 | -0.357832744647 |
| N | -2.103367068329 | 0.145412414581 | 0.686706623291 |
| N | -1.426143063460 | -0.833376312694 | -2.232156547342 |
| N | -1.648139331941 | 1.360521968303 | 2.436072863200 |
| C | -0.012798743582 | 0.624354102615 | -0.755767342681 |
| C | -0.082930129472 | -0.678025754817 | -1.579802812212 |
| C | -0.840021686138 | 0.728458880162 | 0.495601589366 |
| C | 0.250403710808 | -1.892662271386 | -0.724078405645 |
| C | -0.564643369389 | 1.480888816544 | 1.597222788514 |
| C | -2.551211665915 | 0.556750941828 | 1.877275621889 |
| H | 1.032353800068 | 0.776433214778 | -0.485738048570 |
| H | -0.265984103365 | 1.443928022008 | -1.436483699388 |
| H | 0.696216908704 | -0.626616009298 | -2.341541482106 |
| H | -1.747361513643 | 1.811719768509 | 3.337081610179 |
| H | 0.287587190027 | 2.090803276372 | 1.843982099742 |
| H | -1.665385319803 | 0.036525903556 | -2.708238008793 |
| H | -1.378096651786 | -1.541639955014 | -2.965791949005 |
| H | -3.490250554736 | 0.290152393358 | 2.333884582854 |
| H | -0.668717379182 | -3.373056514630 | 0.198448897968 |
| Mn | -2.662286686722 | -1.355539631260 | -0.561849055180 |

1_PCM

| | | | |
|---|---|---|---|
| O | -0.807381707976 | -2.592920011903 | -0.342594213713 |
| O | 1.295946361484 | -1.866580024621 | -0.024362282328 |
| N | -2.128923623459 | 0.258200210850 | 0.582353237960 |
| N | -1.412846567584 | -0.890038471136 | -2.218982193273 |
| N | -1.546430754579 | 1.173164829234 | 2.473000997796 |
| C | -0.073682926051 | 0.701619634624 | -0.840999318908 |
| C | -0.106770131707 | -0.663987881248 | -1.552799295321 |
| C | -0.848028383698 | 0.773332521183 | 0.434451499557 |
| C | 0.225859055662 | -1.766893255781 | -0.563307524843 |
| C | -0.480403689867 | 1.339943749819 | 1.619904020999 |
| C | -2.516844826239 | 0.518432691534 | 1.822826603169 |
| H | 0.964988370842 | 0.944555966391 | -0.626224479011 |
| H | -0.431648303187 | 1.452130608052 | -1.550495265925 |
| H | 0.694084306448 | -0.671814305304 | -2.293907130931 |
| H | -1.592904940897 | 1.485734943408 | 3.430401199668 |
| H | 0.423559647694 | 1.836960269450 | 1.920653225250 |
| H | -1.535156925021 | -0.176523618079 | -2.934044250858 |
| H | -1.395382072210 | -1.777323931761 | -2.715944731131 |
| H | -3.462549763061 | 0.249831160492 | 2.259490635960 |
| H | -0.553103914949 | -3.258254960063 | 0.318952897671 |
| Mn | -3.165681911667 | -0.855947855158 | -0.885184191780 |

1_SMD

| | | | |
|---|---|---|---|
| O | -0.748159565816 | -2.713694366613 | -0.458940515903 |
| O | 1.228731865970 | -1.800125801291 | 0.083082770435 |
| N | -2.157474379929 | 0.310014686087 | 0.592220301246 |
| N | -1.411142751416 | -0.888962908156 | -2.230835977298 |
| N | -1.533506041098 | 1.208134741785 | 2.483266597348 |
| C | -0.094121345377 | 0.697761583986 | -0.838047165515 |
| C | -0.123234638987 | -0.671635900481 | -1.541175741090 |
| C | -0.862770602888 | 0.786661739047 | 0.438229884070 |
| C | 0.200337439632 | -1.776340180871 | -0.556023487947 |
| C | -0.469976351120 | 1.343850425643 | 1.620405844063 |
| C | -2.524971079375 | 0.579105402805 | 1.835763665405 |
| H | 0.942781306005 | 0.952863412822 | -0.628170415510 |
| H | -0.462553422725 | 1.431951027333 | -1.559588216811 |
| H | 0.696757482769 | -0.682145314189 | -2.263444141401 |
| H | -1.566512923787 | 1.522419639625 | 3.442051176314 |
| H | 0.450031564612 | 1.816229352099 | 1.913746273644 |
| H | -1.537349608666 | -0.134426284934 | -2.900693589242 |
| H | -1.366290675877 | -1.750804193552 | -2.768306501196 |
| H | -3.473366942237 | 0.334712998867 | 2.281989027368 |
| H | -0.475591406065 | -3.378094166096 | 0.198554040631 |
| Mn | -3.164920623638 | -0.973853623928 | -0.830894388610 |

2

| | | | |
|---|---|---|---|
| O | -0.921492429678 | -2.623232790180 | -0.356376065137 |
| O | 1.328459421088 | -2.294282677388 | -0.440426064685 |
| N | -2.033816276377 | 0.094957908308 | 0.663103944908 |
| N | -1.474913966117 | -0.786765070321 | -2.123341948735 |
| N | -1.701229447635 | 1.389925972021 | 2.379409417953 |
| C | 0.068523470531 | 0.603462522059 | -0.731922276533 |
| C | -0.084906546886 | -0.678994186910 | -1.569578881681 |
| C | -0.800938422597 | 0.736569620506 | 0.487184076620 |
| C | 0.264447565811 | -1.922369061002 | -0.759888117002 |
| C | -0.600613200333 | 1.541257194930 | 1.568145708661 |
| C | -2.542436254795 | 0.517658318537 | 1.826559537154 |
| H | 1.114661378927 | 0.675848305800 | -0.432787980302 |

| | | | |
|---|---|---|---|
| H | -0.107965808114 | 1.446614191328 | -1.407488371342 |
| H | 0.640226638000 | -0.635208409056 | -2.383751234651 |
| H | -1.853381028434 | 1.868155546707 | 3.258966435405 |
| H | 0.210302169093 | 2.203907339586 | 1.818202366588 |
| H | -1.752520662244 | 0.113286630256 | -2.516524987983 |
| H | -1.495057420116 | -1.448733769175 | -2.901995836994 |
| H | -3.480265729714 | 0.215867928746 | 2.263670045295 |
| H | -0.675498117885 | -3.404250081260 | 0.177709632867 |
| Mn | -2.554888032546 | -1.400053163505 | -0.505679960404 |

2_PCM

| | | | |
|---|---|---|---|
| O | -0.887570797387 | -2.540699388951 | -0.294721573018 |
| O | 1.336032304721 | -2.214762301768 | -0.378256122502 |
| N | -2.022723023249 | 0.112773566487 | 0.601742850102 |
| N | -1.467115110694 | -0.781981700735 | -2.114668026098 |
| N | -1.669633763300 | 1.325368262478 | 2.376939243173 |
| C | 0.067727572559 | 0.620042355635 | -0.763015007093 |
| C | -0.095217867313 | -0.677218025863 | -1.568236539188 |
| C | -0.791428287254 | 0.738957151806 | 0.456633902348 |
| C | 0.237446584879 | -1.881700445417 | -0.703041898235 |
| C | -0.568567481251 | 1.493692043916 | 1.570910538741 |
| C | -2.521856741195 | 0.489640040535 | 1.773372490265 |
| H | 1.113494008742 | 0.711475557798 | -0.474118185207 |
| H | -0.136851320889 | 1.446768023073 | -1.448233606567 |
| H | 0.640241694872 | -0.665198438507 | -2.372180207980 |
| H | -1.812896327357 | 1.754913318298 | 3.277965201147 |
| H | 0.255455325120 | 2.124916201221 | 1.848609379053 |
| H | -1.735113928274 | 0.106416950176 | -2.530092024166 |
| H | -1.494609555500 | -1.465920677726 | -2.867823337363 |
| H | -3.460078864901 | 0.171936134586 | 2.192501782257 |
| H | -0.664136477064 | -3.277605705239 | 0.299762917264 |
| Mn | -2.775900645287 | -1.378190651816 | -0.570862336929 |

2_SMD

| | | | |
|---|---|---|---|
| O | -0.886287114565 | -2.465481054032 | -0.222782450796 |
| O | 1.334964656724 | -2.132948264889 | -0.299923388801 |
| N | -2.078733823373 | 0.190468388054 | 0.546845669216 |
| N | -1.446675871736 | -0.816768206071 | -2.152079037994 |
| N | -1.622468529601 | 1.238289444806 | 2.406974942754 |
| C | 0.019486702243 | 0.655657380596 | -0.808232118077 |
| C | -0.095618436129 | -0.671350826267 | -1.574312838615 |
| C | -0.813524111115 | 0.752430385822 | 0.427432528053 |
| C | 0.224542742805 | -1.828926278308 | -0.651559396063 |
| C | -0.526531205074 | 1.403322463872 | 1.590473491372 |
| C | -2.531080156622 | 0.501942593632 | 1.754943650815 |
| H | 1.063538191660 | 0.812642140355 | -0.542891757838 |
| H | -0.253863256337 | 1.445579312270 | -1.513213033291 |
| H | 0.663680024155 | -0.675228116244 | -2.355993269128 |
| H | -1.724829480736 | 1.600356677946 | 3.343922694663 |
| H | 0.340426911435 | 1.959322832475 | 1.898601483985 |
| H | -1.686168099765 | 0.045468980567 | -2.636358005693 |
| H | -1.438660859674 | -1.553485466886 | -2.854205644175 |
| H | -3.478415919694 | 0.205182938834 | 2.170364249044 |
| H | -0.667284331751 | -3.157233109036 | 0.426727791157 |
| Mn | -2.849800732866 | -1.295619947507 | -0.641546120578 |

3

| | | | |
|---|---|---|---|
| O | -0.967892150292 | -2.543766705276 | -0.306343792597 |
| O | 1.286150780934 | -2.240802699963 | -0.333499298477 |
| N | -2.090896244559 | 0.132760481612 | 0.683288975471 |
| N | -1.412677497819 | -0.844674537241 | -2.256293887510 |
| N | -1.637341446377 | 1.324520775733 | 2.445408169549 |
| C | -0.023245117283 | 0.639709596983 | -0.774217493351 |
| C | -0.077439695171 | -0.675487146918 | -1.584879199207 |
| C | -0.839727966689 | 0.739324387867 | 0.485330664717 |
| C | 0.234252035275 | -1.880647084064 | -0.706032532312 |
| C | -0.563930085833 | 1.477294610893 | 1.595480015753 |
| C | -2.532057549940 | 0.512634918888 | 1.888020809843 |
| H | 1.021666993131 | 0.812073212005 | -0.515643611700 |
| H | -0.296196074152 | 1.447669602666 | -1.461119546634 |
| H | 0.711692545292 | -0.635311641995 | -2.336758945719 |
| H | -1.734268276847 | 1.762292777448 | 3.353594590246 |
| H | 0.281556394586 | 2.095388085514 | 1.845320968372 |
| H | -1.643868727302 | 0.015384308505 | -2.754166108221 |
| H | -1.359910378954 | -1.573605229127 | -2.969469520544 |
| H | -3.461099825410 | 0.220396850677 | 2.349622542807 |
| H | -0.750868812650 | -3.302968983688 | 0.269240308473 |
| Fe | -2.597201599955 | -1.268563310530 | -0.603693668951 |

3_PCM

| | | | |
|---|---|---|---|
| O | -0.934407797766 | -2.503042757297 | -0.291458716179 |
| O | 1.279784294129 | -2.116547811146 | -0.208675359726 |
| N | -2.048403721307 | 0.122616938935 | 0.623228704476 |
| N | -1.411018383819 | -0.825208068223 | -2.217451363613 |

| | | | |
|---|---|---|---|
| N | -1.603854530750 | 1.257050278800 | 2.429529348191 |
| C | -0.005648200223 | 0.656190038321 | -0.804699301928 |
| C | -0.087370066798 | -0.675028697216 | -1.573447330497 |
| C | -0.816882272534 | 0.742836451099 | 0.450076020034 |
| C | 0.199201831139 | -1.834096367603 | -0.634750111561 |
| C | -0.536532675018 | 1.448510155135 | 1.582965944920 |
| C | -2.490510609333 | 0.455050885362 | 1.829383631092 |
| H | 1.038178878744 | 0.839418507390 | -0.556861333347 |
| H | -0.301449525491 | 1.444050952928 | -1.502445814439 |
| H | 0.706430959576 | -0.678682028920 | -2.320370145820 |
| H | -1.701859212205 | 1.649648653848 | 3.353149785941 |
| H | 0.305913026693 | 2.059754853150 | 1.850081087703 |
| H | -1.624353929321 | 0.020650541224 | -2.739757836738 |
| H | -1.390539987908 | -1.580408187327 | -2.898314914126 |
| H | -3.413728767650 | 0.130871880220 | 2.275887821362 |
| H | -0.738488747207 | -3.192326420602 | 0.366284604834 |
| Fe | -2.877763262972 | -1.207687528084 | -0.699165280564 |

3_SMD

| | | | |
|---|---|---|---|
| O | -0.896751585058 | -2.423724857210 | -0.184925397141 |
| O | 1.326302135054 | -2.118232782384 | -0.283971141932 |
| N | -2.110666440587 | 0.223702427967 | 0.543857675991 |
| N | -1.424669827954 | -0.834742181313 | -2.199223127628 |
| N | -1.600185642214 | 1.207474374943 | 2.426318478186 |
| C | -0.012667829042 | 0.668868252721 | -0.822974290496 |
| C | -0.093623330925 | -0.667570395129 | -1.582814307956 |
| C | -0.832254126422 | 0.756106935837 | 0.422513820546 |
| C | 0.217664531722 | -1.809400990434 | -0.637754485104 |
| C | -0.511653338837 | 1.367003331007 | 1.598671564728 |
| C | -2.535074386599 | 0.511381915737 | 1.767128449889 |
| H | 1.028890390511 | 0.855294737324 | -0.566667126487 |
| H | -0.311767535494 | 1.446342766145 | -1.531434350611 |
| H | 0.690857513867 | -0.668863551880 | -2.339193331169 |
| H | -1.681429147095 | 1.547602257512 | 3.373562337353 |
| H | 0.373563260230 | 1.891747503202 | 1.909843312279 |
| H | -1.652322020885 | 0.017330211524 | -2.704914185322 |
| H | -1.386258587380 | -1.583731114118 | -2.885817635009 |
| H | -3.483153352285 | 0.227147437754 | 2.189610994319 |
| H | -0.679056614764 | -3.098332499048 | 0.482611637251 |
| Fe | -2.879046765860 | -1.301781510165 | -0.661239451683 |

4

| | | | |
|---|---|---|---|
| O | -0.957808728891 | -2.545891831587 | -0.322715411933 |
| O | 1.305783034146 | -2.343583225369 | -0.469771514909 |
| N | -1.935059611222 | 0.014930056118 | 0.634014468808 |
| N | -1.500679845772 | -0.747417518868 | -2.036143429574 |
| N | -1.732285980760 | 1.364451416526 | 2.323462132409 |
| C | 0.141566375415 | 0.598645408276 | -0.734674490779 |
| C | -0.078021025105 | -0.670773325090 | -1.571796466996 |
| C | -0.749533834207 | 0.731075771890 | 0.466188881678 |
| C | 0.258616922069 | -1.915551580947 | -0.766694204904 |
| C | -0.628992085301 | 1.574247482952 | 1.530454531351 |
| C | -2.502397411136 | 0.424478268893 | 1.775400736067 |
| H | 1.187458109249 | 0.620203748908 | -0.425782128071 |
| H | 0.006222191137 | 1.456360052704 | -1.400666589144 |
| H | 0.601761627219 | -0.648209039235 | -2.424854038819 |
| H | -1.933221764156 | 1.847316243190 | 3.190607446752 |
| H | 0.129375952617 | 2.296577405788 | 1.780149468700 |
| H | -1.798452711563 | 0.168062509677 | -2.377772406865 |
| H | -1.581199052119 | -1.381715797855 | -2.835556434759 |
| H | -3.424645440431 | 0.065473877638 | 2.202915302688 |
| H | -0.761846130341 | -3.300077646027 | 0.267880651054 |
| Fe | -2.499943290867 | -1.394980007587 | -0.491457062750 |

4_PCM

| | | | |
|---|---|---|---|
| O | -0.946418796560 | -2.451099707555 | -0.250260332264 |
| O | 1.294837821173 | -2.317717254365 | -0.438449201790 |
| N | -1.940618940824 | 0.027980943970 | 0.597333037477 |
| N | -1.482291832900 | -0.748267964598 | -2.055859393370 |
| N | -1.703083719691 | 1.314085010220 | 2.333667306727 |
| C | 0.124229646480 | 0.614375467275 | -0.759396500433 |
| C | -0.082512551470 | -0.670154872683 | -1.572980052218 |
| C | -0.755732504195 | 0.733267192562 | 0.445861327790 |
| C | 0.224462319781 | -1.888310561349 | -0.720816358783 |
| C | -0.606452332071 | 1.537718034014 | 1.537464996851 |
| C | -2.487266500328 | 0.399953602823 | 1.748828239516 |
| H | 1.169714384746 | 0.667825182732 | -0.460022609867 |
| H | -0.047941810828 | 1.453316674199 | -1.438033761323 |
| H | 0.618889095848 | -0.671932447235 | -2.405317355176 |
| H | -1.888943580816 | 1.760047269706 | 3.218683943893 |
| H | 0.165582473319 | 2.236046075098 | 1.803380230819 |
| H | -1.775577474840 | 0.154988846154 | -2.422084305597 |
| H | -1.556410332899 | -1.405380596759 | -2.831761109734 |
| H | -3.405451515548 | 0.025978852386 | 2.165176841476 |

| | | | |
|---|---|---|---|
| H | -0.787196269204 | -3.183539261689 | 0.370976883472 |
| Fe | -2.585120279187 | -1.375558214909 | -0.553202387461 |

4_SMD

| | | | |
|---|---|---|---|
| O | -0.941558912142 | -2.500913827420 | -0.300824079569 |
| O | 1.296165815610 | -2.290559719228 | -0.415642524006 |
| N | -1.920870601722 | 0.013161253798 | 0.608093379590 |
| N | -1.494435412147 | -0.734508019599 | -2.045783788110 |
| N | -1.707867273436 | 1.329551913668 | 2.328402422992 |
| C | 0.133417030405 | 0.604687293159 | -0.753234628597 |
| C | -0.093310169874 | -0.674669545837 | -1.567415331989 |
| C | -0.746489641231 | 0.732368937141 | 0.449439419731 |
| C | 0.209916583239 | -1.893995833194 | -0.722516109503 |
| C | -0.611513318521 | 1.554896896798 | 1.529715266333 |
| C | -2.475135883463 | 0.395435628017 | 1.751360241845 |
| H | 1.179253333454 | 0.645824656812 | -0.452572467465 |
| H | -0.036487927281 | 1.441973462751 | -1.434803712142 |
| H | 0.606838196245 | -0.680084519755 | -2.401617174592 |
| H | -1.905803961462 | 1.785468405059 | 3.207126273220 |
| H | 0.149845901622 | 2.268045535401 | 1.788121674147 |
| H | -1.765603514810 | 0.182460829591 | -2.395627313636 |
| H | -1.563267282373 | -1.383371802341 | -2.828945394167 |
| H | -3.392551645396 | 0.016640246221 | 2.166451166735 |
| H | -0.777218681767 | -3.212165401852 | 0.345282334978 |
| Fe | -2.596625334971 | -1.386624119196 | -0.541820215793 |

5

| | | | |
|---|---|---|---|
| O | -0.920832883464 | -2.602772581141 | -0.360950166765 |
| O | 1.328999838597 | -2.296650098258 | -0.454461897469 |
| N | -2.038948477896 | 0.089052044332 | 0.676896250637 |
| N | -1.465465393174 | -0.796539002199 | -2.146701800607 |
| N | -1.702430165358 | 1.386782316241 | 2.387588358954 |
| C | 0.057941595342 | 0.597960642811 | -0.731467591872 |
| C | -0.082537147935 | -0.679200747569 | -1.581611966388 |
| C | -0.799077346243 | 0.720362178155 | 0.498676385489 |
| C | 0.264824987261 | -1.920878363091 | -0.772887580715 |
| C | -0.595906265008 | 1.526592890296 | 1.575961567093 |
| C | -2.550842210532 | 0.525625010928 | 1.837505683130 |
| H | 1.105690550822 | 0.682236963263 | -0.440949303623 |
| H | -0.135930550393 | 1.445570445934 | -1.396640359094 |
| H | 0.651240523385 | -0.624170668600 | -2.387164351744 |
| H | -1.847538817598 | 1.863978892229 | 3.269495335224 |
| H | 0.217559384314 | 2.186278148389 | 1.825999258077 |
| H | -1.740267866318 | 0.091662269147 | -2.565420527579 |
| H | -1.481661797505 | -1.481608659990 | -2.903914233537 |
| H | -3.490368676968 | 0.228879433635 | 2.274644530262 |
| H | -0.683915907252 | -3.366916027503 | 0.200161979392 |
| Co | -2.543836074097 | -1.362622817015 | -0.491570128860 |

5_PCM

| | | | |
|---|---|---|---|
| O | -0.918096384394 | -2.483131789460 | -0.273912941467 |
| O | 1.304526913825 | -2.134663214171 | -0.254210578746 |
| N | -2.052694855702 | 0.126369660342 | 0.615832811560 |
| N | -1.423759936817 | -0.826049633786 | -2.196223970299 |
| N | -1.615627708262 | 1.255951890180 | 2.422665677561 |
| C | 0.003859919085 | 0.648244996142 | -0.800646701159 |
| C | -0.085848566550 | -0.676618581423 | -1.577843284015 |
| C | -0.812468676020 | 0.732120966386 | 0.451622497425 |
| C | 0.215517465960 | -1.839199308720 | -0.649170481169 |
| C | -0.537968794143 | 1.435170872974 | 1.587465036777 |
| C | -2.506476932610 | 0.464737200341 | 1.815346868174 |
| H | 1.048335539778 | 0.819905066188 | -0.547369405374 |
| H | -0.282682395579 | 1.444809827140 | -1.492258782571 |
| H | 0.689521941946 | -0.673013366837 | -2.343172133347 |
| H | -1.717897513629 | 1.648859865699 | 3.345794336106 |
| H | 0.307518519977 | 2.039008431907 | 1.861484870704 |
| H | -1.648482494051 | 0.015267533326 | -2.721940444322 |
| H | -1.424398588805 | -1.588389271293 | -2.870197110729 |
| H | -3.437464248150 | 0.149885503034 | 2.251912348133 |
| H | -0.724685482498 | -3.170596776945 | 0.386231259682 |
| Co | -2.834030423369 | -1.175047601035 | -0.698220432911 |

5_SMD

| | | | |
|---|---|---|---|
| O | -0.879148988291 | -2.612256298612 | -0.403193590137 |
| O | 1.298562947725 | -2.088538493465 | -0.198205720653 |
| N | -2.046624332938 | 0.165348003119 | 0.584612586103 |
| N | -1.456330224026 | -0.803528249472 | -2.128918881349 |
| N | -1.632820528842 | 1.299389321527 | 2.400601705519 |
| C | 0.035433488243 | 0.645937221371 | -0.788583107742 |
| C | -0.102454302929 | -0.675557323068 | -1.557118066097 |
| C | -0.800101353189 | 0.759801595025 | 0.444302908462 |
| C | 0.211066518415 | -1.853177523737 | -0.656860387441 |
| C | -0.539644681865 | 1.466994070869 | 1.580722340876 |
| C | -2.514651377134 | 0.508833769039 | 1.777923085914 |

| | | | |
|---|---|---|---|
| H | 1.081416052041 | 0.785427344882 | -0.521718805260 |
| H | -0.222283571402 | 1.441747307452 | -1.492675387471 |
| H | 0.653666788742 | -0.682290957325 | -2.343418016908 |
| H | -1.751086830804 | 1.696400707091 | 3.321468506523 |
| H | 0.305904471666 | 2.065459852116 | 1.867683659327 |
| H | -1.700518994203 | 0.065949389971 | -2.595430988757 |
| H | -1.462109308068 | -1.533503568802 | -2.837018220992 |
| H | -3.453855842759 | 0.198290998101 | 2.201392527084 |
| H | -0.670119491793 | -3.314136954203 | 0.238742490500 |
| Co | -2.807603138601 | -1.322967941886 | -0.581119197492 |

6

| | | | |
|---|---|---|---|
| O | -0.843049193301 | -2.721250841150 | -0.455524453162 |
| O | 1.392221736137 | -2.328704513225 | -0.581715658221 |
| N | -1.957166149578 | 0.064196766405 | 0.644049342388 |
| N | -1.546018071011 | -0.736002628573 | -1.928826477947 |
| N | -1.784427131764 | 1.461524976351 | 2.290620569143 |
| C | 0.174577346054 | 0.557470486246 | -0.677602473329 |
| C | -0.100051237320 | -0.683384900131 | -1.538935989120 |
| C | -0.748255413728 | 0.740666057445 | 0.493908848905 |
| C | 0.300664898580 | -1.975927292783 | -0.829328882830 |
| C | -0.645940712317 | 1.614647518967 | 1.535510690957 |
| C | -2.563340639721 | 0.529157517655 | 1.742714973102 |
| H | 1.210217908480 | 0.506781638249 | -0.338793387612 |
| H | 0.112791986765 | 1.432347355385 | -1.331252250263 |
| H | 0.518524746407 | -0.621429992195 | -2.436804421355 |
| H | -2.004139175086 | 1.977978979004 | 3.133861732209 |
| H | 0.123904960015 | 2.322276976157 | 1.792305473237 |
| H | -1.870334745974 | 0.187518298234 | -2.220804196013 |
| H | -1.688786782774 | -1.362179513152 | -2.726652969886 |
| H | -3.516367666045 | 0.216269238341 | 2.137384975486 |
| H | -0.572875860606 | -3.544283870589 | -0.002235845356 |
| Co | -2.445453503230 | -1.424049986652 | -0.388690160334 |

6_PCM

| | | | |
|---|---|---|---|
| O | -0.846975283100 | -2.537782571495 | -0.255122973603 |
| O | 1.370267900661 | -2.172464283866 | -0.382023868395 |
| N | -2.008534565072 | 0.133536629303 | 0.546070079563 |
| N | -1.503344632046 | -0.766971486808 | -2.040442301430 |
| N | -1.677131527293 | 1.277359289863 | 2.359157748764 |
| C | 0.092506201047 | 0.628569389365 | -0.777614382740 |
| C | -0.106583454321 | -0.678988627278 | -1.550083169595 |
| C | -0.770669146758 | 0.746537049375 | 0.435446666130 |
| C | 0.250093732068 | -1.875691756691 | -0.679923725741 |
| C | -0.561765079446 | 1.460636613036 | 1.578002684271 |
| C | -2.532070818603 | 0.472930002937 | 1.719882063536 |
| H | 1.139692465928 | 0.705320825938 | -0.491830498073 |
| H | -0.106226743913 | 1.453108843677 | -1.466769173621 |
| H | 0.577018444658 | -0.687680277834 | -2.398045360207 |
| H | -1.829175637271 | 1.675798680653 | 3.273222576464 |
| H | 0.264801359066 | 2.073023133148 | 1.887812423270 |
| H | -1.745779178278 | 0.090661716859 | -2.534560119633 |
| H | -1.595563351890 | -1.522115060683 | -2.720954750188 |
| H | -3.479786495040 | 0.149844733887 | 2.112076911991 |
| H | -0.599750619072 | -3.271877748750 | 0.332829911940 |
| Co | -2.784326271334 | -1.140132824652 | -0.633941302703 |

6_SMD

| | | | |
|---|---|---|---|
| O | -0.703855493881 | -2.810244051223 | -0.547517294616 |
| O | 1.290811376201 | -1.917623920772 | -0.025192117487 |
| N | -2.059426845223 | 0.240363744473 | 0.542387961738 |
| N | -1.521263326482 | -0.799747853260 | -2.017661750748 |
| N | -1.633286380832 | 1.291267761763 | 2.395309108042 |
| C | 0.044198575826 | 0.648637335241 | -0.791595576890 |
| C | -0.140908468018 | -0.691732166434 | -1.500991395262 |
| C | -0.789393641218 | 0.784840135849 | 0.435433079897 |
| C | 0.232612720520 | -1.860013993031 | -0.607022339282 |
| C | -0.520923023770 | 1.439841811186 | 1.599608721169 |
| C | -2.541137379967 | 0.561173044534 | 1.737976991268 |
| H | 1.093262463786 | 0.773800747100 | -0.533893800458 |
| H | -0.207615411777 | 1.432073528644 | -1.511089626887 |
| H | 0.552550254676 | -0.730364604965 | -2.344384422450 |
| H | -1.748011781154 | 1.659756881928 | 3.328542633024 |
| H | 0.346167619163 | 1.989429452656 | 1.917897447152 |
| H | -1.691583720072 | -0.007268683732 | -2.636850694787 |
| H | -1.613919071456 | -1.644201135808 | -2.583586544251 |
| H | -3.500917929502 | 0.277673344071 | 2.132788030384 |
| H | -0.393774443568 | -3.532850489835 | 0.027164965190 |
| Co | -2.946888793269 | -0.891188618397 | -0.704133934758 |

7

| | | | |
|---|---|---|---|
| O | -0.939057575654 | -2.599861280171 | -0.358002390206 |
| O | 1.314004172668 | -2.303478278023 | -0.435218617970 |
| N | -2.007192233082 | 0.071717392149 | 0.669650002694 |

| | | | |
|---|---|---|---|
| N | -1.471913915766 | -0.780978174776 | -2.125644466512 |
| N | -1.706810638521 | 1.380634416261 | 2.373171323245 |
| C | 0.078376051698 | 0.602223898422 | -0.734744971305 |
| C | -0.082004737034 | -0.678051013971 | -1.575596380697 |
| C | -0.788731374537 | 0.730924622310 | 0.487307040922 |
| C | 0.255424024357 | -1.921007549590 | -0.759912253965 |
| C | -0.608056431520 | 1.548811440585 | 1.562471323683 |
| C | -2.531020251435 | 0.488268696252 | 1.826791874495 |
| H | 1.125764624899 | 0.669146803599 | -0.438420138814 |
| H | -0.097277956604 | 1.449356946704 | -1.405350413636 |
| H | 0.641981358777 | -0.639512523017 | -2.391187361496 |
| H | -1.868409243088 | 1.859911787394 | 3.250656781654 |
| H | 0.187972988990 | 2.230976234515 | 1.808092265974 |
| H | -1.761161129193 | 0.115788792440 | -2.516750878252 |
| H | -1.513399286368 | -1.456468841700 | -2.891041231669 |
| H | -3.461336639617 | 0.165803883783 | 2.264825032791 |
| H | -0.721695187761 | -3.364919618879 | 0.210389554181 |
| Ni | -2.498759321223 | -1.355665364296 | -0.508296655112 |

7_PCM

| | | | |
|---|---|---|---|
| O | -0.928106615550 | -2.476629716106 | -0.248540544735 |
| O | 1.304957337010 | -2.217933710999 | -0.343879837128 |
| N | -2.013056664810 | 0.097862578259 | 0.601369195521 |
| N | -1.451405066992 | -0.788217304424 | -2.145797999646 |
| N | -1.655614741713 | 1.284509854114 | 2.386028136170 |
| C | 0.054380296367 | 0.633634403395 | -0.783022887400 |
| C | -0.088827475713 | -0.675584341822 | -1.575720802748 |
| C | -0.792335533487 | 0.738429799401 | 0.447316634383 |
| C | 0.217297684400 | -1.865024119341 | -0.678913758048 |
| C | -0.566838414343 | 1.479426764493 | 1.570468411790 |
| C | -2.505802955085 | 0.446186993399 | 1.781541498429 |
| H | 1.100777145595 | 0.748853032944 | -0.506065224750 |
| H | -0.175306344778 | 1.450468422878 | -1.472002234152 |
| H | 0.658623115369 | -0.677961439158 | -2.367832585407 |
| H | -1.793106620651 | 1.698707971651 | 3.295163906168 |
| H | 0.252646094261 | 2.116800945037 | 1.847348439405 |
| H | -1.719473507770 | 0.088540547029 | -2.585311680608 |
| H | -1.482669138150 | -1.498543184435 | -2.873903005591 |
| H | -3.434289947516 | 0.108665286504 | 2.206034705790 |
| H | -0.740169329643 | -3.189878281595 | 0.385744265328 |
| Ni | -2.694982016816 | -1.288692231226 | -0.626835192764 |

7_SMD

| | | | |
|---|---|---|---|
| O | -0.931293308962 | -2.564139798375 | -0.359138492121 |
| O | 1.295853443738 | -2.231402334694 | -0.341819419072 |
| N | -1.985475128594 | 0.070668427280 | 0.632451736880 |
| N | -1.464326245548 | -0.767400440092 | -2.118927429472 |
| N | -1.678126616775 | 1.334400742739 | 2.372274035755 |
| C | 0.077249677964 | 0.615019431136 | -0.759881525018 |
| C | -0.092437700432 | -0.679504802842 | -1.569194610212 |
| C | -0.780048224065 | 0.737699172450 | 0.461304464494 |
| C | 0.209409068288 | -1.893066072893 | -0.709800214116 |
| C | -0.588073247184 | 1.528093300713 | 1.555411878579 |
| C | -2.497497952769 | 0.448932847960 | 1.795954287703 |
| H | 1.123555030126 | 0.705992591595 | -0.473768600719 |
| H | -0.137456686977 | 1.435554261886 | -1.449506147515 |
| H | 0.643654964676 | -0.674189371309 | -2.372378108963 |
| H | -1.837388122233 | 1.780508199114 | 3.264337160331 |
| H | 0.208951926291 | 2.201795229225 | 1.812242357146 |
| H | -1.732500230428 | 0.129488550271 | -2.517635893953 |
| H | -1.504643866816 | -1.460800455309 | -2.863714955509 |
| H | -3.420249717788 | 0.100499789164 | 2.225296536580 |
| H | -0.759607499768 | -3.262693124294 | 0.299137012263 |
| Ni | -2.602852262764 | -1.341833873730 | -0.569454633052 |

8

| | | | |
|---|---|---|---|
| O | -0.812752096625 | -2.826602153306 | -0.606137777976 |
| O | 1.402795899038 | -2.335943620579 | -0.609215104296 |
| N | -1.988474119230 | 0.073269400293 | 0.743987544097 |
| N | -1.526994475285 | -0.759549869236 | -1.994009755747 |
| N | -1.789715508222 | 1.549763904161 | 2.326011292885 |
| C | 0.143880444802 | 0.517235625358 | -0.623405724094 |
| C | -0.111163657753 | -0.694509703291 | -1.539340672329 |
| C | -0.806366491315 | 0.754478338805 | 0.522198368432 |
| C | 0.303611623272 | -2.018557757885 | -0.879985362610 |
| C | -0.682640512048 | 1.686696054761 | 1.525399617089 |
| C | -2.574626259664 | 0.591146334790 | 1.834058705802 |
| H | 1.162493725042 | 0.422673527558 | -0.243148220311 |
| H | 0.147160850302 | 1.411336979382 | -1.254084513941 |
| H | 0.547657566880 | -0.588710912816 | -2.405662150989 |
| H | -1.995500471595 | 2.108272991798 | 3.146471646004 |
| H | 0.102937112166 | 2.391114060548 | 1.743106429901 |
| H | -1.875131443663 | 0.154269197541 | -2.281535040262 |
| H | -1.646705015561 | -1.399788702998 | -2.780122253427 |

| | | | |
|---|---|---|---|
| H | -3.530986928115 | 0.307694901902 | 2.242554162659 |
| H | -0.523233601480 | -3.675306210345 | -0.216646772404 |
| Cu | -2.399549340963 | -1.455360116448 | -0.337304978479 |

8_PCM

| | | | |
|---|---|---|---|
| O | -0.838457852679 | -2.627142649258 | -0.364670534165 |
| O | 1.367598613136 | -2.168308976627 | -0.365313398076 |
| N | -2.031772981714 | 0.153829894812 | 0.595902687699 |
| N | -1.488327193989 | -0.784356621557 | -2.077603933471 |
| N | -1.684002242996 | 1.333489412766 | 2.374133984936 |
| C | 0.074193133996 | 0.614882246699 | -0.756627152893 |
| C | -0.106454508012 | -0.684941243448 | -1.550411347201 |
| C | -0.782906807504 | 0.742660779478 | 0.462904925594 |
| C | 0.254659185598 | -1.903429587475 | -0.706549723887 |
| C | -0.565668842655 | 1.480535687180 | 1.588229755828 |
| C | -2.551406647056 | 0.526868876949 | 1.761044511973 |
| H | 1.120803961287 | 0.690737433678 | -0.468607291501 |
| H | -0.122326820130 | 1.444853291967 | -1.439416190778 |
| H | 0.591768392244 | -0.668221282335 | -2.387791585350 |
| H | -1.829000135393 | 1.758212173223 | 3.278221772615 |
| H | 0.266675718950 | 2.093688061757 | 1.880555215075 |
| H | -1.766218572107 | 0.082737349395 | -2.534545560743 |
| H | -1.573842279965 | -1.529881521976 | -2.768297110644 |
| H | -3.502533187693 | 0.225124664867 | 2.161489120724 |
| H | -0.593241649415 | -3.374814775345 | 0.207840689662 |
| Cu | -2.692841983926 | -1.192900944759 | -0.577299395394 |

8_SMD

| | | | |
|---|---|---|---|
| O | -0.670971218341 | -2.847247035343 | -0.614184952021 |
| O | 1.239346264420 | -1.869732410722 | 0.051544290987 |
| N | -2.086620806883 | 0.271504196725 | 0.575223020429 |
| N | -1.504482357160 | -0.831553682209 | -2.073986388866 |
| N | -1.607853728241 | 1.298595204230 | 2.426137745814 |
| C | 0.004846897763 | 0.653180573021 | -0.794873048240 |
| C | -0.147749715552 | -0.693122305104 | -1.505733565789 |
| C | -0.807008974315 | 0.786757392767 | 0.448367836350 |
| C | 0.220292947393 | -1.853199391444 | -0.599063946834 |
| C | -0.504791190611 | 1.427417640583 | 1.612740289049 |
| C | -2.541427520672 | 0.593572662271 | 1.779191679546 |
| H | 1.054173119272 | 0.802410844007 | -0.551049584930 |
| H | -0.271328150634 | 1.429569750011 | -1.512912216727 |
| H | 0.577904371388 | -0.722699367889 | -2.323180737485 |
| H | -1.700739168015 | 1.665809522361 | 3.362494037674 |
| H | 0.378403021529 | 1.956310241550 | 1.921586954374 |
| H | -1.676002676991 | -0.053181232074 | -2.708423284042 |
| H | -1.578019087045 | -1.692162033940 | -2.614303932054 |
| H | -3.501718151726 | 0.330748758219 | 2.186494547116 |
| H | -0.362936960436 | -3.565388219468 | -0.032963740924 |
| Cu | -2.966619615160 | -0.873968837558 | -0.719915563430 |

9

| | | | |
|---|---|---|---|
| O | -0.934080170108 | -2.588611131735 | -0.345110887845 |
| O | 1.314118943045 | -2.256169467945 | -0.388780414144 |
| N | -2.096857984117 | 0.143064239913 | 0.698819221945 |
| N | -1.431636815723 | -0.841628694677 | -2.238496309415 |
| N | -1.659701309007 | 1.354944151131 | 2.441636545643 |
| C | -0.012955912298 | 0.621848266462 | -0.754576260891 |
| C | -0.079441101246 | -0.681988379985 | -1.585223520562 |
| C | -0.835918633968 | 0.732262644923 | 0.503223917288 |
| C | 0.248439630787 | -1.907435444545 | -0.736178580638 |
| C | -0.574343400920 | 1.486977331231 | 1.606634198346 |
| C | -2.556354525046 | 0.541821385271 | 1.885311200426 |
| H | 1.033651695744 | 0.771276643137 | -0.487910337693 |
| H | -0.266023537324 | 1.442850183309 | -1.433613942400 |
| H | 0.692739472506 | -0.626104524682 | -2.353107752822 |
| H | -1.766038265201 | 1.807250273281 | 3.341610928316 |
| H | 0.271164452371 | 2.104830540374 | 1.857388876196 |
| H | -1.676475736372 | 0.013503833457 | -2.737774085184 |
| H | -1.410025354430 | -1.584793430789 | -2.937557173751 |
| H | -3.493950995163 | 0.261612154908 | 2.336225238521 |
| H | -0.710890265124 | -3.352089917788 | 0.221881343824 |
| Zn | -2.508722888423 | -1.229798385263 | -0.581212765157 |

9_PCM

| | | | |
|---|---|---|---|
| O | -0.599030870973 | -2.824572084847 | -0.570731775594 |
| O | 1.225551276323 | -1.721828396766 | 0.140886708753 |
| N | -2.195086425015 | 0.415247612819 | 0.564974852603 |
| N | -1.462528220218 | -0.919857814167 | -2.168330668815 |
| N | -1.560795598967 | 1.193863642029 | 2.489264090745 |
| C | -0.095719322816 | 0.699797156658 | -0.828249146721 |
| C | -0.145626666415 | -0.675580275335 | -1.517186992135 |
| C | -0.866994299066 | 0.807672654767 | 0.445811197316 |
| C | 0.242904704262 | -1.784680904731 | -0.549969626571 |
| C | -0.470320528016 | 1.293376997586 | 1.655995028646 |

| | | | |
|---|---|---|---|
| C | -2.580222000150 | 0.657878069551 | 1.809420776356 |
| H | 0.945962856855 | 0.930879659161 | -0.618734640891 |
| H | -0.444086597906 | 1.444253805044 | -1.548245621496 |
| H | 0.627153547180 | -0.684491927523 | -2.289410548231 |
| H | -1.591070437493 | 1.474927135074 | 3.457313919861 |
| H | 0.471065674846 | 1.692020678984 | 1.986539592026 |
| H | -1.549717060096 | -0.308420589445 | -2.976592194031 |
| H | -1.505892861932 | -1.872425682399 | -2.520797383399 |
| H | -3.552275420416 | 0.458703415232 | 2.223518555606 |
| H | -0.281081907920 | -3.502338871105 | 0.049395044239 |
| Zn | -3.065492542075 | -0.560802010597 | -0.921681728270 |

9_SMD

| | | | |
|---|---|---|---|
| O | -0.707805518301 | -2.719774004219 | -0.438801946010 |
| O | 1.243471647961 | -1.748657973755 | 0.103874032842 |
| N | -2.201513726501 | 0.396814553437 | 0.557882669133 |
| N | -1.428016662885 | -0.912043172276 | -2.203293048660 |
| N | -1.531266030937 | 1.147930861142 | 2.488613014447 |
| C | -0.113888889048 | 0.712426660299 | -0.853093352059 |
| C | -0.132638127437 | -0.670295665716 | -1.528975940554 |
| C | -0.877539255263 | 0.803133150238 | 0.424699432057 |
| C | 0.215626571049 | -1.759466151947 | -0.534864557486 |
| C | -0.459323404549 | 1.270567888582 | 1.634209396425 |
| C | -2.558016237732 | 0.613970197252 | 1.815115990359 |
| H | 0.920923491988 | 0.981005682097 | -0.652416775812 |
| H | -0.496334992497 | 1.430382557596 | -1.582941623763 |
| H | 0.666641684356 | -0.687446920903 | -2.272864277942 |
| H | -1.546607999112 | 1.414019436338 | 3.462784958174 |
| H | 0.486790195117 | 1.670473614563 | 1.951106671575 |
| H | -1.520605765648 | -0.238400780981 | -2.960262363682 |
| H | -1.435399431144 | -1.836298791537 | -2.626541758337 |
| H | -3.518247445668 | 0.398140864877 | 2.249298264648 |
| H | -0.416744625663 | -3.381214471379 | 0.213818783784 |
| Zn | -3.042808178100 | -0.671645263711 | -0.934158129129 |

10

| | | | |
|---|---|---|---|
| O | -0.341406393586 | -2.594325225474 | 0.015210460587 |
| O | 1.707431581309 | -1.728528996901 | 0.392023312793 |
| N | -1.807032001315 | 0.239322841901 | 0.569360389468 |
| N | -0.896448569914 | -1.095191772610 | -2.030548680946 |
| N | -1.606569445828 | 1.760815565157 | 2.129779174624 |
| C | 0.307912693534 | 0.700971150829 | -0.795374169874 |
| C | 0.368703460849 | -0.732134733728 | -1.341779226922 |
| C | -0.620758371763 | 0.934583433421 | 0.356154261972 |
| C | 0.695715543090 | -1.712910250697 | -0.226725536977 |
| C | -0.493350203387 | 1.879428752215 | 1.334296811569 |
| C | -2.371572727524 | 0.769017759618 | 1.649228570522 |
| H | 1.315542197965 | 0.981563340152 | -0.490799576595 |
| H | 0.049097049696 | 1.360272633724 | -1.630062475472 |
| H | 1.212927410083 | -0.780675965383 | -2.033056242110 |
| H | -1.814428110711 | 2.328252820550 | 2.938671067940 |
| H | 0.273968834916 | 2.609510609331 | 1.523379245105 |
| H | -1.147767287507 | -0.339039528018 | -2.663001501646 |
| H | -0.735569510212 | -1.906900381048 | -2.622455283838 |
| H | -3.302147401867 | 0.460770914240 | 2.094753109669 |
| H | -0.050753036556 | -3.218492685495 | 0.702709639541 |
| Mn | -2.471175652566 | -1.557609563405 | -0.452803350906 |
| O | -4.608053173794 | -0.500524880594 | -0.829978552967 |
| O | -6.720453180385 | -1.184326463198 | -0.430590797243 |
| N | -3.350505730124 | -2.739528261591 | 1.142022896459 |
| N | -3.797218677524 | -2.729235086104 | -1.885901130583 |
| N | -3.843976617350 | -3.420970058243 | 3.162453468682 |
| C | -5.243330505276 | -3.835424340680 | -0.186410379339 |
| C | -5.166265217573 | -2.795874934660 | -1.313100779939 |
| C | -4.513222701265 | -3.501096979857 | 1.077764523446 |
| C | -5.626400233678 | -1.435356536895 | -0.813558064044 |
| C | -4.823965659476 | -3.920377266924 | 2.340063246925 |
| C | -2.979196793920 | -2.715935396944 | 2.417554565359 |
| H | -6.295562934717 | -3.988296206974 | 0.050756311957 |
| H | -4.882832972069 | -4.786401203251 | -0.591410188769 |
| H | -5.890705885663 | -3.089414756115 | -2.075962148201 |
| H | -3.784175644309 | -3.564244677175 | 4.160146601943 |
| H | -5.636609649021 | -4.519785839510 | 2.711365290385 |
| H | -3.478192148928 | -3.677447596021 | -2.070379881893 |
| H | -3.834789969862 | -2.270192542684 | -2.793077178159 |
| H | -2.118572721813 | -2.208073883955 | 2.818643615429 |
| H | -4.978890581996 | 0.352435043019 | -0.544200957992 |

10_PCM

| | | | |
|---|---|---|---|
| O | 0.564381841814 | -2.752770862868 | 0.625095544948 |
| O | 1.797243525015 | -0.949527536489 | 1.145494594823 |
| N | -1.904311214790 | 0.194135307290 | 0.189464417377 |
| N | -0.370038206629 | -1.791930369526 | -1.632773362076 |
| N | -1.853024848113 | 1.783620322460 | 1.684825050593 |

| | | | |
|---|---|---|---|
| C | 0.347373084039 | 0.490386819985 | -0.930596840246 |
| C | 0.704867481111 | -1.002000741337 | -0.990490616834 |
| C | -0.690525599417 | 0.855212302881 | 0.076537348214 |
| C | 1.079008030417 | -1.539873163545 | 0.380486920026 |
| C | -0.654368423984 | 1.847890681334 | 1.012708656853 |
| C | -2.576947659020 | 0.778718010792 | 1.169514559301 |
| H | 1.254036968895 | 1.049084008381 | -0.708755845969 |
| H | 0.030440869196 | 0.790596761638 | -1.932749149334 |
| H | 1.622092484916 | -1.091963815637 | -1.580694900942 |
| H | -2.140992847013 | 2.386523490616 | 2.439467935227 |
| H | 0.101322836586 | 2.572828301927 | 1.253772624000 |
| H | -0.421700835036 | -1.519035892746 | -2.611157014998 |
| H | -0.114111940420 | -2.775700635017 | -1.631942753118 |
| H | -3.554948190627 | 0.499291581054 | 1.518453807494 |
| H | 0.885025190173 | -3.060617516595 | 1.489380197385 |
| Mn | -2.425402987820 | -1.632704208963 | -0.805104854874 |
| O | -5.605805955888 | -0.166017090619 | -0.412318285325 |
| O | -7.013137449790 | -1.635672757470 | 0.536304260921 |
| N | -3.191149360073 | -2.824934195243 | 0.817938033411 |
| N | -4.361500939207 | -1.921031408635 | -1.916303780180 |
| N | -3.552864613487 | -3.587785158332 | 2.831734814721 |
| C | -5.206098129533 | -3.726372245241 | -0.423670973289 |
| C | -5.536516359211 | -2.428860254922 | -1.177921806666 |
| C | -4.375918083592 | -3.545763482225 | 0.801912437581 |
| C | -6.131745796596 | -1.387652025103 | -0.245817155193 |
| C | -4.606356625171 | -4.020792445480 | 2.060613151663 |
| C | -2.725028720002 | -2.870509451199 | 2.056819093123 |
| H | -6.141198743847 | -4.204606395305 | -0.140206545565 |
| H | -4.709708901072 | -4.398076307193 | -1.128706784290 |
| H | -6.340299492222 | -2.657732263596 | -1.884363825002 |
| H | -3.423911228849 | -3.771440274479 | 3.814304500467 |
| H | -5.409137590469 | -4.610920520216 | 2.463169736734 |
| H | -4.162066307639 | -2.564149245706 | -2.678226331111 |
| H | -4.591458257581 | -1.033548490734 | -2.354841432505 |
| H | -1.824053763276 | -2.403093807932 | 2.412020018891 |
| H | -6.072064181823 | 0.455423824021 | 0.171785013729 |

10_SMD

| | | | |
|---|---|---|---|
| O | 0.229517116013 | -2.792149932150 | 0.115013636735 |
| O | 1.658487134503 | -1.284400107339 | 0.959072051323 |
| N | -2.006620065617 | 0.076223673774 | 0.237725723525 |
| N | -0.422608608593 | -1.387403494085 | -2.010735294558 |

| | | | |
|---|---|---|---|
| N | -2.050362030487 | 1.373567182105 | 1.996505083675 |
| C | 0.258120194612 | 0.645859168299 | -0.743357234871 |
| C | 0.617802401065 | -0.794784686582 | -1.145471859369 |
| C | -0.813979549286 | 0.780204399361 | 0.286001272998 |
| C | 0.893768336298 | -1.634330152454 | 0.086416852180 |
| C | -0.834796292877 | 1.589233482797 | 1.386498584007 |
| C | -2.723385311404 | 0.455632299204 | 1.283184174360 |
| H | 1.156482226655 | 1.131443622142 | -0.367293131286 |
| H | -0.024903256487 | 1.169946581775 | -1.660174064356 |
| H | 1.572068055155 | -0.750778952197 | -1.677778536042 |
| H | -2.383247717210 | 1.824045747449 | 2.836289533680 |
| H | -0.111824749090 | 2.282111597092 | 1.777859378183 |
| H | -0.571665360639 | -0.760659645070 | -2.796805953258 |
| H | -0.080847548829 | -2.263077195471 | -2.397113750291 |
| H | -3.699357935333 | 0.090778714385 | 1.551954472586 |
| H | 0.440531917415 | -3.264464647428 | 0.940335382438 |
| Mn | -2.353939892580 | -1.777096341368 | -0.914632660528 |
| O | -5.078135303975 | -0.232958786666 | -1.010629261301 |
| O | -6.611806902105 | -1.157412954875 | 0.339194938494 |
| N | -3.103444825333 | -2.860486764266 | 0.855522514559 |
| N | -4.203921723681 | -2.502798913787 | -2.002228811352 |
| N | -3.449218556809 | -3.187453571947 | 2.990457955787 |
| C | -5.283042634855 | -3.706806033641 | -0.126452553517 |
| C | -5.417921077783 | -2.591904688482 | -1.172185695449 |
| C | -4.369765249942 | -3.403422469568 | 1.012744088468 |
| C | -5.766896889501 | -1.267412464013 | -0.522780444477 |
| C | -4.591117443220 | -3.605260617742 | 2.344741225892 |
| C | -2.582581244796 | -2.741006892745 | 2.066552322370 |
| H | -6.269793869982 | -3.931678776598 | 0.273099816946 |
| H | -4.946050472717 | -4.599950679443 | -0.659973887358 |
| H | -6.284685639299 | -2.834336412825 | -1.795501956697 |
| H | -3.287659827701 | -3.207730667928 | 3.986541433985 |
| H | -5.439339676576 | -4.000793546792 | 2.873877019892 |
| H | -4.031872404565 | -3.419553335467 | -2.405868817877 |
| H | -4.370291199507 | -1.871543009596 | -2.781091167010 |
| H | -1.613253359242 | -2.338826800300 | 2.304116168188 |
| H | -5.349041701713 | 0.580064922478 | -0.548468090613 |

11

| | | | |
|---|---|---|---|
| O | -0.651236340120 | -2.630693758360 | -0.229149467942 |
| O | 1.549814371975 | -2.150740420189 | -0.105932501469 |
| N | -1.785168764220 | 0.050246761568 | 0.693000338308 |

| | | | |
|---|---|---|---|
| N | -1.237347078376 | -0.793289178978 | -1.956369652344 |
| N | -1.758740662217 | 1.663871781845 | 2.174292432064 |
| C | 0.326294700989 | 0.615566886839 | -0.637080576111 |
| C | 0.143702270510 | -0.688129089172 | -1.416732242408 |
| C | -0.652838807827 | 0.829934224419 | 0.478450829689 |
| C | 0.480366049136 | -1.876455714586 | -0.529803818112 |
| C | -0.633329518922 | 1.831352832743 | 1.407844664136 |
| C | -2.429049517673 | 0.591491719390 | 1.724115056882 |
| H | 1.345470667498 | 0.633929016581 | -0.249522726478 |
| H | 0.259113175801 | 1.440450817134 | -1.351971208015 |
| H | 0.883257352635 | -0.698498163332 | -2.219530097425 |
| H | -2.037147164752 | 2.251115734360 | 2.946759923866 |
| H | 0.067592393724 | 2.629314527870 | 1.579784395590 |
| H | -1.527029763482 | 0.121078886093 | -2.294992094230 |
| H | -1.229356892633 | -1.393585090462 | -2.777576555863 |
| H | -3.353880001121 | 0.241270544855 | 2.146893950367 |
| H | -0.391810810238 | -3.376817434131 | 0.338832072216 |
| Mn | -2.474070890837 | -1.547735062412 | -0.406728299543 |
| O | -4.262422076918 | -0.564393854501 | -1.008749779150 |
| O | -6.471997327750 | -1.019781659758 | -1.033359239915 |
| N | -3.382698764330 | -2.519317211154 | 1.163911125099 |
| N | -3.482724008996 | -2.952435705070 | -1.635494668453 |
| N | -3.699204381159 | -3.310301638421 | 3.182046851376 |
| C | -5.281575158642 | -3.690004876633 | -0.089290416175 |
| C | -4.933601718700 | -2.858929497401 | -1.325913347026 |
| C | -4.496424235500 | -3.353114698942 | 1.143190361878 |
| C | -5.360389834714 | -1.414201102172 | -1.119245365547 |
| C | -4.697563479622 | -3.839562398789 | 2.404113331043 |
| C | -2.927352753456 | -2.524976905374 | 2.414267254203 |
| H | -6.348663101599 | -3.573460654742 | 0.103029223011 |
| H | -5.133294329286 | -4.742650419397 | -0.346184647705 |
| H | -5.539009443223 | -3.231298522248 | -2.154366477682 |
| H | -3.566294278202 | -3.484592259435 | 4.167454719369 |
| H | -5.446152282463 | -4.503277832158 | 2.799956410454 |
| H | -3.177524075126 | -3.911365858945 | -1.487314504850 |
| H | -3.341819312737 | -2.776095049554 | -2.627388365842 |
| H | -2.066886649616 | -1.991152885464 | 2.776678767624 |
| H | -4.579608497823 | 0.345862058098 | -0.876765194922 |

11_PCM

| | | | |
|---|---|---|---|
| O | -0.633066820225 | -2.577408174885 | -0.200926411115 |
| O | 1.583321064620 | -2.242381633575 | -0.357129777628 |

| | | | |
|---|---|---|---|
| N | -1.796679227901 | 0.039781056805 | 0.708627178142 |
| N | -1.287945446738 | -0.761538952042 | -1.939893443776 |
| N | -1.749370352524 | 1.622246673343 | 2.217749395741 |
| C | 0.311678625824 | 0.600803295623 | -0.619881562109 |
| C | 0.103798912720 | -0.671728119134 | -1.446342723721 |
| C | -0.655051369590 | 0.803495930648 | 0.507420477531 |
| C | 0.473078627763 | -1.897971178670 | -0.628073626060 |
| C | -0.620426174957 | 1.787039522613 | 1.454868040993 |
| C | -2.433950379023 | 0.569784467477 | 1.746716053067 |
| H | 1.333384112112 | 0.596536801400 | -0.240337859383 |
| H | 0.246340663639 | 1.444433934956 | -1.310559510649 |
| H | 0.814495008705 | -0.644595080951 | -2.272464696791 |
| H | -2.021259720019 | 2.192556276367 | 3.002923533594 |
| H | 0.089454872895 | 2.572366396138 | 1.639811125676 |
| H | -1.598558661457 | 0.159633311842 | -2.236754009533 |
| H | -1.310408600973 | -1.336385516078 | -2.777674474650 |
| H | -3.364349965104 | 0.226423192554 | 2.160620757460 |
| H | -0.387182460030 | -3.361070899570 | 0.319908972109 |
| Mn | -2.474647395657 | -1.545221210215 | -0.391757649078 |
| O | -4.288304317088 | -0.600476545401 | -0.958404700072 |
| O | -6.463759442825 | -1.050703464413 | -1.296525785853 |
| N | -3.370000059893 | -2.507327605909 | 1.174249724573 |
| N | -3.437278731575 | -2.969803808400 | -1.602226356229 |
| N | -3.716599801117 | -3.250494721766 | 3.202036088572 |
| C | -5.264782595604 | -3.673026051740 | -0.079376602534 |
| C | -4.890392941062 | -2.885190954534 | -1.338276691855 |
| C | -4.498433131693 | -3.315563451042 | 1.157933461592 |
| C | -5.338165483825 | -1.438449144670 | -1.210635368870 |
| C | -4.720934961428 | -3.772731314687 | 2.425883267644 |
| C | -2.922967355976 | -2.498921783993 | 2.425116757981 |
| H | -6.333738418911 | -3.550787030392 | 0.094136166418 |
| H | -5.111231747781 | -4.730105106537 | -0.308555567389 |
| H | -5.466225880079 | -3.299293623582 | -2.166037260564 |
| H | -3.593796274719 | -3.400266422976 | 4.191046271180 |
| H | -5.483847072285 | -4.414584271739 | 2.826912832328 |
| H | -3.118387159866 | -3.916645203687 | -1.415422483727 |
| H | -3.267008583142 | -2.817684178037 | -2.592629727875 |
| H | -2.053031038694 | -1.979070334828 | 2.782667911653 |
| H | -4.584371256530 | 0.322953773701 | -0.883581266824 |

11_SMD

| | | | |
|---|---|---|---|
| O | 0.067761882670 | -2.993358043162 | -0.570606676120 |

| | | | |
|---|---|---|---|
| O | 1.674452525287 | -1.698304679682 | 0.311404697424 |
| N | -1.751578915164 | 0.108054709406 | 0.753555355080 |
| N | -1.133088362128 | -1.031708578432 | -1.859468165934 |
| N | -1.501910174666 | 1.598318511954 | 2.335391686580 |
| C | 0.279522254778 | 0.629091814460 | -0.699371149853 |
| C | 0.226460113518 | -0.745238135024 | -1.356144655532 |
| C | -0.573682725661 | 0.806204969559 | 0.514358109444 |
| C | 0.733541314970 | -1.840488466447 | -0.436558173811 |
| C | -0.409668240871 | 1.725894761991 | 1.508604013000 |
| C | -2.284506517924 | 0.623555247061 | 1.856156845948 |
| H | 1.312674009809 | 0.857338475546 | -0.444661763750 |
| H | -0.021120459069 | 1.352907752388 | -1.461932529842 |
| H | 0.936305114901 | -0.734625645162 | -2.189986066999 |
| H | -1.687057972978 | 2.141026887324 | 3.166209678876 |
| H | 0.365616004066 | 2.448520000812 | 1.688246025993 |
| H | -1.437039444539 | -0.234799465899 | -2.414909791282 |
| H | -1.095521795183 | -1.822145851063 | -2.498339743202 |
| H | -3.213044302588 | 0.330241896541 | 2.311206977866 |
| H | 0.460958569644 | -3.659035431393 | 0.021212246947 |
| Mn | -2.518609420809 | -1.457457708928 | -0.343537531376 |
| O | -5.050633775810 | -0.451641587925 | -1.614926422720 |
| O | -6.748512466625 | -1.378483996283 | -0.476652498531 |
| N | -3.464815554077 | -2.446145719533 | 1.211519426602 |
| N | -3.597233235233 | -2.653003298261 | -1.685807844528 |
| N | -3.930463592210 | -3.161434143194 | 3.226738473179 |
| C | -5.166642969543 | -3.774988118009 | -0.151173271105 |
| C | -5.020575898051 | -2.820608151755 | -1.328511821168 |
| C | -4.540691799901 | -3.323293494740 | 1.128188397800 |
| C | -5.700572025384 | -1.488051191625 | -1.072123315608 |
| C | -4.840308116207 | -3.758663193665 | 2.385600230940 |
| C | -3.122541878576 | -2.384847315999 | 2.494327049525 |
| H | -6.224239566994 | -3.968756351681 | 0.017172581903 |
| H | -4.720235652260 | -4.724870782454 | -0.458408758911 |
| H | -5.567912553328 | -3.253630190530 | -2.172083044717 |
| H | -3.873658607783 | -3.284473711629 | 4.227101814404 |
| H | -5.597646449322 | -4.432842402272 | 2.742738403456 |
| H | -3.177528261655 | -3.577375356573 | -1.759538067913 |
| H | -3.529298819847 | -2.239435010113 | -2.612690918862 |
| H | -2.309685245101 | -1.819716021957 | 2.911810559675 |
| H | -5.547867930150 | 0.366895866372 | -1.438949902897 |

| | | | |
|---|---|---|---|
| O | -0.451144418523 | -2.626027220712 | -0.097811243889 |
| O | 1.653836413781 | -1.873383082004 | 0.206215459554 |
| N | -1.818040636343 | 0.175322172111 | 0.626086794061 |
| N | -1.037818905152 | -0.999231332907 | -2.032514934208 |
| N | -1.654451479803 | 1.722505349226 | 2.162474557274 |
| C | 0.280541099780 | 0.679836687450 | -0.757384577171 |
| C | 0.268351876639 | -0.716566203924 | -1.391620791009 |
| C | -0.650423250330 | 0.897577502295 | 0.397239074776 |
| C | 0.614856505596 | -1.773090524288 | -0.353886459514 |
| C | -0.546389793157 | 1.858795853497 | 1.361929015434 |
| C | -2.395591570666 | 0.706315975493 | 1.699797779606 |
| H | 1.299513209076 | 0.887058332131 | -0.431353004547 |
| H | 0.064658905672 | 1.400777465939 | -1.552070213649 |
| H | 1.080843620963 | -0.745807664336 | -2.121122902982 |
| H | -1.873915254486 | 2.295033522871 | 2.964927012826 |
| H | 0.201749485267 | 2.611585606723 | 1.538628471831 |
| H | -1.329004400316 | -0.184824545862 | -2.567219554052 |
| H | -0.927094007781 | -1.748965596579 | -2.710515192387 |
| H | -3.318443942749 | 0.381073761634 | 2.148722641899 |
| H | -0.158210043845 | -3.293899757661 | 0.546601238606 |
| Fe | -2.438456568680 | -1.568233023067 | -0.382630078037 |
| O | -4.442133321285 | -0.498812601116 | -0.941813152250 |
| O | -6.587626507232 | -1.100715378924 | -0.594473170378 |
| N | -3.361891047497 | -2.678550834069 | 1.179088742783 |
| N | -3.637857174412 | -2.814739169049 | -1.788946515633 |
| N | -3.832146747737 | -3.393320546098 | 3.193832097978 |
| C | -5.246498171799 | -3.779569957606 | -0.152535213992 |
| C | -5.045142383307 | -2.802888034480 | -1.315398688992 |
| C | -4.516878682467 | -3.450338019761 | 1.113658339532 |
| C | -5.486428999678 | -1.404092416504 | -0.913367534753 |
| C | -4.813662860375 | -3.891062722480 | 2.372134893507 |
| C | -2.978188604153 | -2.672521677975 | 2.451898613447 |
| H | -6.314845216126 | -3.830561827990 | 0.055958978764 |
| H | -4.958405074058 | -4.775288165745 | -0.503936744188 |
| H | -5.724897705506 | -3.106105978176 | -2.115013698581 |
| H | -3.764489459472 | -3.548055693679 | 4.189296209639 |
| H | -5.617228866445 | -4.503763236543 | 2.741531722832 |
| H | -3.327947734176 | -3.780878814308 | -1.864007033631 |
| H | -3.597023035307 | -2.438102282374 | -2.732446853470 |
| H | -2.116149612430 | -2.166768386819 | 2.852157107493 |
| H | -4.796526581482 | 0.378911315671 | -0.716950734520 |

12

12_PCM

| | | | |
|---|---|---|---|
| O | 0.444585002265 | -2.580006867716 | 0.911586367433 |
| O | 1.943683461343 | -0.907954459630 | 0.909795497030 |
| N | -1.857517192350 | 0.377645459135 | 0.322161293810 |
| N | -0.687953035708 | -1.930903298408 | -1.368993783564 |
| N | -1.493648876334 | 2.080799660491 | 1.632685793981 |
| C | 0.238093626146 | 0.367930086493 | -1.094757065953 |
| C | 0.510063139556 | -1.143066641143 | -0.996175046160 |
| C | -0.624495777101 | 0.920521430097 | -0.010790223454 |
| C | 1.047036915951 | -1.509628918502 | 0.377363690736 |
| C | -0.392723519338 | 1.983541971938 | 0.812033017146 |
| C | -2.351318051182 | 1.101250471604 | 1.317179012707 |
| H | 1.193111059878 | 0.888130353438 | -1.076939009436 |
| H | -0.208520767045 | 0.559338305894 | -2.073602425710 |
| H | 1.325482125645 | -1.374444540967 | -1.687426390005 |
| H | -1.632000803433 | 2.769006444457 | 2.356223535975 |
| H | 0.439290820419 | 2.659516476099 | 0.885432688114 |
| H | -0.838092038310 | -1.819793104910 | -2.368558335919 |
| H | -0.502597082976 | -2.920601822696 | -1.227599920520 |
| H | -3.293353307219 | 0.937182162769 | 1.809772957146 |
| H | 0.857046557876 | -2.777973822886 | 1.769128691945 |
| Fe | -2.552005261343 | -1.425588849766 | -0.389684633458 |
| O | -5.690364012144 | -0.156505983507 | -0.292684233765 |
| O | -7.049865390675 | -1.787415996748 | 0.439649293890 |
| N | -3.298338431570 | -2.818410314557 | 0.933651216556 |
| N | -4.190503242116 | -1.716464300563 | -1.784139492345 |
| N | -3.697857057729 | -3.930671502804 | 2.764884010448 |
| C | -5.113915915753 | -3.696993117147 | -0.598237789086 |
| C | -5.419357654328 | -2.333116189251 | -1.233969228626 |
| C | -4.396304480520 | -3.636420455774 | 0.708278646696 |
| C | -6.142637708067 | -1.417073342164 | -0.259811726059 |
| C | -4.651906313654 | -4.328515905956 | 1.855457191595 |
| C | -2.904171037171 | -3.022502045972 | 2.183463990270 |
| H | -6.052258512656 | -4.227670913465 | -0.453906442150 |
| H | -4.534912974835 | -4.273692570616 | -1.324343957218 |
| H | -6.135584881154 | -2.502044559482 | -2.043898033169 |
| H | -3.612210692410 | -4.260275240824 | 3.713686377764 |
| H | -5.410883020457 | -5.049431123131 | 2.097854136997 |
| H | -3.862522354909 | -2.296085204161 | -2.552296937981 |
| H | -4.416321390719 | -0.814873267494 | -2.196874636760 |
| H | -2.075934477475 | -2.538892274232 | 2.670070923783 |
| H | -6.208918388368 | 0.380782662047 | 0.329491437308 |

12_SMD

| | | | |
|---|---|---|---|
| O | 0.156410496049 | -2.649047546841 | 0.529600455765 |
| O | 1.851894606284 | -1.211922962755 | 0.834333300237 |
| N | -1.873352968378 | 0.222359454677 | 0.357938035379 |
| N | -0.636259734750 | -1.653285897032 | -1.766591866999 |
| N | -1.611120135050 | 1.688462535174 | 1.954203277470 |
| C | 0.260892056181 | 0.519500763325 | -0.961716225536 |
| C | 0.526437705059 | -0.985761680693 | -1.148856977910 |
| C | -0.654492076034 | 0.854433796370 | 0.166397743746 |
| C | 0.921484241477 | -1.615896389591 | 0.171478371614 |
| C | -0.484155510853 | 1.767650915879 | 1.166160989559 |
| C | -2.419614999366 | 0.746951445034 | 1.444362276575 |
| H | 1.211983807979 | 1.021606639138 | -0.796853188432 |
| H | -0.139529850405 | 0.892629409915 | -1.907928703562 |
| H | 1.404538893766 | -1.086026280653 | -1.791655891698 |
| H | -1.799823212059 | 2.239097288794 | 2.778997674241 |
| H | 0.319709103286 | 2.448034164462 | 1.381652944068 |
| H | -0.801850073957 | -1.216890346906 | -2.670181470646 |
| H | -0.410717131145 | -2.626578640161 | -1.954549467522 |
| H | -3.367148905730 | 0.471973833502 | 1.874053788668 |
| H | 0.460046656984 | -2.993265041640 | 1.388084096288 |
| Fe | -2.489664377127 | -1.520670799680 | -0.628282392686 |
| O | -5.326194243968 | -0.301955192617 | -0.456294041245 |
| O | -7.042720924084 | -1.623572115329 | 0.126777814357 |
| N | -3.258849232798 | -2.689379050442 | 0.946954610957 |
| N | -4.122091016530 | -2.084534934361 | -1.969086972847 |
| N | -3.749351498658 | -3.384663213005 | 2.957045540955 |
| C | -5.078310024053 | -3.780771896183 | -0.426526780215 |
| C | -5.354396436006 | -2.542692744334 | -1.300286755937 |
| C | -4.384987017512 | -3.493061870150 | 0.861862197644 |
| C | -6.001926108970 | -1.453550454403 | -0.470149722096 |
| C | -4.698845638790 | -3.924593362696 | 2.117913984585 |
| C | -2.904638112516 | -2.645142936518 | 2.221638553817 |
| H | -6.025474333194 | -4.269869832026 | -0.208479706153 |
| H | -4.491715160601 | -4.475039009190 | -1.034169264256 |
| H | -6.104267764567 | -2.825810619462 | -2.042827514105 |
| H | -3.694203528623 | -3.518529173796 | 3.956091668714 |
| H | -5.493954390388 | -4.551778169403 | 2.478669280045 |
| H | -3.784883527866 | -2.844048504603 | -2.555387488920 |
| H | -4.340547191735 | -1.313539012419 | -2.594712490009 |
| H | -2.071133643712 | -2.101177471546 | 2.630656234876 |

| | | | |
|---|---|---|---|
| H | -5.797779737660 | 0.338983752172 | 0.104824541220 |

13

| | | | |
|---|---|---|---|
| O | -0.743483426395 | -2.589099233308 | -0.168029268105 |
| O | 1.485460142054 | -2.246463751305 | -0.192741615889 |
| N | -1.817823886726 | 0.015606577579 | 0.693322783134 |
| N | -1.318914322618 | -0.796604737352 | -1.936226006663 |
| N | -1.797832817737 | 1.641591954916 | 2.160449192906 |
| C | 0.293364337627 | 0.574380302651 | -0.638945589705 |
| C | 0.079236350555 | -0.716978076374 | -1.432463677291 |
| C | -0.684493994454 | 0.792737923114 | 0.477763184611 |
| C | 0.411200703412 | -1.913648950997 | -0.556432835920 |
| C | -0.669179953683 | 1.802347564990 | 1.399204950531 |
| C | -2.467789127833 | 0.566070353562 | 1.714270178690 |
| H | 1.313631300561 | 0.568028800163 | -0.253456263623 |
| H | 0.240981908557 | 1.408587809590 | -1.344135894138 |
| H | 0.798415410934 | -0.730476208287 | -2.252551260632 |
| H | -2.079844201751 | 2.234615480409 | 2.927152324999 |
| H | 0.030282031502 | 2.602673964464 | 1.566040696950 |
| H | -1.608008504657 | 0.127900075500 | -2.247734157663 |
| H | -1.337881816068 | -1.379692607347 | -2.769426013501 |
| H | -3.393244444106 | 0.218288279186 | 2.137157944443 |
| H | -0.511814852959 | -3.297158236498 | 0.457489370862 |
| Fe | -2.474097237049 | -1.546630421706 | -0.403586384310 |
| O | -4.181291169790 | -0.570357067040 | -0.924174364808 |
| O | -6.390177842172 | -0.969554856918 | -1.142373458651 |
| N | -3.349882100240 | -2.487547125177 | 1.152935690380 |
| N | -3.403613459353 | -2.937121502679 | -1.607468075983 |
| N | -3.659484251142 | -3.297513179213 | 3.164142816602 |
| C | -5.247036476733 | -3.652703273604 | -0.107166082698 |
| C | -4.865067112337 | -2.836789680998 | -1.344700311381 |
| C | -4.464117168864 | -3.319894626700 | 1.128599045815 |
| C | -5.285273971008 | -1.389341906699 | -1.149660979884 |
| C | -4.660878611987 | -3.818219126246 | 2.386186144695 |
| C | -2.888215173433 | -2.506690222192 | 2.399680090580 |
| H | -6.314067493225 | -3.514739417991 | 0.071652350603 |
| H | -5.115331761997 | -4.709833763351 | -0.354348080207 |
| H | -5.446962616092 | -3.212398516143 | -2.187467064794 |
| H | -3.522982901998 | -3.480191465166 | 4.147522225610 |
| H | -5.407396792921 | -4.486842546827 | 2.777665793469 |
| H | -3.103838743401 | -3.893076080535 | -1.429662082202 |
| H | -3.233908937990 | -2.781687169984 | -2.598251809300 |
| H | -2.027760005083 | -1.975013271466 | 2.764624224112 |
| H | -4.481477949386 | 0.332066785981 | -0.719697271674 |

13_PCM

| | | | |
|---|---|---|---|
| O | -0.728011461441 | -2.535272666245 | -0.138996476450 |
| O | 1.490352258093 | -2.356701904690 | -0.445759252708 |
| N | -1.833826564489 | 0.007918100987 | 0.707811704798 |
| N | -1.370690757976 | -0.762280497173 | -1.916923715651 |
| N | -1.798970524030 | 1.611848527112 | 2.192952512448 |
| C | 0.282138914150 | 0.551125489969 | -0.615673203843 |
| C | 0.037101897246 | -0.704975299756 | -1.458761699600 |
| C | -0.689002824062 | 0.765948365783 | 0.506760888533 |
| C | 0.390927406517 | -1.943320193968 | -0.654346325337 |
| C | -0.662459299144 | 1.763580429029 | 1.440284433990 |
| C | -2.482295703502 | 0.554417768733 | 1.728725313481 |
| H | 1.301654672830 | 0.512365334681 | -0.231733961670 |
| H | 0.243995616620 | 1.405064710321 | -1.295587723326 |
| H | 0.727645261812 | -0.681642748674 | -2.300708557101 |
| H | -2.078062016637 | 2.193636782925 | 2.967014536451 |
| H | 0.045978771959 | 2.551631983513 | 1.618945213157 |
| H | -1.672400249387 | 0.170495590494 | -2.186292084018 |
| H | -1.423822030130 | -1.318162485813 | -2.765676855418 |
| H | -3.416638139965 | 0.217572331401 | 2.138619718632 |
| H | -0.511770465896 | -3.296734600135 | 0.426054115947 |
| Fe | -2.475290419727 | -1.542116019335 | -0.385721606413 |
| O | -4.206508527639 | -0.606709090967 | -0.870373763310 |
| O | -6.361768623004 | -0.993103824232 | -1.373261145505 |
| N | -3.334665528558 | -2.476111871248 | 1.164277725657 |
| N | -3.356591327929 | -2.949567103181 | -1.576406406439 |
| N | -3.671511233965 | -3.242216099674 | 3.184133596017 |
| C | -5.223249800242 | -3.639422474530 | -0.098754497038 |
| C | -4.818450651314 | -2.859625011653 | -1.353907241616 |
| C | -4.459653083933 | -3.288214868548 | 1.142715919882 |
| C | -5.253966599284 | -1.410338803728 | -1.225669270847 |
| C | -4.675886236164 | -3.760184126450 | 2.406746030988 |
| C | -2.882194390821 | -2.479880140758 | 2.411868726117 |
| H | -6.292040851126 | -3.499200082915 | 0.062539223089 |
| H | -5.083899117835 | -4.699391169454 | -0.323127072080 |
| H | -5.373650095051 | -3.270838299560 | -2.196042188789 |
| H | -3.544538684956 | -3.401368745662 | 4.171076783053 |
| H | -5.433762190343 | -4.411142546766 | 2.802538581004 |
| H | -3.045849179707 | -3.895509162482 | -1.370157482497 |

| | | | |
|---|---|---|---|
| H | -3.160092645571 | -2.809430815646 | -2.563649427323 |
| H | -2.014702537907 | -1.958763980869 | 2.772839700459 |
| H | -4.484173977478 | 0.315248069160 | -0.733214306751 |

13_SMD

| | | | |
|---|---|---|---|
| O | -0.728364702435 | -2.529200192654 | -0.119096151902 |
| O | 1.491319784640 | -2.307082219980 | -0.359825229184 |
| N | -1.831578287185 | 0.011957270446 | 0.694147017332 |
| N | -1.367032741202 | -0.788523256401 | -1.923869977760 |
| N | -1.785732642229 | 1.601437890941 | 2.195334491048 |
| C | 0.265125100718 | 0.562960536815 | -0.647245810907 |
| C | 0.034319839278 | -0.716774625464 | -1.457330420280 |
| C | -0.688522683284 | 0.771691525659 | 0.489655908194 |
| C | 0.380626300578 | -1.925488336255 | -0.611623099019 |
| C | -0.653906726921 | 1.759327898204 | 1.432967070829 |
| C | -2.471251446366 | 0.547069513056 | 1.724575596507 |
| H | 1.292243324247 | 0.562722480529 | -0.282894184142 |
| H | 0.180889788810 | 1.395844815362 | -1.349302424539 |
| H | 0.733547878418 | -0.714990072759 | -2.292870124812 |
| H | -2.060095207703 | 2.174358690206 | 2.979413208352 |
| H | 0.055652820371 | 2.546519424119 | 1.613186001357 |
| H | -1.665274529563 | 0.140800277817 | -2.210265220260 |
| H | -1.414162905915 | -1.373787111187 | -2.754132631765 |
| H | -3.402352498121 | 0.208387614669 | 2.140849260261 |
| H | -0.522130573264 | -3.241411398909 | 0.513433372134 |
| Fe | -2.476013708122 | -1.543500793186 | -0.389575102795 |
| O | -4.201395377992 | -0.601199048592 | -0.871764247967 |
| O | -6.360317369576 | -0.978853198274 | -1.350024532007 |
| N | -3.340775685975 | -2.477287173325 | 1.158619794863 |
| N | -3.360625964631 | -2.944181966510 | -1.577066557206 |
| N | -3.666721651834 | -3.261715685347 | 3.173836855793 |
| C | -5.229361652454 | -3.630891691450 | -0.109847027699 |
| C | -4.819174783663 | -2.844695274933 | -1.358908713111 |
| C | -4.464863173095 | -3.290475716836 | 1.133406651944 |
| C | -5.242359730228 | -1.397081087477 | -1.215629266753 |
| C | -4.674751812526 | -3.773856191627 | 2.393857917404 |
| C | -2.882541033650 | -2.491108224086 | 2.402789885131 |
| H | -6.297901090757 | -3.490277480439 | 0.052948321067 |
| H | -5.083507196522 | -4.687684736658 | -0.345478303861 |
| H | -5.377675227045 | -3.245019343853 | -2.204244744462 |
| H | -3.533446022916 | -3.430274668027 | 4.159931787985 |
| H | -5.429226695460 | -4.430839165232 | 2.787426375104 |

| | | | |
|---|---|---|---|
| H | -3.062067266511 | -3.894435355626 | -1.371467094775 |
| H | -3.156536739938 | -2.785925340407 | -2.560547072445 |
| H | -2.013592960484 | -1.974233975018 | 2.767472840623 |
| H | -4.471065689486 | 0.316344242693 | -0.685683958315 |

14

| | | | |
|---|---|---|---|
| O | -0.541385270572 | -2.672842722940 | -0.234934248628 |
| O | 1.582253642078 | -1.999143659630 | 0.118301811152 |
| N | -1.783785783615 | 0.109272124286 | 0.664127264893 |
| N | -1.115179937654 | -0.867134752531 | -2.000645203623 |
| N | -1.718400399698 | 1.694640187938 | 2.170652029704 |
| C | 0.330807930484 | 0.654358928396 | -0.670800119375 |
| C | 0.224184407696 | -0.693951528437 | -1.387352491637 |
| C | -0.641594746666 | 0.871643836349 | 0.449905290850 |
| C | 0.544991371997 | -1.830512127467 | -0.427993358207 |
| C | -0.597028734477 | 1.856844498589 | 1.395848361967 |
| C | -2.408411617088 | 0.640226604174 | 1.710330066455 |
| H | 1.348763015688 | 0.750219625133 | -0.292775439071 |
| H | 0.212203745044 | 1.438413205252 | -1.424452541012 |
| H | 1.009634326595 | -0.722120407520 | -2.145903440270 |
| H | -1.980389081892 | 2.274423493508 | 2.954653699520 |
| H | 0.119416495691 | 2.638984180641 | 1.576329962051 |
| H | -1.399321908679 | 0.008151588604 | -2.432849664492 |
| H | -1.057154694548 | -1.544238356423 | -2.756689901340 |
| H | -3.333165966955 | 0.294973462171 | 2.138858240336 |
| H | -0.271343168959 | -3.388474219443 | 0.366647769472 |
| Co | -2.473375764413 | -1.550824829588 | -0.424551733640 |
| O | -4.371289287865 | -0.531151739935 | -1.047401164816 |
| O | -6.546501198056 | -1.058163046853 | -0.766057350830 |
| N | -3.381111878791 | -2.585430732346 | 1.163260979847 |
| N | -3.596120111987 | -2.907099388263 | -1.726963105763 |
| N | -3.735999714576 | -3.343865729064 | 3.184668652828 |
| C | -5.282005862968 | -3.740178689819 | -0.102831160080 |
| C | -5.019221290140 | -2.841929433131 | -1.314019478817 |
| C | -4.503295110213 | -3.404887552062 | 1.133994264894 |
| C | -5.443896516826 | -1.411806164607 | -1.015046611387 |
| C | -4.729280584351 | -3.872002864424 | 2.398161051262 |
| C | -2.945358124032 | -2.576863046186 | 2.418939044541 |
| H | -6.348939095335 | -3.698479734431 | 0.116730337222 |
| H | -5.074935684721 | -4.770798204129 | -0.405767016068 |
| H | -5.676948826956 | -3.179484280620 | -2.118115090309 |
| H | -3.619583042501 | -3.507926917101 | 4.174101038841 |

| | | | |
|---|---|---|---|
| H | -5.492894337596 | -4.519446639134 | 2.792263032842 |
| H | -3.279929851387 | -3.872293939872 | -1.681220056916 |
| H | -3.513147740273 | -2.633583319166 | -2.702543264710 |
| H | -2.086121643317 | -2.044565234524 | 2.788224419119 |
| H | -4.705738898172 | 0.365676374611 | -0.871924416812 |

14_PCM

| | | | |
|---|---|---|---|
| O | 0.538141794915 | -2.640023449136 | 0.893738513265 |
| O | 1.890201535130 | -0.847814384458 | 0.964049050172 |
| N | -1.898660722300 | 0.170801181920 | 0.308605082140 |
| N | -0.608024348662 | -2.023924420866 | -1.390011133901 |
| N | -1.729378478727 | 1.940930681280 | 1.564227071598 |
| C | 0.193850044773 | 0.323491158828 | -1.094635008389 |
| C | 0.538737909140 | -1.170620992267 | -0.988811667470 |
| C | -0.724455999453 | 0.824754332796 | -0.031208296837 |
| C | 1.063245045247 | -1.512893262366 | 0.396433920560 |
| C | -0.613953855841 | 1.932132262810 | 0.757672174847 |
| C | -2.478833227548 | 0.869049837762 | 1.273090085558 |
| H | 1.120002426115 | 0.891905800013 | -1.052357235840 |
| H | -0.237581118443 | 0.494552781755 | -2.084090302440 |
| H | 1.378182241341 | -1.363746634395 | -1.662497288746 |
| H | -1.948342030778 | 2.633652245276 | 2.263256918759 |
| H | 0.142386179970 | 2.693126436779 | 0.815148314688 |
| H | -0.724237446678 | -1.941989971025 | -2.397312563793 |
| H | -0.383692369277 | -3.000052107613 | -1.215377803595 |
| H | -3.406778956386 | 0.625020932180 | 1.758561721657 |
| H | 0.942770486674 | -2.821242730104 | 1.758811348258 |
| Co | -2.451839332860 | -1.592018841889 | -0.487765456252 |
| O | -5.565094674489 | -0.072792534656 | -0.200827732105 |
| O | -6.996819714753 | -1.672574357889 | 0.458749419144 |
| N | -3.203103784270 | -2.841503281564 | 0.901978212061 |
| N | -4.128569228001 | -1.633328012418 | -1.764261498528 |
| N | -3.673986104893 | -3.842211642333 | 2.777172833367 |
| C | -5.091722367353 | -3.612215946336 | -0.587058129633 |
| C | -5.366496946982 | -2.232033457233 | -1.206091262938 |
| C | -4.364460421454 | -3.575837351038 | 0.714854181385 |
| C | -6.065734364474 | -1.315163634718 | -0.214927787863 |
| C | -4.663471088499 | -4.200622881906 | 1.890060897635 |
| C | -2.815205957383 | -3.022864455416 | 2.155676700679 |
| H | -6.043632311139 | -4.117337431584 | -0.440972441607 |
| H | -4.532271588029 | -4.197252217731 | -1.321414627193 |
| H | -6.089626533048 | -2.372208256843 | -2.014607976166 |
| H | -3.607346218853 | -4.137362327606 | 3.738796248970 |
| H | -5.474344407561 | -4.849470844319 | 2.165629024402 |
| H | -3.870958810038 | -2.159915721007 | -2.595848871372 |
| H | -4.316931701783 | -0.685793423360 | -2.080966320199 |
| H | -1.950345650786 | -2.583606672745 | 2.619761781054 |
| H | -6.082218842560 | 0.467632443457 | 0.419930364591 |

14_SMD

| | | | |
|---|---|---|---|
| O | 0.349760019363 | -2.603572100273 | 0.715645384561 |
| O | 1.920969790987 | -1.008013918564 | 0.853445519542 |
| N | -1.962079965615 | 0.082067677540 | 0.264812996946 |
| N | -0.565785642992 | -1.871532737165 | -1.645272259235 |
| N | -1.838000059299 | 1.681022085248 | 1.742029051578 |
| C | 0.168956840491 | 0.427869420439 | -1.047178138695 |
| C | 0.548204139312 | -1.062646512788 | -1.106090006140 |
| C | -0.787439358509 | 0.783660341912 | 0.040030302905 |
| C | 1.016265140455 | -1.544491810312 | 0.252442919594 |
| C | -0.706158108026 | 1.783967837795 | 0.964320762858 |
| C | -2.565909335869 | 0.644448455095 | 1.300170091340 |
| H | 1.078388518298 | 1.010111306934 | -0.914297352896 |
| H | -0.242411515807 | 0.692497761698 | -2.024847483293 |
| H | 1.418665814236 | -1.153740611330 | -1.760002295811 |
| H | -2.081036372890 | 2.276378212284 | 2.520263113380 |
| H | 0.039077022780 | 2.540205455006 | 1.132168925135 |
| H | -0.745047522003 | -1.553297761948 | -2.595201334803 |
| H | -0.276793180633 | -2.843726079950 | -1.717731742124 |
| H | -3.493964399302 | 0.328609867415 | 1.742929277358 |
| H | 0.704920419278 | -2.848212701454 | 1.588820591191 |
| Co | -2.364062760802 | -1.739484805033 | -0.560853943616 |
| O | -5.325171998209 | -0.060635153526 | -0.398803480190 |
| O | -6.844572225090 | -1.480652027416 | 0.441048716196 |
| N | -3.150631953698 | -2.879100595738 | 0.922175488705 |
| N | -4.050089817675 | -1.871644655889 | -1.837225369287 |
| N | -3.692624292307 | -3.600285999971 | 2.906138563224 |
| C | -5.121431331281 | -3.642248477674 | -0.460566585718 |
| C | -5.312246723658 | -2.310553778642 | -1.204127406887 |
| C | -4.371329936473 | -3.529519826559 | 0.822476582424 |
| C | -5.907097348756 | -1.255991712212 | -0.292819360485 |
| C | -4.714004234579 | -3.979876429076 | 2.063371444390 |
| C | -2.774565927013 | -2.934600035934 | 2.190854444565 |
| H | -6.100139247051 | -4.069882473237 | -0.254516955552 |
| H | -4.611816962880 | -4.322246538655 | -1.147766642880 |

| | | | |
|---|---|---|---|
| H | -6.069834286169 | -2.471222634482 | -1.976758445094 |
| H | -3.639108845322 | -3.788597481616 | 3.896608828608 |
| H | -5.575353345155 | -4.520401337750 | 2.411735407271 |
| H | -3.823068575480 | -2.547037086112 | -2.563897312099 |
| H | -4.193271082106 | -0.981534958125 | -2.306519029722 |
| H | -1.875663460716 | -2.512706726024 | 2.604816533651 |
| H | -5.765098829837 | 0.565247396125 | 0.203330659136 |

15

| | | | |
|---|---|---|---|
| O | -0.480943329568 | -2.881259850222 | -0.341190917997 |
| O | 1.567694815788 | -2.048147974828 | 0.095691706931 |
| N | -1.827952561762 | -0.023527726776 | 0.697618524020 |
| N | -1.262987333524 | -0.908330414908 | -1.863688864051 |
| N | -1.882609800854 | 1.632378749718 | 2.122758326785 |
| C | 0.302197703138 | 0.541183171098 | -0.594807721649 |
| C | 0.129012861334 | -0.776074903997 | -1.344446631058 |
| C | -0.702593249925 | 0.767497301300 | 0.493117060707 |
| C | 0.519103064011 | -1.949726918853 | -0.452765510286 |
| C | -0.733373517061 | 1.796045668609 | 1.391309815417 |
| C | -2.521824138607 | 0.538446096265 | 1.685024590194 |
| H | 1.312744105406 | 0.562033331987 | -0.186480842839 |
| H | 0.246793338140 | 1.357368488510 | -1.320238297795 |
| H | 0.840434348075 | -0.784103158262 | -2.173302615130 |
| H | -2.197784814358 | 2.237698918764 | 2.866917764910 |
| H | -0.051813184925 | 2.610954630810 | 1.561112010898 |
| H | -1.551023474189 | -0.016178818452 | -2.260837795382 |
| H | -1.259575005944 | -1.566559659355 | -2.638760389925 |
| H | -3.458274679190 | 0.189495832367 | 2.080689592023 |
| H | -0.150292417792 | -3.605999956868 | 0.218611263096 |
| Co | -2.476433866056 | -1.541048961380 | -0.384313908234 |
| O | -4.410578120629 | -0.393238188315 | -1.237979702010 |
| O | -6.530314882934 | -1.027602604668 | -0.806703172882 |
| N | -3.337367684481 | -2.448867910662 | 1.141784562986 |
| N | -3.469573181620 | -2.802507580565 | -1.601981456252 |
| N | -3.564396203376 | -3.301600459971 | 3.141178383437 |
| C | -5.259018079822 | -3.605952486794 | -0.081209567514 |
| C | -4.928621222420 | -2.746626949971 | -1.297577806184 |
| C | -4.444591245951 | -3.290333413152 | 1.135901613906 |
| C | -5.412609846809 | -1.316563863137 | -1.084709361316 |
| C | -4.590799038579 | -3.814295552958 | 2.388762728779 |
| C | -2.826264273066 | -2.490520176551 | 2.370344362859 |
| H | -6.321072549117 | -3.485940111242 | 0.133255298029 |

| | | | |
|---|---|---|---|
| H | -5.117314592146 | -4.654976983566 | -0.355051075439 |
| H | -5.502835689524 | -3.131247195326 | -2.143454978406 |
| H | -3.390722877900 | -3.503613846564 | 4.115112155994 |
| H | -5.319109532657 | -4.495178251505 | 2.792983337106 |
| H | -3.153826437186 | -3.766919812196 | -1.519191455763 |
| H | -3.331287089676 | -2.550125302520 | -2.577272500125 |
| H | -1.951264927016 | -1.967881599587 | 2.711564938628 |
| H | -4.799532327228 | 0.490477293737 | -1.112613006471 |

15_PCM

| | | | |
|---|---|---|---|
| O | -0.492586540647 | -2.776388542548 | -0.233781075624 |
| O | 1.672278480595 | -2.175617721566 | -0.238502564801 |
| N | -1.833049998516 | -0.028503738002 | 0.705302105403 |
| N | -1.297091460493 | -0.879830922780 | -1.859329209921 |
| N | -1.858414474830 | 1.597143328737 | 2.161052991220 |
| C | 0.298398380126 | 0.524288414207 | -0.583100410186 |
| C | 0.104150509352 | -0.762612859665 | -1.385281474475 |
| C | -0.692092848516 | 0.738048382272 | 0.519440285860 |
| C | 0.537972973618 | -1.971142037783 | -0.566757946307 |
| C | -0.702567292211 | 1.749166402770 | 1.437453542480 |
| C | -2.518902799511 | 0.528146512925 | 1.698203332214 |
| H | 1.313172735165 | 0.528228890435 | -0.186777876425 |
| H | 0.238840742615 | 1.359122665657 | -1.284608029880 |
| H | 0.785543303861 | -0.725019990519 | -2.235445566853 |
| H | -2.164897582176 | 2.188908962157 | 2.917287142230 |
| H | -0.007919100852 | 2.547901248380 | 1.621973318434 |
| H | -1.600874880660 | 0.022684266319 | -2.217865105920 |
| H | -1.319130404395 | -1.509000770759 | -2.656970013918 |
| H | -3.463020970978 | 0.191281377259 | 2.083717893447 |
| H | -0.179700383660 | -3.511470086404 | 0.320995785297 |
| Co | -2.478527095339 | -1.535571476733 | -0.375825707584 |
| O | -4.434621588937 | -0.441051692731 | -1.093052059696 |
| O | -6.587328454536 | -1.084198032852 | -1.131800934722 |
| N | -3.336625686121 | -2.434516498753 | 1.146025341642 |
| N | -3.437006886799 | -2.815831930833 | -1.585136250605 |
| N | -3.597649399102 | -3.239340749241 | 3.157969688914 |
| C | -5.241516591384 | -3.603620391667 | -0.081938209890 |
| C | -4.896733900837 | -2.780095736873 | -1.322546013909 |
| C | -4.453985709255 | -3.256696731901 | 1.143205863596 |
| C | -5.421466972788 | -1.357534717295 | -1.179641350667 |
| C | -4.623801892336 | -3.751538422331 | 2.404666483401 |
| C | -2.839455760721 | -2.458338705354 | 2.378017305419 |

| | | | |
|---|---|---|---|
| H | -6.308196212814 | -3.498348470358 | 0.111953587016 |
| H | -5.075205093241 | -4.654206828362 | -0.330183492499 |
| H | -5.439387970004 | -3.210726068022 | -2.164360374651 |
| H | -3.436453275432 | -3.415387564537 | 4.137279838216 |
| H | -5.362556927651 | -4.416646196193 | 2.812541116316 |
| H | -3.104612777443 | -3.770935351288 | -1.473503985325 |
| H | -3.274985003613 | -2.585730974892 | -2.561560712607 |
| H | -1.960739943943 | -1.943120231381 | 2.718122634740 |
| H | -4.809852185599 | 0.446731840510 | -0.962079429388 |

15_SMD

| | | | |
|---|---|---|---|
| O | -0.074338533725 | -2.944394887216 | -0.529736070232 |
| O | 1.804681022456 | -1.800422543580 | -0.081516780651 |
| N | -1.783322813187 | -0.064357163467 | 0.803280634148 |
| N | -1.294697184217 | -0.982820313907 | -1.783565416043 |
| N | -1.709418937781 | 1.497817165313 | 2.330128469123 |
| C | 0.206646002631 | 0.609194085809 | -0.644389208192 |
| C | 0.105082401058 | -0.717597384603 | -1.385121991954 |
| C | -0.674381095850 | 0.734648837600 | 0.556190730667 |
| C | 0.708528109694 | -1.861590221319 | -0.588933682378 |
| C | -0.620178987318 | 1.701029268108 | 1.517959331170 |
| C | -2.388378824816 | 0.437408781614 | 1.875015613062 |
| H | 1.244425276830 | 0.776219158034 | -0.361846611180 |
| H | -0.054020842327 | 1.392228409740 | -1.360901994235 |
| H | 0.729012041500 | -0.639978576868 | -2.279215104052 |
| H | -1.963856827904 | 2.050036563083 | 3.135940530550 |
| H | 0.076263249059 | 2.503972142960 | 1.678143325720 |
| H | -1.668743317623 | -0.140138334525 | -2.216548200240 |
| H | -1.291555978211 | -1.693293296515 | -2.511409471146 |
| H | -3.293835759100 | 0.077525355937 | 2.326367046214 |
| H | 0.371888319379 | -3.639482780826 | -0.013874434971 |
| Co | -2.458656576228 | -1.580942929112 | -0.277555617937 |
| O | -4.654657637628 | -0.451792945142 | -1.873244051256 |
| O | -6.523196667103 | -1.037437878589 | -0.776818634734 |
| N | -3.386175752021 | -2.446157917751 | 1.224498511326 |
| N | -3.452001902466 | -2.793630986240 | -1.519043172876 |
| N | -3.746487925706 | -3.229725946705 | 3.231270780823 |
| C | -5.294793714443 | -3.555224760614 | -0.057880030168 |
| C | -4.916022342601 | -2.752360455492 | -1.292738217966 |
| C | -4.541222405661 | -3.214809099023 | 1.188421593477 |
| C | -5.454628789920 | -1.331507299158 | -1.261503710578 |
| C | -4.773920749024 | -3.692991873804 | 2.445588558146 |

| | | | |
|---|---|---|---|
| C | -2.928491118297 | -2.490776616900 | 2.471308404634 |
| H | -6.364864604772 | -3.451453050684 | 0.112350111096 |
| H | -5.116757300716 | -4.606513255724 | -0.296537313344 |
| H | -5.423445757582 | -3.213172902306 | -2.145812483070 |
| H | -3.621067298757 | -3.411375174643 | 4.216418626807 |
| H | -5.556058360931 | -4.320889235145 | 2.832184987367 |
| H | -3.145631671761 | -3.760478819149 | -1.427358457134 |
| H | -3.268247154133 | -2.531124556343 | -2.483652270951 |
| H | -2.034293390614 | -2.023968789674 | 2.840617272059 |
| H | -5.069777140154 | 0.428959076820 | -1.851321141114 |

16

| | | | |
|---|---|---|---|
| O | -0.562196997476 | -2.690392989207 | -0.221934312216 |
| O | 1.565609265244 | -2.020649295942 | 0.105640532239 |
| N | -1.792628519164 | 0.079439779196 | 0.676028964349 |
| N | -1.151292177302 | -0.879796638183 | -1.989883951715 |
| N | -1.745625568981 | 1.684669256745 | 2.159466856705 |
| C | 0.315300653307 | 0.627963522749 | -0.667656131729 |
| C | 0.195767746029 | -0.716790996803 | -1.388169001927 |
| C | -0.656647925020 | 0.847547230042 | 0.453219345120 |
| C | 0.522452615583 | -1.853883826318 | -0.430376561580 |
| C | -0.623637019468 | 1.846131003058 | 1.385929997094 |
| C | -2.426634143304 | 0.618965168245 | 1.711125236037 |
| H | 1.334440931667 | 0.712662596998 | -0.290049782837 |
| H | 0.202552504897 | 1.416674313978 | -1.417208551083 |
| H | 0.971575060143 | -0.746880768101 | -2.156194182668 |
| H | -2.014358310359 | 2.272178064666 | 2.935405252613 |
| H | 0.084659990823 | 2.637878709142 | 1.556341069048 |
| H | -1.436087196411 | -0.002467166409 | -2.417209323946 |
| H | -1.107058044088 | -1.559875156472 | -2.743710287970 |
| H | -3.349959366879 | 0.271568116990 | 2.140319040502 |
| H | -0.298621467698 | -3.384220019152 | 0.407380737442 |
| Ni | -2.473535999594 | -1.550189091891 | -0.422267508678 |
| O | -4.353350177481 | -0.508976315866 | -1.038902683767 |
| O | -6.528257659913 | -1.045338033746 | -0.783051736231 |
| N | -3.373053288947 | -2.552768994270 | 1.162372932651 |
| N | -3.561536445801 | -2.888712554200 | -1.717803618308 |
| N | -3.707485195825 | -3.338105339547 | 3.175077003124 |
| C | -5.265034577698 | -3.715484925251 | -0.109398093334 |
| C | -4.990030742589 | -2.820994400378 | -1.320254908729 |
| C | -4.487575046633 | -3.381468458079 | 1.128761612817 |
| C | -5.421114421706 | -1.391717031067 | -1.023019205401 |

| | | | |
|---|---|---|---|
| C | -4.700948843780 | -3.865715254168 | 2.388988746276 |
| C | -2.927194808086 | -2.555868260964 | 2.413656741444 |
| H | -6.332785530161 | -3.665415337878 | 0.104567609710 |
| H | -5.063195391109 | -4.748373423918 | -0.407815185927 |
| H | -5.636696119057 | -3.160744311262 | -2.132057996241 |
| H | -3.583804327961 | -3.512041179228 | 4.161947584529 |
| H | -5.455202432884 | -4.526132409537 | 2.779549801271 |
| H | -3.244864586639 | -3.853410535162 | -1.667632299793 |
| H | -3.465698415605 | -2.610221688120 | -2.690424468883 |
| H | -2.069809549961 | -2.020907035964 | 2.782602138815 |
| H | -4.687039410130 | 0.380492525282 | -0.828200948818 |

16_PCM

| | | | |
|---|---|---|---|
| O | -0.512222354529 | -2.637112275635 | -0.163969237652 |
| O | 1.657604816440 | -2.062484285016 | -0.071524855823 |
| N | -1.801744683906 | 0.072892767849 | 0.679135305887 |
| N | -1.171160905180 | -0.891404992152 | -1.982818536266 |
| N | -1.710807127853 | 1.628065675001 | 2.208091372121 |
| C | 0.291171271884 | 0.623801225278 | -0.678012690149 |
| C | 0.180775004739 | -0.713831355295 | -1.416692797392 |
| C | -0.652245090422 | 0.819160650467 | 0.469785796788 |
| C | 0.555665545473 | -1.860781682325 | -0.490129203219 |
| C | -0.589376729143 | 1.787155778691 | 1.432216496323 |
| C | -2.417328943095 | 0.593474183893 | 1.731767056496 |
| H | 1.317136366407 | 0.733692131598 | -0.328996692165 |
| H | 0.133551129871 | 1.410934094294 | -1.419658203400 |
| H | 0.935760718083 | -0.713740081583 | -2.203944153606 |
| H | -1.962737056505 | 2.190424513426 | 3.005897428359 |
| H | 0.135038017496 | 2.558577530988 | 1.619282574217 |
| H | -1.481109922159 | -0.021645321056 | -2.405922151360 |
| H | -1.144750166567 | -1.577460925298 | -2.731072253185 |
| H | -3.345094590648 | 0.254324969786 | 2.155665787325 |
| H | -0.253493460331 | -3.327636608855 | 0.470138686969 |
| Ni | -2.473812071541 | -1.548932666144 | -0.417854010827 |
| O | -4.413705895213 | -0.535438192469 | -0.972460898525 |
| O | -6.590322281151 | -1.092372432373 | -0.955346547863 |
| N | -3.364235098602 | -2.545761396120 | 1.163400023896 |
| N | -3.544400436326 | -2.870822476969 | -1.715905070068 |
| N | -3.751093650603 | -3.261899193404 | 3.188949334122 |
| C | -5.233958520376 | -3.722453599646 | -0.117979169669 |
| C | -4.971723457275 | -2.836878444628 | -1.340361346758 |
| C | -4.491420777995 | -3.352186258206 | 1.129645659948 |

| | | | |
|---|---|---|---|
| C | -5.445347582051 | -1.415493606756 | -1.076576945895 |
| C | -4.740257989858 | -3.793063631564 | 2.398790529872 |
| C | -2.942151429407 | -2.519863295853 | 2.419999434349 |
| H | -6.304752420003 | -3.718105486876 | 0.081306736928 |
| H | -4.979841709314 | -4.745798497919 | -0.406697349867 |
| H | -5.597637950871 | -3.209438811590 | -2.152396755753 |
| H | -3.648869476469 | -3.399783307455 | 4.182248539023 |
| H | -5.512429057523 | -4.428384149439 | 2.792383432895 |
| H | -3.201114126451 | -3.825370748652 | -1.668486276069 |
| H | -3.435431581335 | -2.577552446771 | -2.681705636726 |
| H | -2.081402942629 | -1.991948240761 | 2.788811891706 |
| H | -4.741324325076 | 0.353769739556 | -0.753844845003 |

16_SMD

| | | | |
|---|---|---|---|
| O | -0.493465389006 | -2.652362483804 | -0.160606129381 |
| O | 1.645145231337 | -1.996401797105 | 0.032016643425 |
| N | -1.802482626693 | 0.070613641984 | 0.660080570945 |
| N | -1.152295127849 | -0.925972690161 | -1.985811824876 |
| N | -1.718716468019 | 1.621176212696 | 2.194630031465 |
| C | 0.277642630967 | 0.628687019712 | -0.703033967381 |
| C | 0.188525915275 | -0.728364673602 | -1.408655444499 |
| C | -0.657213969090 | 0.822710459021 | 0.450527102396 |
| C | 0.550718575619 | -1.844765847745 | -0.446341032965 |
| C | -0.597663540696 | 1.787338491243 | 1.415903559917 |
| C | -2.419336773477 | 0.583225623066 | 1.714044667929 |
| H | 1.303286355766 | 0.777264405459 | -0.368493299874 |
| H | 0.082871419676 | 1.387700162826 | -1.465019297552 |
| H | 0.956518753740 | -0.745247296821 | -2.183784842340 |
| H | -1.975094828533 | 2.180135172242 | 2.995158615122 |
| H | 0.122303920038 | 2.563176978471 | 1.603931181559 |
| H | -1.454184347341 | -0.067785615848 | -2.437688735383 |
| H | -1.121186113041 | -1.645748500242 | -2.702262074647 |
| H | -3.344398716951 | 0.238904652710 | 2.140621489503 |
| H | -0.263815693633 | -3.296709138745 | 0.533154089083 |
| Ni | -2.465953680138 | -1.561557725264 | -0.433806634314 |
| O | -4.409295209011 | -0.520691588968 | -0.957373919398 |
| O | -6.580658557862 | -1.080946193984 | -0.855242692394 |
| N | -3.359038313963 | -2.562467180099 | 1.146647514170 |
| N | -3.556485940959 | -2.852596523591 | -1.730587165140 |
| N | -3.748201919184 | -3.267888567346 | 3.175693998737 |
| C | -5.230974830444 | -3.722190138081 | -0.134932153900 |
| C | -4.977431453916 | -2.812525928886 | -1.341608994461 |

| | | | |
|---|---|---|---|
| C | -4.493661543922 | -3.357957347343 | 1.116039518235 |
| C | -5.432890403572 | -1.397905212585 | -1.033761058600 |
| C | -4.744871110136 | -3.792098532256 | 2.386630201039 |
| C | -2.934051525102 | -2.535770994283 | 2.400804163009 |
| H | -6.301068811951 | -3.740347118687 | 0.066320644159 |
| H | -4.951686979389 | -4.732457416005 | -0.445332497858 |
| H | -5.616749120029 | -3.160407827136 | -2.154285773531 |
| H | -3.642464285710 | -3.405611691221 | 4.170206896848 |
| H | -5.521742678724 | -4.419927735768 | 2.784191473737 |
| H | -3.230482635179 | -3.814299180260 | -1.703514773837 |
| H | -3.448177408744 | -2.527486603649 | -2.687294256028 |
| H | -2.067667528040 | -2.016251413592 | 2.769285020378 |
| H | -4.714206212136 | 0.352438993666 | -0.651290353304 |

17

| | | | |
|---|---|---|---|
| O | 0.073040215762 | -3.314159597362 | -0.691586742658 |
| O | 0.482267335237 | -2.080347027181 | 1.148515184966 |
| N | -1.897774600523 | 0.225476047194 | 0.542575968686 |
| N | -0.922461379287 | -1.040142599062 | -1.833240675335 |
| N | -1.913773006689 | 1.847506745098 | 1.992208044712 |
| C | 0.491587065670 | 0.358122500810 | -0.324649862305 |
| C | 0.325844881073 | -0.988483291367 | -1.010231564533 |
| C | -0.631683583618 | 0.784974525734 | 0.566695879088 |
| C | 0.311596176023 | -2.162609689770 | -0.033414634249 |
| C | -0.638796582727 | 1.801162032277 | 1.478353637352 |
| C | -2.649487283612 | 0.890279612922 | 1.412727083252 |
| H | 1.415951565588 | 0.327640679157 | 0.252040885060 |
| H | 0.644809184882 | 1.111903575595 | -1.102967322652 |
| H | 1.189428050757 | -1.141637385804 | -1.664044158170 |
| H | -2.245481499290 | 2.505000265128 | 2.682470824241 |
| H | 0.134287277670 | 2.479016947473 | 1.794110112193 |
| H | -0.978537552836 | -0.200081397533 | -2.405161342229 |
| H | -0.857721497234 | -1.826563991647 | -2.474265984180 |
| H | -3.689443585151 | 0.708863437313 | 1.616241366920 |
| H | 0.163646661351 | -4.058737040861 | -0.072736111027 |
| Cu | -2.565260623756 | -1.267935086111 | -0.560405670331 |
| O | -6.791233752525 | -1.116936828827 | -0.891195905058 |
| O | -4.708147500048 | -0.284145380856 | -0.773742513620 |
| N | -3.212649926312 | -2.412066488139 | 0.985744758414 |
| N | -3.618848968679 | -2.495424610831 | -1.851287698072 |
| N | -3.395291145719 | -3.247836538902 | 2.989807037680 |
| C | -5.137026873121 | -3.582844380378 | -0.209605259801 |

| | | | |
|---|---|---|---|
| C | -5.022400491216 | -2.594693549077 | -1.377661789366 |
| C | -4.312698822081 | -3.257551272130 | 1.000442382316 |
| C | -5.481189639685 | -1.197205011849 | -0.985480507092 |
| C | -4.432788876876 | -3.776255277746 | 2.258413959665 |
| C | -2.680747279450 | -2.431527996133 | 2.201128436577 |
| H | -6.188251223484 | -3.657165764003 | 0.068721517124 |
| H | -4.859532332727 | -4.569984669426 | -0.592238076625 |
| H | -5.673294030822 | -2.950875407114 | -2.177952318942 |
| H | -3.198108425107 | -3.446675278078 | 3.959748060270 |
| H | -5.142784118541 | -4.465256701940 | 2.681136802648 |
| H | -3.216718454054 | -3.427183169555 | -1.919168523580 |
| H | -3.601384256262 | -2.101482783508 | -2.789243370764 |
| H | -1.800373737262 | -1.896993978294 | 2.513359773028 |
| H | -7.069168305362 | -0.218515325140 | -0.639001223550 |

17_PCM

| | | | |
|---|---|---|---|
| O | -0.104583928824 | -2.986186368051 | -0.090559862002 |
| O | 1.637534804734 | -1.796449180220 | 0.684978752797 |
| N | -1.792430215708 | 0.043058424120 | 0.633896110955 |
| N | -0.915413527582 | -1.197522229035 | -1.813834659408 |
| N | -1.791073290015 | 1.622051142553 | 2.133358434662 |
| C | 0.382073750237 | 0.517747702847 | -0.589381417238 |
| C | 0.374602018518 | -0.901535417549 | -1.150690856680 |
| C | -0.621961502181 | 0.769431321299 | 0.488516019798 |
| C | 0.715566329121 | -1.925054546803 | -0.075741392024 |
| C | -0.614984865709 | 1.753753774459 | 1.434410918297 |
| C | -2.477353736396 | 0.588645247694 | 1.630919506991 |
| H | 1.379674604975 | 0.730824534529 | -0.210668587259 |
| H | 0.212626194876 | 1.203477170872 | -1.423721927448 |
| H | 1.183891097700 | -0.971053357371 | -1.882688108674 |
| H | -2.090628265977 | 2.203593232091 | 2.900454329801 |
| H | 0.104739886428 | 2.519507383350 | 1.658397366172 |
| H | -1.123935046272 | -0.453498850688 | -2.474922629149 |
| H | -0.830300462675 | -2.050107270172 | -2.359988594805 |
| H | -3.438476293327 | 0.268753861632 | 1.989425998010 |
| H | 0.164202173394 | -3.608102478857 | 0.606575079559 |
| Cu | -2.464736668842 | -1.463258267777 | -0.481451475543 |
| O | -6.718947229908 | -1.026881539321 | -0.911402804289 |
| O | -4.615294635663 | -0.248276065646 | -0.840668342288 |
| N | -3.323620930394 | -2.486676524733 | 1.028096571825 |
| N | -3.578390136994 | -2.539993783956 | -1.831063150606 |
| N | -3.688623428366 | -3.289999707390 | 3.020815270206 |

| | | | |
|---|---|---|---|
| C | -5.224458262980 | -3.553792982718 | -0.284791488826 |
| C | -4.997928290923 | -2.557681159976 | -1.426112526432 |
| C | -4.467273500796 | -3.266607418250 | 0.973684372798 |
| C | -5.403842821206 | -1.146991302265 | -1.032998960370 |
| C | -4.702825045521 | -3.762962148972 | 2.223973236522 |
| C | -2.877420181844 | -2.527463595037 | 2.276216686293 |
| H | -6.290619220128 | -3.594125947006 | -0.067902116724 |
| H | -4.950404596704 | -4.542118230202 | -0.663009005272 |
| H | -5.628557099371 | -2.862665206512 | -2.262371429136 |
| H | -3.573401104287 | -3.476991330779 | 4.004756357578 |
| H | -5.480264892729 | -4.402092266582 | 2.600554746731 |
| H | -3.239946300977 | -3.493603957275 | -1.927250533960 |
| H | -3.489409866746 | -2.107159594258 | -2.746438603564 |
| H | -2.001622095343 | -2.031240497593 | 2.652892398848 |
| H | -6.936784355658 | -0.122123720372 | -0.629103226083 |

17_SMD

| | | | |
|---|---|---|---|
| O | -0.105683428553 | -3.151656181890 | -0.180205264732 |
| O | 1.340647189439 | -1.875538717388 | 0.966736775214 |
| N | -1.783437561832 | 0.165097523368 | 0.564268601481 |
| N | -0.850385229017 | -1.204026902439 | -1.819452458783 |
| N | -1.695547529569 | 1.692353375383 | 2.115079524284 |
| C | 0.486173596941 | 0.426819001445 | -0.533485351579 |
| C | 0.407006700575 | -0.998521391582 | -1.070971621276 |
| C | -0.538529611416 | 0.766995353365 | 0.498050833377 |
| C | 0.595744150739 | -2.033692362260 | 0.026116884495 |
| C | -0.477759508127 | 1.719151280197 | 1.473864344124 |
| C | -2.455448977653 | 0.746080528814 | 1.548085979471 |
| H | 1.479004948300 | 0.593426089358 | -0.120725632047 |
| H | 0.385726677692 | 1.096906429021 | -1.391373745091 |
| H | 1.259458477763 | -1.145404870592 | -1.741966838112 |
| H | -1.973898429953 | 2.279344365696 | 2.887858096072 |
| H | 0.305700617582 | 2.398592877705 | 1.756620603083 |
| H | -0.954066222816 | -0.453926927494 | -2.497536510194 |
| H | -0.807846215019 | -2.075458288024 | -2.341409159300 |
| H | -3.457329361789 | 0.511024567242 | 1.860069071874 |
| H | 0.072098835890 | -3.780479464156 | 0.541376438274 |
| Cu | -2.479255155815 | -1.355619391614 | -0.549302708070 |
| O | -6.927261175555 | -1.448857771305 | -0.570859359363 |
| O | -5.121912156748 | -0.181386534596 | -0.991979092363 |
| N | -3.302376918677 | -2.396359225291 | 0.982123513147 |
| N | -3.667756366291 | -2.301778281310 | -1.916515308388 |

| | | | |
|---|---|---|---|
| N | -3.704962328478 | -3.128604939361 | 2.995537348021 |
| C | -5.046890990136 | -3.642081395933 | -0.364591415918 |
| C | -5.039649214167 | -2.559099022794 | -1.447580555804 |
| C | -4.386377070926 | -3.255405092533 | 0.916380612152 |
| C | -5.671069755416 | -1.260842872215 | -0.983030567735 |
| C | -4.647526292477 | -3.708045458384 | 2.177312807263 |
| C | -2.914263286980 | -2.345577411144 | 2.247775031138 |
| H | -6.074968688404 | -3.936188843895 | -0.164470242133 |
| H | -4.545679532150 | -4.515737261445 | -0.790602063672 |
| H | -5.654641596492 | -2.919214033796 | -2.276233549874 |
| H | -3.619225964084 | -3.263342078166 | 3.992480396615 |
| H | -5.397540677252 | -4.381350909512 | 2.551149839024 |
| H | -3.248243381725 | -3.181003059001 | -2.208561854875 |
| H | -3.688673557141 | -1.695622978872 | -2.733455124208 |
| H | -2.093086209947 | -1.775237192056 | 2.642899460293 |
| H | -7.300869740355 | -0.597103682477 | -0.284317275819 |

18

| | | | |
|---|---|---|---|
| O | 0.625973933483 | -2.678080541553 | 0.973687265732 |
| O | 1.679925704331 | -0.687975145108 | 1.090837816787 |
| N | -1.913313096389 | 0.277638798892 | 0.315910214836 |
| N | -0.689587233381 | -2.097186004183 | -1.239489120764 |
| N | -1.676172280167 | 2.156362758391 | 1.391066592338 |
| C | 0.160657376184 | 0.263331123807 | -1.141803550571 |
| C | 0.478826396052 | -1.218819147702 | -0.913123396700 |
| C | -0.732937309501 | 0.875718985287 | -0.114678000288 |
| C | 0.996358479810 | -1.465907976891 | 0.499162988000 |
| C | -0.583617113850 | 2.048806606010 | 0.562254248840 |
| C | -2.454892414994 | 1.079884195432 | 1.226031200977 |
| H | 1.100807452260 | 0.811422075138 | -1.148852027872 |
| H | -0.265834335466 | 0.374035390438 | -2.143767910584 |
| H | 1.310525223237 | -1.491070398826 | -1.570027279203 |
| H | -1.859255702801 | 2.921084425828 | 2.024635153057 |
| H | 0.193437954337 | 2.791833554294 | 0.533296691368 |
| H | -0.783370814564 | -2.128293995029 | -2.252202461079 |
| H | -0.467078289601 | -3.047998686193 | -0.949336406269 |
| H | -3.376222047170 | 0.909780874811 | 1.755085915193 |
| H | 1.105947379284 | -2.838543153490 | 1.804617474526 |
| Zn | -2.481969868922 | -1.525694947947 | -0.314825032800 |
| O | -5.707772834718 | -0.035665877284 | -0.165285467483 |
| O | -6.838817902014 | -1.806676440749 | 0.652820457192 |
| N | -3.206803085834 | -2.885242377089 | 0.949851182959 |

| | | | |
|---|---|---|---|
| N | -4.087593689580 | -1.485275571935 | -1.661971499619 |
| N | -3.675699498370 | -4.104935453441 | 2.691768607479 |
| C | -5.028178341890 | -3.589726534727 | -0.666552857364 |
| C | -5.323678654960 | -2.171106318822 | -1.167050627951 |
| C | -4.327249436916 | -3.654051363926 | 0.649909921903 |
| C | -6.044138853236 | -1.345349315977 | -0.107565131557 |
| C | -4.622113928406 | -4.413211057890 | 1.742331663337 |
| C | -2.842208490389 | -3.184069522996 | 2.192024428023 |
| H | -5.975265985085 | -4.118226367857 | -0.576828755616 |
| H | -4.456053792751 | -4.115347954452 | -1.437570696521 |
| H | -6.033244780468 | -2.246538147634 | -1.996627450705 |
| H | -3.620831643515 | -4.504969192044 | 3.617343746452 |
| H | -5.411803725493 | -5.120445596081 | 1.923858771549 |
| H | -3.837184382533 | -1.900572257435 | -2.556495617849 |
| H | -4.317445480016 | -0.512622831011 | -1.859140094484 |
| H | -2.008382408922 | -2.765019021155 | 2.727565578118 |
| H | -6.304343417097 | 0.457351261080 | 0.424293926672 |

18_PCM

| | | | |
|---|---|---|---|
| O | 0.537132227284 | -2.611997823905 | 0.934583065679 |
| O | 1.869530549010 | -0.804377714016 | 0.985110117611 |
| N | -1.907955479832 | 0.208010365605 | 0.298165092150 |
| N | -0.614213861904 | -2.033784140978 | -1.355086106637 |
| N | -1.729280260906 | 1.997808071637 | 1.522120961041 |
| C | 0.184893291232 | 0.321483258515 | -1.106395013052 |
| C | 0.530221404951 | -1.170117349318 | -0.968822387559 |
| C | -0.730360531259 | 0.848314536360 | -0.053051136672 |
| C | 1.051758698326 | -1.485883176160 | 0.423815642254 |
| C | -0.614110201015 | 1.969244421421 | 0.715971761115 |
| C | -2.484420450686 | 0.924630474123 | 1.249979429035 |
| H | 1.111328974972 | 0.890392278422 | -1.079737713458 |
| H | -0.249041323395 | 0.469976371276 | -2.098349644483 |
| H | 1.371498302462 | -1.376627825097 | -1.636111769385 |
| H | -1.946039954424 | 2.705683534100 | 2.206421226630 |
| H | 0.145909513004 | 2.727439285822 | 0.760031679285 |
| H | -0.733188144377 | -1.971393989847 | -2.362903883570 |
| H | -0.390196294263 | -3.005519730807 | -1.159448298908 |
| H | -3.413405944796 | 0.693525546354 | 1.739628665417 |
| H | 0.942167087273 | -2.777747235443 | 1.802523417651 |
| Zn | -2.449875158770 | -1.583164106740 | -0.450671961224 |
| O | -5.562289140972 | -0.078926023308 | -0.159379524291 |
| O | -6.979000925475 | -1.696873326054 | 0.488295434573 |

| | | | |
|---|---|---|---|
| N | -3.204394832342 | -2.870275222472 | 0.906676818967 |
| N | -4.121955220304 | -1.611190110114 | -1.744984285211 |
| N | -3.673094101269 | -3.924647177944 | 2.751693866826 |
| C | -5.081906097096 | -3.614554962102 | -0.609133510211 |
| C | -5.358220437442 | -2.221701817080 | -1.197385116980 |
| C | -4.358970684066 | -3.607498095873 | 0.695259402330 |
| C | -6.054885275520 | -1.324485886869 | -0.186853441291 |
| C | -4.656696420107 | -4.266586854191 | 1.852084702064 |
| C | -2.818743829797 | -3.081872371127 | 2.155386039062 |
| H | -6.032582402301 | -4.126009094273 | -0.477999360060 |
| H | -4.518488540784 | -4.180402891097 | -1.355363457476 |
| H | -6.083611508225 | -2.344011157744 | -2.006758026560 |
| H | -3.605555651093 | -4.247716557621 | 3.704156164541 |
| H | -5.463251922308 | -4.929169680356 | 2.106814862959 |
| H | -3.858885302149 | -2.123201752926 | -2.583197326063 |
| H | -4.310669506247 | -0.658707758122 | -2.044822926871 |
| H | -1.958195398013 | -2.649748467576 | 2.633754843354 |
| H | -6.081556187413 | 0.450313005511 | 0.469142157512 |

18_SMD

| | | | |
|---|---|---|---|
| O | 0.393867227338 | -2.465270209152 | 1.129691568288 |
| O | 1.817153373546 | -0.731172015915 | 1.104264967965 |
| N | -1.946449631638 | 0.271125989067 | 0.232382749522 |
| N | -0.576144631217 | -2.044135015974 | -1.283730097576 |
| N | -1.757226504927 | 2.095479894509 | 1.409726741955 |
| C | 0.201980812395 | 0.305305518527 | -1.104009636061 |
| C | 0.545858913562 | -1.174741135093 | -0.876357956489 |
| C | -0.744638551574 | 0.877282809640 | -0.104887745237 |
| C | 0.984599605116 | -1.417088518724 | 0.553024339911 |
| C | -0.623071254389 | 2.017328514576 | 0.633308713335 |
| C | -2.528009448813 | 1.029634266112 | 1.147470771606 |
| H | 1.121612789629 | 0.885904676329 | -1.082429567044 |
| H | -0.205118903280 | 0.386440213889 | -2.115073308669 |
| H | 1.426393130466 | -1.407415326396 | -1.481670567226 |
| H | -1.977804149240 | 2.829557412804 | 2.066743143748 |
| H | 0.153597431409 | 2.759596910786 | 0.672003687940 |
| H | -0.654243187180 | -1.989639758652 | -2.296254564194 |
| H | -0.361749262880 | -3.012495562639 | -1.062880983647 |
| H | -3.470981948548 | 0.836701125224 | 1.626971342839 |
| H | 0.741989362834 | -2.575251441949 | 2.032447992744 |
| Zn | -2.489212269788 | -1.560154875001 | -0.503477053037 |
| O | -5.351617865029 | -0.079187992174 | -0.014150042851 |

| | | | |
|---|---|---|---|
| O | -6.889337058162 | -1.581286223522 | 0.626943899075 |
| N | -3.151866066312 | -3.040141246934 | 0.773232518982 |
| N | -4.174171356278 | -1.641750916749 | -1.777869762278 |
| N | -3.589214087404 | -4.096633688480 | 2.630329910938 |
| C | -5.187255723259 | -3.597162158662 | -0.619890059033 |
| C | -5.407212680833 | -2.175393228404 | -1.160048507994 |
| C | -4.375591250169 | -3.675564902715 | 0.627626602036 |
| C | -5.961342898178 | -1.263329118132 | -0.084037427049 |
| C | -4.652180637207 | -4.336417854023 | 1.788724146533 |
| C | -2.711046178010 | -3.309047336590 | 1.992471008441 |
| H | -6.158710620811 | -4.048176125094 | -0.428781084013 |
| H | -4.717807142748 | -4.174917208377 | -1.420250368555 |
| H | -6.197885237876 | -2.230969677445 | -1.913869895221 |
| H | -3.484744111123 | -4.448445076715 | 3.570728360753 |
| H | -5.491910645587 | -4.939960716906 | 2.082188999376 |
| H | -3.941477866987 | -2.237950641217 | -2.568110798290 |
| H | -4.353649172242 | -0.715880508383 | -2.155894498465 |
| H | -1.795536360126 | -2.951099042681 | 2.428408160666 |
| H | -5.760446884517 | 0.454949041199 | 0.690144756333 |

19

| | | | |
|---|---|---|---|
| O | -0.900836328410 | -2.600966152479 | -0.334049884791 |
| O | 1.307358244076 | -2.171162195462 | -0.370533963774 |
| N | -2.106725997778 | 0.162321536776 | 0.661087610645 |
| N | -1.444652922649 | -0.784312789537 | -2.235198877093 |
| N | -1.653652466091 | 1.367307772836 | 2.421551103962 |
| C | -0.002370702284 | 0.616779378747 | -0.761627839757 |
| C | -0.096764715951 | -0.680161608966 | -1.578792073745 |
| C | -0.849140854952 | 0.745302102031 | 0.474760200639 |
| C | 0.176069047441 | -1.930210229007 | -0.693319751714 |
| C | -0.572027207593 | 1.493457629423 | 1.580691103065 |
| C | -2.552652774966 | 0.559167340908 | 1.847629161503 |
| H | 1.039891683932 | 0.724483494014 | -0.463063898445 |
| H | -0.217218115335 | 1.457655642116 | -1.430217972291 |
| H | 0.684963796614 | -0.647339416367 | -2.336635930590 |
| H | -1.751179050583 | 1.801468702928 | 3.326808217042 |
| H | 0.283809921805 | 2.093887640630 | 1.832307672946 |
| H | -1.695961473689 | 0.097377182016 | -2.674774654913 |
| H | -1.404624304701 | -1.478814138941 | -2.977277396638 |
| H | -3.487641658372 | 0.279663533887 | 2.302237475794 |
| Mn | -2.488251632755 | -1.527363536696 | -0.488780416017 |

19_PCM

| | | | |
|---|---|---|---|
| O | -0.842320326345 | -2.548493016105 | -0.242062139364 |
| O | 1.354993676945 | -2.179415966632 | -0.424556734779 |
| N | -2.071211177338 | 0.161527691980 | 0.618975020571 |
| N | -1.448296646646 | -0.779564517743 | -2.180418029827 |
| N | -1.658198876300 | 1.355425653466 | 2.400811332931 |
| C | 0.028107512021 | 0.620830913575 | -0.763709631443 |
| C | -0.099943995258 | -0.679143443367 | -1.570480450001 |
| C | -0.821583994637 | 0.747402300380 | 0.465537420075 |
| C | 0.182918198772 | -1.910048543070 | -0.679069466946 |
| C | -0.560639257914 | 1.489946267771 | 1.582576314206 |
| C | -2.542040301654 | 0.548554477220 | 1.794626371845 |
| H | 1.071918491680 | 0.728468484273 | -0.471161809168 |
| H | -0.187129609775 | 1.453569635118 | -1.440838637108 |
| H | 0.669375717571 | -0.656458835722 | -2.342700045414 |
| H | -1.778768817263 | 1.784501563874 | 3.304663172191 |
| H | 0.286482086184 | 2.092358390373 | 1.855725732035 |
| H | -1.730626474050 | 0.113436332572 | -2.572254002164 |
| H | -1.433072946971 | -1.441272258641 | -2.950345935908 |
| H | -3.486135078186 | 0.261675519490 | 2.223486115374 |
| Mn | -2.665435693075 | -1.584758759953 | -0.506004711281 |

19_SMD

| | | | |
|---|---|---|---|
| O | -0.889559846242 | -2.539883468066 | -0.257513265752 |
| O | 1.303078224258 | -2.105570139858 | -0.269531050967 |
| N | -2.090385561745 | 0.186383037244 | 0.619725646964 |
| N | -1.409242092329 | -0.796759197459 | -2.231536703477 |
| N | -1.607517582512 | 1.311601782864 | 2.433207023868 |
| C | -0.018563661657 | 0.655837456523 | -0.795312769039 |
| C | -0.099849023645 | -0.673348092150 | -1.561389015589 |
| C | -0.838387575450 | 0.764418392264 | 0.451842205523 |
| C | 0.135295948679 | -1.871167034417 | -0.625809578904 |
| C | -0.535055512072 | 1.464555925938 | 1.584498086911 |
| C | -2.516282113397 | 0.533901372798 | 1.824290124964 |
| H | 1.022684597639 | 0.837025633239 | -0.532220197781 |
| H | -0.304269964003 | 1.447323566598 | -1.495742248184 |
| H | 0.709559337444 | -0.674462774270 | -2.292524552596 |
| H | -1.696319704454 | 1.707157895313 | 3.357421346003 |
| H | 0.327079029499 | 2.049592054573 | 1.850044131834 |
| H | -1.638071340157 | 0.072721582581 | -2.702773408142 |
| H | -1.358970132591 | -1.518736245618 | -2.944104724529 |
| H | -3.447653058562 | 0.238070396970 | 2.275389936807 |

| | | | |
|---|---|---|---|
| Mn | -2.779177480943 | -1.510120256206 | -0.585161102088 |

20

| | | | |
|---|---|---|---|
| O | -0.919040841658 | -2.561410513672 | -0.311424807500 |
| O | 1.307547920976 | -2.283139293950 | -0.478240634485 |
| N | -2.019699608695 | 0.087996894318 | 0.644273733843 |
| N | -1.485478258289 | -0.749941322875 | -2.123629664407 |
| N | -1.706044011422 | 1.389134554136 | 2.364687912443 |
| C | 0.079014775053 | 0.598103523549 | -0.737698717090 |
| C | -0.090662158606 | -0.679389501074 | -1.565872576510 |
| C | -0.805181843467 | 0.750794911267 | 0.467139232508 |
| C | 0.184080815907 | -1.951292441287 | -0.728013387084 |
| C | -0.612435797158 | 1.560990247817 | 1.546891912007 |
| C | -2.528873861069 | 0.495495124667 | 1.802948304630 |
| H | 1.119812326988 | 0.634646683650 | -0.415616850230 |
| H | -0.065545509498 | 1.457056917951 | -1.401651364641 |
| H | 0.637342060750 | -0.651029337539 | -2.376015976665 |
| H | -1.859129442064 | 1.848963793484 | 3.249270822354 |
| H | 0.192295217828 | 2.228492958931 | 1.798053937176 |
| H | -1.769113805548 | 0.154599028331 | -2.493244182935 |
| H | -1.503187052736 | -1.402479676377 | -2.905400281158 |
| H | -3.452960664838 | 0.163791326669 | 2.244539826349 |
| Mn | -2.434347774699 | -1.512841989125 | -0.478197352785 |

20_PCM

| | | | |
|---|---|---|---|
| O | -0.896586140471 | -2.497624941862 | -0.225250422246 |
| O | 1.314709565889 | -2.286309907266 | -0.483054378936 |
| N | -2.001969041279 | 0.076127532391 | 0.644270901099 |
| N | -1.461080356182 | -0.753992663504 | -2.134489517443 |
| N | -1.691289204604 | 1.370736162153 | 2.370644201016 |
| C | 0.072734434734 | 0.605656751627 | -0.746922613222 |
| C | -0.090800316420 | -0.681006507574 | -1.565210904088 |
| C | -0.794858756974 | 0.740188214537 | 0.469990669821 |
| C | 0.161827222260 | -1.930128188647 | -0.697727112774 |
| C | -0.598681719667 | 1.547775253716 | 1.553482408055 |
| C | -2.512959802638 | 0.479776391470 | 1.798985408505 |
| H | 1.116520540192 | 0.678218726983 | -0.443090599856 |
| H | -0.109663847542 | 1.451017711676 | -1.417627549870 |
| H | 0.655760570663 | -0.664832255968 | -2.358886960623 |
| H | -1.848487223394 | 1.829452569526 | 3.254250689312 |
| H | 0.200746374464 | 2.220775786639 | 1.804143904205 |
| H | -1.743961250971 | 0.146602206073 | -2.510243654367 |
| H | -1.484246386976 | -1.409380922242 | -2.911160161137 |
| H | -3.438407483450 | 0.144732876453 | 2.232801980683 |
| Mn | -2.580914689878 | -1.489242907306 | -0.532106402314 |

20_SMD

| | | | |
|---|---|---|---|
| O | -0.859636282983 | -2.568679683294 | -0.281066164361 |
| O | 1.342821643461 | -2.239624563310 | -0.459234552337 |
| N | -2.006687811160 | 0.093670409299 | 0.626431594587 |
| N | -1.474688524355 | -0.751412952486 | -2.109576989183 |
| N | -1.691914971699 | 1.377293050903 | 2.364708881735 |
| C | 0.079544535664 | 0.602906464381 | -0.744930099316 |
| C | -0.101470248926 | -0.682957910516 | -1.558603698228 |
| C | -0.791161663833 | 0.744893130215 | 0.465392033062 |
| C | 0.170052439797 | -1.929692154116 | -0.706375444947 |
| C | -0.591664438960 | 1.543983752189 | 1.554138584373 |
| C | -2.517158111591 | 0.496858099483 | 1.781510746367 |
| H | 1.123109487314 | 0.671977333506 | -0.439642824185 |
| H | -0.103219518843 | 1.440599791001 | -1.424745901046 |
| H | 0.635921411487 | -0.667683904235 | -2.361200087142 |
| H | -1.851973695883 | 1.832518992412 | 3.251193274597 |
| H | 0.215417505880 | 2.204819215989 | 1.814160457378 |
| H | -1.747653294299 | 0.161254322928 | -2.463503509527 |
| H | -1.492121976067 | -1.392090898211 | -2.898625925648 |
| H | -3.449638210815 | 0.170446166787 | 2.208067403698 |
| Mn | -2.619485786439 | -1.530536774056 | -0.515297894055 |

21

| | | | |
|---|---|---|---|
| O | -0.909866546630 | -2.596529454327 | -0.339115164584 |
| O | 1.306924812595 | -2.221585726741 | -0.414480777362 |
| N | -2.088221460427 | 0.137103328342 | 0.662242960961 |
| N | -1.452876455844 | -0.776474685897 | -2.202918816667 |
| N | -1.675789146539 | 1.379601099534 | 2.403892933961 |
| C | 0.020607005916 | 0.604954163989 | -0.752048889644 |
| C | -0.092050917613 | -0.682407539367 | -1.582846108920 |
| C | -0.841636832663 | 0.742493711801 | 0.473996768566 |
| C | 0.182293479762 | -1.941177221147 | -0.717455456486 |
| C | -0.589277609602 | 1.514172467364 | 1.569492212665 |
| C | -2.553283317209 | 0.543586376837 | 1.838987227662 |
| H | 1.061602914497 | 0.691152076072 | -0.441498962384 |
| H | -0.169614282082 | 1.453839225235 | -1.417732803320 |
| H | 0.677719550374 | -0.644443547491 | -2.353296293162 |
| H | -1.789631534294 | 1.827625130952 | 3.300607483746 |

| | | | |
|---|---|---|---|
| H | 0.251989245313 | 2.135604819282 | 1.819248035693 |
| H | -1.724608672064 | 0.115714464172 | -2.607799558848 |
| H | -1.443347049098 | -1.451290324672 | -2.963817454057 |
| H | -3.485973797257 | 0.251089143914 | 2.290069308548 |
| Fe | -2.416566899385 | -1.504485618989 | -0.462726760541 |

21_PCM

| | | | |
|---|---|---|---|
| O | -0.885548325978 | -2.602796343388 | -0.330014550901 |
| O | 1.318910938976 | -2.265391325817 | -0.472343136780 |
| N | -2.011220629099 | 0.074808790740 | 0.672792270310 |
| N | -1.458383210257 | -0.745581689802 | -2.156175450133 |
| N | -1.695524164501 | 1.403683397053 | 2.373118074388 |
| C | 0.067456860798 | 0.589163137100 | -0.732203904841 |
| C | -0.099264558112 | -0.686400543703 | -1.569860476084 |
| C | -0.807054087408 | 0.738337042223 | 0.479897883413 |
| C | 0.163323214396 | -1.955405554107 | -0.728486443118 |
| C | -0.607121986036 | 1.566777198625 | 1.548029488694 |
| C | -2.515595587423 | 0.497993857295 | 1.822580588657 |
| H | 1.109002773479 | 0.644861469700 | -0.417148643987 |
| H | -0.094384027507 | 1.441399816697 | -1.399248812645 |
| H | 0.661212597431 | -0.658549737839 | -2.350913234473 |
| H | -1.851743555530 | 1.880964430401 | 3.246981573389 |
| H | 0.192406076423 | 2.245225662989 | 1.783260782237 |
| H | -1.746739195046 | 0.167463082476 | -2.493076259283 |
| H | -1.473850214762 | -1.370468204484 | -2.955794926209 |
| H | -3.439315533568 | 0.170878307563 | 2.266181070553 |
| Fe | -2.558174898519 | -1.558420904861 | -0.484776007366 |

21_SMD

| | | | |
|---|---|---|---|
| O | -0.889370786747 | -2.563440257398 | -0.286004709691 |
| O | 1.310029028923 | -2.190041196519 | -0.365031627321 |
| N | -2.054582419166 | 0.130725709873 | 0.643356449285 |
| N | -1.434289634750 | -0.778350038178 | -2.196049289830 |
| N | -1.650039933585 | 1.357059246251 | 2.406535818965 |
| C | 0.020290167105 | 0.623339007830 | -0.765751503230 |
| C | -0.101520300770 | -0.681233601804 | -1.565745150647 |
| C | -0.823749239545 | 0.748996300643 | 0.465750908270 |
| C | 0.145635704168 | -1.907448948538 | -0.674966080121 |
| C | -0.567825589793 | 1.510903490899 | 1.569926159476 |
| C | -2.517396316522 | 0.517617145880 | 1.822571185870 |
| H | 1.063675183125 | 0.750743570128 | -0.479431628926 |
| H | -0.214224216303 | 1.440854037933 | -1.454702322357 |
| H | 0.685444449059 | -0.670171534453 | -2.320638116332 |
| H | -1.771479661596 | 1.792922147968 | 3.308710911466 |
| H | 0.267037708580 | 2.138821893370 | 1.824078537774 |
| H | -1.692680702110 | 0.119608109455 | -2.593750716518 |
| H | -1.403860072563 | -1.445923748475 | -2.960548730541 |
| H | -3.446965785679 | 0.208993881951 | 2.268401450298 |
| Fe | -2.655735094083 | -1.525433327943 | -0.543911660059 |

22

| | | | |
|---|---|---|---|
| O | -0.927277466281 | -2.495825820112 | -0.248333509229 |
| O | 1.295832341611 | -2.356328358258 | -0.555280883872 |
| N | -1.954134182162 | 0.039072528530 | 0.626070998345 |
| N | -1.509595358703 | -0.726167065091 | -2.046317468799 |
| N | -1.738344378627 | 1.383756649037 | 2.323274121389 |
| C | 0.132643712550 | 0.592204918680 | -0.731185122317 |
| C | -0.085982486627 | -0.675702089639 | -1.559020381747 |
| C | -0.770092911319 | 0.750682364921 | 0.457637262122 |
| C | 0.182889941288 | -1.952320926410 | -0.733522423091 |
| C | -0.638386265989 | 1.591060839018 | 1.523856044339 |
| C | -2.510418343705 | 0.442479481182 | 1.764477241528 |
| H | 1.171083726640 | 0.587797020939 | -0.398620568679 |
| H | 0.024589323012 | 1.460723324649 | -1.389145158339 |
| H | 0.603498590626 | -0.660004261847 | -2.402271043392 |
| H | -1.931317404515 | 1.853656168713 | 3.194511667585 |
| H | 0.129200365366 | 2.300403365218 | 1.776412613834 |
| H | -1.810621065476 | 0.186399352259 | -2.383917720399 |
| H | -1.578186371053 | -1.363636559441 | -2.839074553204 |
| H | -3.427304332328 | 0.075997982721 | 2.193200831014 |
| Fe | -2.389684946560 | -1.455707026203 | -0.469952061268 |

22_PCM

| | | | |
|---|---|---|---|
| O | -0.914470515967 | -2.459381887179 | -0.202852436289 |
| O | 1.292542515006 | -2.362114631975 | -0.549822922696 |
| N | -1.929556954240 | 0.023698107624 | 0.621761840179 |
| N | -1.496596311748 | -0.721528946253 | -2.040195030292 |
| N | -1.731375235519 | 1.371420727387 | 2.320174305476 |
| C | 0.137469484771 | 0.596871992179 | -0.736077286659 |
| C | -0.089457690550 | -0.676351759560 | -1.555238221216 |
| C | -0.756725329202 | 0.745041560498 | 0.457916246082 |
| C | 0.157427523163 | -1.938106967783 | -0.712333397036 |
| C | -0.632048134589 | 1.588883608584 | 1.524369856166 |
| C | -2.492636571462 | 0.423081489896 | 1.754399964819 |

H 1.178934632149 0.612559901717 -0.415767074004

H 0.007444956372 1.455057093926 -1.401619036305

H 0.607300660478 -0.672805951093 -2.392160113239

H -1.933902220667 1.841181080461 3.188323323245

H 0.123928372651 2.309957348239 1.775552030826

H -1.797028964596 0.190440597014 -2.375965090118

H -1.574643605029 -1.357627285802 -2.831118787371

H -3.408290315908 0.046301404286 2.173986830990

Fe -2.479923807361 -1.438035593296 -0.500535116737

22_SMD

O -0.889892946945 -2.488035844811 -0.214035432636

O 1.308911084364 -2.375460867628 -0.587194010031

N -1.926049651244 0.013428530097 0.628310351822

N -1.501141533180 -0.712182484227 -2.024672268066

N -1.741696751599 1.386356062371 2.311760576609

C 0.145198718814 0.585246389832 -0.725879897663

C -0.093153960418 -0.680019095850 -1.552599614814

C -0.755339633002 0.738822420825 0.461104335156

C 0.161601019883 -1.945798938118 -0.731595109100

C -0.639575406505 1.596971755214 1.516454889396

C -2.493904091556 0.426120624112 1.753619094598

H 1.185158034950 0.595694584459 -0.400368582569

H 0.016924052116 1.440341367842 -1.395558588798

H 0.597662365979 -0.670620658517 -2.394438320029

H -1.952215834814 1.866432780370 3.174091158495

H 0.112357743011 2.325396552567 1.760189821669

H -1.786764848136 0.214703300904 -2.332055662532

H -1.579144847781 -1.330396408965 -2.829836562060

H -3.410344494181 0.051193707169 2.174174263918

Fe -2.490196532009 -1.459651888777 -0.488670557539

23

O -0.912928769105 -2.598604872647 -0.330244621046

O 1.305445635525 -2.264126389108 -0.463493692837

N -2.038111884875 0.097774628972 0.657953583490

N -1.477643957273 -0.748004545556 -2.157057522752

N -1.700270345463 1.392252644172 2.375979858620

C 0.061747414084 0.594640630969 -0.738394305923

C -0.096858091657 -0.683014966147 -1.571723962546

C -0.818892386115 0.749003453368 0.470494257010

C 0.177708160205 -1.957538096478 -0.729318617323

C -0.611636892825 1.555843757496 1.550456354068

C -2.534521165803 0.505774040939 1.820394863230

H 1.102016644457 0.637444481536 -0.415809338315

H -0.087259900574 1.453024730556 -1.402042842584

H 0.648619141687 -0.654779782885 -2.365744058500

H -1.843868790655 1.854018520087 3.261240106291

H 0.200670342436 2.216215770688 1.796059692031

H -1.755232589965 0.155873475148 -2.530152361364

H -1.491355050619 -1.407320639944 -2.931485869179

H -3.458473147696 0.182172073309 2.268015680629

Co -2.400761878025 -1.502107025605 -0.462327317178

23_PCM

O -0.865136888317 -2.629614501408 -0.363064036462

O 1.337743911940 -2.302394563628 -0.553009958533

N -1.971425389466 0.046420731250 0.670725143279

N -1.485385818215 -0.720069287141 -2.092819637437

N -1.729518473271 1.423727317930 2.340809823450

C 0.112088267931 0.571294366043 -0.710247832782

C -0.101723140508 -0.684697042474 -1.563225940323

C -0.783652413628 0.737656835264 0.483158903483

C 0.176056086989 -1.978270475386 -0.765868142504

C -0.630897906368 1.599651259044 1.531948724460

C -2.512847421309 0.481742307384 1.799513501232

H 1.150184039734 0.575671790189 -0.378829790390

H -0.001864880756 1.439203768606 -1.366228731570

H 0.623354350793 -0.649080910863 -2.376508191205

H -1.915754813341 1.916625991874 3.200041900647

H 0.142300555088 2.308877756750 1.763965148022

H -1.781777453007 0.198505601567 -2.406863020725

H -1.540609595110 -1.339766961718 -2.895181584961

H -3.435183044118 0.137600759038 2.232760503976

Co -2.517557487320 -1.554542853447 -0.448276895845

23_SMD

O -0.823854904216 -2.561549458887 -0.252270331814

O 1.370588046129 -2.203037466663 -0.455824889765

N -2.073413655643 0.178938323955 0.582518569028

N -1.463829853851 -0.777415339923 -2.122834251021

N -1.674943655771 1.349533192786 2.383195800900

C 0.047599223928 0.621609778882 -0.762880115832

C -0.100000223896 -0.678721002120 -1.562859839579

| | | | |
|---|---|---|---|
| C | -0.816063895399 | 0.756828206823 | 0.452150757334 |
| C | 0.191099992654 | -1.911749800602 | -0.695338094281 |
| C | -0.566145090105 | 1.485833355673 | 1.578312288050 |
| C | -2.553960853578 | 0.552988931026 | 1.760027830944 |
| H | 1.089650316789 | 0.725617601481 | -0.462976447079 |
| H | -0.163368292377 | 1.444535245801 | -1.452823819631 |
| H | 0.644984438337 | -0.658405600782 | -2.358409875284 |
| H | -1.804399178644 | 1.768056353334 | 3.292571073905 |
| H | 0.282373612364 | 2.079038902710 | 1.868154189859 |
| H | -1.750984030183 | 0.120684100359 | -2.500245084583 |
| H | -1.473933391599 | -1.444823540686 | -2.888450320589 |
| H | -3.502111000541 | 0.261252061521 | 2.177049091839 |
| Co | -2.590895116649 | -1.530671955816 | -0.476266646580 |

24

| | | | |
|---|---|---|---|
| O | -1.033175345385 | -2.380350180473 | -0.207437475037 |
| O | 1.199914711030 | -2.296209385906 | -0.337394802100 |
| N | -2.044854947109 | 0.119603693842 | 0.639216597298 |
| N | -1.424881555167 | -0.808810734318 | -2.212385616939 |
| N | -1.645590557861 | 1.315124296116 | 2.414828517386 |
| C | 0.011447236934 | 0.654498882258 | -0.787341017008 |
| C | -0.073915752445 | -0.665716626973 | -1.568995716891 |
| C | -0.824636837425 | 0.766729029902 | 0.455771512376 |
| C | 0.121929097276 | -1.878387675442 | -0.644041808182 |
| C | -0.577084622655 | 1.512557121652 | 1.570057738988 |
| C | -2.505237570635 | 0.467529453669 | 1.833589229222 |
| H | 1.055959510134 | 0.795368629635 | -0.509964552809 |
| H | -0.232930336184 | 1.472726791889 | -1.473773078818 |
| H | 0.712413234368 | -0.670701747472 | -2.322784368330 |
| H | -1.760643245508 | 1.729753872537 | 3.326973530961 |
| H | 0.248509706149 | 2.151050478668 | 1.828767521573 |
| H | -1.701286896560 | 0.054424504164 | -2.674713259649 |
| H | -1.392365198053 | -1.530047585930 | -2.930193697240 |
| H | -3.419075437738 | 0.126350697526 | 2.288645335647 |
| Co | -2.446102705420 | -1.356951626474 | -0.586024704620 |

24_PCM

| | | | |
|---|---|---|---|
| O | -0.995831724155 | -2.404340212090 | -0.201345327737 |
| O | 1.222995100550 | -2.326610621075 | -0.396837038874 |
| N | -1.991566761675 | 0.073515415646 | 0.645153654716 |
| N | -1.437432211566 | -0.778861225665 | -2.168838957195 |
| N | -1.668982893340 | 1.342509304096 | 2.387021550247 |
| C | 0.052583555166 | 0.632678009686 | -0.767802386477 |
| C | -0.081975190614 | -0.672891080642 | -1.565295577552 |
| C | -0.797144681257 | 0.753878742954 | 0.463209948947 |
| C | 0.115466900176 | -1.896553725667 | -0.663087003661 |
| C | -0.592660815318 | 1.547618021082 | 1.556231098880 |
| C | -2.487933799151 | 0.446919020603 | 1.813439392031 |
| H | 1.098583124273 | 0.742034271174 | -0.483051529121 |
| H | -0.165105154004 | 1.459050611060 | -1.451181372257 |
| H | 0.689440365822 | -0.678752089202 | -2.334434820775 |
| H | -1.819158189838 | 1.786042229235 | 3.279414081912 |
| H | 0.202257635379 | 2.226461818144 | 1.805524233597 |
| H | -1.736661417757 | 0.109722607933 | -2.559919950830 |
| H | -1.436384678530 | -1.451502982849 | -2.930435215522 |
| H | -3.400659519580 | 0.091091017499 | 2.257354292344 |
| Co | -2.501437156831 | -1.423467243048 | -0.582319186863 |

24_SMD

| | | | |
|---|---|---|---|
| O | -0.791304076649 | -2.594728911963 | -0.279717255526 |
| O | 1.401120811253 | -2.269791340405 | -0.589763858282 |
| N | -1.963213124692 | 0.077646232757 | 0.585811105474 |
| N | -1.522209714746 | -0.726905496905 | -1.984784955554 |
| N | -1.742254680298 | 1.377601737108 | 2.316169531282 |
| C | 0.143388945925 | 0.586986066039 | -0.725895118930 |
| C | -0.103713263558 | -0.680715630998 | -1.546308003627 |
| C | -0.755250184259 | 0.744596617967 | 0.460279386364 |
| C | 0.212099652555 | -1.955081747248 | -0.746932145469 |
| C | -0.615319289976 | 1.557512924582 | 1.547454202461 |
| C | -2.533143492865 | 0.479697136719 | 1.713595203707 |
| H | 1.183250744179 | 0.592321844678 | -0.401626714965 |
| H | 0.016811621898 | 1.443417264571 | -1.394043532828 |
| H | 0.550015788372 | -0.653000273069 | -2.416986968628 |
| H | -1.943836658898 | 1.837739601390 | 3.191730160497 |
| H | 0.169110315433 | 2.235435849015 | 1.830827830873 |
| H | -1.823885205064 | 0.189708504792 | -2.309491145433 |
| H | -1.625981535472 | -1.377448714180 | -2.762251642691 |
| H | -3.475589017092 | 0.138036462452 | 2.103984664300 |
| Co | -2.511705148308 | -1.424486238427 | -0.489250857199 |

25

| | | | |
|---|---|---|---|
| O | -0.903056565437 | -2.563068092594 | -0.282852463063 |
| O | 1.313530544523 | -2.300481368429 | -0.519251405133 |
| N | -2.029141556645 | 0.096949583882 | 0.650797782704 |

| | | | |
|---|---|---|---|
| N | -1.481966744358 | -0.751370140891 | -2.138485950030 |
| N | -1.710892941626 | 1.387420337333 | 2.370699867958 |
| C | 0.071513570001 | 0.595418736193 | -0.736350231468 |
| C | -0.092564353434 | -0.681414481983 | -1.570719664750 |
| C | -0.810166856366 | 0.748218957210 | 0.472638388336 |
| C | 0.184188787780 | -1.958379594371 | -0.732668172292 |
| C | -0.615527568336 | 1.555321715260 | 1.554774040516 |
| C | -2.539746946861 | 0.500187211634 | 1.807468084444 |
| H | 1.112324365043 | 0.632950256296 | -0.414538384711 |
| H | -0.074567523449 | 1.456330836697 | -1.397359015469 |
| H | 0.640957586705 | -0.652451498083 | -2.375386739291 |
| H | -1.863486978137 | 1.848165722084 | 3.255024115814 |
| H | 0.191645604164 | 2.219376905477 | 1.807217355342 |
| H | -1.774604509029 | 0.145951861070 | -2.516779435922 |
| H | -1.515529476638 | -1.425027810549 | -2.900171557945 |
| H | -3.466526760878 | 0.171837633754 | 2.245384885195 |
| Ni | -2.367989189272 | -1.447394881119 | -0.476641614407 |

25_PCM

| | | | |
|---|---|---|---|
| O | -0.903436351335 | -2.519168813435 | -0.232243360239 |
| O | 1.305226005033 | -2.295384900663 | -0.487271338642 |
| N | -1.988976674369 | 0.075805999097 | 0.638983617670 |
| N | -1.468890525078 | -0.744930421371 | -2.125147226183 |
| N | -1.699777277440 | 1.368930623271 | 2.363790255867 |
| C | 0.082017143391 | 0.605432458705 | -0.746443387626 |
| C | -0.095854286807 | -0.682328225365 | -1.561393345334 |
| C | -0.788846411844 | 0.747802062592 | 0.467039986429 |
| C | 0.153078833637 | -1.938686086566 | -0.697649968607 |
| C | -0.606488586396 | 1.558201597998 | 1.551240454107 |
| C | -2.510373781848 | 0.468413066137 | 1.790855377198 |
| H | 1.125880051206 | 0.666425434570 | -0.440989506858 |
| H | -0.090109067735 | 1.450998219679 | -1.419178801619 |
| H | 0.644382108982 | -0.674854463028 | -2.360413222716 |
| H | -1.865728193263 | 1.826059797015 | 3.246636653201 |
| H | 0.184508003129 | 2.239788129193 | 1.804926359981 |
| H | -1.760858257843 | 0.155038299825 | -2.493861698134 |
| H | -1.511586956184 | -1.409507582914 | -2.892296156348 |
| H | -3.432617791566 | 0.120539363871 | 2.220913560354 |
| Ni | -2.503155495915 | -1.440032669737 | -0.524698366676 |

25_SMD

| | | | |
|---|---|---|---|
| O | -0.890976190270 | -2.524894336327 | -0.229457407804 |
| O | 1.312813600999 | -2.306480413648 | -0.502406257341 |
| N | -1.999265266341 | 0.081665941678 | 0.637665620309 |
| N | -1.470402661911 | -0.746691912577 | -2.119974338659 |
| N | -1.702453315743 | 1.370365085370 | 2.364749554650 |
| C | 0.079503707812 | 0.602865225406 | -0.744214832832 |
| C | -0.096193722969 | -0.683854341263 | -1.561047986631 |
| C | -0.791351830727 | 0.745067592306 | 0.467793492906 |
| C | 0.156494492591 | -1.936745173601 | -0.708149181002 |
| C | -0.606143231644 | 1.551400440799 | 1.552507748805 |
| C | -2.517665121856 | 0.476432809470 | 1.791303787083 |
| H | 1.122835706739 | 0.673538606575 | -0.439538510619 |
| H | -0.106107669644 | 1.438825721134 | -1.424954874150 |
| H | 0.639955801706 | -0.672478449817 | -2.363206268153 |
| H | -1.867665586953 | 1.827719144876 | 3.249536615455 |
| H | 0.190049338424 | 2.227081045299 | 1.807266095513 |
| H | -1.751242051314 | 0.160579596555 | -2.482084513866 |
| H | -1.508533962266 | -1.413170453330 | -2.887046433287 |
| H | -3.442878814124 | 0.134417850643 | 2.220572908237 |
| Ni | -2.482380734760 | -1.427102090673 | -0.526515332787 |

26

| | | | |
|---|---|---|---|
| O | -0.834823575825 | -2.727348625158 | -0.553176416459 |
| O | 1.357458450953 | -2.295783642530 | -0.535768097140 |
| N | -2.092244371241 | 0.148488128317 | 0.666120321158 |
| N | -1.474955436409 | -0.786674964827 | -2.112736958595 |
| N | -1.726267643344 | 1.440123397353 | 2.379818219515 |
| C | 0.061385712579 | 0.577109976463 | -0.704944816525 |
| C | -0.080251496983 | -0.676307427668 | -1.586220651982 |
| C | -0.833529334719 | 0.737723375272 | 0.500340836845 |
| C | 0.236334158470 | -1.996367902969 | -0.835823494084 |
| C | -0.611362502928 | 1.541571725773 | 1.580129016612 |
| C | -2.592917323459 | 0.592415888016 | 1.810134351328 |
| H | 1.098014613916 | 0.607594236991 | -0.368731690302 |
| H | -0.071495901072 | 1.447446499597 | -1.356038922814 |
| H | 0.632148642830 | -0.581168863240 | -2.405761631073 |
| H | -1.866725639857 | 1.917188994148 | 3.257562589372 |
| H | 0.223546947438 | 2.168713545421 | 1.837337367753 |
| H | -1.846809657146 | 0.102645687764 | -2.434033248798 |
| H | -1.535110852411 | -1.461225995332 | -2.870146897374 |
| H | -3.543031169515 | 0.318479654035 | 2.235344323600 |
| Cu | -2.230971133542 | -1.496081798501 | -0.400604315494 |

26_PCM

| | | | |
|---|---|---|---|
| O | -0.104583928824 | -2.986186368051 | -0.090559862002 |
| O | 1.637534804734 | -1.796449180220 | 0.684978752797 |
| N | -1.792430215708 | 0.043058424120 | 0.633896110955 |
| N | -0.915413527582 | -1.197522229035 | -1.813834659408 |
| N | -1.791073290015 | 1.622051142553 | 2.133358434662 |
| C | 0.382073750237 | 0.517747702847 | -0.589381417238 |
| C | 0.374602018518 | -0.901535417549 | -1.150690856680 |
| C | -0.621961502181 | 0.769431321299 | 0.488516019798 |
| C | 0.715566329121 | -1.925054546803 | -0.075741392024 |
| C | -0.614984865709 | 1.753753774459 | 1.434410918297 |
| C | -2.477353736396 | 0.588645247694 | 1.630919506991 |
| H | 1.379674604975 | 0.730824534529 | -0.210668587259 |
| H | 0.212626194876 | 1.203477170872 | -1.423721927448 |
| H | 1.183891097700 | -0.971053357371 | -1.882688108674 |
| H | -2.090628265977 | 2.203593232091 | 2.900454329801 |
| H | 0.104739886428 | 2.519507383350 | 1.658397366172 |
| H | -1.123935046272 | -0.453498850688 | -2.474922629149 |
| H | -0.830300462675 | -2.050107270172 | -2.359988594805 |
| H | -3.438476293327 | 0.268753861632 | 1.989425998010 |
| H | 0.164202173394 | -3.608102478857 | 0.606575079559 |
| Cu | -2.464736668842 | -1.463258267777 | -0.481451475543 |
| O | -6.718947229908 | -1.026881539321 | -0.911402804289 |
| O | -4.615294635663 | -0.248276065646 | -0.840668342288 |
| N | -3.323620930394 | -2.486676524733 | 1.028096571825 |
| N | -3.578390136994 | -2.539993783956 | -1.831063150606 |
| N | -3.688623428366 | -3.289999707390 | 3.020815270206 |
| C | -5.224458262980 | -3.553792982718 | -0.284791488826 |
| C | -4.997928290923 | -2.557681159976 | -1.426112526432 |
| C | -4.467273500796 | -3.266607418250 | 0.973684372798 |
| C | -5.403842821206 | -1.146991302265 | -1.032998960370 |
| C | -4.702825045521 | -3.762962148972 | 2.223973236522 |
| C | -2.877420181844 | -2.527463595037 | 2.276216686293 |
| H | -6.290619220128 | -3.594125947006 | -0.067902116724 |
| H | -4.950404596704 | -4.542118230202 | -0.663009005272 |
| H | -5.628557099371 | -2.862665206512 | -2.262371429136 |
| H | -3.573401104287 | -3.476991330779 | 4.004756357578 |
| H | -5.480264892729 | -4.402092266582 | 2.600554746731 |
| H | -3.239946300977 | -3.493603957275 | -1.927250533960 |
| H | -3.489409866746 | -2.107159594258 | -2.746438603564 |
| H | -2.001622095343 | -2.031240497593 | 2.652892398848 |
| H | -6.936784355658 | -0.122123720372 | -0.629103226083 |

26_SMD

| | | | |
|---|---|---|---|
| O | -0.105683428553 | -3.151656181890 | -0.180205264732 |
| O | 1.340647189439 | -1.875538717388 | 0.966736775214 |
| N | -1.783437561832 | 0.165097523368 | 0.564268601481 |
| N | -0.850385229017 | -1.204026902439 | -1.819452458783 |
| N | -1.695547529569 | 1.692353375383 | 2.115079524284 |
| C | 0.486173596941 | 0.426819001445 | -0.533485351579 |
| C | 0.407006700575 | -0.998521391582 | -1.070971621276 |
| C | -0.538529611416 | 0.766995353365 | 0.498050833377 |
| C | 0.595744150739 | -2.033692362260 | 0.026116884495 |
| C | -0.477759508127 | 1.719151280197 | 1.473864344124 |
| C | -2.455448977653 | 0.746080528814 | 1.548085979471 |
| H | 1.479004948300 | 0.593426089358 | -0.120725632047 |
| H | 0.385726677692 | 1.096906429021 | -1.391373745091 |
| H | 1.259458477763 | -1.145404870592 | -1.741966838112 |
| H | -1.973898429953 | 2.279344365696 | 2.887858096072 |
| H | 0.305700617582 | 2.398592877705 | 1.756620603083 |
| H | -0.954066222816 | -0.453926927494 | -2.497536510194 |
| H | -0.807846215019 | -2.075458288024 | -2.341409159300 |
| H | -3.457329361789 | 0.511024567242 | 1.860069071874 |
| H | 0.072098835890 | -3.780479464156 | 0.541376438274 |
| Cu | -2.479255155815 | -1.355619391614 | -0.549302708070 |
| O | -6.927261175555 | -1.448857771305 | -0.570859359363 |
| O | -5.121912156748 | -0.181386534596 | -0.991979092363 |
| N | -3.302376918677 | -2.396359225291 | 0.982123513147 |
| N | -3.667756366291 | -2.301778281310 | -1.916515308388 |
| N | -3.704962328478 | -3.128604939361 | 2.995537348021 |
| C | -5.046890990136 | -3.642081395933 | -0.364591415918 |
| C | -5.039649214167 | -2.559099022794 | -1.447580555804 |
| C | -4.386377070926 | -3.255405092533 | 0.916380612152 |
| C | -5.671069755416 | -1.260842872215 | -0.983030567735 |
| C | -4.647526292477 | -3.708045458384 | 2.177312807263 |
| C | -2.914263286980 | -2.345577411144 | 2.247775031138 |
| H | -6.074968688404 | -3.936188843895 | -0.164470242133 |
| H | -4.545679532150 | -4.515737261445 | -0.790602063672 |
| H | -5.654641596492 | -2.919214033796 | -2.276233549874 |
| H | -3.619225964084 | -3.263342078166 | 3.992480396615 |
| H | -5.397540677252 | -4.381350909512 | 2.551149839024 |
| H | -3.248243381725 | -3.181003059001 | -2.208561854875 |
| H | -3.688673557141 | -1.695622978872 | -2.733455124208 |
| H | -2.093086209947 | -1.775237192056 | 2.642899460293 |

| | | | |
|---|---|---|---|
| H | -7.300869740355 | -0.597103682477 | -0.284317275819 |

27

| | | | |
|---|---|---|---|
| O | -0.864626618300 | -2.654118315355 | -0.326848695261 |
| O | 1.338359368141 | -2.269126610516 | -0.540285298230 |
| N | -2.084115201362 | 0.128023645413 | 0.689140258214 |
| N | -1.471030546986 | -0.755823571602 | -2.203714631628 |
| N | -1.704478744956 | 1.410311697317 | 2.400438342142 |
| C | 0.031263307171 | 0.582704424715 | -0.728062915472 |
| C | -0.101574571669 | -0.691587816591 | -1.581080014453 |
| C | -0.842180221114 | 0.742656342119 | 0.491770845044 |
| C | 0.189725498433 | -1.989809960837 | -0.753675609812 |
| C | -0.614046891406 | 1.543854583773 | 1.571360110381 |
| C | -2.566010779271 | 0.547324306873 | 1.851483895364 |
| H | 1.070721283262 | 0.630759125175 | -0.403939448960 |
| H | -0.123529622187 | 1.445949920508 | -1.383962301485 |
| H | 0.658476001191 | -0.647121968025 | -2.359247642945 |
| H | -1.832235041273 | 1.877776906658 | 3.285406693090 |
| H | 0.214570670745 | 2.185485397686 | 1.812408291043 |
| H | -1.738324281299 | 0.137688583395 | -2.607376507805 |
| H | -1.481092363305 | -1.443835536485 | -2.951916553306 |
| H | -3.495946571764 | 0.245903147292 | 2.301715521761 |
| Zn | -2.315532186305 | -1.448472412642 | -0.460814451862 |

27_PCM

| | | | |
|---|---|---|---|
| O | -0.867069006303 | -2.596456460968 | -0.294062584091 |
| O | 1.333350931991 | -2.256192157645 | -0.489885522822 |
| N | -2.030381384225 | 0.092924648313 | 0.676447586814 |
| N | -1.455529217450 | -0.755973172728 | -2.176071927730 |
| N | -1.690467756077 | 1.390425391030 | 2.388982816157 |
| C | 0.048405963960 | 0.593276500110 | -0.736961055687 |
| C | -0.101262397394 | -0.689312088703 | -1.569789200633 |
| C | -0.813473600647 | 0.735799328511 | 0.485934623762 |
| C | 0.164925787703 | -1.957200210198 | -0.718795942346 |
| C | -0.601130564384 | 1.546914298482 | 1.564198125121 |
| C | -2.528249834022 | 0.506621474026 | 1.830774310657 |
| H | 1.091483715399 | 0.666092573170 | -0.431412013725 |
| H | -0.133477473058 | 1.441954689790 | -1.403373493640 |
| H | 0.661213175469 | -0.662836318973 | -2.347453897023 |
| H | -1.836860523966 | 1.859367877522 | 3.269161999355 |
| H | 0.208781876323 | 2.210798643694 | 1.805177519630 |
| H | -1.731154097250 | 0.141487883965 | -2.561350764051 |
| H | -1.472120085503 | -1.426210400015 | -2.937885061612 |
| H | -3.455813431295 | 0.187533177124 | 2.271393021669 |
| Zn | -2.522779591528 | -1.450473787640 | -0.522228653997 |

27_SMD

| | | | |
|---|---|---|---|
| O | -0.823007421181 | -2.550947636945 | -0.215649283333 |
| O | 1.367411863782 | -2.188170552640 | -0.444392893880 |
| N | -2.094383811715 | 0.184759155442 | 0.605550729007 |
| N | -1.452588637633 | -0.788521044092 | -2.168693850881 |
| N | -1.660400441916 | 1.344716198696 | 2.402868822470 |
| C | 0.018615294301 | 0.620960993184 | -0.767610846456 |
| C | -0.104550052866 | -0.684107471418 | -1.567280892939 |
| C | -0.831331704958 | 0.748772153900 | 0.459310609148 |
| C | 0.182908283299 | -1.908397836688 | -0.680032949533 |
| C | -0.560621827597 | 1.471356418263 | 1.585067176885 |
| C | -2.556007585938 | 0.559176943504 | 1.788808912641 |
| H | 1.060879166397 | 0.743715668373 | -0.475742257716 |
| H | -0.211662580930 | 1.439297483928 | -1.456764539965 |
| H | 0.658931410917 | -0.665523081471 | -2.344194354990 |
| H | -1.775541953834 | 1.765089209896 | 3.313354206722 |
| H | 0.297388859657 | 2.055613115710 | 1.865021206381 |
| H | -1.734158952419 | 0.107312054721 | -2.555351452306 |
| H | -1.440109364487 | -1.459004107367 | -2.930964745287 |
| H | -3.502750217393 | 0.278256453584 | 2.215870089298 |
| Zn | -2.570627837746 | -1.495812229716 | -0.526373799452 |

28

| | | | |
|---|---|---|---|
| O | -0.741695596617 | -2.019574188006 | 0.888538087422 |
| O | 1.454375278384 | -1.537993580888 | 0.954876408882 |
| N | -1.969948386924 | 0.728776274638 | 0.201529300368 |
| N | -0.952949562792 | -1.416148281062 | -1.704662156258 |
| N | -1.652247591163 | 2.380308321051 | 1.599625026692 |
| C | 0.284924061987 | 0.587673785916 | -0.935853003930 |
| C | 0.268452924275 | -0.947061060350 | -1.012072799410 |
| C | -0.676018583727 | 1.190439002477 | 0.037998696201 |
| C | 0.358314565365 | -1.562415260120 | 0.414023315081 |
| C | -0.467342751759 | 2.221030387642 | 0.909873812604 |
| C | -2.526911905910 | 1.455214313904 | 1.150512777774 |
| H | 1.292957496826 | 0.889906207455 | -0.654484963509 |
| H | 0.103412344169 | 0.989193416257 | -1.940863222288 |
| H | 1.169240976254 | -1.256040668742 | -1.546990964610 |
| H | -1.822199375847 | 3.037193284087 | 2.341619435878 |

| | | | |
|---|---|---|---|
| H | 0.399460963933 | 2.824970388969 | 1.108926433778 |
| H | -1.096264790836 | -0.901633574042 | -2.567561824551 |
| H | -0.851579663132 | -2.395269759244 | -1.950979684744 |
| H | -3.515513048326 | 1.292027592614 | 1.546433366994 |
| Mn | -2.571106749672 | -1.445898821239 | -0.008649163071 |
| O | -4.309236352130 | -0.927702419383 | 1.082719194585 |
| O | -6.491975833915 | -1.415030201502 | 1.321890734786 |
| N | -3.156519819354 | -3.630258462004 | 0.133846327645 |
| N | -4.337648417542 | -1.384103798599 | -1.548600462870 |
| N | -3.351063294530 | -5.355282302790 | 1.464170579945 |
| C | -5.505625910642 | -3.430497498553 | -0.782538142198 |
| C | -5.492108830451 | -1.893828668695 | -0.774706598294 |
| C | -4.461782052124 | -4.083900957846 | 0.065122651067 |
| C | -5.449602593375 | -1.359057482022 | 0.685650140396 |
| C | -4.593614033307 | -5.160261859144 | 0.895693254866 |
| C | -2.517709985555 | -4.406327318874 | 0.987167718639 |
| H | -6.484720988956 | -3.750291699837 | -0.427918727389 |
| H | -5.417149184652 | -3.775324096457 | -1.820543524388 |
| H | -6.437090814410 | -1.557721595596 | -1.207205454049 |
| H | -3.116335404522 | -6.051045825216 | 2.151012244059 |
| H | -5.440853044246 | -5.774994760000 | 1.140643785270 |
| H | -4.275470711779 | -1.848902596783 | -2.448577078772 |
| H | -4.457945682453 | -0.392843387756 | -1.729059520016 |
| H | -1.496365330537 | -4.263702690293 | 1.298967957432 |

28_PCM

| | | | |
|---|---|---|---|
| O | -0.690156537656 | -1.941297589903 | 1.228782093411 |
| O | 1.486230755175 | -1.414703624018 | 1.227929550517 |
| N | -1.948726769772 | 0.708098853127 | 0.266583746369 |
| N | -0.937459590107 | -1.622290201355 | -1.420616010827 |
| N | -1.730497379503 | 2.686476343626 | 1.176819245238 |
| C | 0.290278897269 | 0.459948930307 | -0.910244744618 |
| C | 0.280413162027 | -1.072851062254 | -0.797259124585 |
| C | -0.691868277702 | 1.194388151221 | -0.050607087573 |
| C | 0.380723312503 | -1.518033827009 | 0.681434065609 |
| C | -0.548145833220 | 2.429588963782 | 0.520001509836 |
| C | -2.543412068528 | 1.626568580864 | 1.006531913079 |
| H | 1.291448678987 | 0.811677834705 | -0.664189529234 |
| H | 0.125049642357 | 0.716215335965 | -1.962418863692 |
| H | 1.179249347628 | -1.436717574390 | -1.299873266825 |
| H | -1.946110723743 | 3.516393765946 | 1.705345992840 |
| H | 0.267456121417 | 3.130033326938 | 0.514668018817 |
| H | -1.105688482444 | -1.186768876590 | -2.321305087558 |
| H | -0.825387618128 | -2.615914028348 | -1.593153235006 |
| H | -3.526214240019 | 1.540796315565 | 1.436042284950 |
| Mn | -2.556091329284 | -1.457389348981 | 0.213303512569 |
| O | -4.319393587227 | -1.029067431264 | 1.424623339017 |
| O | -6.492652715959 | -1.543281322353 | 1.588920140799 |
| N | -3.170646124431 | -3.624296742927 | 0.191012375705 |
| N | -4.315017477772 | -1.195087804699 | -1.251493636532 |
| N | -3.327750587359 | -5.641769095488 | 1.025085825299 |
| C | -5.508078533827 | -3.299219407988 | -0.751183453023 |
| C | -5.476167602853 | -1.775288653332 | -0.552350136053 |
| C | -4.458622159797 | -4.083582059958 | -0.026075577415 |
| C | -5.439155827169 | -1.414819995106 | 0.952469488054 |
| C | -4.563843577476 | -5.343223666892 | 0.497467991541 |
| C | -2.520627059134 | -4.582258743222 | 0.827150450682 |
| H | -6.485732253906 | -3.662561090999 | -0.436949789861 |
| H | -5.439241704321 | -3.494771123661 | -1.826908752988 |
| H | -6.413448169383 | -1.380570983780 | -0.950578439865 |
| H | -3.073815998754 | -6.497848623207 | 1.491178181943 |
| H | -5.385630019078 | -6.035194585096 | 0.537920799059 |
| H | -4.232719288082 | -1.578817973872 | -2.186993150467 |
| H | -4.433500322654 | -0.192830121782 | -1.356303802006 |
| H | -1.500505738066 | -4.526110683596 | 1.164807122835 |

28_SMD

| | | | |
|---|---|---|---|
| O | -0.533794628311 | -2.040151681191 | 0.805674112888 |
| O | 1.633510019433 | -1.494773433250 | 0.740176017421 |
| N | -1.901490550285 | 0.684104984799 | 0.391569519741 |
| N | -0.959091907532 | -1.336624537647 | -1.746814955934 |
| N | -1.513473127303 | 2.356253156182 | 1.755264643854 |
| C | 0.246527544344 | 0.670199837012 | -0.969399734069 |
| C | 0.283474475387 | -0.859194878459 | -1.120070601182 |
| C | -0.641636758377 | 1.193680194143 | 0.112392756923 |
| C | 0.483516327217 | -1.526003763644 | 0.249630062221 |
| C | -0.393837723865 | 2.233656277437 | 0.963558451691 |
| C | -2.391038571565 | 1.405573748387 | 1.385556295330 |
| H | 1.258866202729 | 1.024053612178 | -0.778345526983 |
| H | -0.053304614912 | 1.085128140414 | -1.937263553827 |
| H | 1.153138729783 | -1.106759991232 | -1.731905373923 |
| H | -1.651056550457 | 3.031945672161 | 2.491752102526 |
| H | 0.457873022380 | 2.881596800871 | 1.069685791879 |
| H | -1.170934555752 | -0.771089578782 | -2.562608102604 |

| | | | |
|---|---|---|---|
| H | -0.831700151865 | -2.289830641879 | -2.072736371997 |
| H | -3.347097934672 | 1.263360593378 | 1.857442664346 |
| Mn | -2.570063375645 | -1.439133088756 | -0.098877619926 |
| O | -4.491180905278 | -0.950723451447 | 1.042871171720 |
| O | -6.680479239024 | -1.400178787705 | 1.106320901185 |
| N | -3.202303650658 | -3.635128417495 | 0.278468709130 |
| N | -4.313386722154 | -1.470830453077 | -1.598172273175 |
| N | -3.494675258495 | -5.334620385858 | 1.633841941793 |
| C | -5.484804586602 | -3.502721701361 | -0.825075573840 |
| C | -5.503768866234 | -1.966431463174 | -0.887636884928 |
| C | -4.502399033163 | -4.095005088768 | 0.131382429672 |
| C | -5.569743915907 | -1.382807521795 | 0.532813578757 |
| C | -4.691088281189 | -5.151795316788 | 0.977168666589 |
| C | -2.631060318191 | -4.401531545762 | 1.191776159305 |
| H | -6.479983804700 | -3.849919765948 | -0.549905157830 |
| H | -5.293015877234 | -3.867853245947 | -1.839264184683 |
| H | -6.419361892629 | -1.671296022007 | -1.403324346946 |
| H | -3.298789488033 | -6.036817490336 | 2.331434487634 |
| H | -5.548592121238 | -5.773415183513 | 1.163701417233 |
| H | -4.171741413705 | -2.009061465478 | -2.446882271411 |
| H | -4.463711093951 | -0.507264842554 | -1.881181006529 |
| H | -1.623757082345 | -4.304969113154 | 1.556555617934 |

29

| | | | |
|---|---|---|---|
| O | -0.969596311698 | -2.556672042584 | -0.828655703221 |
| O | 1.248841946581 | -2.327191564153 | -1.110833178493 |
| N | -1.753784291067 | -0.179622941384 | 0.757659234000 |
| N | -1.551853330128 | -0.255007659791 | -1.997789906515 |
| N | -1.633372085590 | 1.137306804972 | 2.504033587929 |
| C | 0.306267310248 | 0.524518391329 | -0.545705649022 |
| C | -0.127485785708 | -0.458709533371 | -1.633445261564 |
| C | -0.607639317448 | 0.588685988171 | 0.642691489603 |
| C | 0.097273963938 | -1.920985130364 | -1.154724712686 |
| C | -0.526347310920 | 1.412857908576 | 1.731545342603 |
| C | -2.350220972213 | 0.174483731979 | 1.881215510885 |
| H | 1.311638217628 | 0.230686509071 | -0.239292503471 |
| H | 0.389798837311 | 1.527215624553 | -0.977413161513 |
| H | 0.528848014538 | -0.303259473036 | -2.491823918582 |
| H | -1.884059691100 | 1.591944392345 | 3.364238637288 |
| H | 0.201037990323 | 2.152533177412 | 2.013853918095 |
| H | -1.824049033825 | 0.722269427823 | -1.944880250363 |
| H | -1.705266796668 | -0.548329552214 | -2.956885855191 |

| | | | |
|---|---|---|---|
| H | -3.292842732612 | -0.205546247381 | 2.231262616168 |
| Mn | -2.597703696616 | -1.428312464845 | -0.606004537458 |
| O | -4.236465294961 | -0.293163323600 | -0.615056242753 |
| O | -6.471821744226 | -0.510825346817 | -0.712845946613 |
| N | -3.320108605479 | -2.756285155094 | 0.752481106787 |
| N | -3.766816308356 | -2.520268033053 | -1.965356938448 |
| N | -3.290816331421 | -4.169745844245 | 2.425769970023 |
| C | -5.489787432619 | -3.388513079161 | -0.401357348299 |
| C | -5.152462968389 | -2.342116904519 | -1.464057252039 |
| C | -4.474856435383 | -3.518207015472 | 0.696259738568 |
| C | -5.329823870857 | -0.911046072064 | -0.882304584575 |
| C | -4.462873014804 | -4.402469560437 | 1.740007666709 |
| C | -2.628066259579 | -3.172548897110 | 1.797126538748 |
| H | -6.463857631858 | -3.116947489340 | 0.009024770905 |
| H | -5.612307952610 | -4.364721100958 | -0.881779715267 |
| H | -5.883177914876 | -2.448930834729 | -2.268045820721 |
| H | -2.966971888107 | -4.671706294868 | 3.233637820488 |
| H | -5.165383646740 | -5.157632552782 | 2.043506973120 |
| H | -3.493665765442 | -3.498267632840 | -1.994779204226 |
| H | -3.698733801255 | -2.170367251027 | -2.915289590852 |
| H | -1.656945738008 | -2.811482798994 | 2.083586319955 |

29_PCM

| | | | |
|---|---|---|---|
| O | -0.893846647728 | -2.495363491062 | -0.817165991741 |
| O | 1.276814550537 | -2.240856740521 | -1.298657438443 |
| N | -1.735812092127 | -0.175116930476 | 0.784684348846 |
| N | -1.613226714442 | -0.240562336895 | -2.012797299288 |
| N | -1.430403961800 | 0.981978958296 | 2.621761338800 |
| C | 0.237816484694 | 0.616869892922 | -0.615931855420 |
| C | -0.175243784360 | -0.385233563452 | -1.695026168192 |
| C | -0.580414477388 | 0.577669429795 | 0.641433260492 |
| C | 0.112554507627 | -1.831433115058 | -1.240386132087 |
| C | -0.383526330749 | 1.296630960312 | 1.789199297196 |
| C | -2.222530363023 | 0.096257225487 | 1.985922153066 |
| H | 1.288912037772 | 0.443095165231 | -0.382135623509 |
| H | 0.182422001949 | 1.621436919730 | -1.045694705817 |
| H | 0.444319045923 | -0.191355882992 | -2.572143029322 |
| H | -1.580621961022 | 1.344965413444 | 3.548706326101 |
| H | 0.385663370018 | 1.991886749476 | 2.073111044180 |
| H | -1.887387068311 | 0.737113185416 | -1.995114833654 |
| H | -1.783868667554 | -0.560940314105 | -2.961092124567 |
| H | -3.120625863442 | -0.315517913912 | 2.408580785391 |

| | | | |
|---|---|---|---|
| Mn | -2.598328528904 | -1.427192220477 | -0.587727572690 |
| O | -4.313883797967 | -0.352386626122 | -0.608986387327 |
| O | -6.519765860241 | -0.589483186894 | -0.902309531439 |
| N | -3.339194791595 | -2.757265751396 | 0.781595233089 |
| N | -3.707247745583 | -2.534266822644 | -1.985778475876 |
| N | -3.479327387106 | -4.021503868982 | 2.567390333502 |
| C | -5.428846089801 | -3.477019279770 | -0.482505536553 |
| C | -5.111311193964 | -2.413204543006 | -1.534590849455 |
| C | -4.502465042109 | -3.506720162210 | 0.697607364574 |
| C | -5.355391585987 | -0.996279486754 | -0.974362044261 |
| C | -4.596053308604 | -4.292644409736 | 1.814050825788 |
| C | -2.747197636248 | -3.095778652203 | 1.916602744799 |
| H | -6.454876312884 | -3.320668359086 | -0.147185144826 |
| H | -5.412850326866 | -4.455148694399 | -0.972207485470 |
| H | -5.806129121162 | -2.558571296596 | -2.363385048676 |
| H | -3.246498833254 | -4.438302049314 | 3.453758401922 |
| H | -5.336637603792 | -5.007734005616 | 2.123596364268 |
| H | -3.434844594720 | -3.510507989720 | -2.049245870823 |
| H | -3.620473036873 | -2.158779256289 | -2.925117044926 |
| H | -1.815128948911 | -2.704446790422 | 2.281120332355 |

29_SMD

| | | | |
|---|---|---|---|
| O | -0.821590066325 | -2.481329836104 | -0.812894928038 |
| O | 1.337470195742 | -2.169495941598 | -1.289968955598 |
| N | -1.718903129292 | -0.161935774551 | 0.793806731327 |
| N | -1.603513335647 | -0.248988033467 | -2.036932231654 |
| N | -1.339333036195 | 0.925905999852 | 2.658513639162 |
| C | 0.214506877993 | 0.670068755424 | -0.644897703670 |
| C | -0.165627414137 | -0.356007011828 | -1.713611636999 |
| C | -0.560238470780 | 0.585371531935 | 0.636033338495 |
| C | 0.157021299427 | -1.781262256780 | -1.243462541240 |
| C | -0.316982827008 | 1.258380240915 | 1.801187695637 |
| C | -2.160892783820 | 0.071571897314 | 2.020918556299 |
| H | 1.280211837476 | 0.571202181380 | -0.436634901931 |
| H | 0.075059349563 | 1.663011462669 | -1.082044312014 |
| H | 0.450926489429 | -0.156216546182 | -2.591007975178 |
| H | -1.455003540641 | 1.257259226837 | 3.603976665391 |
| H | 0.470429577080 | 1.934261062198 | 2.082944207917 |
| H | -1.878594975995 | 0.728479140791 | -2.062328420612 |
| H | -1.769581401597 | -0.621128330597 | -2.967687778262 |
| H | -3.046794758518 | -0.344080603777 | 2.466071111399 |
| Mn | -2.596070734570 | -1.429399402548 | -0.584919762284 |
| O | -4.383315823373 | -0.369022227538 | -0.591008382977 |
| O | -6.577175997077 | -0.658896058225 | -0.890920480956 |
| N | -3.347848412351 | -2.780643169397 | 0.788053787232 |
| N | -3.719428971188 | -2.522877810338 | -2.010136448060 |
| N | -3.570579205668 | -3.967192832705 | 2.617621587522 |
| C | -5.406394960978 | -3.528186341141 | -0.517287790707 |
| C | -5.122106938809 | -2.439251518410 | -1.553055917537 |
| C | -4.521727908008 | -3.514954103520 | 0.693021424119 |
| C | -5.397968679636 | -1.045490281945 | -0.972467107625 |
| C | -4.666882019706 | -4.249469012187 | 1.837067587026 |
| C | -2.801678310831 | -3.081810376789 | 1.957200944367 |
| H | -6.449390985800 | -3.447644412052 | -0.210049449262 |
| H | -5.305670258656 | -4.492798848529 | -1.023225118571 |
| H | -5.815777174018 | -2.588416554769 | -2.381565040435 |
| H | -3.375027312414 | -4.350912358020 | 3.529576543372 |
| H | -5.431612071078 | -4.937385861640 | 2.150349579630 |
| H | -3.451121144077 | -3.496857747404 | -2.116424313265 |
| H | -3.636763517713 | -2.097403912535 | -2.929160585408 |
| H | -1.877487140806 | -2.692834174736 | 2.344922343378 |

30

| | | | |
|---|---|---|---|
| O | -0.940968386267 | -2.593848714701 | -0.829359532668 |
| O | 1.280336429220 | -2.375491258657 | -0.587273843168 |
| N | -1.678511311941 | -0.054127902990 | 0.794274391136 |
| N | -1.228222149960 | -0.262076244364 | -2.089555337303 |
| N | -1.765502325809 | 1.551923692013 | 2.275033948459 |
| C | 0.471736023894 | 0.503988531918 | -0.434985138861 |
| C | 0.104467753509 | -0.527977116776 | -1.506671012179 |
| C | -0.567112446593 | 0.746909321500 | 0.621314326302 |
| C | 0.176514697930 | -1.969240037153 | -0.919952977267 |
| C | -0.613574789169 | 1.757648463305 | 1.543582250439 |
| C | -2.380005723178 | 0.454298554217 | 1.784437848279 |
| H | 1.396873626109 | 0.146801818520 | 0.021370395452 |
| H | 0.706795299475 | 1.458593079516 | -0.915431809952 |
| H | 0.887256644149 | -0.479845239652 | -2.267277926705 |
| H | -2.110842300531 | 2.138393790420 | 3.014943171710 |
| H | 0.051470255551 | 2.579037543253 | 1.741614338264 |
| H | -1.423909070187 | 0.731048125138 | -2.151559006341 |
| H | -1.284606156519 | -0.644145201516 | -3.025516211295 |
| H | -3.334805127314 | 0.091324552512 | 2.122921299467 |
| Fe | -2.600214717882 | -1.427684552220 | -0.670580067208 |
| O | -4.264227882298 | -0.257720526535 | -0.609198710720 |

| | | | |
|---|---|---|---|
| O | -6.454112855594 | -0.497463974818 | -0.176121767321 |
| N | -3.389790435838 | -2.885270760730 | 0.789269658857 |
| N | -4.098814265548 | -2.510177816057 | -2.025131216386 |
| N | -3.180890682344 | -4.564497695247 | 2.173417090892 |
| C | -5.643209197402 | -3.378201057033 | -0.271454569995 |
| C | -5.372249954398 | -2.283187967319 | -1.308501244290 |
| C | -4.516380997521 | -3.675359357079 | 0.675134455294 |
| C | -5.386646099464 | -0.879574197113 | -0.632946073764 |
| C | -4.394149276583 | -4.731604792700 | 1.537577923190 |
| C | -2.606232071362 | -3.443873574914 | 1.686471544329 |
| H | -6.525586499535 | -3.054354492478 | 0.283697282965 |
| H | -5.917853965087 | -4.304540235698 | -0.785179441902 |
| H | -6.221083131143 | -2.288247245332 | -1.996312437192 |
| H | -2.774944199911 | -5.187901714747 | 2.849624873871 |
| H | -5.043642086091 | -5.561244041581 | 1.752160630272 |
| H | -3.912347247831 | -3.497280242938 | -2.164042838278 |
| H | -4.126788344875 | -2.072627959896 | -2.937793026772 |
| H | -1.623684711643 | -3.098783392059 | 1.957572720392 |

30_PCM

| | | | |
|---|---|---|---|
| O | -0.762566931824 | -2.422939492915 | -0.860175481576 |
| O | 1.427886742147 | -2.024715054036 | -1.080844703576 |
| N | -1.719171677561 | -0.030172279891 | 0.793942851490 |
| N | -1.472019517929 | -0.213275740234 | -2.183225924719 |
| N | -1.201402243668 | 0.899822436970 | 2.707782001653 |
| C | 0.207565739744 | 0.783423688329 | -0.667082020506 |
| C | -0.072100268425 | -0.287808276904 | -1.727603577219 |
| C | -0.526917199387 | 0.655238015750 | 0.635055452117 |
| C | 0.240390472928 | -1.699920319944 | -1.183371765605 |
| C | -0.193978361788 | 1.231239456149 | 1.831822582969 |
| C | -2.096474966991 | 0.140798498010 | 2.047986570512 |
| H | 1.278736453542 | 0.783815674583 | -0.466439816649 |
| H | -0.018944208393 | 1.757065712150 | -1.114296961275 |
| H | 0.621614436075 | -0.106311494019 | -2.551238722212 |
| H | -1.257463178287 | 1.169865980722 | 3.676572767863 |
| H | 0.643743194012 | 1.837481308794 | 2.125862501643 |
| H | -1.764887353744 | 0.752642619041 | -2.285220009380 |
| H | -1.563714981448 | -0.642703781861 | -3.097223310693 |
| H | -2.984844788744 | -0.262998592424 | 2.500595348752 |
| Fe | -2.596223142884 | -1.432850313250 | -0.659124995568 |
| O | -4.435232155322 | -0.434202300924 | -0.623101036558 |
| O | -6.640019786462 | -0.810466430843 | -0.676326408850 |

| | | | |
|---|---|---|---|
| N | -3.344991075534 | -2.918145872198 | 0.785382130233 |
| N | -3.864624003305 | -2.557187321915 | -2.142230095076 |
| N | -3.705920029428 | -3.938607907243 | 2.689298630589 |
| C | -5.403202008141 | -3.640124079684 | -0.538269696184 |
| C | -5.216582443557 | -2.508937249007 | -1.556279784862 |
| C | -4.555772193332 | -3.584003042830 | 0.698585188275 |
| C | -5.468736570476 | -1.131961678024 | -0.902069689127 |
| C | -4.790458431227 | -4.216221653824 | 1.890172402268 |
| C | -2.861918832721 | -3.155414110301 | 1.991471688734 |
| H | -6.452100379591 | -3.655068324794 | -0.243041087865 |
| H | -5.219828488612 | -4.585945779030 | -1.058946814014 |
| H | -5.984249189192 | -2.641188198745 | -2.321703931536 |
| H | -3.569199270965 | -4.257412184146 | 3.634922127042 |
| H | -5.607495873294 | -4.828604139813 | 2.226673298366 |
| H | -3.588073332782 | -3.515303839844 | -2.328975855705 |
| H | -3.853900852360 | -2.073163319093 | -3.033275140931 |
| H | -1.932380981041 | -2.782120452753 | 2.383515247208 |

30_SMD

| | | | |
|---|---|---|---|
| O | -0.657282188863 | -2.436456163443 | -0.815289664487 |
| O | 1.521817270913 | -1.956502814758 | -0.919926408376 |
| N | -1.782981725616 | -0.014259582170 | 0.725721816503 |
| N | -1.377824604995 | -0.293713573594 | -2.245957468593 |
| N | -1.244828379728 | 0.776624462341 | 2.695997577407 |
| C | 0.177172996470 | 0.806357531341 | -0.674301251261 |
| C | -0.004280233794 | -0.304668951560 | -1.717662392283 |
| C | -0.562724684605 | 0.633353508889 | 0.615634204639 |
| C | 0.323014224183 | -1.676425570822 | -1.110276160563 |
| C | -0.217746866921 | 1.121133201586 | 1.845872463370 |
| C | -2.161782714210 | 0.093611750698 | 1.987093818188 |
| H | 1.239815664683 | 0.902790065041 | -0.453817140845 |
| H | -0.128323278547 | 1.744657814407 | -1.148799118382 |
| H | 0.725563929535 | -0.124803131814 | -2.509386478259 |
| H | -1.301488250076 | 0.993014147201 | 3.679800796456 |
| H | 0.640920832616 | 1.675370287911 | 2.180465544457 |
| H | -1.672916738650 | 0.662343566530 | -2.415589019966 |
| H | -1.410979491175 | -0.778763047616 | -3.136665839466 |
| H | -3.066710758803 | -0.302671333283 | 2.413348154225 |
| Fe | -2.585038363400 | -1.454414963140 | -0.738354915604 |
| O | -4.517216799867 | -0.463525991541 | -0.483444868693 |
| O | -6.711461502214 | -0.885864905511 | -0.493956190422 |
| N | -3.262583830905 | -2.983653808574 | 0.712267234036 |

| | | | |
|---|---|---|---|
| N | -3.961378751211 | -2.451500474806 | -2.187408711794 |
| N | -3.693087007460 | -3.800568556227 | 2.698986429285 |
| C | -5.355743622968 | -3.674923863443 | -0.556377751889 |
| C | -5.276953034921 | -2.488450212910 | -1.528510658025 |
| C | -4.516086608872 | -3.574852864217 | 0.677898758867 |
| C | -5.533198343766 | -1.170625530874 | -0.784261687959 |
| C | -4.793388644053 | -4.078097405044 | 1.918547378786 |
| C | -2.799925641760 | -3.139660783606 | 1.940038080787 |
| H | -6.394769047653 | -3.806202931605 | -0.256045415060 |
| H | -5.078216308560 | -4.571806449159 | -1.119925166078 |
| H | -6.080360116506 | -2.611612769234 | -2.256942263102 |
| H | -3.577533288344 | -4.040861069979 | 3.672019950574 |
| H | -5.650746087614 | -4.601432598306 | 2.302560524009 |
| H | -3.693247645028 | -3.388505559141 | -2.471455376300 |
| H | -4.012401788980 | -1.888913143962 | -3.030624137158 |
| H | -1.850556248296 | -2.791898125595 | 2.308299313026 |

31

| | | | |
|---|---|---|---|
| O | -0.973310748908 | -2.556670109642 | -0.757054280547 |
| O | 1.218923535742 | -2.361856270296 | -1.218731816641 |
| N | -1.780120498236 | -0.220941587218 | 0.767828084922 |
| N | -1.615366292950 | -0.292774889821 | -1.968908329219 |
| N | -1.657018969085 | 1.095117380826 | 2.510755072875 |
| C | 0.266402179095 | 0.493939021487 | -0.550697262176 |
| C | -0.177210442813 | -0.481828599949 | -1.641225057562 |
| C | -0.632963607215 | 0.543300461032 | 0.649787555077 |
| C | 0.069536001870 | -1.943654238266 | -1.175967557015 |
| C | -0.548402451083 | 1.367183433020 | 1.739176226655 |
| C | -2.379483360767 | 0.139023592452 | 1.885913428268 |
| H | 1.278339509866 | 0.204785736400 | -0.261651803683 |
| H | 0.336747328787 | 1.501777102177 | -0.973188793353 |
| H | 0.454049337007 | -0.308625864694 | -2.514114360525 |
| H | -1.907066877544 | 1.550931600819 | 3.370585380838 |
| H | 0.179633353527 | 2.106698858188 | 2.019842560864 |
| H | -1.895225728947 | 0.683224335952 | -1.920125242823 |
| H | -1.788196272574 | -0.596581671837 | -2.921467421857 |
| H | -3.327074576475 | -0.232967783092 | 2.230140052686 |
| Fe | -2.596595866084 | -1.428722648634 | -0.588305619682 |
| O | -4.225644908398 | -0.296976571839 | -0.546633668677 |
| O | -6.450465768602 | -0.469994950062 | -0.824526385215 |
| N | -3.293954343063 | -2.715466861056 | 0.761869600857 |
| N | -3.698481591219 | -2.484010994724 | -1.941365166269 |

| | | | |
|---|---|---|---|
| N | -3.269769884550 | -4.127959056213 | 2.431995859352 |
| C | -5.449969601733 | -3.357576952469 | -0.411650279520 |
| C | -5.101725284751 | -2.318330303745 | -1.477844498884 |
| C | -4.449799257562 | -3.473303702982 | 0.701323019265 |
| C | -5.302857547596 | -0.887011372747 | -0.907612442133 |
| C | -4.442344982051 | -4.357369253420 | 1.745864809276 |
| C | -2.600893915282 | -3.137236451654 | 1.801441080535 |
| H | -6.432580847652 | -3.089695546139 | -0.019388025250 |
| H | -5.557990806342 | -4.339362076121 | -0.884582370001 |
| H | -5.807812688535 | -2.441770709404 | -2.300422066898 |
| H | -2.947615637477 | -4.631083226142 | 3.239881773187 |
| H | -5.145974348671 | -5.112373393138 | 2.046801949795 |
| H | -3.418037525543 | -3.460478376511 | -1.975171754000 |
| H | -3.609301849724 | -2.123930956310 | -2.885781677571 |
| H | -1.625832444452 | -2.783806944238 | 2.082783385068 |

31_PCM

| | | | |
|---|---|---|---|
| O | -0.910939211226 | -2.501765226735 | -0.759730831030 |
| O | 1.233949294648 | -2.286521959862 | -1.364411340571 |
| N | -1.763621727920 | -0.217271722014 | 0.787219386607 |
| N | -1.667146600829 | -0.278126819672 | -1.982091151116 |
| N | -1.478280031737 | 0.960202025299 | 2.611486106902 |
| C | 0.206320553527 | 0.578031277690 | -0.615170897999 |
| C | -0.219387934367 | -0.416896861384 | -1.695552135917 |
| C | -0.610236571223 | 0.536883911574 | 0.643512358487 |
| C | 0.077027685711 | -1.861948031454 | -1.248101719936 |
| C | -0.425023818809 | 1.269239579289 | 1.785100300961 |
| C | -2.264508254720 | 0.068354083306 | 1.977552745078 |
| H | 1.257576411643 | 0.396967241656 | -0.387166281640 |
| H | 0.154135344188 | 1.585605446018 | -1.038390993572 |
| H | 0.378704588543 | -0.213780143270 | -2.584696008291 |
| H | -1.637740085421 | 1.332423585300 | 3.533211784784 |
| H | 0.337991288270 | 1.972177723003 | 2.066438427488 |
| H | -1.946421986274 | 0.698460069061 | -1.956007841032 |
| H | -1.854209899311 | -0.595722221508 | -2.927959838469 |
| H | -3.167618633436 | -0.337651043297 | 2.394077587688 |
| Fe | -2.595692967460 | -1.429750946126 | -0.577304471012 |
| O | -4.286155253964 | -0.352242913715 | -0.547749305786 |
| O | -6.476833182889 | -0.534524061000 | -0.972133682575 |
| N | -3.308253026518 | -2.721107514480 | 0.782677200953 |
| N | -3.648442615801 | -2.498300718505 | -1.960249302570 |
| N | -3.439366317015 | -3.999473070057 | 2.556104633366 |

| | | | |
|---|---|---|---|
| C | -5.395795908465 | -3.437423702922 | -0.485490887769 |
| C | -5.064770106520 | -2.379631844080 | -1.539104185646 |
| C | -4.473076689462 | -3.466439922776 | 0.697813504777 |
| C | -5.315823226794 | -0.963765367565 | -0.983355622308 |
| C | -4.561725662860 | -4.261873723646 | 1.808299568344 |
| C | -2.707231688180 | -3.073006795933 | 1.907100534131 |
| H | -6.423024742690 | -3.274395460400 | -0.156697770577 |
| H | -5.381446789531 | -4.418407214786 | -0.969703205394 |
| H | -5.739595740917 | -2.531855830685 | -2.382256555496 |
| H | -3.201853664734 | -4.423567964349 | 3.437784905012 |
| H | -5.300638493864 | -4.979842883188 | 2.114904875601 |
| H | -3.372176979910 | -3.474370191710 | -2.017489377891 |
| H | -3.544537343887 | -2.124628770357 | -2.898371895436 |
| H | -1.769587689787 | -2.690431856730 | 2.265475341891 |

31_SMD

| | | | |
|---|---|---|---|
| O | -0.888435472859 | -2.490208588692 | -0.747670811145 |
| O | 1.249509856602 | -2.257234383121 | -1.351574396459 |
| N | -1.754215978435 | -0.214936434151 | 0.795807026806 |
| N | -1.678567208280 | -0.292801490981 | -1.989945829180 |
| N | -1.417875310408 | 0.926618615265 | 2.635058682843 |
| C | 0.179518638260 | 0.611182688152 | -0.641721923723 |
| C | -0.230505336776 | -0.405276979353 | -1.707726416220 |
| C | -0.603898991752 | 0.543057418078 | 0.635163802707 |
| C | 0.083529665703 | -1.831959892920 | -1.244692324382 |
| C | -0.387166054755 | 1.250571369462 | 1.785363694281 |
| C | -2.220261551997 | 0.047164956775 | 2.006779977373 |
| H | 1.242496714022 | 0.483264867776 | -0.433854352761 |
| H | 0.066094631621 | 1.608457462774 | -1.076374701000 |
| H | 0.361837557528 | -0.205102222079 | -2.601003699615 |
| H | -1.552970943663 | 1.280879002047 | 3.569481784747 |
| H | 0.385203822380 | 1.946601644164 | 2.059267746494 |
| H | -1.954260694467 | 0.685447949043 | -1.995893104404 |
| H | -1.866475619886 | -0.651286949869 | -2.921844167643 |
| H | -3.109061936563 | -0.364498626527 | 2.448925652739 |
| Fe | -2.594300280517 | -1.431293755227 | -0.569239937927 |
| O | -4.304541289040 | -0.365609916329 | -0.529031409084 |
| O | -6.485814824433 | -0.555488725736 | -0.970995545253 |
| N | -3.314642788891 | -2.728073261345 | 0.790633965923 |
| N | -3.636878904159 | -2.486209403611 | -1.966506872094 |
| N | -3.497585475757 | -3.970556395754 | 2.585487186499 |
| C | -5.375267372467 | -3.463495116761 | -0.513796895011 |

| | | | |
|---|---|---|---|
| C | -5.054132703976 | -2.388327529198 | -1.552508445406 |
| C | -4.482050573169 | -3.470268886192 | 0.690747986523 |
| C | -5.318414707227 | -0.990495328753 | -0.983235018579 |
| C | -4.602620605374 | -4.241042832273 | 1.814046365226 |
| C | -2.745546647352 | -3.060580669219 | 1.938588804670 |
| H | -6.414963675874 | -3.346430930511 | -0.206006906816 |
| H | -5.306587268568 | -4.434215536502 | -1.013156661698 |
| H | -5.723647758112 | -2.536616155263 | -2.400147041720 |
| H | -3.283606671896 | -4.379186962561 | 3.482288337476 |
| H | -5.354118937890 | -4.948170224071 | 2.116195028425 |
| H | -3.369044288089 | -3.463293692629 | -2.048420288928 |
| H | -3.529003808010 | -2.078670154275 | -2.890796706519 |
| H | -1.817184885461 | -2.678294769646 | 2.321881372856 |

32

| | | | |
|---|---|---|---|
| O | -0.970051569360 | -2.611986082793 | -0.924294861255 |
| O | 1.253443232651 | -2.357664048660 | -0.724360775240 |
| N | -1.696789810950 | -0.115535871572 | 0.790258215081 |
| N | -1.343606619442 | -0.237399339726 | -2.056736862061 |
| N | -1.740604394773 | 1.405836232944 | 2.357929908099 |
| C | 0.420446736347 | 0.496925981460 | -0.462265260499 |
| C | 0.019043277389 | -0.503439224170 | -1.549645936038 |
| C | -0.583430313940 | 0.685691382061 | 0.636925550915 |
| C | 0.132075437608 | -1.964250115153 | -1.015013660067 |
| C | -0.602780674419 | 1.643680704125 | 1.614716308441 |
| C | -2.374739163746 | 0.342160550656 | 1.820960560716 |
| H | 1.366255902063 | 0.136974820809 | -0.053428405615 |
| H | 0.623454221124 | 1.470909350377 | -0.917815470944 |
| H | 0.763335600721 | -0.419906150374 | -2.344937269269 |
| H | -2.065050546426 | 1.952391546910 | 3.136823263488 |
| H | 0.072460552823 | 2.448708079934 | 1.842704907858 |
| H | -1.566330760054 | 0.751696530483 | -2.073989578945 |
| H | -1.444460844487 | -0.591352024097 | -2.999909052114 |
| H | -3.323715596117 | -0.034206557338 | 2.161009925877 |
| Co | -2.600540308649 | -1.426650252410 | -0.663373894514 |
| O | -4.244987384028 | -0.233134401444 | -0.711046312089 |
| O | -6.442722233191 | -0.504240322859 | -0.330351201784 |
| N | -3.375034590813 | -2.819842287483 | 0.788243477884 |
| N | -3.978679045486 | -2.539427945348 | -2.004440623847 |
| N | -3.197408363561 | -4.424377069648 | 2.260599480188 |
| C | -5.596343267299 | -3.366804955961 | -0.303878218621 |
| C | -5.290441846448 | -2.305787867818 | -1.364584438514 |

| | | | |
|---|---|---|---|
| C | -4.499900867625 | -3.613557373102 | 0.690375084968 |
| C | -5.352314605690 | -0.877687017477 | -0.740561623966 |
| C | -4.397138151955 | -4.623430200694 | 1.608966363832 |
| C | -2.610045246131 | -3.331759981135 | 1.728521501222 |
| H | -6.501839258476 | -3.033390713049 | 0.206190326118 |
| H | -5.840409467496 | -4.314338159695 | -0.793793080157 |
| H | -6.102407777325 | -2.346432260009 | -2.094295139557 |
| H | -2.807144516094 | -5.011946145397 | 2.976922179691 |
| H | -5.052036684994 | -5.440903829073 | 1.851529376705 |
| H | -3.760153236308 | -3.525409386001 | -2.095685491130 |
| H | -3.961196939954 | -2.133958880214 | -2.931985616784 |
| H | -1.633668555504 | -2.972536557061 | 2.003290301889 |

32_PCM

| | | | |
|---|---|---|---|
| O | -0.812570016470 | -2.483460608836 | -0.929316018323 |
| O | 1.380386547305 | -2.098787895730 | -1.155997332843 |
| N | -1.695837999323 | -0.107249315330 | 0.798826008260 |
| N | -1.522460396565 | -0.200048220369 | -2.105885564582 |
| N | -1.297557364867 | 0.952629479202 | 2.671788037440 |
| C | 0.244084475970 | 0.710987387158 | -0.638793621688 |
| C | -0.106978737047 | -0.321110919634 | -1.713088419626 |
| C | -0.532828262512 | 0.625813784540 | 0.642395247635 |
| C | 0.193475195861 | -1.758464823423 | -1.227661833671 |
| C | -0.275355457952 | 1.285216792111 | 1.814436349429 |
| C | -2.130651061327 | 0.115730200522 | 2.026491946076 |
| H | 1.308181677554 | 0.617979174379 | -0.421713713297 |
| H | 0.106795356394 | 1.706288646570 | -1.073098397804 |
| H | 0.555269763879 | -0.133254138195 | -2.560736410220 |
| H | -1.404038470190 | 1.273821183267 | 3.620491116684 |
| H | 0.520002597530 | 1.948848311803 | 2.101916746647 |
| H | -1.806510021812 | 0.772664470178 | -2.149987458072 |
| H | -1.659306453084 | -0.584926284129 | -3.033770669459 |
| H | -3.019654165768 | -0.299087845145 | 2.466980360735 |
| Co | -2.594972298715 | -1.432423852918 | -0.630410094513 |
| O | -4.392254612493 | -0.366933904038 | -0.695839904833 |
| O | -6.599102459177 | -0.734212813224 | -0.752852515343 |
| N | -3.367373196073 | -2.841832522695 | 0.794490332056 |
| N | -3.803983185514 | -2.574140474920 | -2.071841295016 |
| N | -3.617884212685 | -3.989300643610 | 2.641505142703 |
| C | -5.435799399935 | -3.568901131735 | -0.506958942001 |
| C | -5.177825642496 | -2.475636079433 | -1.546559601336 |
| C | -4.550178350022 | -3.554373130542 | 0.704504830252 |

| | | | |
|---|---|---|---|
| C | -5.425288386589 | -1.070434311934 | -0.949786858824 |
| C | -4.715066933694 | -4.268140216355 | 1.861196141005 |
| C | -2.831581316907 | -3.129966801553 | 1.967513679391 |
| H | -6.476568057361 | -3.491609428077 | -0.192569316562 |
| H | -5.338943758997 | -4.537192792826 | -1.008184971527 |
| H | -5.914839923546 | -2.612581671077 | -2.340592078088 |
| H | -3.434173596641 | -4.359218319628 | 3.560190039829 |
| H | -5.492351825371 | -4.936822200995 | 2.184255202659 |
| H | -3.529895650010 | -3.542903020349 | -2.195428391500 |
| H | -3.749150439614 | -2.137989598623 | -2.985467725213 |
| H | -1.902671641757 | -2.745356304402 | 2.349133913494 |

32_SMD

| | | | |
|---|---|---|---|
| O | -0.735294748613 | -2.464693007226 | -0.819805830013 |
| O | 1.439944157418 | -2.009643703249 | -1.048092844792 |
| N | -1.714572957194 | -0.064804014260 | 0.795045416610 |
| N | -1.516766446918 | -0.263523885100 | -2.130689878594 |
| N | -1.201904497439 | 0.875702030035 | 2.704383911812 |
| C | 0.185744593640 | 0.770220040236 | -0.678927905193 |
| C | -0.106003483992 | -0.319777774601 | -1.714792821883 |
| C | -0.532310619080 | 0.637869231292 | 0.628806088004 |
| C | 0.234683653301 | -1.710554863410 | -1.154906458610 |
| C | -0.203397721463 | 1.219273409723 | 1.822118092069 |
| C | -2.088926058790 | 0.103669590155 | 2.051866407472 |
| H | 1.259118610161 | 0.792844692462 | -0.493030508950 |
| H | -0.069608055005 | 1.730252098350 | -1.138538379313 |
| H | 0.561310564350 | -0.147268875674 | -2.561193462054 |
| H | -1.259338600013 | 1.147315103849 | 3.674338677140 |
| H | 0.628067713768 | 1.837352408586 | 2.110100304417 |
| H | -1.807519452315 | 0.701170866255 | -2.252121410954 |
| H | -1.635415826175 | -0.727017820301 | -3.025733216119 |
| H | -2.971554721074 | -0.304153631510 | 2.512035459685 |
| Co | -2.615210595531 | -1.414898293040 | -0.621246038536 |
| O | -4.483775935152 | -0.355263299645 | -0.679087200213 |
| O | -6.670611894974 | -0.808874259711 | -0.728001417098 |
| N | -3.376942607844 | -2.836015081311 | 0.785090778310 |
| N | -3.813796351436 | -2.530901047342 | -2.084521205497 |
| N | -3.666032367019 | -3.936229548628 | 2.654836717219 |
| C | -5.410723885039 | -3.603809726715 | -0.544177750487 |
| C | -5.188900473422 | -2.481969493752 | -1.562402402087 |
| C | -4.558379217169 | -3.552798857226 | 0.687375255238 |
| C | -5.479734841913 | -1.104928546775 | -0.943433970406 |

| | | | |
|---|---|---|---|
| C | -4.747163258655 | -4.234938712897 | 1.857594982658 |
| C | -2.866974390385 | -3.093316596304 | 1.977902415924 |
| H | -6.460654669775 | -3.602595684230 | -0.252401302427 |
| H | -5.235188850695 | -4.550846625188 | -1.063787486551 |
| H | -5.919437492733 | -2.625519826717 | -2.360829944201 |
| H | -3.500877826370 | -4.282895198727 | 3.587803444165 |
| H | -5.531516917182 | -4.895776491587 | 2.180562459895 |
| H | -3.538571131575 | -3.493133379696 | -2.252204659366 |
| H | -3.760860501894 | -2.043948506296 | -2.973386720313 |
| H | -1.946360575829 | -2.701952559799 | 2.373026363083 |

33

| | | | |
|---|---|---|---|
| O | -1.248929328232 | -2.473746697456 | -0.555448815497 |
| O | 0.985454213890 | -2.589136258468 | -0.670850617873 |
| N | -1.894245778622 | 0.038564058845 | 0.633218890187 |
| N | -1.355669730347 | -0.334563744739 | -2.185196775578 |
| N | -1.729618348909 | 1.362747518795 | 2.356269738867 |
| C | 0.325896726217 | 0.433424533235 | -0.536054960937 |
| C | -0.054859799113 | -0.640721449492 | -1.557578398861 |
| C | -0.657154938106 | 0.648346873633 | 0.578006015724 |
| C | -0.085289874281 | -2.034283678424 | -0.873471961172 |
| C | -0.545091740435 | 1.479831495452 | 1.659198377812 |
| C | -2.520209725037 | 0.486715894253 | 1.702053393625 |
| H | 1.294234717483 | 0.148980668462 | -0.121778134543 |
| H | 0.477565303681 | 1.382453832797 | -1.062041435069 |
| H | 0.752412214607 | -0.679184629146 | -2.292162665248 |
| H | -1.974665963548 | 1.857239664913 | 3.196200467563 |
| H | 0.249764828351 | 2.127490853952 | 1.982076881818 |
| H | -1.472402132773 | 0.659009813399 | -2.350980293005 |
| H | -1.430348437334 | -0.801127548635 | -3.081189503211 |
| H | -3.516815956582 | 0.206936004047 | 1.993251345230 |
| Co | -2.741676067777 | -1.223648346207 | -0.613799929540 |
| O | -4.335179406184 | -0.136526291653 | -0.640196967482 |
| O | -6.560974909192 | -0.413019053464 | -0.575117314923 |
| N | -3.658913399089 | -2.545672246334 | 1.219148081660 |
| N | -3.843414832408 | -2.445934202242 | -1.755856536244 |
| N | -2.945022383442 | -4.463086515711 | 2.020231097001 |
| C | -5.606035953174 | -3.278699844098 | -0.194499386986 |
| C | -5.245220823041 | -2.261655240825 | -1.292310380879 |
| C | -4.483066676226 | -3.552589120918 | 0.752389068989 |
| C | -5.425618873005 | -0.805329471712 | -0.789147476280 |
| C | -4.041457082075 | -4.755196925834 | 1.234487196964 |

| | | | |
|---|---|---|---|
| C | -2.746500753180 | -3.126051059156 | 1.965421903057 |
| H | -6.478225641790 | -2.876209934172 | 0.325173281757 |
| H | -5.912299266466 | -4.223895160315 | -0.648944436460 |
| H | -5.947530567837 | -2.395943076547 | -2.116320280582 |
| H | -2.346251034304 | -5.130191801905 | 2.475318398048 |
| H | -4.399864575241 | -5.760269921395 | 1.100148506194 |
| H | -3.520269958603 | -3.404176199647 | -1.657244970597 |
| H | -3.759770189132 | -2.190840445432 | -2.732963385720 |
| H | -1.908191538739 | -2.636422187863 | 2.430135942219 |

33_PCM

| | | | |
|---|---|---|---|
| O | -0.724951635525 | -2.641615627605 | -0.878631341194 |
| O | 1.426292682892 | -2.165017573713 | -1.306319247855 |
| N | -1.763199218551 | -0.243461327017 | 0.776588677188 |
| N | -1.627570431316 | -0.363980139628 | -1.968641906054 |
| N | -1.510083460715 | 0.942644202964 | 2.594592477128 |
| C | 0.212286258859 | 0.566843587388 | -0.611058503208 |
| C | -0.174148767964 | -0.449208863511 | -1.683880759927 |
| C | -0.611888668541 | 0.514492678154 | 0.640591390512 |
| C | 0.215346161111 | -1.887475042659 | -1.257414331469 |
| C | -0.446156615626 | 1.251018611202 | 1.781470212780 |
| C | -2.284716912685 | 0.047625760934 | 1.958232685828 |
| H | 1.265715593345 | 0.418444819876 | -0.373493426546 |
| H | 0.131545176647 | 1.570392408713 | -1.039228291710 |
| H | 0.398761983855 | -0.211395833824 | -2.580900722686 |
| H | -1.683897716796 | 1.319377137896 | 3.512281564861 |
| H | 0.310155867499 | 1.958306317759 | 2.069222467604 |
| H | -1.931115962691 | 0.605593658963 | -1.999381176560 |
| H | -1.803785229886 | -0.741338302775 | -2.893802375147 |
| H | -3.193468632276 | -0.356421022380 | 2.363493940862 |
| Co | -2.594772686769 | -1.432387920282 | -0.570490704812 |
| O | -4.479253986839 | -0.210370498228 | -0.639817711418 |
| O | -6.662428255197 | -0.663578532117 | -0.898280551722 |
| N | -3.306955119710 | -2.693116202322 | 0.780528288416 |
| N | -3.688440645368 | -2.422431832687 | -1.932912851770 |
| N | -3.407543261069 | -3.970314746383 | 2.551337860517 |
| C | -5.400677713925 | -3.429203533885 | -0.468254513301 |
| C | -5.109683198706 | -2.354430997427 | -1.513449023424 |
| C | -4.469999071531 | -3.441805583168 | 0.707333077144 |
| C | -5.453170260911 | -0.942645281467 | -0.974071522943 |
| C | -4.539829981967 | -4.235488567115 | 1.819339791061 |
| C | -2.686434157964 | -3.044605037120 | 1.896138948127 |

| | | | |
|---|---|---|---|
| H | -6.429063537269 | -3.298668401072 | -0.131304319589 |
| H | -5.357893134845 | -4.407221342033 | -0.956700799754 |
| H | -5.762527030882 | -2.542952755748 | -2.366497853454 |
| H | -3.156273534880 | -4.393778182359 | 3.429920005862 |
| H | -5.272248459959 | -4.955794097867 | 2.135310699288 |
| H | -3.394484549991 | -3.388741504892 | -2.047106079951 |
| H | -3.594683543785 | -1.991721043685 | -2.846662900157 |
| H | -1.744216020059 | -2.661949230870 | 2.241492787504 |

33_SMD

| | | | |
|---|---|---|---|
| O | -0.675196187222 | -2.639601714043 | -0.867491670788 |
| O | 1.451162253706 | -2.137586292592 | -1.357563956648 |
| N | -1.750074780177 | -0.246945654824 | 0.780685762569 |
| N | -1.635771735998 | -0.369376750886 | -1.967223600498 |
| N | -1.445694574345 | 0.898924702402 | 2.617576732576 |
| C | 0.201956332381 | 0.582858222316 | -0.631454205138 |
| C | -0.181222525061 | -0.439658868688 | -1.699190585769 |
| C | -0.593642495358 | 0.504524327203 | 0.635396532899 |
| C | 0.232588522330 | -1.861941512536 | -1.279268439354 |
| C | -0.396059215629 | 1.212898007038 | 1.787249650500 |
| C | -2.241600224589 | 0.024832676766 | 1.981173920383 |
| H | 1.264118581761 | 0.474676326402 | -0.412314936730 |
| H | 0.072928005059 | 1.579101507983 | -1.063754087881 |
| H | 0.377801114307 | -0.195459377967 | -2.602762575727 |
| H | -1.597305959735 | 1.258550244565 | 3.547850756908 |
| H | 0.374657871749 | 1.905918597485 | 2.072577575239 |
| H | -1.931404792146 | 0.603362323557 | -1.994588672261 |
| H | -1.818679883450 | -0.749590941144 | -2.890757813618 |
| H | -3.143451374387 | -0.375349039995 | 2.406120386892 |
| Co | -2.593591295854 | -1.432837307581 | -0.566363614763 |
| O | -4.532839625881 | -0.213677451394 | -0.631536957276 |
| O | -6.697269291149 | -0.713754406094 | -0.911040320035 |
| N | -3.317086385562 | -2.692557749291 | 0.783706989918 |
| N | -3.678986936323 | -2.412652122889 | -1.935503154366 |
| N | -3.478228853131 | -3.911725447744 | 2.591357245043 |
| C | -5.381567379107 | -3.457472621575 | -0.497110867565 |
| C | -5.102423393948 | -2.368155382654 | -1.530151473827 |
| C | -4.486759129378 | -3.433610834367 | 0.703491766497 |
| C | -5.475866167078 | -0.977770028102 | -0.984895708538 |
| C | -4.594610975379 | -4.187490484995 | 1.838054408097 |
| C | -2.730998930446 | -3.015080548811 | 1.927750521069 |
| H | -6.423440541326 | -3.384153881432 | -0.186295799062 |
| H | -5.272014314032 | -4.424326260328 | -0.996772036277 |
| H | -5.742734827986 | -2.560294563151 | -2.391430521830 |
| H | -3.253609712137 | -4.310422764676 | 3.490454119409 |
| H | -5.344455014129 | -4.888440941886 | 2.157597979003 |
| H | -3.387854784240 | -3.380451742669 | -2.050156790608 |
| H | -3.581734693475 | -1.975428058383 | -2.846823318042 |
| H | -1.794494362619 | -2.636214025030 | 2.292980719678 |

34

| | | | |
|---|---|---|---|
| O | -0.945942505422 | -2.614924393907 | -0.820510288279 |
| O | 1.284780097472 | -2.341517571602 | -0.838660068611 |
| N | -1.729242292622 | -0.149465539688 | 0.778139428604 |
| N | -1.425971867441 | -0.297378007228 | -2.050031292271 |
| N | -1.718239830260 | 1.297673326897 | 2.412258663745 |
| C | 0.355815693121 | 0.504667051152 | -0.506593474457 |
| C | -0.038889456052 | -0.515952804952 | -1.576285056374 |
| C | -0.607631475811 | 0.640992686466 | 0.636266640296 |
| C | 0.134335452348 | -1.966594478144 | -1.029811512159 |
| C | -0.591099295956 | 1.551756849445 | 1.658435521566 |
| C | -2.381655627522 | 0.271051341990 | 1.839914513027 |
| H | 1.337008802130 | 0.197723724698 | -0.140520957803 |
| H | 0.485763672938 | 1.487769803206 | -0.970353458083 |
| H | 0.676386873304 | -0.412064395954 | -2.394433967229 |
| H | -2.017017280598 | 1.811409194954 | 3.223073519936 |
| H | 0.100831309857 | 2.335799518516 | 1.908188448446 |
| H | -1.676833442289 | 0.685145002369 | -2.081169589928 |
| H | -1.536541561832 | -0.671309819892 | -2.984616460239 |
| H | -3.330379884375 | -0.106312090402 | 2.178224723857 |
| Ni | -2.599793022609 | -1.426901094651 | -0.649291730784 |
| O | -4.259388146288 | -0.235513588006 | -0.602704902878 |
| O | -6.483784406977 | -0.512444260958 | -0.439836508429 |
| N | -3.342854270202 | -2.786629600620 | 0.774344906893 |
| N | -3.896827208456 | -2.477333073110 | -2.002206580132 |
| N | -3.214133146741 | -4.322710895065 | 2.319841733698 |
| C | -5.535987941175 | -3.370949147096 | -0.354712912421 |
| C | -5.235609954225 | -2.289616559097 | -1.395057110987 |
| C | -4.475473218723 | -3.569411801250 | 0.688789913655 |
| C | -5.355742775767 | -0.873160632364 | -0.752984992234 |
| C | -4.404606784230 | -4.535814214971 | 1.656077839908 |
| C | -2.600394215955 | -3.264816713262 | 1.748983546541 |
| H | -6.480352022417 | -3.088676338230 | 0.113739788638 |
| H | -5.708337841367 | -4.326292178438 | -0.860768507130 |

| | | | |
|---|---|---|---|
| H | -6.021200349423 | -2.348306459403 | -2.150807758512 |
| H | -2.846612887162 | -4.880333983695 | 3.071241302531 |
| H | -5.074525945153 | -5.333469965205 | 1.922199398377 |
| H | -3.651926375989 | -3.455841175404 | -2.110827652744 |
| H | -3.869198578684 | -2.051191064416 | -2.920439080950 |
| H | -1.624185969437 | -2.905436492672 | 2.022477932926 |

34_PCM

| | | | |
|---|---|---|---|
| O | -0.824358392013 | -2.503031477889 | -0.818874848657 |
| O | 1.358981989758 | -2.157161231515 | -1.169242986971 |
| N | -1.735364967079 | -0.131678787974 | 0.789005508900 |
| N | -1.554912563033 | -0.265295184620 | -2.086542677744 |
| N | -1.367323862989 | 0.954146047588 | 2.651105855191 |
| C | 0.215391326233 | 0.669574737449 | -0.636774583985 |
| C | -0.134251589648 | -0.376107427030 | -1.697946532949 |
| C | -0.570717416865 | 0.598509156924 | 0.639380711806 |
| C | 0.171636371264 | -1.804743727376 | -1.191091442965 |
| C | -0.331345992260 | 1.274738718567 | 1.805705342053 |
| C | -2.191598983664 | 0.109196407395 | 2.004357351749 |
| H | 1.278581420084 | 0.577420853649 | -0.414313382481 |
| H | 0.081177201937 | 1.660056880011 | -1.083043060762 |
| H | 0.519014464527 | -0.195115538692 | -2.553162783277 |
| H | -1.488341429013 | 1.287994860785 | 3.593619613573 |
| H | 0.458527469414 | 1.943828612895 | 2.095513429804 |
| H | -1.842638645328 | 0.706125757980 | -2.141985488524 |
| H | -1.690036962627 | -0.660792941440 | -3.010131294757 |
| H | -3.088182288691 | -0.299244585931 | 2.434547825143 |
| Ni | -2.601959108107 | -1.423606080423 | -0.628869405941 |
| O | -4.386382974399 | -0.341526865866 | -0.611167435377 |
| O | -6.592026638455 | -0.674092136120 | -0.791586332571 |
| N | -3.343591850114 | -2.792482901935 | 0.785909595451 |
| N | -3.770973126400 | -2.512998070307 | -2.047214712300 |
| N | -3.531660602928 | -3.999671889006 | 2.599448392061 |
| C | -5.416553410842 | -3.520229185483 | -0.501478980651 |
| C | -5.153445950828 | -2.422889344966 | -1.534935468528 |
| C | -4.514265745765 | -3.523012970349 | 0.697823594845 |
| C | -5.411724802631 | -1.022271697006 | -0.932541324114 |
| C | -4.640724586890 | -4.275391089511 | 1.834863190820 |
| C | -2.774535897708 | -3.106899800491 | 1.934982145949 |
| H | -6.453559354530 | -3.430845965362 | -0.177275655869 |
| H | -5.336800708547 | -4.486931359760 | -1.008472972228 |
| H | -5.877281052232 | -2.560816446758 | -2.339887195398 |

| | | | |
|---|---|---|---|
| H | -3.320899517589 | -4.394241626146 | 3.501874449147 |
| H | -5.397159264449 | -4.970036922589 | 2.152453715481 |
| H | -3.489465398305 | -3.480307817157 | -2.168284961795 |
| H | -3.713970398471 | -2.077776770487 | -2.961010392833 |
| H | -1.842714440798 | -2.718772031059 | 2.304817158707 |

34_SMD

| | | | |
|---|---|---|---|
| O | -0.783848726577 | -2.493257180084 | -0.809295534146 |
| O | 1.381311743091 | -2.114029967204 | -1.203454367831 |
| N | -1.740008472837 | -0.127040758936 | 0.790303774839 |
| N | -1.579091009849 | -0.275948119813 | -2.086622176698 |
| N | -1.304022484165 | 0.891695182996 | 2.676071030327 |
| C | 0.183858076760 | 0.696194942966 | -0.661227021534 |
| C | -0.153965666918 | -0.358914226693 | -1.717523534562 |
| C | -0.565808026697 | 0.589819864777 | 0.632536492120 |
| C | 0.183257259764 | -1.769806201329 | -1.210163559467 |
| C | -0.284785728695 | 1.221471566221 | 1.812716718289 |
| C | -2.158161880378 | 0.081203309924 | 2.026572248309 |
| H | 1.254698889090 | 0.654170235841 | -0.462546558294 |
| H | -0.010338398851 | 1.675884675988 | -1.108457340992 |
| H | 0.485031107935 | -0.168122996289 | -2.580827547347 |
| H | -1.395704821107 | 1.197017296571 | 3.633237438298 |
| H | 0.524857834084 | 1.867276374509 | 2.101956925030 |
| H | -1.868555432587 | 0.694917904374 | -2.151137324667 |
| H | -1.724413782489 | -0.694119590547 | -2.999714766719 |
| H | -3.048324809578 | -0.323907735476 | 2.473786329441 |
| Ni | -2.599335710111 | -1.426018976459 | -0.620885428977 |
| O | -4.424945816539 | -0.356451655749 | -0.588761958050 |
| O | -6.617435228856 | -0.738207294345 | -0.779545116916 |
| N | -3.333846811617 | -2.804426593268 | 0.786080199512 |
| N | -3.748695532436 | -2.492143714519 | -2.055307398155 |
| N | -3.603653339643 | -3.926280222832 | 2.644320107553 |
| C | -5.372529652157 | -3.561393149250 | -0.537861719987 |
| C | -5.134032061347 | -2.441062133957 | -1.553261026130 |
| C | -4.515071530680 | -3.520822819232 | 0.690674432479 |
| C | -5.424252398295 | -1.066371778180 | -0.930321016300 |
| C | -4.692212723243 | -4.216827049385 | 1.854602737261 |
| C | -2.810692101600 | -3.075639784874 | 1.969144279063 |
| H | -6.422344520358 | -3.544293936905 | -0.245670246800 |
| H | -5.210614751803 | -4.510573921801 | -1.057840527385 |
| H | -5.849460117614 | -2.581516705515 | -2.364762808351 |
| H | -3.429518400800 | -4.283011112456 | 3.571817329683 |

| | | | |
|---|---|---|---|
| H | -5.472343922938 | -4.883026185320 | 2.176691131858 |
| H | -3.465565603195 | -3.455468384028 | -2.204801910357 |
| H | -3.687988247978 | -2.019147597928 | -2.951245253710 |
| H | -1.886904878764 | -2.688201401782 | 2.360296929300 |

35

| | | | |
|---|---|---|---|
| O | -0.701823790054 | -2.755017836712 | -0.985362075476 |
| O | 1.501508573198 | -2.447208328857 | -0.734352121861 |
| N | -1.116093174636 | -0.574195783769 | 1.530145881720 |
| N | -1.167352548406 | -0.336179569412 | -1.884020396165 |
| N | -2.482786635435 | 1.133392066288 | 1.815119814271 |
| C | 0.490358268522 | 0.371634278190 | -0.163043906405 |
| C | 0.204753097005 | -0.554953819154 | -1.365565469475 |
| C | -0.665739553540 | 0.490581842406 | 0.772460137398 |
| C | 0.380111249205 | -2.047041180397 | -0.988852123005 |
| C | -1.515610236794 | 1.554371167825 | 0.933455635676 |
| C | -2.209978947324 | -0.156415422597 | 2.125143269868 |
| H | 1.371025259353 | -0.027759081245 | 0.340699878821 |
| H | 0.743774659860 | 1.367448981026 | -0.534196504315 |
| H | 0.946462754512 | -0.342381318575 | -2.136967944262 |
| H | -3.354338352626 | 1.601744550900 | 1.998389108237 |
| H | -1.517510636893 | 2.543939467467 | 0.511471591085 |
| H | -1.541416958668 | 0.579347894197 | -1.654840711750 |
| H | -1.209920804133 | -0.469945382730 | -2.886622885441 |
| H | -2.846671782392 | -0.743945611835 | 2.765405386017 |
| Cu | -2.342782352136 | -1.710548939684 | -0.911164187015 |
| O | -3.692996527954 | -0.301781463365 | -0.585980100918 |
| O | -5.782835863925 | -0.112248101512 | 0.185220403489 |
| N | -3.107345628259 | -3.033948026492 | 0.426968210787 |
| N | -4.270102092727 | -2.342844110308 | -2.210106842819 |
| N | -3.079478492487 | -4.326457338898 | 2.176919367131 |
| C | -5.556403921946 | -3.108846518039 | -0.238063588198 |
| C | -5.305906279664 | -1.969679000868 | -1.229855688368 |
| C | -4.397808873771 | -3.471018856269 | 0.643337598707 |
| C | -4.914893779548 | -0.674558310302 | -0.468965611484 |
| C | -4.390511333856 | -4.282489816753 | 1.744454440768 |
| C | -2.335188410858 | -3.561327935422 | 1.354959008682 |
| H | -6.399915578359 | -2.811357240549 | 0.385265451288 |
| H | -5.876134078080 | -3.995725981009 | -0.796542883307 |
| H | -6.263946362817 | -1.754648777060 | -1.711009620456 |
| H | -2.731532405188 | -4.844950684612 | 2.964922700165 |
| H | -5.178076076166 | -4.818397924719 | 2.242551242946 |

| | | | |
|---|---|---|---|
| H | -4.399184013979 | -3.292028003774 | -2.541547646675 |
| H | -4.312369199572 | -1.729470791566 | -3.014929135121 |
| H | -1.276796849441 | -3.391468931810 | 1.444674275477 |

35_PCM

| | | | |
|---|---|---|---|
| O | -0.757164663343 | -2.684523456270 | -0.928269748095 |
| O | 1.444340725944 | -2.350640598960 | -1.061125674363 |
| N | -0.669259389370 | -0.570350087329 | 1.630769293636 |
| N | -1.403753712356 | -0.294913022172 | -1.829617782849 |
| N | -1.978179953632 | 1.085502152180 | 2.267911295827 |
| C | 0.461774742366 | 0.468621802389 | -0.375499278081 |
| C | 0.026678447933 | -0.484033144961 | -1.505299245613 |
| C | -0.474106076483 | 0.508017838631 | 0.786288229680 |
| C | 0.280298118753 | -1.956819837812 | -1.133463576332 |
| C | -1.287089997432 | 1.542547201776 | 1.172187964757 |
| C | -1.576664915754 | -0.186429992552 | 2.504208320805 |
| H | 1.459794882663 | 0.161274092820 | -0.059826912858 |
| H | 0.547406248382 | 1.475230553344 | -0.788095023397 |
| H | 0.645333199864 | -0.268709587984 | -2.376972170033 |
| H | -2.665793764371 | 1.599441753688 | 2.794148274091 |
| H | -1.427610616637 | 2.532564926303 | 0.775912667811 |
| H | -1.733949071592 | 0.632302121241 | -1.582340035483 |
| H | -1.570346749192 | -0.423662206309 | -2.821651144778 |
| H | -1.972755804711 | -0.778075253949 | 3.312831431773 |
| Cu | -2.464810876477 | -1.699158181553 | -0.805569322497 |
| O | -3.969401150325 | -0.410908035083 | -0.476198508462 |
| O | -6.192713053071 | -0.254411950029 | -0.403892361125 |
| N | -3.217747635495 | -2.998078381440 | 0.530590638014 |
| N | -4.064973085940 | -2.427238830310 | -2.292948699171 |
| N | -3.430950533051 | -4.100324574499 | 2.397044520772 |
| C | -5.520989981232 | -3.318539022087 | -0.509402582557 |
| C | -5.273090159002 | -2.167832492119 | -1.494597156018 |
| C | -4.508663326060 | -3.490272828297 | 0.584307818598 |
| C | -5.151706765151 | -0.828389435652 | -0.734522016240 |
| C | -4.647624499186 | -4.179578610361 | 1.757786945141 |
| C | -2.594544285960 | -3.380263860458 | 1.630714544428 |
| H | -6.502102935544 | -3.172000468171 | -0.058041132258 |
| H | -5.587408328558 | -4.244177758433 | -1.090092707029 |
| H | -6.163205497101 | -2.088054382992 | -2.121920749688 |
| H | -3.204193262537 | -4.507008776665 | 3.290250133893 |
| H | -5.481540870064 | -4.712225453762 | 2.177425845334 |
| H | -4.040451463838 | -3.391192368368 | -2.606276747952 |

| | | | |
|---|---|---|---|
| H | -4.059302586272 | -1.846964632855 | -3.124166591455 |
| H | -1.578989036146 | -3.137105050918 | 1.886985201800 |

35_SMD

| | | | |
|---|---|---|---|
| O | -0.331695577201 | -2.787492386113 | -0.541885984963 |
| O | 1.823919720381 | -2.221532250182 | -0.551759474492 |
| N | -0.449232658696 | -0.330937162920 | 1.770512986698 |
| N | -1.055926836718 | -0.838848146635 | -2.168485453581 |
| N | -2.571667194237 | 0.274624622289 | 1.773855104202 |
| C | 0.261016357425 | 0.478142623493 | -0.516284779252 |
| C | 0.269008239066 | -0.688234771089 | -1.527316026629 |
| C | -0.733742351835 | 0.326655580426 | 0.584492661560 |
| C | 0.637387204264 | -1.997379555693 | -0.828862264878 |
| C | -2.049764673105 | 0.707348018970 | 0.579199108423 |
| C | -1.579752375395 | -0.350734221475 | 2.450186503001 |
| H | 1.267002758069 | 0.565128852690 | -0.106187690242 |
| H | 0.053800121067 | 1.390768094418 | -1.078209906019 |
| H | 1.031352716514 | -0.477879376520 | -2.276838420976 |
| H | -3.536975963465 | 0.352229555306 | 2.057639112184 |
| H | -2.645973041168 | 1.225164947943 | -0.148573451950 |
| H | -1.505419579584 | 0.064118021287 | -2.291620455368 |
| H | -0.949668227344 | -1.253038308926 | -3.090030189992 |
| H | -1.727317578149 | -0.784282862361 | 3.425088160401 |
| Cu | -2.154109864019 | -2.112663469671 | -1.013771913999 |
| O | -5.035422437424 | 0.570777200705 | -0.638274083711 |
| O | -6.099138897069 | -0.734810974822 | 0.838573244418 |
| N | -3.014888853239 | -3.142784671798 | 0.451520419039 |
| N | -3.939081954047 | -1.572445475170 | -1.876066938864 |
| N | -3.367068055735 | -3.869312987593 | 2.474667419120 |
| C | -5.333469471345 | -3.120569956040 | -0.516384520430 |
| C | -5.226961085723 | -1.742799255345 | -1.163981356591 |
| C | -4.373795385271 | -3.359932851580 | 0.593467956071 |
| C | -5.460369432592 | -0.538876469836 | -0.223971434803 |
| C | -4.599656071986 | -3.814529702880 | 1.859473444893 |
| C | -2.437422417898 | -3.451658799371 | 1.603671096426 |
| H | -6.345978994594 | -3.253635800616 | -0.140935786370 |
| H | -5.183993692422 | -3.869665505520 | -1.300165164165 |
| H | -6.033567455502 | -1.674188073176 | -1.901815382278 |
| H | -3.188716798931 | -4.165130679529 | 3.422899378903 |
| H | -5.506040230961 | -4.095067397813 | 2.364575450972 |
| H | -3.970489961904 | -2.079735795265 | -2.756922743040 |
| H | -3.853558230843 | -0.582146612883 | -2.096647279616 |

| | | | |
|---|---|---|---|
| H | -1.388079448372 | -3.391023836699 | 1.824742615933 |

36

| | | | |
|---|---|---|---|
| O | -0.983227079069 | -2.651114864700 | -0.934096875702 |
| O | 1.232436340803 | -2.441093273278 | -0.634479944038 |
| N | -1.703502201384 | -0.134770851321 | 0.816683602673 |
| N | -1.249936186975 | -0.242122027049 | -2.060049654287 |
| N | -1.876817306150 | 1.518358182444 | 2.233677904430 |
| C | 0.459276499063 | 0.429055856165 | -0.374543959032 |
| C | 0.077870297698 | -0.552711974186 | -1.489146398438 |
| C | -0.609557836290 | 0.689459315999 | 0.644484029314 |
| C | 0.130411328237 | -2.023565839739 | -0.966999491121 |
| C | -0.710068593399 | 1.731081663232 | 1.526879632206 |
| C | -2.449283759185 | 0.390514134658 | 1.764341060121 |
| H | 1.347166021203 | 0.009062395220 | 0.102265009072 |
| H | 0.759064822231 | 1.385868815580 | -0.811646063504 |
| H | 0.859595960361 | -0.487538645865 | -2.248981502434 |
| H | -2.261473631812 | 2.122848758683 | 2.938980755731 |
| H | -0.073800854904 | 2.577539016337 | 1.713128634258 |
| H | -1.437474956348 | 0.753853308425 | -2.089898774665 |
| H | -1.319659726816 | -0.599010764144 | -3.004976943547 |
| H | -3.406602596806 | 0.017613356477 | 2.084170470725 |
| Zn | -2.603569779975 | -1.425368619602 | -0.715703765934 |
| O | -4.234027785223 | -0.192939165053 | -0.706860261560 |
| O | -6.412346657404 | -0.427653468951 | -0.214063948577 |
| N | -3.360587738393 | -2.806287927052 | 0.814491089530 |
| N | -4.079986659439 | -2.529196885343 | -1.995831615202 |
| N | -3.066823066097 | -4.532340937535 | 2.120212880194 |
| C | -5.626240263742 | -3.305723416886 | -0.201818705182 |
| C | -5.347809789080 | -2.257396545033 | -1.285697231496 |
| C | -4.469807161497 | -3.619914921961 | 0.699761786023 |
| C | -5.347413633677 | -0.820873115257 | -0.673058932002 |
| C | -4.294368050455 | -4.706920524918 | 1.513037451300 |
| C | -2.533959344534 | -3.381196176076 | 1.660836195349 |
| H | -6.467596891019 | -2.920060595659 | 0.377348270087 |
| H | -5.963912347343 | -4.236550461801 | -0.666617917010 |
| H | -6.197203914739 | -2.278996448611 | -1.971820126951 |
| H | -2.622593518706 | -5.173350758523 | 2.754718257423 |
| H | -4.915260551970 | -5.561905028222 | 1.711830645035 |
| H | -3.898463536500 | -3.521026637270 | -2.102387963097 |
| H | -4.098034259831 | -2.116478081052 | -2.920197009532 |
| H | -1.549869270815 | -3.025526688123 | 1.911603369865 |

36_PCM

| | | | |
|---|---|---|---|
| O | -0.760699369854 | -2.483806088347 | -0.853183779443 |
| O | 1.427255782107 | -2.064726148255 | -1.077555520922 |
| N | -1.735508855251 | -0.070187959117 | 0.783455061344 |
| N | -1.488069276306 | -0.256184522005 | -2.157545667849 |
| N | -1.280400803493 | 0.917462708212 | 2.680578131008 |
| C | 0.208321283767 | 0.731990892102 | -0.657593440977 |
| C | -0.084573251259 | -0.339510853039 | -1.712590462278 |
| C | -0.549745023016 | 0.627026822582 | 0.632775787887 |
| C | 0.231359705152 | -1.755142672285 | -1.171955695208 |
| C | -0.256713020617 | 1.241077159899 | 1.820808910300 |
| C | -2.148941339128 | 0.129214035571 | 2.021508698566 |
| H | 1.276545512599 | 0.706961576591 | -0.443649264660 |
| H | 0.010325097034 | 1.709034294577 | -1.110594967271 |
| H | 0.599964031959 | -0.162779452816 | -2.544310271924 |
| H | -1.364166718253 | 1.212347140140 | 3.640104077689 |
| H | 0.563582118900 | 1.869047786051 | 2.117994341490 |
| H | -1.777120102778 | 0.709838232534 | -2.269000396402 |
| H | -1.590661335726 | -0.698110598293 | -3.064166826052 |
| H | -3.044346295278 | -0.272750101960 | 2.461136462868 |
| Zn | -2.600047931191 | -1.428285760411 | -0.654138435205 |
| O | -4.445712338337 | -0.367466200293 | -0.615360669299 |
| O | -6.647005970232 | -0.773576289647 | -0.665364923218 |
| N | -3.331617345047 | -2.869847122212 | 0.776539930755 |
| N | -3.851567821130 | -2.513084999303 | -2.113174237903 |
| N | -3.617396280559 | -3.960122157761 | 2.650505338306 |
| C | -5.405780226911 | -3.587483903069 | -0.520723485649 |
| C | -5.207519442203 | -2.457139948591 | -1.535663082733 |
| C | -4.531230686225 | -3.555204917137 | 0.697846715470 |
| C | -5.466095043173 | -1.076055197767 | -0.886161768195 |
| C | -4.718055800651 | -4.233386856158 | 1.872316755102 |
| C | -2.807596007944 | -3.138268551585 | 1.958402117904 |
| H | -6.449468001553 | -3.575791514700 | -0.207548459037 |
| H | -5.254773464092 | -4.536933386994 | -1.044862837568 |
| H | -5.967203155193 | -2.586254905272 | -2.308922813746 |
| H | -3.447491489674 | -4.308848768677 | 3.580120538469 |
| H | -5.512042920418 | -4.875137420330 | 2.208948936903 |
| H | -3.576736575072 | -3.470705875085 | -2.304874603988 |
| H | -3.831582253042 | -2.021050341760 | -2.999515533771 |
| H | -1.872943067889 | -2.762537975380 | 2.334989299271 |

36_SMD

| | | | |
|---|---|---|---|
| O | -0.546278963047 | -2.521687821988 | -0.643487027037 |
| O | 1.604861029713 | -1.905106454728 | -0.721726685298 |
| N | -1.800281210003 | -0.014255594036 | 0.737515108151 |
| N | -1.375835557202 | -0.508337889967 | -2.188607856800 |
| N | -1.256186213845 | 0.842327467238 | 2.673808791939 |
| C | 0.128585171500 | 0.774971556680 | -0.712328178194 |
| C | -0.001094071553 | -0.420841520425 | -1.664797222895 |
| C | -0.590598004541 | 0.645169630347 | 0.592262468558 |
| C | 0.386344638459 | -1.728856698032 | -0.955942312907 |
| C | -0.243191829375 | 1.176483538887 | 1.802841443899 |
| C | -2.170503087006 | 0.124887730839 | 1.998050242614 |
| H | 1.185385794647 | 0.944363869605 | -0.510278146508 |
| H | -0.233279688439 | 1.659747523261 | -1.246282155149 |
| H | 0.711860152212 | -0.271241177638 | -2.477500782414 |
| H | -1.306218985352 | 1.088131822342 | 3.651122518899 |
| H | 0.610358366563 | 1.753322372214 | 2.110847246226 |
| H | -1.679108488720 | 0.404988428797 | -2.513121806239 |
| H | -1.400143142684 | -1.132030532146 | -2.989327998376 |
| H | -3.065285237217 | -0.272199801637 | 2.444449762023 |
| Zn | -2.646761020118 | -1.387147004865 | -0.683801048305 |
| O | -4.618359173423 | -0.354712894473 | -0.419365490940 |
| O | -6.795060728401 | -0.869460184874 | -0.426571644153 |
| N | -3.270328158226 | -2.911385477228 | 0.676242464866 |
| N | -3.986746777352 | -2.296949288474 | -2.156604278246 |
| N | -3.596616863805 | -3.891702618433 | 2.602863330407 |
| C | -5.365900338763 | -3.606388375557 | -0.586784564998 |
| C | -5.306626589619 | -2.382396344234 | -1.510499610158 |
| C | -4.497235835396 | -3.554839273658 | 0.630450378067 |
| C | -5.602829218601 | -1.093216031570 | -0.726795790209 |
| C | -4.707462225881 | -4.163865611396 | 1.835950156107 |
| C | -2.758823230708 | -3.134375675399 | 1.874128413015 |
| H | -6.398185053250 | -3.751660443833 | -0.270536928434 |
| H | -5.098608054083 | -4.480222187358 | -1.189935431689 |
| H | -6.098773755387 | -2.495920529767 | -2.252382671602 |
| H | -3.438838513782 | -4.198600798004 | 3.551014172623 |
| H | -5.525345840236 | -4.757769055518 | 2.202738467923 |
| H | -3.706828193923 | -3.210390192546 | -2.499755553288 |
| H | -4.030707003552 | -1.675052361660 | -2.957574323137 |
| H | -1.814811779579 | -2.770161940765 | 2.239296501701 |
